# The value of serum cystatin c in predicting acute kidney injury after cardiac surgery: A systematic review and meta-analysis

**DOI:** 10.1371/journal.pone.0310049

**Published:** 2024-11-20

**Authors:** PeiQiang Peng, Xiao Chen Fu, YueTing Wang, XuFei Zheng, Linfang Bian, Nuer Zhati, Shuang Zhang, Wei Wei

**Affiliations:** 1 Department of Rehabilitation Medicine Technology, School of Nursing, Jilin University, Changchun, Jilin Province, China; 2 Urology surgery, The first hospital of Jilin University, Changchun, Jilin Province, China; University of Montenegro-Faculty of Medicine, MONTENEGRO

## Abstract

**Objective:**

This study aims to review relevant research and assess the diagnostic value of serum cystatin C (CysC) for post-cardiac surgery acute kidney injury (PCSAKI).

**Method:**

We searched databases (PubMed, Embase, Cochrane, WanFang, CNKI, VIP) for literature published up to January 10, 2024. Quality was assessed using Quality Assessment of Diagnostic Accuracy Studies-2 (QUADAS-2). Extracted data from eligible studies and summarized sensitivity, specificity, and area under the curve (AUC) values.

**Results:**

A total of 24 studies involving 3,427 patients were included. The estimated diagnostic sensitivity of CysC for PCSAKI was 0.67 (95% CI, 0.57–0.76), with a specificity of 0.87 (95% CI, 0.81–0.91). The positive likelihood ratio (+LR) was 5.17 (95% CI, 3.45–7.73), and the negative likelihood ratio (-LR) was 0.38 (95% CI, 0.28–0.51). The diagnostic odds ratio (DOR) was 14 (95% CI, 7–26), the diagnostic score (DS) was 2.62 (95% CI, 1.99–3.24), and AUC was 0.86 (95% CI, 0.83–0.89). The sub-analysis results indicate that gender distribution, serum storage temperature, CysC detection method, and detection time all have a significant impact on sensitivity and specificity.

**Conclusion:**

CysC has high specificity and good sensitivity in diagnosing PCSAKI during the perioperative period, with better detection results 24 hours before surgery, making it suitable for early detection. However, whether and how CysC is commonly used in clinical diagnosis still requires further research and clinical trials.

## Introduction

Acute kidney injury (AKI) is a sudden loss of kidney function, characterized by elevated serum creatinine and blood urea nitrogen levels. It commonly occurs after cardiac surgery with cardiopulmonary bypass, with an incidence of 30%-40%. The duration and severity of AKI are closely linked to poor patient outcomes, increasing morbidity and mortality. Even with recovery of kidney function, some patients may develop subclinical or subacute kidney disease (AKD), leading to progressive renal disease [1–3].

Despite the advances in AKI research, current clinical diagnostic standards remain controversial, primarily relying on elevated serum creatinine or reduced urine output [4–6]. However, these standards are influenced by non-renal factors such as muscle mass and surgical environment, often leading to delayed and inaccurate diagnoses [7]. Therefore, there is an urgent need for more effective diagnostic markers for acute kidney injury, especially post-cardiac surgery.

Cystatin C (CysC) has been found to be a crucial predictive biomarker for detecting acute kidney injury following cardiac surgery [8]. It is a 13-kDa endogenous cysteine protease inhibitor produced by nucleated cells at a constant rate, freely filtered in the glomerulus, and metabolized without increases due to urinary tract infections or chronic non-renal diseases like malignancies [9]. Thus, its serum concentration provides a highly sensitive estimate of glomerular filtration rate [10].

So far, despite several studies using serum CysC to predict acute kidney injury after cardiac surgery, its effectiveness remains controversial due to differences in detection time, methods, sample sizes, etc. [11]. Therefore, this study aims to systematically evaluate the diagnostic value of serum CysC for PCSAKI through meta-analysis. We opt for a bivariate model as our primary analytical approach to enhance accuracy and minimize heterogeneity effects [12].

## Methods

This meta-analysis was carried out according to the Preferred Reporting Items for Systematic Reviews and Meta-Analyses (PRISMA) guidelines [13].

### Data sources and search strategy

Conducted a comprehensive search for literature published on PubMed, Embase, Cochrane Library, Wanfang, CNKI, and VIP databases up to January 10, 2024. We employed a search strategy to identify all trials using the following keywords: serum cystatin C or CysC, post-cardiac surgery, acute kidney injury, or renal failure. (see S1 Table for details). Additionally, we scanned the reference lists of all included studies and relevant reviews. The search was independently conducted by two researchers.

### Study selection

We included all retrieved articles without restrictions on sample size or language. Initially, studies meeting the following criteria were identified:(1) Included articles with prospective cohort, case-control, or cross-sectional designs exploring serum CysC performance in PCSAKI detection. (2) Encompassed studies allowing the calculation of estimated sensitivity and specificity of serum CysC in diagnosing PCSAKI.

Two reviewers checked titles and abstracts of all citations using EndNote, followed by a full-text retrieval and reevaluation. The reference lists of reviewed articles were examined to ensure no omission of relevant studies. Any discrepancies were resolved by a third researcher.

### Data extraction

A reviewer used a standardized form to extract information from each eligible study. The following information was extracted: (1) Study details: first author, publication year, country of origin, study design, sample size, and population characteristics; (2) Characteristics of study subjects: age, gender, baseline serum creatinine; (3) AKI information: AKI criteria and the number of AKI patients; (4) Serum CysC: measurement timing, measurement method, and optimal cutoff value; (5) Outcome information: sensitivity and specificity, true positive (TP), true negative (TN), false positive (FP), false negative (FN), and AUC value. If a study proposed more than one cutoff threshold, we used the threshold with the highest product of sensitivity and specificity.

### Quality assessment of evidence

The methodological quality of these studies was independently assessed by two authors. We utilized the QUADAS-2 tool [14] to evaluate the quality of each trial. Risk of bias comprises four domains: patient selection, index test, reference test, and flow and timing. Applicability concerns encompass three domains: patient selection, index test, and reference test. Any discrepancies were resolved by a third researcher.

### Data synthesis and analysis

We used a bivariate model for diagnostic test studies, offering stability with minimal additional information. This approach is especially valuable in situations with limited data, where the prior for the covariance matrix of the bivariate structure plays a crucial role, enhancing precision sensitivity and specificity, along with all hyperparameters and covariates, were accurately derived directly from the bivariate model, eliminating the need for Markov Chain Monte Carlo (MCMC) sampling [12]. Moreover, univariate estimates of sensitivity and specificity, with 95% confidence intervals (CIs), as well as the aggregated summary receiver operating characteristic (SROC) curve, can all be directly utilized for interpretation.

Calculate the 95% CI for the AUC of the SROC. Summarized +LR and -LR are derived from the summary estimates of sensitivity and specificity, respectively. We also assessed funnel plot asymmetry to effectively evaluate the degree and impact of publication and selective reporting bias in diagnostic accuracy studies. Subgroup analysis was performed considering study design, age range, gender ratio, sample storage temperature, diagnostic and testing criteria, and testing time.

## Results

### Search results

**Fig 1:**
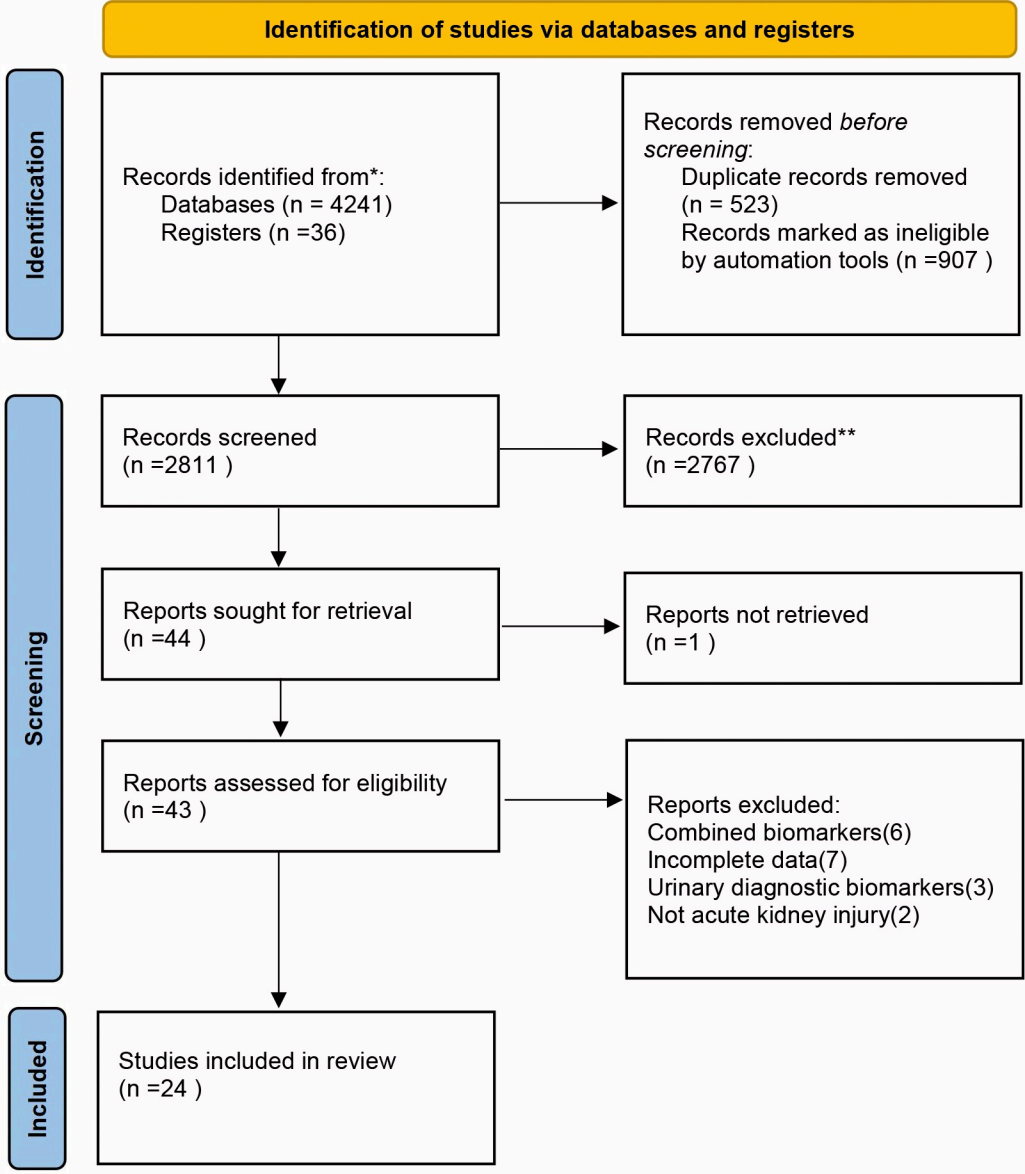
PRISMA flowchart of the literature search.

The initial search identified 4,277 records. After removing 523 studies due to duplicates, and further excluding 907 studies using automated tools, we screened the titles and abstracts of the remaining 2,811 studies. 44 studies underwent full-text review, with 24 ultimately included in the meta-analysis [15–38] (Fig 1).

### Study and patient characteristics

This study included 24 studies with a total of 3427 patients. Technical features of individual studies are in Table 1. All these studies, published between 2008 and 2023, come from diverse countries and feature varied research designs, sample sizes (ranging from 40 to 628 individuals), and population settings. It’s worth noting that these articles use different definitions for AKI. The meta-analysis includes 22 prospective cohort studies and 2 retrospective case-control studies. Twenty studies used immunofluorescence to measure serum cystatin C levels, and the other four used commercial ELISA.

**Table 1:** Characteristics of studies included in the meta-analysis.

Study	Country	Research type	Patients					Sample		CysC Assay	Baseline SCr (mg/dL)	AKI criteria
			type	age(y)	Men(%)	AKI Diabetes(%)	size(AKI)	source	Storage(°C)			
Koyner J 2008	United States	Prospective study	Post-cardiac surgery	63±13	70.8	21%	72(34)	Serum	-80	ELISA kit	1.25±NA	RIFLE
Haase M 2009	Australia	Prospective study	Post-cardiac surgery	69.55±11.55	61	59%	100(46)	Serum	-80	Immunonephelometric	NA	AKIN
Haase-Fielitz 2009	Australia	Prospective study	Post-cardiac surgery	70.1±12.1	61.54	43%	73(1))	Serum	-80	Immunonephelometric	NA	RIFLE
Che M 2010	China	Retrospective study	Post-cardiac surgery	62.9±13.7	68.96	NA	29(14)	Serum	-80	Immunonephelometric	0.827±0.135	RIFLE-R
Krawczeski C 2010	United States	Prospective study	Post-cardiac surgery	4.2±5.6	53.47	NA	374(119)	Serum	-70	Immunonephelometric	0.435±0.235	AKIN
Wald R 2010	Canada	Prospective study	Post-cardiac surgery	67±11.8	68.67	36%	150(47)	Serum	-80	Immunonephelometric	1±0.1	AKIN
Ristikankare A 2010	Finland	Prospective study	Post-cardiac surgery	77±3	54.54	34%	110(62)	Serum	NA	Immunonephelometric	NA	RIFLE
Seitz S 2013	Germany	Prospective study	Post-cardiac surgery	1d to 44.3y	54.68	NA	139(76)	Serum	-80	Immunonephelometric	0.38±0.21	RIFLE
Yu CJ 2013	China	Prospective study	Post-cardiac surgery	61±12	40.5	NA	79(34)	Serum	-80	Immunonephelometric	0.773±0.264	AKIN
Liebetrau C 2013	Germany	Prospective study	Post-cardiac surgery	69.5±12.2	68.79	33%	141(47)	Serum	-80	Immunonephelometric	0.919±0.334	KDIGO
Peco-Antić A 2013	Serbia	Prospective study	Post-cardiac surgery	1.6±3.19	58.03	NA	112(18)	Serum	-80	ELISA kit	0.6±0.124	RIFLE
Zheng JY 2013	China	Prospective study	Post-cardiac surgery	12.55±13.95	69.77	NA	43(21)	Serum	-80	Immunonephelometric	0.362±0.169	AKIN
Magro MC 2013	Brazil	Prospective study	Post-cardiac surgery	50±11	61.2	NA	121(16)	Serum	NA	Immunonephelometric	NA	RIFLE
Prowle JH 2015	Australia	Prospective study	Post-cardiac surgery	70±11.11	69	8%	93(25)	Serum	-70	Immunonephelometric	1.086±0.31	RIFLE
Hu XH 2015	China	Prospective study	Post-cardiac surgery	4.69±1.23	57	NA	100(26)	Serum	-40	Immunonephelometric	NA	AKIN
Yong ZZ 2017	China	Retrospective study	Post-cardiac surgery	65.5±5.5	78.8	38%	495(91)	Serum	NA	Immunonephelometric	0.91±0.13	AKIN
Kararmaz A 2019	Turkey	Prospective study	Post-cardiac surgery	56.9±20.2	21.4	NA	42(22)	Serum	NA	Immunonephelometric	0.855±0.335	KDIGO
Wang XD 2020	China	Prospective study	Post-cardiac surgery	63±11.85	64.17	24%	628(178)	Serum	-80	Immunonephelometric	0.807±0.235	KDIGO
Zheng XF 2021	China	Prospective study	Post-cardiac surgery	57±16.5	60	23%	145(90)	Serum	-80	Immunonephelometric	1.25±0.4	AKIN
Szymanowicz W 2021	Poland	Prospective study	Post-cardiac surgery	69±13	50	39%	114(18)	Serum	-70	Immunonephelometric	1±0.29	KDIGO
Lakhal K 2021	France	Prospective study	Post-cardiac surgery	78.5±8.15	49.2	19%	65(27)	Serum	-80	Immunonephelometric	NA	KDIGO
Kalisnik JM 2022	Germany	Prospective study	Post-cardiac surgery	73.1±11.1	36.97	22%	119(51)	Serum	-20	ELISA kit	0.785±0.335	KDIGO
Zakaria M 2022	Egypt	Prospective study	Post-cardiac surgery	3.65±NA	52.5	NA	40(12)	Serum	NA	Immunonephelometric	0.8±0.12	AKIN
Abadeer M 2023	United States	Prospective study	Post-cardiac surgery	3.7±4.3m	67.44	NA	43(18)	Serum	-80	ELISA kit	NA	KDIGO

Note: NA, Not Available

### Quality assessment

**Fig 2:**
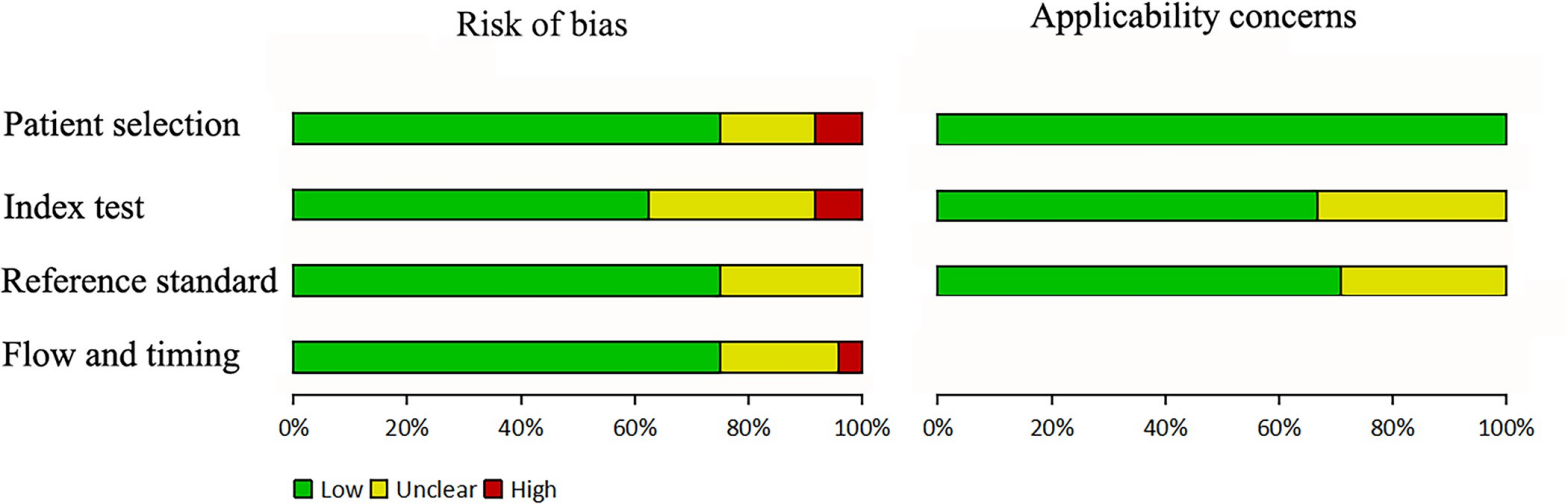
Overall level of risk of bias and applicability concerns.

The methodological quality of the studies according to the QUADAS-2 is summarized in Fig 2 and Table 2. The literature included in this study exhibits an overall low risk of bias and applicability concerns.

**Table 2:** Quality assessment of the included studies.

Studys	Risk of bias				Applicability concerns		
	patient selection	Index test	Reference standard	Flow and timing	patient selection	Index test	Reference standard
Koyner J 2008	High	Low	Unclear	Low	Low	Unclear	Unclear
Haase M 2009	Unclear	Unclear	Low	Low	Low	Low	Low
Haase-Fielitz 2009	Unclear	High	Unclear	Low	Low	Low	Unclear
Che M 2010	High	Low	Unclear	Low	Low	Low	Unclear
Krawczeski C 2010	Low	Low	Low	Low	Low	Low	Low
Wald R 2010	Low	Unclear	Low	Low	Low	Low	Low
Ristikankare A 2010	Low	Unclear	Unclear	Low	Low	Low	Unclear
Seitz S 2013	Low	Low	Low	Unclear	Low	Low	Unclear
Yu CJ 2013	Low	Unclear	Low	Low	Low	Low	Low
Liebetrau C 2013	Low	Low	Low	Unclear	Low	Unclear	Low
Peco-Antić A 2013	Low	Low	Unclear	Low	Low	Low	Unclear
Zheng JY 2013	Low	High	Low	High	Low	Low	Low
Magro MC 2013	Unclear	Low	Low	Low	Low	Low	Low
Prowle JH 2015	Unclear	Low	Unclear	Low	Low	Unclear	Unclear
Hu XH 2015	Low	Low	Low	Low	Low	Unclear	Low
Yong ZZ 2017	Low	Low	Low	Unclear	Low	Low	Low
Kararmaz A 2019	Low	Low	Low	Low	Low	Low	Low
Wang XD 2020	Low	Unclear	Low	Low	Low	Unclear	Low
Zheng XF 2021	Low	Low	Low	Unclear	Low	Unclear	Low
Szymanowicz W 2021	Low	Low	Low	Low	Low	Unclear	Low
Lakhal K 2021	Low	Unclear	Low	Low	Low	Unclear	Low
Kalisnik JM 2022	Low	Unclear	Low	Low	Low	Low	Low
Zakaria M 2022	Low	Low	Low	Low	Low	Low	Low
Abadeer M 2023	Low	Low	Low	Unclear	Low	Low	Low

### Data synthesis

**Fig 3:**
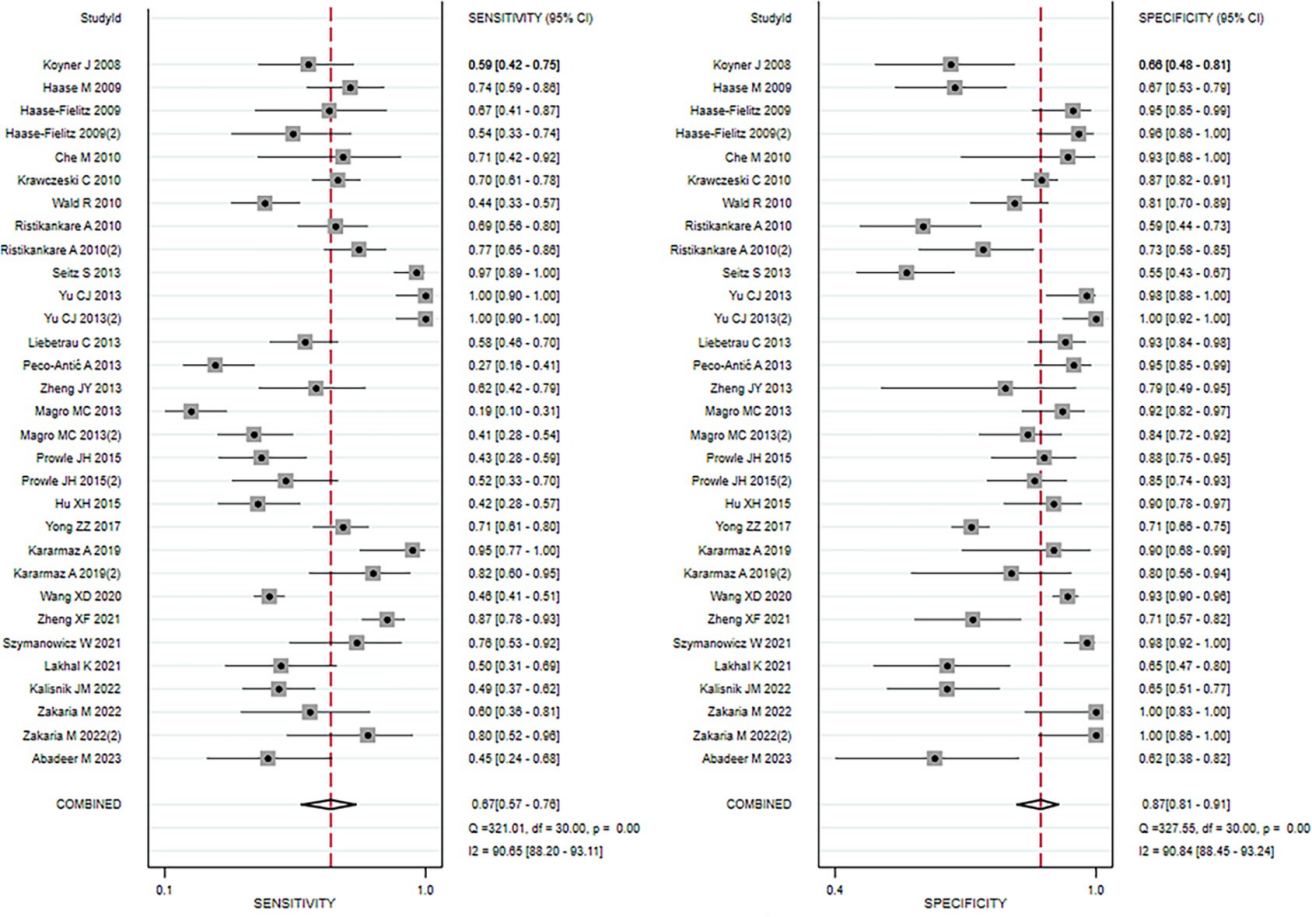
Forest plots illustrating overall sensitivity (left) and specificity (right) of serum cystatin C in diagnosing PCSAKI.

**Fig 4:**
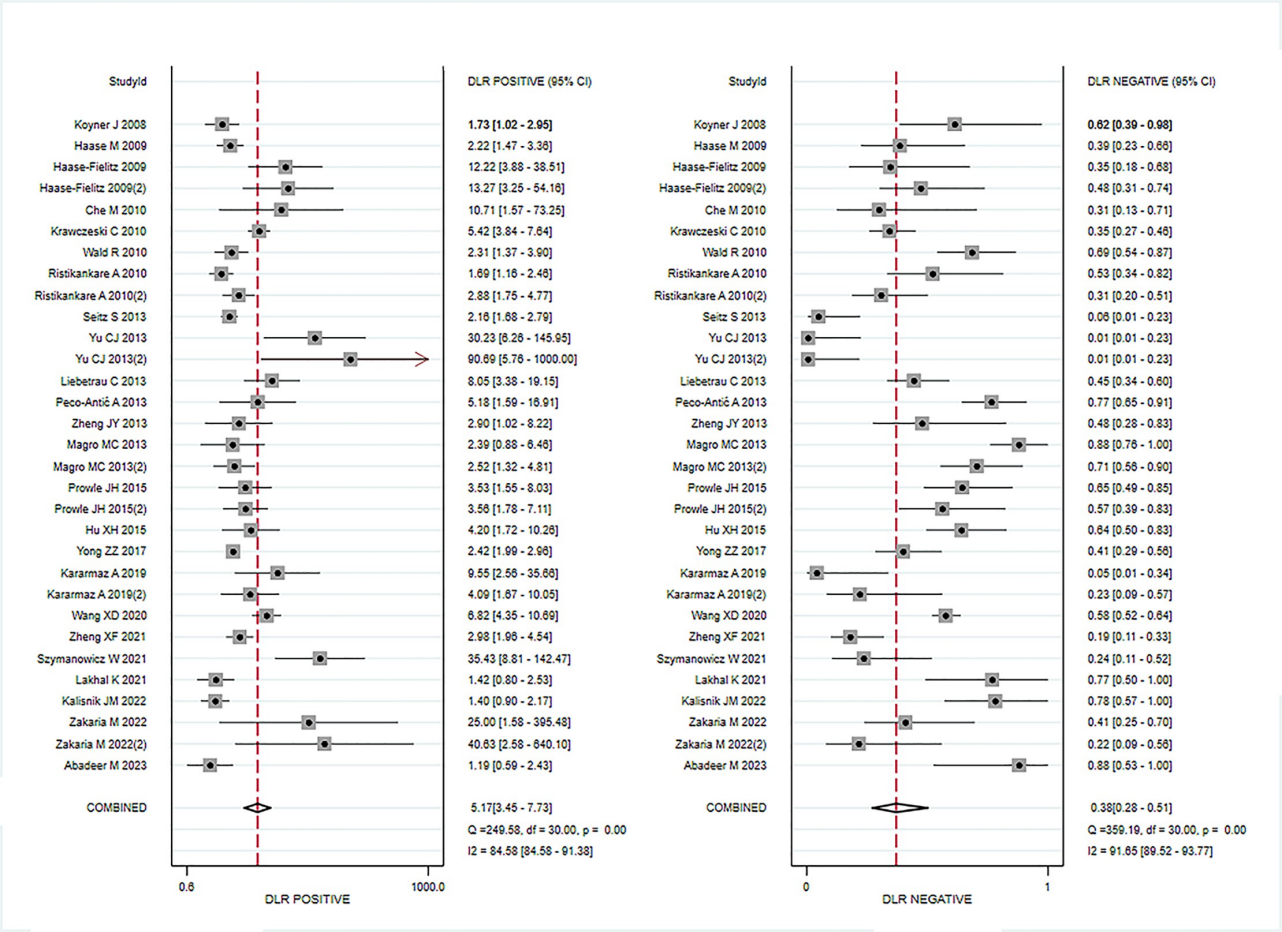
Forest plots illustrating overall +LR (left) and -LR (right) of serum cystatin C in diagnosing.

**Fig 5:**
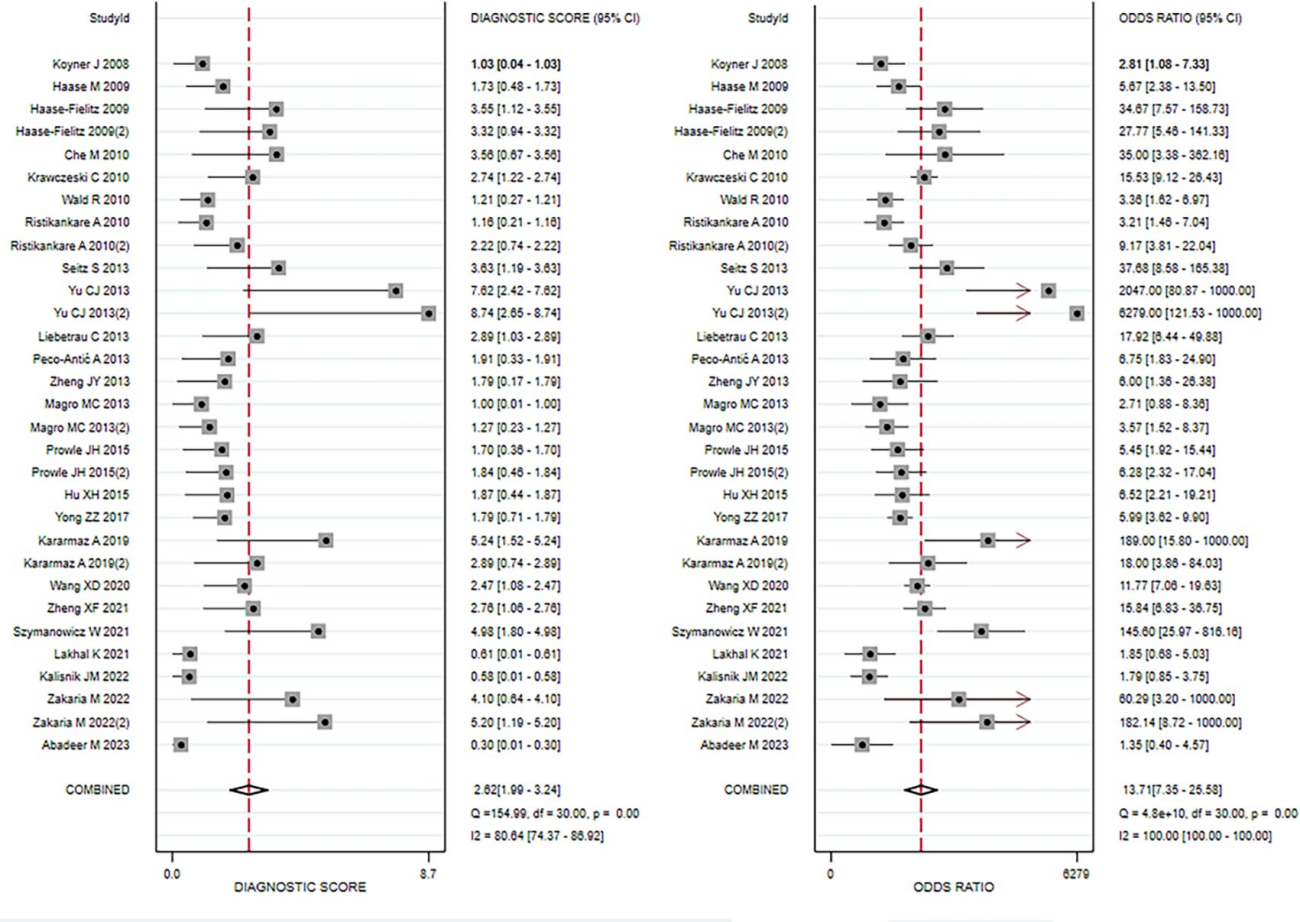
Forest plots illustrating dagnostic score (left) and odds ratio (right) of serum cystatin C in diagnosing PCSAKI.

**Fig 6:**
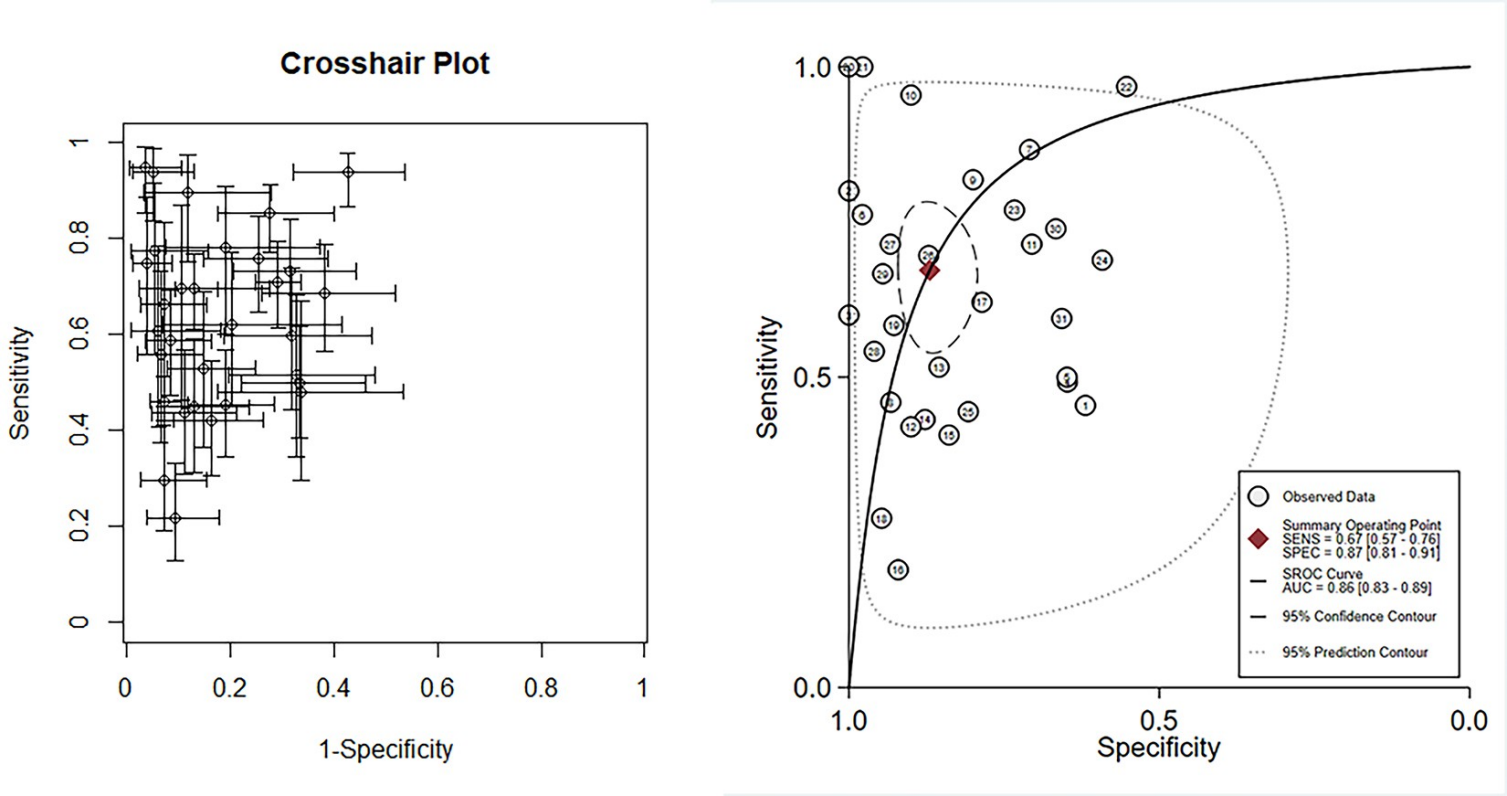
Crosshair plots of the pooled sensitivity (left) and SROC curve (right) of serum cystatin C in diagnosing PCSAKI.

**Fig 7:**
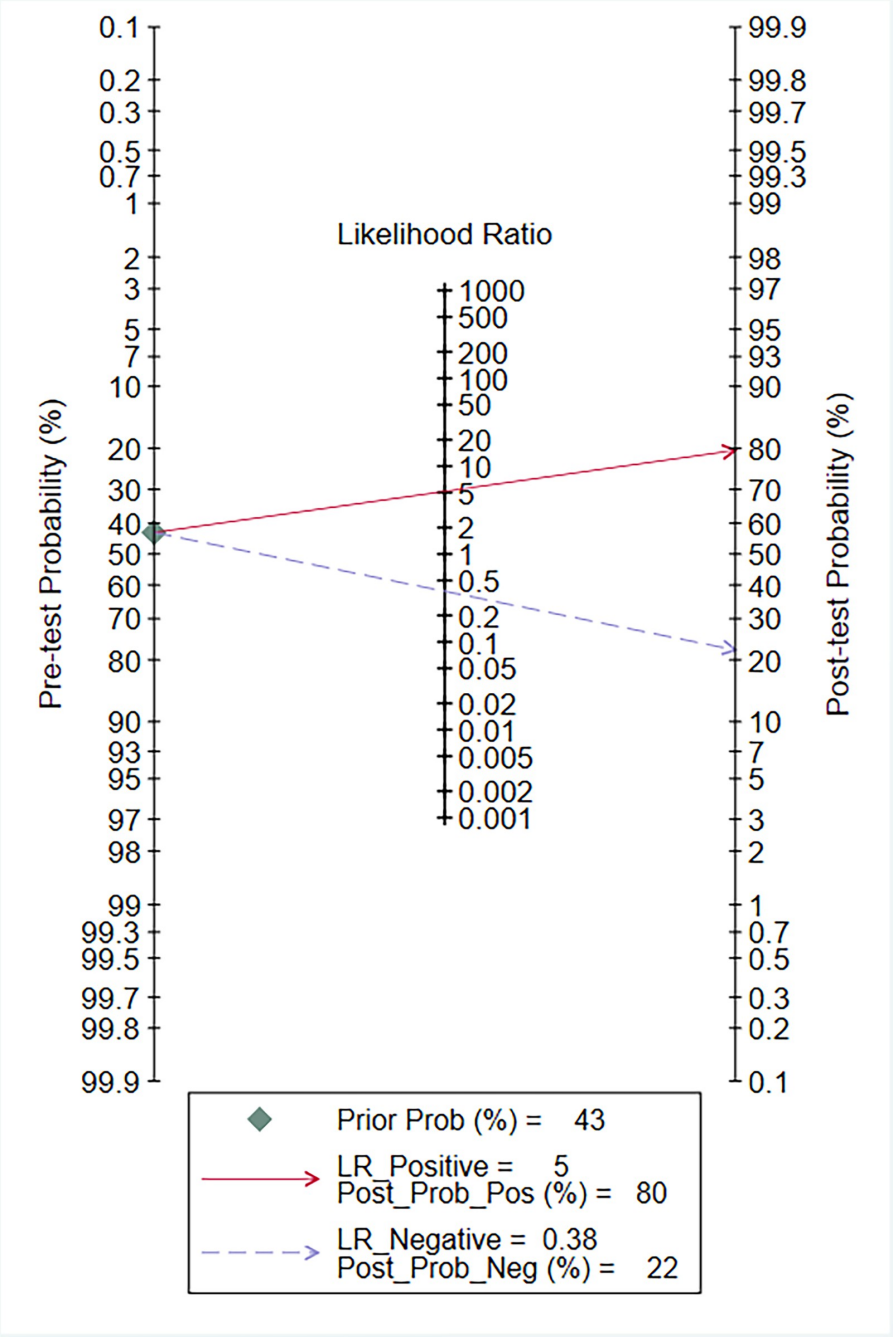
Fagan plot: A significant improvement in post-test probability compared to pre-test probability.

**Fig 8:**
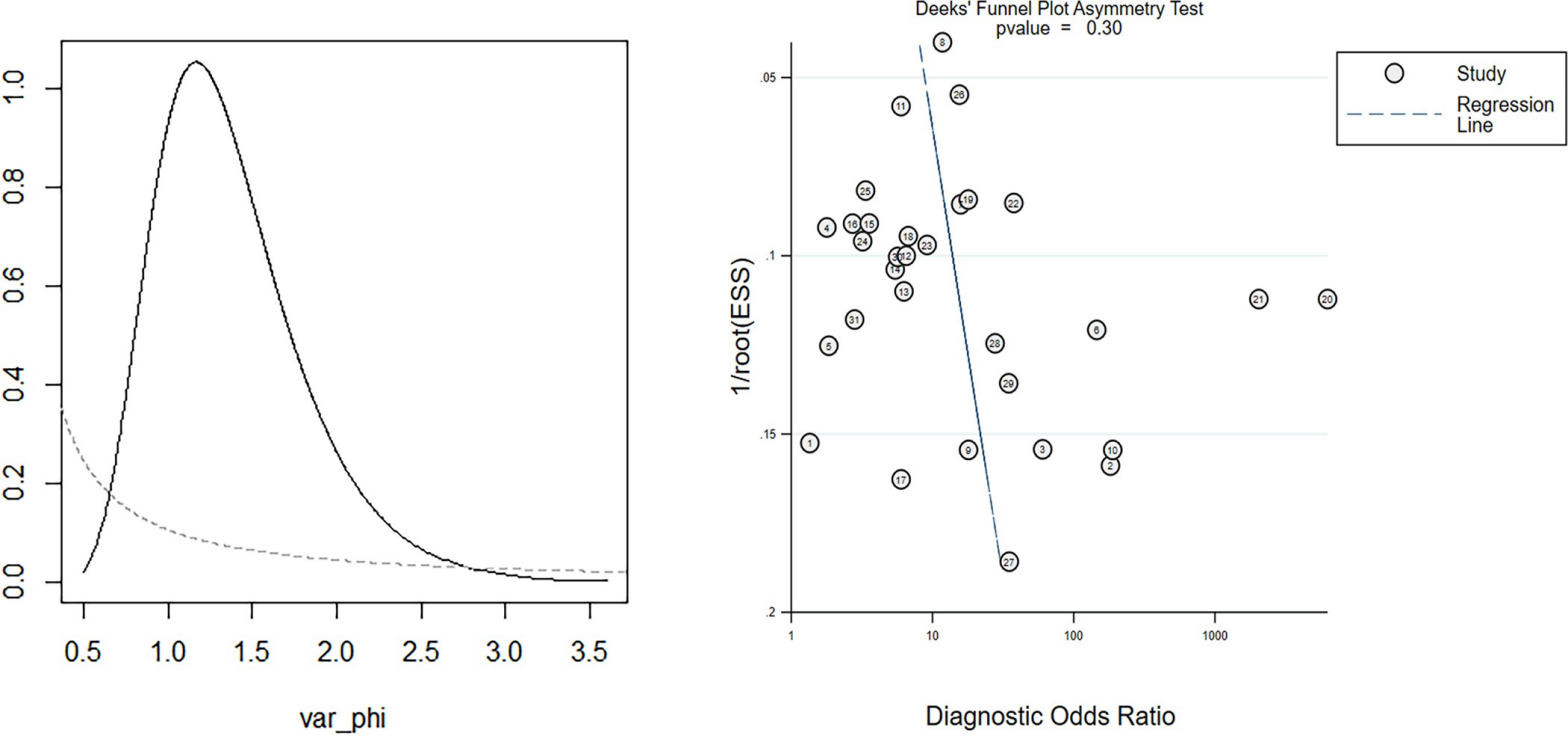
Posterior Density Distribution Plot (Left) and Funnel Plot (Right): Assessing Potential Bias in the Diagnosis of PCSAKI Using Serum CysC.

Data from 24 eligible studies, detailed in Table 3, include TP, FN, FP, and TN. The information covers Cysc measurement methods, timing, optimal cutoff values, as well as sensitivity (95% CI), specificity (95% CI), and AUC-ROC (95% CI). Serum CysC showed a sensitivity of 0.67 (95% CI, 0.57–0.76) and specificity of 0.87 (95% CI, 0.81–0.91) in diagnosing PCSAKI (Fig 3). The +LR was 5.17 (95% CI, 3.45–7.73), and the -LR was 0.38 (95% CI, 0.28–0.51) (Fig 4). The diagnostic score was 2.62 (95% CI, 1.99–3.24), with an odds ratio of 13.7 (95% CI, 7.35–25.58) (Fig 5).The cross-axis plot displays sensitivity, false positive rate values, and confidence intervals for each included study. The SROC curve indicates that serum CysC1 exhibits high efficiency in the diagnosis of PCSAKI, with an AUC of 0.86 (95% CI, 0.83–0.89) (Fig 6). The Fagan plot demonstrates a significant improvement in post-test probability compared to pre-test probability when using CysC as a biomarker (Fig 7). Furthermore, a good consistency of results is indicated by the proximity of the peak to the coordinates (1, 1) in the posterior density distribution plot and a funnel plot p-value above 0.05 indicates no bias risk (Fig 8).

**Table 3:** Serum CysC performance in PCSAKI diagnosis.

Studys	Patients				CysC		Sensitivity(%)	Specificity(%)	AUROC(95%CI)
	TP	FP	FN	TN	Time of Measurement	Cutoff Value			
Koyner J 2008	22	12	15	23	<24h after surgery	NA	65	61	0.624(0.493–0.754)
Haase M 2009	34	18	12	36	<24h after surgery	1.1mg/L	74	67	0.76(0.61–0.91)
Haase-Fielitz 2009	12	3	6	52	<24h before surgery	1.1mg/L	75	89	0.78(0.58–0.99)
Haase-Fielitz 2009(2)	13	2	11	47	<24h after surgery	1.2mg/L	86	80	0.84(0.75–0.93)
Che M 2010	10	1	4	14	10h after surgery	1.31mg/L	71	92	0.83(0.67–1)
Krawczeski C 2010	87	32	38	217	12h after surgery	1.16mg/L	73	85	0.81(0.74–0.88)
Wald R 2010	32	15	40	63	2h after surgery	NA	67	61	0.68(0.58–0.78)
Ristikankare A 2010	42	20	19	29	<24h after surgery	NA	67	61	0.71(0.61–0.81)
Ristikankare A 2010(2)	50	12	15	33	<48h after surgery	NA	79	68	0.77(0.68–0.86)
Seitz S 2013	61	34	2	42	2h after surgery	0.995mg/L	80	66	0.7401(0.606–0.875)
Yu CJ 2013	34	1	0	44	6h after surgery	NA	100	98	0.95(NA)
Yu CJ 2013(2)	34	0	0	45	48h after surgery	NA	100	100	1(NA)
Liebetrau C 2013	42	5	30	64	4h after surgery	NA	89	68	0.76(0.65–0.94)
Peco-AntićA 2013	15	3	40	54	2h after surgery	NA	81.85	57.2	0.73(NA)
Zheng JY 2013	18	3	11	11	6h after surgery	0.47mg/L	85.7	50	0.69(NA)
Magro MC 2013	11	5	47	58	<24h after surgery	NA	66	55	0.719(0.62–0.817)
Magro MC 2013(2)	24	10	35	52	<24h after surgery	NA	71	60	0.721(NA)
Prowle JHF 2015	19	6	25	43	<24h after surgery	1.24	76	63.2	0.69(0.56–0.82)
Prowle JH 2015(2)	16	9	15	53	<48h after surgery	1.57	64	77.9	0.72(0/59-0.85)
Hu XH 2015	21	5	29	45	<24h after surgery	NA	80	60	0.7(NA)
Yong ZZ 2017	65	119	26	285	4-5d after surgery	1.03mg/L	71.43	70.54	0.77(0.71–0.82)
Kararmaz A 2019	21	2	1	18	2h after surgery	0.85mg/L	93	87	0.902(0.79–1)
Kararmaz A 2019(2)	18	4	4	16	2h before surgery	0.85mg/L	78	80	0.796(NA)
Wang XD 2020	159	19	187	263	10h after surgery	1.32mg/L	88.8	58.3	0.75(NA)
Zheng XF 2021	78	16	12	39	<24h after surgery	0.975mg/L	86.5	71.4	0.804(0.726–0.881)
Szymanowicz W 2021	16	2	5	91	<24h after surgery	1.23mg/L	88	94	0.914(0.82–1)
Lakhal K 2021	14	13	14	24	<24h after surgery	NA	51	63	0.69(0.56–0.81)
Kalisnik JM 2022	32	19	33	35	<24h after surgery	NA	62	51	59(NA)
Zakaria M 2022	12	0	8	20	<24h after surgery	1.37mg/L	100	68.8	0.781(0.58–0.97)
Zakaria M 2022(2)	12	0	3	25	<48h after surgery	1.27mg/L	1	87.5	1(1–1)
Abadeer M 2023	10	8	12	13	<24h after surgery	NA	56	52	0.54(NA)

Note:NA,Not Available; TP: true positive; TN: true negative; FP: false positive; FN: false negative.

This study found significant heterogeneity in combining sensitivity and specificity. Threshold analysis with a Spearman correlation coefficient of -0.077 and a p-value of 0.680 indicated that heterogeneity was not due to threshold effects. Subgroup analyses based on various criteria were then performed, and the results are presented in Table 4.

**Table 4:** Subgroup analysis using various criteria.

studies	Sensitivity(95%Cl)	Specifcity(95%CI)	+LR (95%CI)	−LR (95%CI)	DOR(95%CI)	DS(95%CI)	AUC(95%CI)
**All studies (31)**	0.67(0.57–0.76)	0.87(0.81–0.91)	5.17(3.45–7.73)	0.38(0.28–0.51)	14(7,26)	2.62(1.99–3.24)	0.86(0.83–0.89)
**Study design**							
Prospective (29)	0.67(0.56–0.76)	0.87(0.82–0.91)	5.3(3.4–8.1)	0.38(0.27–0.52)	14(7,27)	2.64(1.97–3.3)	0.86(0.83–0.89)
Retrospective study(2)	0.71(0.62–0.80)	0.73(0.68–0.77)	2.42(1.94–2.91)	0.39(0.27–0.51)	9.87(2.07–47.09)	2.01(1.50–2.51)	NA
**Age**							
Children(7)	0.64(0.41–0.83)	0.89(0.74–0.96)	6(2.4–14.7)	0.4(0.22–0.72)	15(5–47)	2.71(1.56–3.88)	0.86(0.83–0.89)
adult(24)	0.68(0.57–0.78)	0.86(0.80–0.91)	5.0(3.2–8.0)	0.37(0.26–0.52)	14(6–29)	2.62(1.87–3.37)	0.86(0.83–0.89)
**Sex ratio**							
Men>50%(24)	0.61(0.52–0.69)	0.85(0.79–0.89)	4.0(3.1–5.2)	0.46(0.38–0.56)	9(6–12)	2.15(1.80–2.51)	0.81(0.78–0.85)
Men≤50%(7)	0.87(0.63–0.96)	0.93(0.78–0.98)	13.1(3.1–56.3)	0.14(0.04–0.49)	92(7–1252)	4.52(1.92–7.13)	0.96(0.94–0.97)
**Sample Storage**							
-80°C(16)	0.72(0.55–0.84)	0.87(0.78–0.93)	5.6(2.9–10.6)	0.32(0.18–0.56)	17(6–51)	2.88(1.79–3.93)	0.88(0.85–0.91)
<-80°C(6)	0.56(0.45–0.67)	0.88(0.78–0.94)	4.6(2.2–9.6)	0.50(0.37–0.68)	9(3–25)	2.21(1.21–3.21)	0.76(0.72–0.80)
unclear(9)	0.68(0.50–0.81)	0.86(0.74–0.93)	4.7(2.4–9.3)	0.38(0.23–0.63)	12(5–34)	2.52(1.51–3.53)	0.85(0.82–0.88)
**Diagnostic criteria**							
RIFLE criteria(10)	0.57(0.38–0.74)	0.86(0.77–0.92)	4.2(2.8–6.1)	0.50(0.34–0.74)	8(5–15)	2.12(1.53–2.71)	0.83(0.80–0.86)
AKIN criteria(9)	0.72(0.55–0.84)	0.84(0.74–0.91)	4.6(2.5–8.4)	0.34(0.20–0.57)	14(5–39)	2.62(1.59–3.65)	0.86(0.83–0.89)
KDIGO Classification(12)	0.71(0.55–0.83)	0.90(0.77–0.96)	6.9(2.6–18.3)	0.33(0.19–0.56)	21(5–90)	3.05(1.61–4.50)	0.87(0.84–0.90)
**Assay method**							
ELISA kit(4)	0.45(0.33–0.58)	0.76(0.55–0.89)	1.9(1.1–3.1)	0.73(0.61–0.86)	3(1–5)	0.94(0.29–1.58)	0.58(0.54–0.62)
Immunonephelometric assay(27)	0.70(0.60–0.79)	0.88(0.83–0.92)	6.0(3.9–9.2)	0.34(0.24–0.47)	18(9–35)	2.88(2.21–3.55)	0.8(0.85–0.91)
**Test time**							
<24h before surgery(2)	0.77(0.63–0.90)	0.93(0.87–1.00)	2.31(1.39–3.23)	0.32(0.16–0.48)	2.02(1.48–2.56)	5.90(0.40–11.41)	NA
<24h after surgery(24)	0.63(0.51–0.73)	0.87(0.81–0.91)	4.8(3.2–7.3)	0.43(0.32–0.58)	11(6–21)	2.42(1.79–3.04)	0.84(0.81–0.87)
>24h after surgery(5)	0.83(0.58–0.95)	0.94(0.59–0.99)	14.3(1.3–162.3)	0.18(0.06–0.56)	79(3–2336)	4.38(0.99–7.76)	0.94(0.92–0.96)
**AKI Diabetes(%)**							
≤25%(7)	0.57(0.44–0.69)	0.79(0.68–0.87)	2.7(1.8–4.2)	0.54(0.42–0.71)	5(3–9)	1.62(1.00–2.23)	0.74(0.70–0.77)
>25%(9)	0.65(0.57–0.72)	0.86(0.74–0.93)	4.6(2.4–8.6)	0.40(0.33–0.50)	11(5–24)	2.43(1.69–3.17)	0.76(0.72–0.81)

Note:NA,Not Available; +LR,positive likelihood ratio; -LR,negative likelihood ratio; DOR,dagnostic odds ratio;DS,dagnostic score; AUC,area under the cu

The findings reveal that gender distribution, serum storage temperature, CysC detection method, and testing time have a substantial impact on sensitivity, specificity, and AUC area. In diagnostics, accuracy is higher in the female sample group, -80°C storage, and immunofluorescence testing. Testing more than 24 hours after surgery is superior to less than 24 hours before surgery, which, in turn, is better than less than 24 hours after surgery.

## Discussion

This article reviews relevant studies and systematically evaluates the diagnostic value of serum CysC for PCSAKI. By including 24 studies with a total of 3427 patients, it was found that CysC has high specificity (0.87, 95% CI, 0.81–0.91) and moderate sensitivity (0.67, 95% CI, 0.57–0.76) in diagnosing PCSAKI during the perioperative period. The comprehensive analysis showed that the area under the SROC curve was 0.86 (95% CI, 0.83–0.89), indicating that serum CysC has a high predictive value for diagnosing PCSAKI during the perioperative period.

Early diagnosis of AKI is crucial for treatment and prognosis. Currently, PCSAKI is typically diagnosed by observing elevated serum creatinine levels or reduced urine output [39]. However, existing diagnostic standards fail to meet clinical needs, as the damage may already be irreversible by the time AKI is detected [40]. Therefore, there is an urgent need for more effective methods to diagnose AKI at an earlier stage.

To achieve the goal of early prediction of PCSAKI, we divided the detection times into three subgroups: 0–24 hours preoperatively, 0–24 hours postoperatively, and 24–72 hours postoperatively. We assessed the diagnostic performance at these different time points. The results showed that the detection at 24–72 hours postoperatively had the best performance: sensitivity of 0.83 (95% CI, 0.58–0.95) and specificity of 0.94 (95% CI, 0.59–0.99). The next best was 0–24 hours preoperatively: sensitivity of 0.77 (95% CI, 0.63–0.90) and specificity of 0.93 (95% CI, 0.87–1.00). The worst performance was 0–24 hours postoperatively: sensitivity of 0.63 (95% CI, 0.51–0.73) and specificity of 0.87 (95% CI, 0.81–0.91). Comprehensive analysis indicates that 0–24 hours postoperatively has the poorest diagnostic performance, which is not in line with the goal of early prediction. The diagnostic specificity of 0–24 hours preoperatively is similar to that of 24–72 hours postoperatively, with a narrower 95% confidence interval, indicating a more precise diagnosis, and the sensitivity is also similar. More importantly, the 0–24 hours preoperative detection allows for earlier identification of patients who may develop PCSAKI, enabling preparations for potential kidney injury. This allows medical staff to better manage potential AKI situations and save medical resources by focusing on patients who are predicted to develop PCSAKI.

To facilitate its clinical application, we analyzed the cut-off values used in the studies. The results showed that most of the studies had cut-off values concentrated around 1.2 mg/L, which could provide a reference for the establishment of future detection standards. However, the cut-off values in the included studies varied widely (ranging from 0.47 to 1.57 mg/L). Therefore, more research is needed to further standardize these values to achieve consistent diagnostic results in clinical practice.

In addition, we conducted a series of subgroup analyses to explore factors potentially influencing the CysC. We found that the sensitivity and specificity of CysC were influenced by gender, sample storage temperature, detection method, detection time, and the presence of diabetes. Firstly, our subgroup analysis revealed that CysC exhibited higher sensitivity and specificity in samples where males accounted for less than 50%. Literature reports also indicate gender differences in CysC, especially in children, which is consistent with our findings [41]. Secondly, sample storage temperature significantly affected CysC detection results. Samples stored at -70°C showed lower sensitivity and specificity compared to those stored at -80°C. This suggests that sample storage conditions should be strictly controlled in standardized detection processes. Additionally, we found that immunofluorescence detection methods have higher sensitivity and specificity relative to ELISA assay kits. Therefore, selecting appropriate detection methods is crucial for improving the diagnostic accuracy of CysC. It is noteworthy that we found a higher incidence of diabetes among PCSAKI patients, indicating that these patients are more prone to AKI, consistent with existing research findings [42, 43]. Additionally, in high-risk diabetic patients, there is a significant increase in the sensitivity and specificity of CysC detection, further supporting the close association between diabetes and PCSAKI. Therefore, we recommend considering the use of new antidiabetic medications during the perioperative period, especially those that do not harm kidney function and do not interfere with testing. This approach may have significant benefits in reducing the risk of kidney injury after cardiac surgery [44].

This study also assessed the consistency of included studies through density plot analysis, with results showing a peak close to (1, 1), indicating good consistency among the study outcomes. Funnel plot analysis revealed no publication bias, and overall quality assessment indicated high study quality. Considering potential threshold effects and the correlation between sensitivity and specificity, threshold effect analysis was conducted, which indicated no threshold effect. This suggests that the results of this study are reliable.

## Strengths and limitations

This study integrated multiple research results, increased sample size, and improved the accuracy of CysC in diagnosing PCSAKI. It found that CysC can predict PCSAKI before surgery, enabling early intervention, and proposed diagnostic cutoff values for clinical use. The study also identified factors affecting CysC’s diagnosis of PCSAKI, offering guidance for precise diagnostics and ensuring result reliability through various quality control assessments.

However, studies included in this research have shown extreme variability in the diagnostic cutoff values of CysC, necessitating further clinical studies for validation and supplementation. Moreover, blind controls in some included studies were flawed, potentially affecting the objectivity of the results. Lastly, the conclusions are only applicable to patients after cardiac surgery; further research is needed to assess applicability to other patient populations.

## Conclusion

Serum CysC demonstrates high specificity in the diagnosis of acute kidney injury following cardiac surgery, with high sensitivity when detected within 0–24 hours preoperatively, enabling early prediction. However, further research is needed to determine its practical application in clinical settings.

### Registration number

This study has been registered with PROSPERO and the registration number is CRD42024521067.

## Supporting information

S1 Table Detailed search strategy terms. (DOCX)

S2 Table All studies identified in the literature search. (DOCX)

S3 Table Data extracted from the primary research sources for the systematic review and meta-analysis. (DOCX)

S4 Table Missing data handling. (DOCX)

S5 Table Screening summary and details of 2811 records for analysis. (DOCX)

S6 Table PRISMA 2020 checklist. (DOCX)
