# Supplementary material for: The value of serum cystatin c in predicting acute kidney injury after cardiac surgery: A systematic review and meta-analysis
Source: PLoS One. 2024 Nov 20;19(11):e0310049. doi: 10.1371/journal.pone.0310049 (PMC11578473; doi:10.1371/journal.pone.0310049)
Supplement: S1 Table — (DOCX) [file pone.0310049.s001.docx]

**S1 Table**: Detailed search strategy terms

| PubMed | 1. “Serum cystatin C” OR “Cystatin C” OR “Cystatin-C” OR “Cys-C” OR OR “CysC” OR “CST3” 2. “Acute Kidney Injury” OR “AKI” OR “Acute renal failure” OR “Renal injury” OR “Kidney damage” OR “Kidney dysfunction” OR “Nephropathy” OR “Renal impairment” 3. “Post-cardiac surgery” OR “Cardiac surgery recovery” OR “Cardiac surgical procedures” OR “Coronary artery bypass graft surgery” OR “Cardiac valve surgery” OR “Cardiac rehabilitation” OR “Heart surgery outcomes” 4. “Biomarkers” OR “Diagnostic biomarkers” OR “Diagnostic markers” OR “Biomarkers for diagnosis” OR “Diagnostic tests” OR “Disease biomarkers” OR “Prognostic biomarkers” OR “Predictive biomarkers” OR “Molecular diagnostics” OR “Clinical biomarkers” 5. “1” AND “2” AND “3” AND “4” |
| --- | --- |
| Embase |  |
| Cochrane |  |
| WanFang | 1、“血清胱抑素C” OR “胱抑素C” OR “Cysc”  2、“心脏手术后” OR “心脏术后” OR “心脏术后康复” OR“心脏术后恢复” OR “冠状动脉搭桥术后” OR “心脏瓣膜术后” OR “心脏康复” OR “心脏术后预后”  3、“诊断标志物”OR “标志物” OR “生物标志物” OR “血液标志物” OR “预测标志物” OR “特异性标志物”  4、“1” AND “2” AND “3” |
| CNKI |  |
| VIP |  |
