# Supplementary material for: The value of serum cystatin c in predicting acute kidney injury after cardiac surgery: A systematic review and meta-analysis
Source: PLoS One. 2024 Nov 20;19(11):e0310049. doi: 10.1371/journal.pone.0310049 (PMC11578473; doi:10.1371/journal.pone.0310049)
Supplement: S2 Table — (DOCX) [file pone.0310049.s002.docx]

**S2 Table.** All studies identified in the literature search.

|  | Studys | Reference |
| --- | --- | --- |
| 1 | Koyner J 2008 | Koyner, J.L., et al., Urinary cystatin C as an early biomarker of acute kidney injury following adult cardiothoracic surgery. Kidney Int, 2008. 74(8): p. 1059-69. |
| 2 | Haase M 2009 | Haase, M., et al., Novel biomarkers early predict the severity of acute kidney injury after cardiac surgery in adults. Ann Thorac Surg, 2009. 88(1): p. 124-30. |
| 3 | Haase-Fielitz 2009 | Haase-Fielitz, A., et al., Novel and conventional serum biomarkers predicting acute kidney injury in adult cardiac surgery--a prospective cohort study. Crit Care Med, 2009. 37(2): p. 553-60. |
| 4 | Che M 2010 | Che, M., et al., Clinical usefulness of novel biomarkers for the detection of acute kidney injury following elective cardiac surgery. Nephron Clin Pract, 2010. 115(1): p. c66-72. |
| 5 | Krawczeski C 2010 | Krawczeski, C.D., et al., Serum cystatin C is an early predictive biomarker of acute kidney injury after pediatric cardiopulmonary bypass. Clin J Am Soc Nephrol, 2010. 5(9): p. 1552-7. |
| 6 | Wald R 2010 | Wald, R., et al., Plasma cystatin C and acute kidney injury after cardiopulmonary bypass. Clin J Am Soc Nephrol, 2010. 5(8): p. 1373-9. |
| 7 | Ristikankare A 2010 | Ristikankare, A., et al., Serum cystatin C in elderly cardiac surgery patients. Ann Thorac Surg, 2010. 89(3): p. 689-94. |
| 8 | Seitz S 2013 | Seitz, S., et al., Cystatin C and neutrophil gelatinase-associated lipocalin: biomarkers for acute kidney injury after congenital heart surgery. Swiss Med Wkly, 2013. 143: p. w13744. |
| 9 | Yu CJ 2013 | Yu CJ. Changes in Blood Cys-C and Urine Kim-1 Concentrations After Cardiopulmonary Bypass Surgery and Their Diagnostic Value for AKI[D]. Southern Medical University.2013. |
| 10 | Liebetrau C 2013 | Liebetrau, C., et al., Neutrophil gelatinase-associated lipocalin (NGAL) for the early detection of cardiac surgery associated acute kidney injury. Scand J Clin Lab Invest, 2013. 73(5): p. 392-9. |
| 11 | Peco-Antić A 2013 | Peco-Antić, A., et al., Biomarkers of acute kidney injury in pediatric cardiac surgery. Clin Biochem, 2013. 46(13-14): p. 1244-51. |
| 12 | Zheng JY 2013 | Zheng, J.Y., et al., Is serum cystatin C an early predictor for acute kidney injury following cardiopulmonary bypass surgery in infants and young children? Kaohsiung J Med Sci, 2013. 29(9): p. 494-9. |
| 13 | Magro MC 2013 | Magro, M.C. and F. Vattimo Mde, Impact of cystatin C and RIFLE on renal function assessment after cardiac surgery. Biol Res Nurs, 2013. 15(4): p. 451-8. |
| 14 | Prowle JR 2015 | Prowle, J.R., et al., Combination of biomarkers for diagnosis of acute kidney injury after cardiopulmonary bypass. Ren Fail, 2015. 37(3): p. 408-16. |
| 15 | Hu XH 2015 | Hu XH, Niu Zl, Wang J, et al., Diagnostic value of kidney injury molecular 1, cystatin C and urinary creatinine in acute kidney injury induced by cardiopulmonary bypass of congenital heart disease. Hainan Medicine, 2015. 26(13): p. 1934-1937 |
| 16 | Yong ZZ 2017 | Yong ZZ, Application of serum cystatin C in acute kidney injury [D]. Nanjing Medical University, 2017. |
| 17 | Kararmaz A 2019 | Kararmaz, A., et al., Evaluation of acute kidney injury with oxidative stress biomarkers and Renal Resistive Index after cardiac surgery. Acta Chir Belg, 2021. 121(3): p. 189-197. |
| 18 | Wang XD 2020 | Wang, X., et al., Early serum cystatin C-enhanced risk prediction for acute kidney injury post cardiac surgery: a prospective, observational, cohort study. Biomarkers, 2020. 25(1): p. 20-26. |
| 19 | Zheng XF 2021 | Zheng XF, Study and Validation of Cystatin C in Predicting Postoperative Acute Kidney Injury and Outcomes in Surgical Patients.Shantou University[D]. 2021. |
| 20 | Szymanowicz W 2021 | Szymanowicz, W., et al., Brain and Muscle Oxygen Saturation Combined with Kidney Injury Biomarkers Predict Cardiac Surgery Related Acute Kidney Injury. Diagnostics (Basel), 2021. 11(9). |
| 21 | Lakhal K 2021 | Lakhal, K., et al., Early recognition of cardiac surgery-associated acute kidney injury: lack of added value of TIMP2 IGFBP7 over short-term changes in creatinine (an observational pilot study). BMC Anesthesiol, 2021. 21(1): p. 244. |
| 22 | Kalisnik JM 2022 | Kalisnik, J.M., et al., Enhanced Detection of Cardiac Surgery-Associated Acute Kidney Injury by a Composite Biomarker Panel in Patients with Normal Preoperative Kidney Function. J Cardiovasc Dev Dis, 2022. 9(7). |
| 23 | Zakaria M 2022 | Zakaria, M., et al., Role of serum cystatin C in the prediction of acute kidney injury following pediatric cardiac surgeries: A single center experience. Medicine (Baltimore), 2022. 101(49): p. e31938. |
| 24 | Abadeer M 2023 | Abadeer, M., et al., *Using Serum Cystatin C to Predict Acute Kidney Injury Following Infant Cardiac Surgery.* Pediatr Cardiol, 2023. **44**(4): p. 855-866. |
