# Supplementary material for: The value of serum cystatin c in predicting acute kidney injury after cardiac surgery: A systematic review and meta-analysis
Source: PLoS One. 2024 Nov 20;19(11):e0310049. doi: 10.1371/journal.pone.0310049 (PMC11578473; doi:10.1371/journal.pone.0310049)
Supplement: S3 Table — (DOCX) [file pone.0310049.s003.docx]

**S3 Table.** Data extracted from the primary research sources for the systematic review and meta-analysis

| Data extractors and extraction date | Studys | TP | FP | FN | TN |
| --- | --- | --- | --- | --- | --- |
| 1&2,2024,3,2 | Koyner J 2008 | 22 | 12 | 15 | 23 |
| 1&2,2024,3,2 | Haase M 2009 | 34 | 18 | 12 | 36 |
| 1&2,2024,3,2 | Haase-Fielitz 2009 | 12 | 3 | 6 | 52 |
| 1&2,2024,3,2 | Haase-Fielitz 2009(2) | 13 | 2 | 11 | 47 |
| 1&2,2024,3,2 | Che M 2010 | 10 | 1 | 4 | 14 |
| 1&2,2024,3,2 | Krawczeski C 2010 | 87 | 32 | 38 | 217 |
| 1&2,2024,3,3 | Wald R 2010 | 32 | 15 | 40 | 63 |
| 1&2,2024,3,3 | Ristikankare A 2010 | 42 | 20 | 19 | 29 |
| 1&2,2024,3,3 | Ristikankare A 2010(2) | 50 | 12 | 15 | 33 |
| 1&2,2024,3,3 | Seitz S 2013 | 61 | 34 | 2 | 42 |
| 1&2,2024,3,3 | Yu CJ 2013 | 34 | 1 | 0 | 44 |
| 1&2,2024,3,3 | Yu CJ 2013(2) | 34 | 0 | 0 | 45 |
| 1&2,2024,3,4 | Liebetrau C 2013 | 42 | 5 | 30 | 64 |
| 1&2,2024,3,4 | Peco-Antić A 2013 | 15 | 3 | 40 | 54 |
| 1&2,2024,3,4 | Zheng JY 2013 | 18 | 3 | 11 | 11 |
| 1&2,2024,3,4 | Magro MC 2013 | 11 | 5 | 47 | 58 |
| 1&2,2024,3,4 | Magro MC 2013(2) | 24 | 10 | 35 | 52 |
| 1&2,2024,3,5 | Prowle JH 2015 | 19 | 6 | 25 | 43 |
| 1&2,2024,3,5 | Prowle JH 2015(2) | 16 | 9 | 15 | 53 |
| 1&2,2024,3,5 | Hu XH 2015 | 21 | 5 | 29 | 45 |
| 1&2,2024,3,5 | Yong ZZ 2017 | 65 | 119 | 26 | 285 |
| 1&2,2024,3,5 | Kararmaz A 2019 | 21 | 2 | 1 | 18 |
| 1&2,2024,3,5 | Kararmaz A 2019(2) | 18 | 4 | 4 | 16 |
| 1&2,2024,3,5 | Wang XD 2020 | 159 | 19 | 187 | 263 |
| 1&2,2024,3,6 | Zheng XF 2021 | 78 | 16 | 12 | 39 |
| 1&2,2024,3,6 | Szymanowicz W 2021 | 16 | 2 | 5 | 91 |
| 1&2,2024,3,6 | Lakhal K 2021 | 14 | 13 | 14 | 24 |
| 1&2,2024,3,6 | Kalisnik JM 2022 | 32 | 19 | 33 | 35 |
| 1&2,2024,3,6 | Zakaria M 2022 | 12 | 0 | 8 | 20 |
| 1&2,2024,3,6 | Zakaria M 2022(2) | 12 | 0 | 3 | 25 |
| 1&2,2024,3,6 | Abadeer M 2023 | 10 | 8 | 12 | 13 |

Note：1：Pq Peng; 2:Xc F; TP: true positive; TN: true negative; FP: false positive; FN: false negative.
