# Supplementary material for: The value of serum cystatin c in predicting acute kidney injury after cardiac surgery: A systematic review and meta-analysis
Source: PLoS One. 2024 Nov 20;19(11):e0310049. doi: 10.1371/journal.pone.0310049 (PMC11578473; doi:10.1371/journal.pone.0310049)
Supplement: S4 Table — (DOCX) [file pone.0310049.s004.docx]

**S4 Table.** Missing data handling

This study contains no missing values. If not directly specified in the literature, conversions can be performed using the following formula.

| Sensitivity=TP/(TP+FN) | Specificity=TN/(TN+FP) | N=TP+FP+FN+TN |
| --- | --- | --- |

|  |
| --- |

Note：TP: true positive; TN: true negative; FP: false positive; FN: false negative; N：total number of participants
