# Supplementary material for: The value of serum cystatin c in predicting acute kidney injury after cardiac surgery: A systematic review and meta-analysis
Source: PLoS One. 2024 Nov 20;19(11):e0310049. doi: 10.1371/journal.pone.0310049 (PMC11578473; doi:10.1371/journal.pone.0310049)
Supplement: S5 Table — (DOCX) [file pone.0310049.s005.docx]

**S5 Table.** Screening Summary and Details of 2811 Records for Analysis

| Preliminary screening | Filter further | Number of articles | Page |  |
| --- | --- | --- | --- | --- |
| Meta |  | 91 | 1 | Exclusion |
| Report |  | 10 | 6 |  |
| Non-randomized controlled trial |  | 126 | 7 |  |
| Non-cardiac postoperative injuries |  | 1005 | 14 |  |
| Basic experiments, proteomics, other small molecules, drug trials, correlation research analysis, modeling |  | 528 | 64 |  |
| Kidney-related but not acute kidney injury |  | 136 | 93 |  |
| The diagnostic marker is not serum cystatin C, or is not a diagnostic test or cannot react alone or extract serum cystatin c |  | 413 | 100 |  |
| Review |  | 110 | 122 |  |
| There is cystatin c but it does not match the theme |  | 248 | 127 |  |
| Duplicate article |  | 101 |  |  |
| Remaining | Include | 24 | 143 | Include |
|  | Combined biomarkers | 6 | 145 | Exclusion |
|  | Not acute kidney injury | 2 | 145 |  |
|  | Incomplete Data | 7 | 145 |  |
|  | Not retrieved | 1 | 146 |  |
|  | Urinary diagnostic biomarkers | 3 | 145 |  |

Meta(91)

1 "Abdulle, A., et al., Metabolic dysfunction in Emirati subjects in Abu Dhabi: Relationship to levels of soluble RAGEs. Journal of Clinical & Translational Endocrinology, 2019. 16: p. 100192."

2 "Adiels, M., et al., Niacin action in the atherogenic mixed dyslipidemia of metabolic syndrome: Insights from metabolic biomarker profiling and network analysis. J Clin Lipidol, 2018. 12(3): p. 810-821.e1."

3 "Agarwal, I., et al., Associations between metabolic dysregulation and circulating biomarkers of fibrosis: the Cardiovascular Health Study. Metabolism, 2015. 64(10): p. 1316-1323."

4 "Ashton, E., et al., Impact of sarcopenia indexes on survival and severe immune acute toxicity in metastatic non-small cell lung cancer patients treated with PD-1 immune checkpoint inhibitors. Clinical Nutrition, 2023. 42(6): p. 944-953."

5 "Azimi, A., et al., Investigating proteome changes between primary and metastatic cutaneous squamous cell carcinoma using SWATH mass spectrometry. Journal of Dermatological Science, 2020. 99(2): p. 119-127."

6 "Bai, Y., et al., Use of ultra high performance liquid chromatography with high resolution mass spectrometry to analyze urinary metabolome alterations following acute kidney injury in post-cardiac surgery patients. Journal of Mass Spectrometry and Advances in the Clinical Lab, 2022. 24: p. 31-40."

7 "Bansal, N., et al., Vitamin D Metabolic Ratio and Risks of Death and CKD Progression. Kidney International Reports, 2019. 4(11): p. 1598-1607."

8 "Bayrasheva, V.K., et al., Short-term renal and metabolic effects of low dose vildagliptin treatment added-on insulin therapy in non-proteinuric patients with type 2 diabetes: open-label randomized prospective study. Arch Endocrinol Metab, 2020. 64(4): p. 418-426."

9 "Benito, S., et al., Plasma biomarker discovery for early chronic kidney disease diagnosis based on chemometric approaches using LC-QTOF targeted metabolomics data. Journal of Pharmaceutical and Biomedical Analysis, 2018. 149: p. 46-56."

10 "Brosnan, J.T., et al., Lifestyle, metabolite, and genetic determinants of formate concentrations in a cross-sectional study in young, healthy adults. The American Journal of Clinical Nutrition, 2018. 107(3): p. 345-354."

11 "Bustamante, A., et al., Prognostic value of blood interleukin-6 in the prediction of functional outcome after stroke: A systematic review and meta-analysis. Journal of Neuroimmunology, 2014. 274(1): p. 215-224."

12 "Cabral, M., et al., Renal impairment assessment on adults living nearby a landfill: Early kidney dysfunction biomarkers linked to the environmental exposure to heavy metals. Toxicology Reports, 2021. 8: p. 386-394."

13 "Ca?adas-Garre, M., et al., Proteomic and metabolomic approaches in the search for biomarkers in chronic kidney disease. Journal of Proteomics, 2019. 193: p. 93-122."

14 "Cao, T., et al., Serum uric acid to creatinine ratio and metabolic syndrome in middle-aged and elderly population: Based on the 2015 CHARLS. Nutrition, Metabolism and Cardiovascular Diseases, 2023. 33(7): p. 1339-1348."

15 "Castro-Mejía, M.A., et al., Evaluation of renal function in precarious workers exposed to heavy metals in vulnerable scenarios in the metropolitan area of San Luis Potosí, México. Environmental Toxicology and Pharmacology, 2024. 106: p. 104350."

16 "Chen, C.-T., et al., Optimal measuring timing of cystatin C for early detection of contrast-induced acute kidney injury: A systematic review and meta-analysis. Toxicology Letters, 2020. 318: p. 65-73."

17 "Chen, F., et al., Coenzyme Q10 combined with trimetazidine in the prevention of contrast-induced nephropathy in patients with coronary heart disease complicated with renal dysfunction undergoing elective cardiac catheterization: a randomized control study and in vivo study. Eur J Med Res, 2018. 23(1): p. 23."

18 "Chen, H., et al., Metabolomics insights into activated redox signaling and lipid metabolism dysfunction in chronic kidney disease progression. Redox Biology, 2016. 10: p. 168-178."

19 "Chicas, R.C., et al., The impact of heat exposures on biomarkers of AKI and plasma metabolome among agricultural and non-agricultural workers. Environment International, 2023. 180: p. 108206."

20 "Danielli, M., et al., Blood biomarkers to predict the onset of pre-eclampsia: A systematic review and meta-analysis. Heliyon, 2022. 8(11): p. e11226."

21 "Domingueti, C.P., et al., Association between Von Willebrand factor, disintegrin and metalloproteinase with thrombospondin type 1 motif member 13, d-Dimer and cystatin C levels with retinopathy in type 1 diabetes mellitus. Clinica Chimica Acta, 2016. 459: p. 1-4."

22 "Dowling, P., et al., Analysis of the saliva proteome from patients with head and neck squamous cell carcinoma reveals differences in abundance levels of proteins associated with tumour progression and metastasis. Journal of Proteomics, 2008. 71(2): p. 168-175."

23 "Eerik, K., et al., Effects of RIPC on the Metabolome in Patients Undergoing Vascular Surgery: A Randomized Controlled Trial. Biomolecules, 2022. 12(9)."

24 "Feng, Y.-L., et al., Activated NF-κB/Nrf2 and Wnt/β-catenin pathways are associated with lipid metabolism in CKD patients with microalbuminuria and macroalbuminuria. Biochimica et Biophysica Acta (BBA) - Molecular Basis of Disease, 2019. 1865(9): p. 2317-2332."

25 "Fino, N.F., et al., Evaluation of novel candidate filtration markers from a global metabolomic discovery for glomerular filtration rate estimation. Kidney International, 2024. 105(3): p. 582-592."

26 "Fonseca, R.I.D., et al., Untargeted plasma 1H NMR-based metabolomic profiling in different stages of chronic kidney disease. Journal of Pharmaceutical and Biomedical Analysis, 2023. 229: p. 115339."

27 "Fu, C.P., et al., Weight loss reduces serum monocyte chemoattractant protein-1 concentrations in association with improvements in renal injury in obese men with metabolic syndrome. Clin Chem Lab Med, 2015. 53(4): p. 623-9."

28 "Ganda, A., et al., Mild renal dysfunction and metabolites tied to low HDL cholesterol are associated with monocytosis and atherosclerosis. Circulation, 2013. 127(9): p. 988-96."

29 "Goraya, N., et al., A comparison of treating metabolic acidosis in CKD stage 4 hypertensive kidney disease with fruits and vegetables or sodium bicarbonate. Clin J Am Soc Nephrol, 2013. 8(3): p. 371-81."

30 "Goraya, N., et al., Treatment of metabolic acidosis in patients with stage 3 chronic kidney disease with fruits and vegetables or oral bicarbonate reduces urine angiotensinogen and preserves glomerular filtration rate. Kidney Int, 2014. 86(5): p. 1031-8."

31 "Goraya, N., et al., Urine citrate excretion as a marker of acid retention?in patients with chronic kidney disease without overt metabolic acidosis. Kidney International, 2019. 95(5): p. 1190-1196."

32 "Gorski, M., et al., Genetic loci and prioritization of genes for kidney function decline derived from a meta-analysis of 62 longitudinal genome-wide association studies. Kidney International, 2022. 102(3): p. 624-639."

33 "Guo, M., et al., Targeted metabolomic analysis of plasma fatty acids in acute myocardial infarction in young adults. Nutrition, Metabolism and Cardiovascular Diseases, 2021. 31(11): p. 3131-3141."

34 "Hanssen, R., et al., Chromosomal deletions on 16p11.2 encompassing SH2B1 are associated with accelerated metabolic disease. Cell Reports Medicine, 2023. 4(8): p. 101155."

35 "Harada, M., et al., Importance of cystatin C and uric acid levels in the association of cardiometabolic risk factors in Japanese junior high school students. Journal of Cardiology, 2017. 69(1): p. 222-227."

36 "Heringlake, M., et al., The metabolic and renal effects of adrenaline and milrinone in patients with myocardial dysfunction after coronary artery bypass grafting. Crit Care, 2007. 11(2): p. R51."

37 "Ho, J., et al., Urinary, Plasma, and Serum Biomarkers’ Utility for Predicting Acute Kidney Injury Associated With Cardiac Surgery in Adults: A Meta-analysis. American Journal of Kidney Diseases, 2015. 66(6): p. 993-1005."

38 "Hou, W., et al., Development of Multimarker Diagnostic Models from Metabolomics Analysis for Gestational Diabetes Mellitus (GDM)*. Molecular & Cellular Proteomics, 2018. 17(3): p. 431-441."

39 "Hsiao, Y.-C., et al., An immuno-MALDI mass spectrometry assay for the oral cancer biomarker, matrix metalloproteinase-1, in dried saliva spot samples. Analytica Chimica Acta, 2020. 1100: p. 118-130."

40 "Hu, Z., et al., Effects of long-term vitamin D supplementation on metabolic profile in middle-aged and elderly patients with type 2 diabetes. The Journal of Steroid Biochemistry and Molecular Biology, 2023. 225: p. 106198."

41 "Kakuda, H., et al., Effects of change in high-density lipoprotein cholesterol by statin switching on glucose metabolism and renal function in hypercholesterolemia. J Clin Lipidol, 2015. 9(5): p. 709-15."

42 "Kasiske, B.L., et al., Abnormalities in biomarkers of mineral and bone metabolism in kidney donors. Kidney International, 2016. 90(4): p. 861-868."

43 "Khaliq, W., et al., Lipid metabolic signatures deviate in sepsis survivors compared to non-survivors. Computational and Structural Biotechnology Journal, 2020. 18: p. 3678-3691."

44 "Kiess, A., et al., Influence of growth and metabolic markers on hs-troponin T and NT-proBNP levels in healthy children. Endocrine Connections, 2023. 12(10)."

45 "Kirk, B., et al., Leucine-enriched whey protein supplementation, resistance-based exercise, and cardiometabolic health in older adults: a randomized controlled trial. J Cachexia Sarcopenia Muscle, 2021. 12(6): p. 2022-2033."

46 "Koh, S.S., et al., Differential gene expression profiling of primary cutaneous melanoma and sentinel lymph node metastases. Modern Pathology, 2012. 25(6): p. 828-837."

47 "Lee, J.G., et al., Multiple biomarkers and their relative contributions to identifying metabolic syndrome. Clinica Chimica Acta, 2009. 408(1): p. 50-55."

48 "Lee, S.K., et al., Metabolomics Study for Identification of Potential Biomarkers of Long-term Survival in Kidney Transplantation Recipients. Transplantation Proceedings, 2017. 49(5): p. 1005-1011."

49 "Li, Y., et al., Ultrasensitive electrochemiluminescent immunosensing based on trimetallic Au–Pd–Pt/MoS2 nanosheet as coreaction accelerator and self-enhanced ABEI-centric complex. Analytica Chimica Acta, 2020. 1125: p. 86-93."

50 "Li, Y.-m., et al., Evaluation of vitamin D storage in patients with chronic kidney disease: Detection of serum vitamin D metabolites using high performance liquid chromatography-tandem mass spectrometry. The Journal of Steroid Biochemistry and Molecular Biology, 2021. 210: p. 105860."

51 "Lim, Y.J., et al., Metabolomics for the identification of early biomarkers of nephrotoxicity in a mouse model of cisplatin-induced acute kidney injury. Biomedicine & Pharmacotherapy, 2023. 163: p. 114787."

52 "Liu, C., et al., An enzyme-free electrochemical immunosensor based on quaternary metallic/nonmetallic PdPtBP alloy mesoporous nanoparticles/MXene and conductive CuCl2 nanowires for ultrasensitive assay of kidney injury molecule-1. Sensors and Actuators B: Chemical, 2021. 334: p. 129585."

53 "Liu, H., et al., QEEG indices are associated with inflammatory and metabolic risk factors in Parkinson's disease dementia: An observational study. eClinicalMedicine, 2022. 52: p. 101615."

54 "Liu, W., et al., Trimetazidine Prevention of Contrast-Induced Nephropathy in Coronary Angiography. Am J Med Sci, 2015. 350(5): p. 398-402."

55 "Matsushita, K., et al., Measures of chronic kidney disease and risk of incident peripheral artery disease: a collaborative meta-analysis of individual participant data. The Lancet Diabetes & Endocrinology, 2017. 5(9): p. 718-728."

56 "Medeiros, T., et al., Metabolic syndrome components and estimated glomerular filtration rate based on creatinine and/or cystatin C in young adults: A gender issue? Diabetes & Metabolic Syndrome: Clinical Research & Reviews, 2017. 11: p. S351-S357."

57 "Mindikoglu, A.L., et al., Unique metabolomic signature associated with hepatorenal dysfunction and mortality in cirrhosis. Translational Research, 2018. 195: p. 25-47."

58 "Monin, M., et al., Switching to a NRTI-free 2 drug regimen (2DR) -a sub-analysis of the 48 weeks DUALIS study on metabolic and renal changes. HIV Res Clin Pract, 2021. 23(1): p. 15-21."

59 "Neunhoeffer, F., et al., Non-invasive measurement of renal perfusion and oxygen metabolism to predict postoperative acute kidney injury in neonates and infants after cardiopulmonary bypass surgery. British Journal of Anaesthesia, 2016. 117(5): p. 623-634."

60 "Nibali, L., et al., Analysis of gingival crevicular fluid biomarkers in patients with metabolic syndrome. Journal of Dentistry, 2022. 118: p. 104065."

61 "Nicholson, G., et al., Osteocalcin and the hormonal, inflammatory and metabolic response to major orthopaedic surgery. Anaesthesia, 2002. 57(4): p. 319-25."

62 "Omran, A., et al., Potential role of blood microRNAs as non-invasive biomarkers for early detection of asymptomatic coronary atherosclerosis in obese children with metabolic syndrome. Medical Hypotheses, 2012. 79(6): p. 889-893."

63 "Prozialeck, W.C., P.C. Lamar, and J.R. Edwards, Effects of sub-chronic Cd exposure on levels of copper, selenium, zinc, iron and other essential metals in rat renal cortex. Toxicology Reports, 2016. 3: p. 740-746."

64 "Roos, J.F., et al., Diagnostic accuracy of cystatin C compared to serum creatinine for the estimation of renal dysfunction in adults and children—A meta-analysis. Clinical Biochemistry, 2007. 40(5): p. 383-391."

65 "Santos, G.B.d., et al., Excretory/secretory products in the Echinococcus granulosus metacestode: is the intermediate host complacent with infection caused by the larval form of the parasite? International Journal for Parasitology, 2016. 46(13): p. 843-856."

66 "Schaub, J.A., et al., Amino-Terminal Pro-B-Type Natriuretic Peptide for?Diagnosis and Prognosis in Patients With Renal?Dysfunction: A Systematic Review and Meta-Analysis. JACC: Heart Failure, 2015. 3(12): p. 977-989."

67 "Silva, R.E., et al., Predictive metabolomic signatures of end-stage renal disease: A multivariate analysis of population-based data. Biochimie, 2018. 152: p. 14-30."

68 "Singh, C., et al., ChREBP is activated by reductive stress and mediates GCKR-associated metabolic traits. Cell Metabolism, 2024. 36(1): p. 144-158.e7."

69 "Spoto, B., et al., Inflammation biomarkers and inflammatory genes expression in metabolically healthy obese patients. Nutrition, Metabolism and Cardiovascular Diseases, 2023. 33(3): p. 584-591."

70 "Trivedi, T.S., et al., Identification of hub genes associated with human cystic fibrosis: A Meta-analysis approach. Human Gene, 2023. 35: p. 201139."

71 "van Ballegooijen, A.J., et al., Vitamin D metabolites and bone mineral density: The multi-ethnic study of atherosclerosis. Bone, 2015. 78: p. 186-193."

72 "Vigil, L., et al., Cystatin C is associated with the metabolic syndrome and other cardiovascular risk factors in a hypertensive population. Journal of the American Society of Hypertension, 2009. 3(3): p. 201-209."

73 "Wang, C.-l., et al., Urinary metabolomics analysis to reveal metabolic mechanism of guanxinning injection on heart failure with renal dysfunction. Journal of Pharmaceutical and Biomedical Analysis, 2022. 209: p. 114516."

74 "Wang, Y. and Y. Guo, RenalGuard system and conventional hydration for preventing contrast-associated acute kidney injury in patients undergoing cardiac interventional procedures: A systematic review and meta-analysis. International Journal of Cardiology, 2021. 333: p. 83-89."

75 "Wang, Y., et al., Decreased CSTA expression promotes lymphatic metastasis and predicts poor survival in oral squamous cell carcinoma. Archives of Oral Biology, 2021. 126: p. 105116."

76 "Weaver, V.M., et al., Impact of urine concentration adjustment method on associations between urine metals and estimated glomerular filtration rates (eGFR) in adolescents. Environmental Research, 2014. 132: p. 226-232."

77 "Wikstrom, J., et al., Diastolic dysfunction and impaired cardiac output reserve in dysmetabolic nonhuman primate with proteinuria. Journal of Diabetes and its Complications, 2021. 35(4): p. 107881."

78 "Woon Kim, D., et al., Virtual diagnosis of diabetic nephropathy using metabolomics in place of kidney biopsy: The DIAMOND study. Diabetes Research and Clinical Practice, 2023. 205: p. 110986."

79 "Xiao, X., et al., Metaproteomics Characterizes the Human Gingival Crevicular Fluid Microbiome Function in Periodontitis. Journal of Proteome Research, 2023. 22(7): p. 2411-2420."

80 "Xu, B., et al., Untargeted and spatial-resolved metabolomics characterize serum and tissue-specific metabolic reprogramming in acute kidney injury. Heliyon, 2023. 9(11): p. e21171."

81 "Yang, C., et al., Kidney function, bone-mineral metabolism markers, and future risk of peripheral artery disease. Atherosclerosis, 2017. 267: p. 167-174."

82 "Yen, C.-H., et al., Relationship Between Metabolic Scores, Systemic Inflammation, Renal Function, and High-risk Peripheral Arterial Disease. International Journal of Gerontology, 2013. 7(3): p. 142-146."

83 "Yu, M., et al., Serum ProGRP as a novel biomarker of bone metastasis in prostate cancer. Clinica Chimica Acta, 2020. 510: p. 437-441."

84 "Yu, Z., et al., Plasma metabolic disturbances during pregnancy and postpartum in women with depression. iScience, 2022. 25(12): p. 105666."

85 "Yuan, T.-H., et al., Relationship between renal function and metal exposure of residents living near the No. 6 Naphtha Cracking Complex: A cross-sectional study. Journal of the Formosan Medical Association, 2021. 120(10): p. 1845-1854."

86 "Zhang, R., et al., [Effect of trimetazidine on renal function in patients with shock]. Zhonghua Wei Zhong Bing Ji Jiu Yi Xue, 2014. 26(4): p. 219-22."

87 "Zhang, X., et al., Preventive effect of trimetazidine on contrast-induced nephropathy undergoing percutaneous coronary intervention in elderly moderate and high risk diabetics stratified by mehran score. Perfusion, 2021. 36(5): p. 491-500."

88 "Zhang, Z., et al., Cystatin C in Prediction of Acute Kidney Injury: A Systemic Review and Meta-analysis. American Journal of Kidney Diseases, 2011. 58(3): p. 356-365."

89 "Zhou, M., et al., Changes in glomerular filtration rate and metabolomic differences in severely ill coronavirus disease survivors 3?months after discharge. Biochimica et Biophysica Acta (BBA) - Molecular Basis of Disease, 2022. 1868(1): p. 166289."

90 "吴意, 张.A.肖.A., 胱抑素C诊断早期急性肾损伤价值的Meta分析. 检验医学与临床, 2018. 15(23)."

91 "张毕明, 肖帅, and 吴意, 胱抑素C诊断早期急性肾损伤价值的Meta分析. 检验医学与临床, 2018. 15(23): p. 3485-3487,3491."

Report(10)

1 "Delgado, C., et al., Association of Frailty based on self-reported physical function with directly measured kidney function and mortality. BMC Nephrol, 2015. 16: p. 203."

2 "Delgado, C., et al., Reassessing the Inclusion of Race in Diagnosing Kidney Diseases: An Interim Report From the NKF-ASN Task Force. American Journal of Kidney Diseases, 2021. 78(1): p. 103-115."

3 "Duan, W., et al., Thallium exposure at low concentration leads to early damage on multiple organs in children: A case study followed-up for four years. Environmental Pollution, 2020. 258: p. 113319."

4 "Ergatoudes, C., et al., Natriuretic and Inflammatory Biomarkers as Risk Predictors of Heart Failure in Middle-Aged Men From the General Population: A 21-Year Follow-Up. Journal of Cardiac Failure, 2018. 24(9): p. 594-600."

5 "Kalogeropoulos, A.P., V.V. Georgiopoulou, and J. Butler, Clinical Adoption of Prognostic Biomarkers: The Case for Heart Failure. Progress in Cardiovascular Diseases, 2012. 55(1): p. 3-13."

6 "Kunutsor, S.K., et al., Self-reported alcohol consumption, carbohydrate deficient transferrin and risk of cardiovascular disease: The PREVEND prospective cohort study. Clinica Chimica Acta, 2021. 520: p. 1-7."

7 "Kwinta-Rybicka, J., et al., [Mycophenolate mofetil in treatment of childhood nephrotic syndrome--preliminary report]. Przegl Lek, 2006. 63 Suppl 3: p. 44-8."

8 "Morales Corado, J.A., C.U. Lee, and G.M. Enns, Carnitine-Acylcarnitine Translocase Deficiency, in GeneReviews(?), M.P. Adam, et al., Editors. 1993, University of Washington, Seattle"

9 "Park, M., et al., Soluble endothelial cell selective adhesion molecule and cardiovascular outcomes in patients with stable coronary disease: A report from the Heart and Soul Study. Atherosclerosis, 2015. 243(2): p. 546-552."

10 "Rubio-Gracia, J., et al., Intraabdominal pressure and worsening renal function during decompensations of heart failure. A preliminary report from the PIA study. Revista Clínica Espa?ola (English Edition), 2019. 219(5): p. 229-235."

Non-randomized controlled trial(126)

1 "Alcazar, O., et al., Longitudinal proteomics analysis in the immediate microenvironment of islet allografts during progression of rejection. Journal of Proteomics, 2020. 223: p. 103826."

2 "Alehagen, U., et al., Selenium and Coenzyme Q10 Supplementation Improves Renal Function in Elderly Deficient in Selenium: Observational Results and Results from a Subgroup Analysis of a Prospective Randomised Double-Blind Placebo-Controlled Trial. Nutrients, 2020. 12(12)."

3 "Askenazi, D.J., et al., Prevalence of acute kidney injury (AKI) in extremely low gestational age neonates (ELGAN). Pediatr Nephrol, 2020. 35(9): p. 1737-1748."

4 "Askenazi, D.J., et al., The Impact of Erythropoietin on Short- and Long-Term Kidney-Related Outcomes in Neonates of Extremely Low Gestational Age. Results of a Multicenter, Double-Blind, Placebo-Controlled Randomized Clinical Trial. J Pediatr, 2021. 232: p. 65-72.e7."

5 "Badimón, J.J., et al., Nuevas herramientas en la estratificación del riesgo cardiovascular. Revista Espa?ola de Cardiología Suplementos, 2011. 11: p. 21-28."

6 "Baldimtsi, E., P.A. Whiss, and J. Wahlberg, Systemic biomarkers of microvascular alterations in type 1 diabetes associated neuropathy and nephropathy - A prospective long-term follow-up study. Journal of Diabetes and its Complications, 2023. 37(12): p. 108635."

7 "Bao, X., et al., Growth differentiation factor-15 is a biomarker for all-cause mortality but less evident for cardiovascular outcomes: A prospective study. American Heart Journal, 2021. 234: p. 81-89."

8 "Beck, L.H., et al., KDOQI US Commentary on the 2021 KDIGO Clinical Practice Guideline for the Management of Glomerular Diseases. American Journal of Kidney Diseases, 2023. 82(2): p. 121-175."

9 "Bhandari, S., et al., Cardiological Society of India Practice Guidelines for Angiography in Patients with Renal Dysfunction. Indian Heart Journal, 2012. 64: p. S18-S43."

10 "Brismar, K. and S.E. Nilsson, Interrelations and associations of serum levels of steroids and pituitary hormones with markers of insulin resistance, inflammatory activity, and renal function in men and women aged >70 years in an 8-year longitudinal study of opposite-sex twins. Gender Medicine, 2009. 6: p. 123-136."

11 "Bush, N., et al., Renal doppler changes in patients with acute pancreatitis: A prospective study. Pancreatology, 2020. 20(7): p. 1275-1280."

12 "Chen, Y., et al., Assessment of cisplatin-induced kidney injury using an integrated rodent platform. Toxicology and Applied Pharmacology, 2013. 268(3): p. 352-361."

13 "Chow, S.L., et al., Modulation of novel cardiorenal and inflammatory biomarkers by intravenous nitroglycerin and nesiritide in acute decompensated heart failure: an exploratory study. Circ Heart Fail, 2011. 4(4): p. 450-5."

14 "Chuasuwan, A. and J.A. Kellum, Cardio-Renal Syndrome Type 3: Epidemiology, Pathophysiology, and Treatment. Seminars in Nephrology, 2012. 32(1): p. 31-39."

15 "Cohen, E.P., et al., Onco-Nephrology: Core Curriculum 2015. American Journal of Kidney Diseases, 2015. 66(5): p. 869-883."

16 "Cooperrider, J., B. Bluett, and S.E. Jones, Methods and utility of quantitative brainstem measurements in progressive supranuclear palsy versus Parkinson's disease in a routine clinical setting. Clinical Parkinsonism & Related Disorders, 2020. 3: p. 100033."

17 "Cruz, I., et al., SalivaPRINT Toolkit – Protein profile evaluation and phenotype stratification. Journal of Proteomics, 2018. 171: p. 81-86."

18 "Davis, J., et al., Ability of different assay platforms to measure renal biomarker concentrations during ischaemia-reperfusion acute kidney injury in dogs. Research in Veterinary Science, 2021. 135: p. 547-554."

19 "Demissei, B.G., et al., A multimarker multi-time point-based risk stratification strategy in acute heart failure: results from the RELAX-AHF trial. Eur J Heart Fail, 2017. 19(8): p. 1001-1010."

20 "Deng, M., et al., Association between sarcopenia and multimorbidity among middle-aged and older adults in China: Findings from the China Health and Retirement Longitudinal Study. Experimental Gerontology, 2024. 185: p. 112348."

21 "Dhont, E., et al., Reliability of glomerular filtration rate estimating formulas compared to iohexol plasma clearance in critically ill children. Eur J Pediatr, 2022. 181(11): p. 3851-3866."

22 "Dodig-Crnkovi?, T., et al., Facets of individual-specific health signatures determined from longitudinal plasma proteome profiling. EBioMedicine, 2020. 57: p. 102854."

23 "Enger, T.B., et al., A Preoperative Multimarker Approach to Evaluate Acute Kidney Injury After Cardiac Surgery. Journal of Cardiothoracic and Vascular Anesthesia, 2017. 31(3): p. 837-846."

24 "Eurlings, L.W., et al., Multimarker Strategy for Short-Term Risk Assessment in Patients With Dyspnea in the Emergency Department: The MARKED (Multi mARKer Emergency Dyspnea)-Risk Score. Journal of the American College of Cardiology, 2012. 60(17): p. 1668-1677."

25 "Fang, M., et al., Prediction of acute kidney injury after total aortic arch replacement with serum cystatin C and urine N-acetyl-β-d-glucosaminidase: A prospective observational study. Clinica Chimica Acta, 2023. 539: p. 105-113."

26 "Filler, G., et al., A cross-sectional study measuring vanadium and chromium levels in paediatric patients with CKD. BMJ Open, 2017. 7(5): p. e014821."

27 "Focà, E., et al., Prospective evaluation of bone markers, parathormone and 1,25-(OH)? vitamin D in HIV-positive patients after the initiation of tenofovir/emtricitabine with atazanavir/ritonavir or efavirenz. BMC Infect Dis, 2012. 12: p. 38."

28 "Folsom, A.R., et al., Longitudinal increases in blood biomarkers of inflammation or cardiovascular disease and the incidence of venous thromboembolism. Journal of Thrombosis and Haemostasis, 2018. 16(10): p. 1964-1972."

29 "Fu, N., et al., The efficacy of probucol combined with hydration in preventing contrast-induced nephropathy in patients with coronary heart disease undergoing percutaneous coronary intervention: a multicenter, prospective, randomized controlled study. Int Urol Nephrol, 2018. 50(1): p. 105-112."

30 "Gaither, C., et al., Multiple Reaction Monitoring-Mass Spectrometry Enables Robust Quantitation of Plasma Proteins Regardless of Whole Blood Processing Delays That May Occur in the Clinic. Molecular & Cellular Proteomics, 2022. 21(5): p. 100212."

31 "Gawenda, M. and J. Brunkwall, Renal response to open and endovascular repair of abdominal aortic aneurysm: a prospective study. Ann Vasc Surg, 2008. 22(1): p. 1-4."

32 "Gil Rosa, B., et al., Multiplexed immunosensors for point-of-care diagnostic applications. Biosensors and Bioelectronics, 2022. 203: p. 114050."

33 "Gold, L., et al., Advances in human proteomics at high scale with the SOMAscan proteomics platform. New Biotechnology, 2012. 29(5): p. 543-549."

34 "Gómez Dammeier, B.H., et al., Anuria during pneumoperitoneum in infants and children: a prospective study. J Pediatr Surg, 2005. 40(9): p. 1454-8."

35 "Granger, D.A., et al., Focus on Methodology: Salivary bioscience and research on adolescence: An integrated perspective. Journal of Adolescence, 2012. 35(4): p. 1081-1095."

36 "Graziani, F., et al., Effects of non-surgical periodontal therapy on the glomerular filtration rate of the kidney: an exploratory trial. J Clin Periodontol, 2010. 37(7): p. 638-43."

37 "Haase, M., et al., The identification of three novel biomarkers of major adverse kidney events. Biomark Med, 2014. 8(10): p. 1207-17."

38 "Hammami, R., et al., Impact of atorvastatin reload on the prevention of contrast-induced nephropathy in patients on chronic statin therapy: A prospective randomized trial. PLoS One, 2023. 18(5): p. e0270000."

39 "Herlyn, P.K.E., et al., Fibroblast Growth Factor-23, Sclerostin, and Bone Microarchitecture in Patients With Osteoporotic Fractures of the Proximal Femur: A Cross-sectional Study. Journal of Clinical Densitometry, 2016. 19(2): p. 192-201."

40 "Hicken, M.T., et al., Neighborhood Social Context and Kidney Function Over Time: The Multi-Ethnic Study of Atherosclerosis. American Journal of Kidney Diseases, 2019. 73(5): p. 585-595."

41 "Holmstr?m, A., et al., An integrated multiple marker modality is superior to NT-proBNP alone in prognostic prediction in all-cause mortality in a prospective cohort of elderly heart failure patients. European Geriatric Medicine, 2013. 4(6): p. 365-371."

42 "Hoogeveen, E.K., et al., Effect of omega-3 fatty acids on kidney function after myocardial infarction: the Alpha Omega Trial. Clin J Am Soc Nephrol, 2014. 9(10): p. 1676-83."

43 "Hu, J., et al., Gender-based relationship between serum creatinine and carotid plaque stability: A cross-sectional study in China. Clinical Neurology and Neurosurgery, 2023. 233: p. 107966."

44 "Husain-Syed, F., et al., Advances in laboratory detection of acute kidney injury. Practical Laboratory Medicine, 2022. 31: p. e00283."

45 "Ikizler, T.A., et al., A prospective cohort study of acute kidney injury and kidney outcomes, cardiovascular?events, and death. Kidney International, 2021. 99(2): p. 456-465."

46 "Imamura, K., et al., Safety and tolerability of bosutinib in patients with amyotrophic lateral sclerosis (iDReAM study): A multicentre, open-label, dose-escalation phase 1 trial. eClinicalMedicine, 2022. 53: p. 101707."

47 "Innes, H., et al., Performance of routine risk scores for predicting cirrhosis-related morbidity in the community. Journal of Hepatology, 2022. 77(2): p. 365-376."

48 "Iyngkaran, P., et al., Cardio-Renal Syndrome: New Perspective in Diagnostics. Seminars in Nephrology, 2012. 32(1): p. 3-17."

49 "Jia, F., et al., Association of renal function and depressive symptoms: Evidence from the China health and retirement longitudinal study. Journal of Psychosomatic Research, 2020. 137: p. 110224."

50 "Julier, K., et al., Preconditioning by sevoflurane decreases biochemical markers for myocardial and renal dysfunction in coronary artery bypass graft surgery: a double-blinded, placebo-controlled, multicenter study. Anesthesiology, 2003. 98(6): p. 1315-27."

51 "Kanagasabai, T., et al., Cross-sectional study of household solid fuel use and renal function in older adults in China. Environmental Research, 2023. 219: p. 115117."

52 "Karimzadeh, I., et al., A double-blinded, placebo-controlled, multicenter clinical trial of N-acetylcysteine for preventing amphotericin B-induced nephrotoxicity. Expert Opin Drug Metab Toxicol, 2015. 11(9): p. 1345-55."

53 "Kasiske, B.L., et al., A Prospective Controlled Study of Kidney Donors: Baseline and 6-Month Follow-up. American Journal of Kidney Diseases, 2013. 62(3): p. 577-586."

54 "Katz, D.H., et al., Multiomic Profiling in Black and White Populations Reveals Novel Candidate Pathways in Left Ventricular Hypertrophy and Incident Heart Failure Specific to Black Adults. Circ Genom Precis Med, 2021. 14(3): p. e003191."

55 "Kedia, K., et al., Application of multiplexed ion mobility spectrometry towards the identification of host protein signatures of treatment effect in pulmonary tuberculosis. Tuberculosis, 2018. 112: p. 52-61."

56 "Kiernan, M.S., et al., Determinants of Diuretic Responsiveness and Associated Outcomes During Acute Heart Failure Hospitalization: An Analysis From the NHLBI Heart Failure Network Clinical Trials. J Card Fail, 2018. 24(7): p. 428-438."

57 "Kim, B.J., et al., Effect of N-acetylcysteine on cystatin C-based renal function after elective coronary angiography (ENABLE Study): a prospective, randomized trial. Int J Cardiol, 2010. 138(3): p. 239-45."

58 "Kitago, M., et al., Cross-Sectional and Longitudinal Associations of Creatinine-to-Cystatin C Ratio with Sarcopenia Parameters in Older Adults. The Journal of nutrition, health and aging, 2023. 27(11): p. 946-952."

59 "Koz?owski, T., et al., The impact of laparoscopic adrenalectomy on renal function. Results of a prospective randomised clinical trial. Endokrynol Pol, 2019. 70(5): p. 409-416."

60 "Kunutsor, S.K., et al., Plasma neutrophil gelatinase-associated lipocalin and risk of cardiovascular disease: Findings from the PREVEND prospective cohort study. Clinica Chimica Acta, 2018. 486: p. 66-75."

61 "Lang, H., et al., Serum Fstl1, a novel biomarker screened based on protein array technology, predict acute kidney injury and major renal adverse events after cardiac surgery: A prospective cohort study. Clinica Chimica Acta, 2023. 539: p. 79-86."

62 "Laugsand, L.E., et al., Fetuin-A and risk of coronary heart disease: A Mendelian randomization analysis and a pooled analysis of AHSG genetic variants in 7 prospective studies. Atherosclerosis, 2015. 243(1): p. 44-52."

63 "Lee, B., et al., Distinct Serum Immune Profiles Define the Spectrum of Acute and Chronic Pancreatitis From the Multicenter Prospective Evaluation of Chronic Pancreatitis for Epidemiologic and Translational Studies (PROCEED) Study. Gastroenterology, 2023. 165(1): p. 173-186."

64 "Linefsky, J.P., et al., Serum phosphate is associated with aortic valve calcification in the Multi-ethnic Study of Atherosclerosis (MESA). Atherosclerosis, 2014. 233(2): p. 331-337."

65 "López-Gálvez, N., et al., Longitudinal assessment of kidney function in migrant farm workers. Environmental Research, 2021. 202: p. 111686."

66 "Ma, Y., et al., ExPRSweb: An online repository with polygenic risk scores for common health-related exposures. The American Journal of Human Genetics, 2022. 109(10): p. 1742-1760."

67 "Maguire, D., et al., Post-traumatic stress disorder: A biopsychosocial case-control study investigating peripheral blood protein biomarkers. Biomarkers in Neuropsychiatry, 2021. 5: p. 100042."

68 "Mammen, C., et al., Long-term Risk of CKD in Children Surviving Episodes of Acute Kidney Injury in the Intensive Care Unit: A Prospective Cohort Study. American Journal of Kidney Diseases, 2012. 59(4): p. 523-530."

69 "Masson, I., et al., GFR Estimation Using Standardized Cystatin C in Kidney Transplant Recipients. American Journal of Kidney Diseases, 2013. 61(2): p. 279-284."

70 "Masson, I., et al., KDIGO Guidelines and Kidney Transplantation: Is the Cystatin-C Based Recommendation Relevant? American Journal of Transplantation, 2015. 15(8): p. 2211-2214."

71 "Matsushita, K., et al., Clinical Risk Implications of the CKD Epidemiology Collaboration (CKD-EPI) Equation Compared With the Modification of Diet in Renal Disease (MDRD) Study Equation for Estimated GFR. American Journal of Kidney Diseases, 2012. 60(2): p. 241-249."

72 "Meesters, K., et al., Results of a Multicenter Population Pharmacokinetic Study of Ciprofloxacin in Children with Complicated Urinary Tract Infection. Antimicrob Agents Chemother, 2018. 62(9)."

73 "Mengi, A., et al., Effects of Therapeutic Ultrasound Applied to the Lumbar Region on Renal Function: A Randomized Controlled Prospective Trial. J Ultrasound Med, 2020. 39(7): p. 1327-1333."

74 "Micha?lsson, K., et al., Plasma vitamin D and mortality in older men: a community-based prospective cohort study123. The American Journal of Clinical Nutrition, 2010. 92(4): p. 841-848."

75 "Milks, M.W., et al., Usefulness of Integrating Heart Failure Risk Factors Into Impairment of Global Longitudinal Strain to Predict Anthracycline-Related Cardiac Dysfunction. The American Journal of Cardiology, 2018. 121(7): p. 867-873."

76 "Mitaka, C., et al., Effects of low-dose atrial natriuretic peptide infusion on cardiac surgery–associated acute kidney injury: A multicenter randomized controlled trial. Journal of Critical Care, 2017. 38: p. 253-258."

77 "Mohammadi-Shemirani, P., et al., ACLY and CKD: A Mendelian Randomization Analysis. Kidney International Reports, 2022. 7(7): p. 1673-1681."

78 "Nechaeva, N.L., et al., Simultaneous express immunoassay of multiple cardiac biomarkers with an automatic platform in human plasma. Talanta, 2021. 224: p. 121860."

79 "Nickolas, T.L., et al., Diagnostic and Prognostic Stratification in the Emergency Department Using Urinary Biomarkers of Nephron Damage: A Multicenter Prospective Cohort Study. Journal of the American College of Cardiology, 2012. 59(3): p. 246-255."

80 "Nielsen, E.I., et al., Developmental pharmacokinetics of gentamicin in preterm and term neonates: population modelling of a prospective study. Clin Pharmacokinet, 2009. 48(4): p. 253-63."

81 "Nielsen, M.B., et al., Remote ischaemic conditioning and early changes in plasma creatinine as markers of one year kidney graft function-A follow-up of the CONTEXT study. PLoS One, 2019. 14(12): p. e0226882."

82 "Oh, J., et al., Effect of high-dose statin loading on biomarkers related to inflammation and renal injury in patients hospitalized with acute heart failure. Randomized, controlled, open-label, prospective pilot study. Circ J, 2014. 78(10): p. 2447-54."

83 "Osthoff, M., et al., Impact of mannose-binding lectin deficiency on radiocontrast-induced renal dysfunction: a post-hoc analysis of a multicenter randomized controlled trial. BMC Nephrol, 2012. 13: p. 99."

84 "Patterson, C.C., et al., Which biomarkers are predictive specifically for cardiovascular or for non-cardiovascular mortality in men? Evidence from the Caerphilly Prospective Study (CaPS). International Journal of Cardiology, 2015. 201: p. 113-118."

85 "Peng, S., et al., Short-term exposure to fine particulate matter and its constituents may affect renal function via oxidative stress: A longitudinal panel study. Chemosphere, 2022. 293: p. 133570."

86 "Perna, L., et al., Risk of Late-Onset Depression and Cognitive Decline: Results From Inflammatory Proteome Analyses in a Prospective Population-Based Cohort Study. The American Journal of Geriatric Psychiatry, 2022. 30(6): p. 689-700."

87 "Petermann-Rocha, F., et al., Biomarkers Profile of People With Sarcopenia: A Cross-sectional Analysis From UK Biobank. Journal of the American Medical Directors Association, 2020. 21(12): p. 2017.e1-2017.e9."

88 "Petrera, A., et al., Multiplatform Approach for Plasma Proteomics: Complementarity of Olink Proximity Extension Assay Technology to Mass Spectrometry-Based Protein Profiling. Journal of Proteome Research, 2021. 20(1): p. 751-762."

89 "Plácido, R., et al., Prognostic stratification in pulmonary hypertension: A multi-biomarker approach. Revista Portuguesa de Cardiologia (English Edition), 2017. 36(2): p. 111-125."

90 "Polak, J.F., M. Szklo, and D.H. O’Leary, Associations of Coronary Heart Disease with Common Carotid Artery Near and Far Wall Intima-Media Thickness: The Multi-Ethnic Study of Atherosclerosis. Journal of the American Society of Echocardiography, 2015. 28(9): p. 1114-1121."

91 "Prasad, N. and A. Krishna, Acute kidney injury: definition controversies and epidemiology. Clinical Queries: Nephrology, 2012. 1(1): p. 1-5."

92 "Risberg, A., et al., Water balance during parturition and early puerperium: A prospective open trial. Clin Biochem, 2015. 48(13-14): p. 837-42."

93 "Roos, M., et al., Serum Fetuin-A, Cardiovascular Risk Factors, and Six-Year Follow-up Outcome in Patients With Coronary Heart Disease. The American Journal of Cardiology, 2010. 105(12): p. 1666-1672."

94 "Rosa, L.K., et al., Oral health, organic and inorganic saliva composition of men with Schizophrenia: Case-control study. Journal of Trace Elements in Medicine and Biology, 2021. 66: p. 126743."

95 "Salminen, M., et al., Biomarkers of kidney function and prediction of death from cardiovascular and other causes in the elderly: A 9-year follow-up study. European Journal of Internal Medicine, 2016. 33: p. 98-101."

96 "Schepke, M., et al., Hemodynamic effects of the angiotensin II receptor antagonist irbesartan in patients with cirrhosis and portal hypertension. Gastroenterology, 2001. 121(2): p. 389-95."

97 "?entürk, G.?., et al., The Prognostic Value of Cystatin C Compared with Trauma Scores in?Multiple Blunt Trauma: A Prospective Cohort Study. The Journal of Emergency Medicine, 2013. 44(6): p. 1070-1076."

98 "Shah, L.N., et al., Cystatin C and Creatinine Concentrations Are Uninformative Biomarkers of Sarcopenia: A Cross-Sectional NHANES Study. Journal of Renal Nutrition, 2023. 33(4): p. 538-545."

99 "Sharan, K., et al., Neutrophil Gelatinase–associated Lipocalin Predicts Short-term Outcomes in Decompensated Cirrhosis With Acute Kidney Injury. Journal of Clinical and Experimental Hepatology, 2024. 14(1): p. 101274."

100 "Shen, Z., et al., Effects on Suxiao Jiuxin Pills in the Treatment of Patients with Acute Coronary Syndrome Undergoing Early Percutaneous Coronary Intervention: A Multicenter Randomized Double-Blind Placebo-Controlled Trial. J Altern Complement Med, 2020. 26(11): p. 1055-1063."

101 "Shintani, S., et al., Identification of a truncated cystatin SA-I as a saliva biomarker for oral squamous cell carcinoma using the SELDI ProteinChip platform. International Journal of Oral and Maxillofacial Surgery, 2010. 39(1): p. 68-74."

102 "Sugimoto, K., et al., Urinary Albumin Levels Predict Development of Acute Kidney Injury After Pediatric Cardiac Surgery: A Prospective Observational Study. Journal of Cardiothoracic and Vascular Anesthesia, 2016. 30(1): p. 64-68."

103 "Suominen, A., et al., Long-term renal prognosis and risk for hypertension after myeloablative therapies in survivors of childhood high-risk neuroblastoma: A nationwide study. Pediatr Blood Cancer, 2020. 67(8): p. e28209."

104 "Tamba, K., et al., [Prospective evaluation of renal function by serum cystatin-C: comparison with three other parameters of glomerular filtration rate]. Nihon Jinzo Gakkai Shi, 2001. 43(8): p. 646-50."

105 "Trindade, E.K.G., B.V.M. Silva, and R.F. Dutra, A probeless and label-free electrochemical immunosensor for cystatin C detection based on ferrocene functionalized-graphene platform. Biosensors and Bioelectronics, 2019. 138: p. 111311."

106 "Tsai, C.-Y., et al., Evolution of estimated glomerular filtration rate in HIV/HCV-coinfected patients who received direct-acting antivirals: A multicenter retrospective study. Journal of Microbiology, Immunology and Infection, 2023. 56(4): p. 718-728."

107 "van Westing, A.C., et al., Dairy products and kidney function decline after myocardial infarction: A prospective analysis in the Alpha Omega Cohort. Clinical Nutrition, 2023. 42(8): p. 1501-1509."

108 "Wasinger, V.C., et al., Low Mass Blood Peptides Discriminative of Inflammatory Bowel Disease (IBD) Severity: A Quantitative Proteomic Perspective*. Molecular & Cellular Proteomics, 2016. 15(1): p. 256-265."

109 "Weinberg, E., et al., INFUSE: Rationale and design of a multi-center, open label, collaborative study to treat HRS-AKI with continuous terlipressin infusion. Contemporary Clinical Trials Communications, 2023. 36: p. 101211."

110 "Welsh, P., et al., Prognostic importance of emerging cardiac, inflammatory, and renal biomarkers in chronic heart failure patients with reduced ejection fraction and anaemia: RED-HF study. Eur J Heart Fail, 2018. 20(2): p. 268-277."

111 "Wu, J., et al., Serum Cystatin C Predicts Mortality in HBV-Related Decompensated Cirrhosis. Biomed Res Int, 2019. 2019: p. 7272045."

112 "Wu, X., et al., Assessment of the association of exposure to polycyclic aromatic hydrocarbons, oxidative stress, and inflammation: A cross-sectional study in Augsburg, Germany. International Journal of Hygiene and Environmental Health, 2022. 244: p. 113993."

113 "Xiang, Y., et al., The risk factors for probable REM sleep behavior disorder: A case-control study. Sleep Medicine, 2023. 110: p. 99-105."

114 "Xie, L., et al., Influence of the Interval Between Renal Computed Tomographic Angiography and Kidney Transplantation on Allograft Function: A Prospective, Randomized, Controlled Trial. Transplant Proc, 2015. 47(10): p. 2822-6."

115 "Xu, H., et al., A sample-to-answer quantitative platform for point-of-care testing of biochemical markers in whole blood. Sensors and Actuators B: Chemical, 2020. 308: p. 127750."

116 "Yanagimoto, Y., et al., Improvement of cisplatin-related renal dysfunction by synthetic ghrelin: a prospective randomised phase II trial. Br J Cancer, 2016. 114(12): p. 1318-25."

117 "Yesilipek, M.A., et al., A Phase II, Multicenter, Single-Arm Study to Evaluate the Safety and Efficacy of Deferasirox after Hematopoietic Stem Cell Transplantation in Children with β-Thalassemia Major. Biol Blood Marrow Transplant, 2018. 24(3): p. 613-618."

118 "Yuan, S., et al., Mendelian randomization and clinical trial evidence supports TYK2 inhibition as a therapeutic target for autoimmune diseases. eBioMedicine, 2023. 89: p. 104488."

119 "Zapater, P., et al., Acute effects of dipyrone on renal function in patients with cirrhosis: a randomized controlled trial. Basic Clin Pharmacol Toxicol, 2015. 116(3): p. 257-63."

120 "Zappitelli, M., et al., Derivation and validation of cystatin C-based prediction equations for GFR in children. Am J Kidney Dis, 2006. 48(2): p. 221-30."

121 "Zavvos, V., et al., A prospective, proteomics study identified potential biomarkers of encapsulating peritoneal sclerosis in peritoneal effluent. Kidney International, 2017. 92(4): p. 988-1002."

122 "Zhou, G., et al., Measurement and Estimation of Glomerular Filtration Rate in Children With Neurogenic Bladder: A Prospective Study. Urology, 2022. 170: p. 174-178."

123 "Z?llner, F.G., et al., Pre-clinical functional Magnetic Resonance Imaging part I: The kidney. Zeitschrift für Medizinische Physik, 2014. 24(4): p. 286-306."

124 "孔丽蕊, et al., 肾损伤血清标志物24h内的生物学变异. 邵阳学院学报（自然科学版）, 2022. 19(2): p. 96-103."

125 "李克鹏, 血清胱抑素C与血清肌酐在急性肾损伤患者GFR评估中的比较研究. 2009."

126 "栾如梅, 多中心IgA肾病临床病理特征及尿外泌体环状RNA表达分析. 2021."

Non-cardiac postoperative injuries（1005)

1 "Abe, M., et al., Effects of lipid-lowering therapy with rosuvastatin on kidney function and oxidative stress in patients with diabetic nephropathy. J Atheroscler Thromb, 2011. 18(11): p. 1018-28."

2 "Abouchacra, S., et al., Renal biomarkers for assessment of kidney function in renal transplant recipients: how do they compare? Int Urol Nephrol, 2012. 44(6): p. 1871-6."

3 "Acikel, S., et al., Prevention of contrast-induced impairment of renal function by short-term or long-term statin therapy in patients undergoing elective coronary angiography. Blood Coagul Fibrinolysis, 2010. 21(8): p. 750-7."

4 "Adeoye, J. and P. Thomson, ‘The Double-Edged Sword’ – An hypothesis for Covid-19-induced salivary biomarkers. Medical Hypotheses, 2020. 143: p. 110124."

5 "Agarwal, I., et al., Fibrosis-related biomarkers and large and small vessel disease: The Cardiovascular Health Study. Atherosclerosis, 2015. 239(2): p. 539-546."

6 "Agarwala, A., et al., Biomarkers and degree of atherosclerosis are independently associated with incident atherosclerotic cardiovascular disease in a primary prevention cohort: The ARIC study. Atherosclerosis, 2016. 253: p. 156-163."

7 "Ahiawodzi, P., et al., Non-esterified fatty acids and telomere length in older adults: The Cardiovascular Health Study. Metabolism Open, 2020. 8: p. 100058."

8 "Akerblom, ?., et al., Cystatin C and estimated glomerular filtration rate as predictors for adverse outcome in patients with ST-elevation and non-ST-elevation acute coronary syndromes: results from the Platelet Inhibition and Patient Outcomes study. Clin Chem, 2012. 58(1): p. 190-9."

9 "Aksoy, F., et al., Predictive value of oxidant and antioxidant status for contrast-induced nephropathy after percutaneous coronary intervention for ST-segment elevation myocardial infarction. Revista Portuguesa de Cardiologia (English Edition), 2021. 40(7): p. 489-497."

10 "Al Musaimi, O., et al., Influence of age, gender, smoking, diabetes, thyroid and cardiac dysfunctions on cystatin C biomarker. Medicina de Familia. SEMERGEN, 2019. 45(1): p. 44-51."

11 "Albini, L., et al., A randomized, pilot trial to evaluate glomerular filtration rate by creatinine or cystatin C in naive HIV-infected patients after tenofovir/emtricitabine in combination with atazanavir/ritonavir or efavirenz. J Acquir Immune Defic Syndr, 2012. 59(1): p. 18-30."

12 "Alfageme, I., et al., 10 Years After EPISCAN: A New Study on the Prevalence of COPD in Spain—A Summary of the EPISCAN II Protocol. Archivos de Bronconeumología (English Edition), 2019. 55(1): p. 38-47."

13 "Al-Ghonaim, M. and N. Pannu, Prevention and Treatment of Contrast-Induced Nephropathy. Techniques in Vascular and Interventional Radiology, 2006. 9(2): p. 42-49."

14 "Allwright, M., et al., Machine learning analysis of the UK Biobank reveals prognostic and diagnostic immune biomarkers for polyneuropathy and neuropathic pain in diabetes. Diabetes Research and Clinical Practice, 2023. 201: p. 110725."

15 "Altalhi, S.A., et al., Therapeutic potential and protection enhancement of mesenchymal stem cell against cisplatin-induced nephrotoxicity using hyaluronic acid-chitosan nanoparticles as an adjuvant. International Journal of Pharmaceutics, 2023. 640: p. 123023."

16 "Alvarez, O., et al., Effect of hydroxyurea treatment on renal function parameters: results from the multi-center placebo-controlled BABY HUG clinical trial for infants with sickle cell anemia. Pediatr Blood Cancer, 2012. 59(4): p. 668-74."

17 "André, C., et al., Two rapid, accurate liquid chromatography tandem mass spectrometry methods for the quantification of seven uremic toxins: An application for describing their accumulation kinetic profile in a context of acute kidney injury. Journal of Chromatography B, 2020. 1152: p. 122234."

18 "Aomatsu, A., et al., MicroRNA expression profiling in acute kidney injury. Translational Research, 2022. 244: p. 1-31."

19 "Aparna, R., et al., Detection,discrimination and aging of human tears stains using ATR-FTIR spectroscopy for forensic purposes. Forensic Science International: Reports, 2022. 6: p. 100290."

20 "Araki, H., et al., Safety and efficacy of skin patches containing loxoprofen sodium in diabetic patients with overt nephropathy. Clin Exp Nephrol, 2014. 18(3): p. 487-91."

21 "Arce, C.M., et al., Kidney Function and Cardiovascular Events in Postmenopausal Women: The Impact of Race and Ethnicity in the Women’s Health Initiative. American Journal of Kidney Diseases, 2016. 67(2): p. 198-208."

22 "Ashrafizadeh, M., et al., A bioinformatics analysis, pre-clinical and clinical conception of autophagy in pancreatic cancer: Complexity and simplicity in crosstalk. Pharmacological Research, 2023. 194: p. 106822."

23 "Aslanabadi, N., et al., Pentoxifylline for the prevention of contrast-induced nephropathy in diabetic patients undergoing angioplasty: a randomized controlled trial. Int Urol Nephrol, 2019. 51(4): p. 699-705."

24 "Audzeyenka, I., et al., Cathepsin C is a novel mediator of podocyte and renal injury induced by hyperglycemia. Biochimica et Biophysica Acta (BBA) - Molecular Cell Research, 2020. 1867(8): p. 118723."

25 "Aulin, J., et al., Biomarkers and heart failure events in patients with atrial fibrillation in the ARISTOTLE trial evaluated by a multi-state model. Am Heart J, 2022. 251: p. 13-24."

26 "Aulin, J., et al., Interleukin-6 and C-reactive protein and risk for death and cardiovascular events in patients with atrial fibrillation. Am Heart J, 2015. 170(6): p. 1151-60."

27 "Azzouzi, S., et al., Spatially hierarchical nano-architecture for real time detection of Interleukin-8 cancer biomarker. Talanta, 2022. 246: p. 123436."

28 "Bachorzewska-Gajewska, H., et al., Could neutrophil-gelatinase-associated lipocalin and cystatin C predict the development of contrast-induced nephropathy after percutaneous coronary interventions in patients with stable angina and normal serum creatinine values? Kidney Blood Press Res, 2007. 30(6): p. 408-15."

29 "Ballew, S.H., et al., Frailty, Kidney Function, and Polypharmacy: The?Atherosclerosis Risk in Communities (ARIC) Study. American Journal of Kidney Diseases, 2017. 69(2): p. 228-236."

30 "Bansal, S., Post-surgical acute kidney injury. Clinical Queries: Nephrology, 2012. 1(1): p. 50-57."

31 "Bao, H.L., et al., [Infect of pingshen decoction on serum HGF, Cys C and TGF-beta1 diabetic nephropathy in early stage]. Zhongguo Zhong Yao Za Zhi, 2014. 39(6): p. 1128-31."

32 "Barasch, E., et al., The Associations of Aortic Valve Sclerosis, Aortic Annular Increased Reflectivity, and Mitral Annular Calcification with Subsequent Aortic Stenosis in Older Individuals: Findings from the Cardiovascular Health Study. Journal of the American Society of Echocardiography, 2023. 36(1): p. 41-49.e1."

33 "Barrantes, F., et al., Acute Kidney Injury Predicts Outcomes of Non-Critically Ill Patients. Mayo Clinic Proceedings, 2009. 84(5): p. 410-416."

34 "Barros, O., et al., Multi-omics approach reveals promising salivary protein markers for head and neck squamous cell carcinoma prognosis. Oral Oncology Reports, 2023. 7: p. 100084."

35 "Bastard, J.P., et al., Diabetes and dyslipidaemia are associated with oxidative stress independently of inflammation in long-term antiretroviral-treated HIV-infected patients. Diabetes & Metabolism, 2019. 45(6): p. 573-581."

36 "Bendszus, M., et al., Dose Finding Study of Gadopiclenol, a New Macrocyclic Contrast Agent, in MRI of Central Nervous System. Invest Radiol, 2020. 55(3): p. 129-137."

37 "Ben?hr, P., et al., Cystatin C--a marker for assessment of the glomerular filtration rate in patients with cisplatin chemotherapy. Kidney Blood Press Res, 2006. 29(1): p. 32-5."

38 "Benoit, S.W., et al., A novel strategy for identifying early acute kidney injury in pediatric hematopoietic stem cell transplantation. Bone Marrow Transplant, 2019. 54(9): p. 1453-1461."

39 "Berger, R.P., K. Hymel, and W.-M. Gao, The Use of Biomarkers After Inflicted Traumatic Brain Injury: Insight into Etiology, Pathophysiology, and Biochemistry. Clinical Pediatric Emergency Medicine, 2006. 7(3): p. 186-193."

40 "Beringer, P.M., et al., GFR estimates using cystatin C are superior to serum creatinine in adult patients with cystic fibrosis. J Cyst Fibros, 2009. 8(1): p. 19-25."

41 "Bernard, A. and C. Hermans, Biomonitoring of early effects on the kidney or the lung. Science of The Total Environment, 1997. 199(1): p. 205-211."

42 "Bhattacharjee, S., et al., An in-vivo interpretation for validating the ameliorative efficacy of green synthesized MnO2 nano-conjugate using Carica Papaya (Papaya) leaf extract against acute hepatic damage. Journal of Drug Delivery Science and Technology, 2021. 66: p. 102774."

43 "Bian, Z., R. Zhu, and S. Chen, The predict value of serum/urocystatin C on acute kidney injury in elderly patients with sepsis. Experimental Gerontology, 2021. 155: p. 111576."

44 "Bielecka-Dabrowa, A., et al., Differences in biochemical and genetic biomarkers in patients with heart failure of various etiologies. International Journal of Cardiology, 2016. 221: p. 1073-1080."

45 "Bielecka-Dabrowa, A., et al., Heart failure biomarkers in patients with dilated cardiomyopathy. Int J Cardiol, 2013. 168(3): p. 2404-10."

46 "Bishu, K., et al., Biomarkers in acutely decompensated heart failure with preserved or reduced ejection fraction. Am Heart J, 2012. 164(5): p. 763-770.e3."

47 "Bjornstad, P., et al., ABC goal achievement predicts microvascular but not macrovascular complications over 6-years in adults with type 1 diabetes: The Coronary Artery Calcification in Type 1 Diabetes Study. Journal of Diabetes and its Complications, 2014. 28(6): p. 762-766."

48 "Bjornstad, P., et al., Five-year kidney outcomes of bariatric surgery differ in severely obese adolescents and adults with and without type 2 diabetes. Kidney International, 2020. 97(5): p. 995-1005."

49 "Bjornstad, P., et al., Serum uric acid predicts vascular complications in adults with type 1 diabetes: the coronary artery calcification in type 1 diabetes study. Acta Diabetol, 2014. 51(5): p. 783-91."

50 "Bliss, D., Innovations and controversies in the monitoring of pediatric patients in the ICU. Seminars in Pediatric Surgery, 2015. 24(1): p. 32-36."

51 "B?hme, M., et al., Impact of Clonal Hematopoiesis in Patients With Cardiogenic Shock Complicating Acute Myocardial Infarction. Journal of the American College of Cardiology, 2022. 80(16): p. 1545-1556."

52 "Boldt, J., et al., Influence of volume therapy with a modern hydroxyethylstarch preparation on kidney function in cardiac surgery patients with compromised renal function: a comparison with human albumin. Crit Care Med, 2007. 35(12): p. 2740-6."

53 "Borgo?o, C.A., et al., Expression and Functional Characterization of the Cancer-related Serine Protease, Human Tissue Kallikrein 14*. Journal of Biological Chemistry, 2007. 282(4): p. 2405-2422."

54 "Borkham-Kamphorst, E., et al., Protective effects of lipocalin-2 (LCN2) in acute liver injury suggest a novel function in liver homeostasis. Biochimica et Biophysica Acta (BBA) - Molecular Basis of Disease, 2013. 1832(5): p. 660-673."

55 "Bornebroek, M. and M.M.B. Breteler, Epidemiology of non-AD dementias. Clinical Neuroscience Research, 2004. 3(6): p. 349-361."

56 "Borrows, R., et al., Serum-free light chains adjusted for renal function are a potential biomarker for post-transplant lymphoproliferative disorders. Ann Hematol, 2019. 98(3): p. 625-632."

57 "Bouquegneau, A., et al., Biomarkers and physiopathology in the cardiorenal syndrome. Clinica Chimica Acta, 2015. 443: p. 100-107."

58 "Bracchi, M., et al., A phase IV, open-label three-arm study investigating the impact of a combination of tenofovir disoproxil fumarate/emtricitabine with raltegravir or dolutegravir or elvitegravir/cobicistat on renal function in HIV-1 antiretroviral na?ve patients. HIV Res Clin Pract, 2021. 22(5): p. 128-139."

59 "Breitling, L.P., et al., Gamma-glutamyltransferase and prognosis in patients with stable coronary heart disease followed over 8 years. Atherosclerosis, 2010. 210(2): p. 649-655."

60 "Briguori, C., et al., Renal Insufficiency After Contrast Media Administration Trial II (REMEDIAL II): RenalGuard System in high-risk patients for contrast-induced acute kidney injury. Circulation, 2011. 124(11): p. 1260-9."

61 "Brott, D.A., et al., Characterization of renal biomarkers for use in clinical trials: biomarker evaluation in healthy volunteers. Drug Des Devel Ther, 2014. 8: p. 227-37."

62 "Bu, X., et al., Relation of neutrophil-to-lymphocyte ratio to acute kidney injury in patients with sepsis and septic shock: A retrospective study. International Immunopharmacology, 2019. 70: p. 372-377."

63 "Bueter, M., et al., Renal cytokines improve early after bariatric surgery. Br J Surg, 2010. 97(12): p. 1838-44."

64 "Burchard, G.D., et al., Renal dysfunction in children with uncomplicated, Plasmodium falciparum malaria in Tamale, Ghana. Ann Trop Med Parasitol, 2003. 97(4): p. 345-50."

65 "Burke, T.W., et al., Nasopharyngeal Protein Biomarkers of Acute Respiratory Virus Infection. EBioMedicine, 2017. 17: p. 172-181."

66 "Buscher, K., et al., Plasma protein signatures reflect systemic immunity and allograft function in kidney transplantation. Translational Research, 2023. 262: p. 35-43."

67 "Buyukaydin, B., et al., The effect of sleep apnea syndrome on the development of diabetic nephropathy in patients with type 2 diabetes. Diabetes Research and Clinical Practice, 2012. 98(1): p. 140-143."

68 "Cabras, T., et al., Alterations of the Salivary Secretory Peptidome Profile in Children Affected by Type 1 Diabetes. Molecular & Cellular Proteomics, 2010. 9(10): p. 2099-2108."

69 "Cai, L., et al., Combination of serum CST4 and DR-70 contributes to early diagnosis of colorectal cancer. Clinica Chimica Acta, 2022. 531: p. 318-324."

70 "Campbell, L.J., et al., Total protein, albumin and low-molecular-weight protein excretion in HIV-positive patients. BMC Nephrol, 2012. 13: p. 85."

71 "Cantinotti, M., et al., Diagnostic accuracy and prognostic valued of plasmatic Cystatin-C in children undergoing pediatric cardiac surgery. Clinica Chimica Acta, 2017. 471: p. 113-118."

72 "Cao, Z., et al., ACSL1: A preliminary study that provides a new target for the treatment of renal fibrosis could bring new insights in diabetic kidney disease. Nefrología, 2023. 43: p. 38-46."

73 "Captur, G., et al., Plasma proteomic signature predicts who will get persistent symptoms following SARS-CoV-2 infection. eBioMedicine, 2022. 85: p. 104293."

74 "Carino, M., et al., Comparison of Clinical and Social Characteristics of Canadian Youth Living With Type 1 and Type 2 Diabetes. Canadian Journal of Diabetes, 2021. 45(5): p. 428-435."

75 "Carnicelli, A.P., et al., Individual Patient Data from the Pivotal Randomized Controlled Trials of Non-Vitamin K Antagonist Oral Anticoagulants in Patients with Atrial Fibrillation (COMBINE AF): Design and Rationale: From the COMBINE AF (A Collaboration between Multiple institutions to Better Investigate Non-vitamin K antagonist oral anticoagulant use in Atrial Fibrillation) Investigators. American Heart Journal, 2021. 233: p. 48-58."

76 "Cavusoglu, E., et al., Elevated baseline plasma phospholipid protein (PLTP) levels are an independent predictor of long-term all-cause mortality in patients with diabetes mellitus and known or suspected coronary artery disease. Atherosclerosis, 2015. 239(2): p. 503-508."

77 "Ceccarelli Ceccarelli, D., et al., Re-thinking diabetic nephropathy: Microalbuminuria is just a piece of the diagnostic puzzle. Clinica Chimica Acta, 2022. 524: p. 146-153."

78 "Cepeda, F.J., et al., [Utility of cystatin-C in hospitalized patients. Comparing with different methods of assessing renal function]. Nefrologia, 2007. 27(2): p. 168-74."

79 "Chan, K.W., et al., Semi-individualised Chinese medicine treatment as an adjuvant management for diabetic nephropathy: a pilot add-on, randomised, controlled, multicentre, open-label pragmatic clinical trial. BMJ Open, 2016. 6(8): p. e010741."

80 "Chao, C.-T., et al., Advanced age affects the outcome-predictive power of RIFLE classification in geriatric patients with acute kidney injury. Kidney International, 2012. 82(8): p. 920-927."

81 "Chen, D.C., et al., Modification of Association of Cystatin C With Kidney and Cardiovascular Outcomes by Obesity. American Journal of Kidney Diseases, 2024. 83(4): p. 489-496.e1."

82 "Chen, T., et al., Risk factors for peripheral artery disease and diabetic peripheral neuropathy among patients with type 2 diabetes. Diabetes Research and Clinical Practice, 2024. 207: p. 111079."

83 "Cheng, J., et al., A low-cost paper-based blood urea nitrogen optical biosensor for renal surveillance in fingertip blood. Sensors and Actuators B: Chemical, 2023. 387: p. 133795."

84 "Chi, L.-M., et al., Assessment of candidate biomarkers in paired saliva and plasma samples from oral cancer patients by targeted mass spectrometry. Journal of Proteomics, 2020. 211: p. 103571."

85 "Chiang, H.-L., et al., Analyses of transthyretin concentration in the cerebrospinal fluid of patients with Guillain-Barré syndrome and other neurological disorders. Clinica Chimica Acta, 2009. 405(1): p. 143-147."

86 "Chiasserini, D., et al., Proteomic analysis of cerebrospinal fluid extracellular vesicles: A comprehensive dataset. Journal of Proteomics, 2014. 106: p. 191-204."

87 "Chimonidou, M., et al., CST6 promoter methylation in circulating cell-free DNA of breast cancer patients. Clinical Biochemistry, 2013. 46(3): p. 235-240."

88 "Chiu, K.-H., Y.-H. Chang, and P.-C. Liao, Secretome analysis using a hollow fiber culture system for cancer biomarker discovery. Biochimica et Biophysica Acta (BBA) - Proteins and Proteomics, 2013. 1834(11): p. 2285-2292."

89 "Chivte, P., et al., MALDI-ToF protein profiling as a potential rapid diagnostic platform for COVID-19. Journal of Mass Spectrometry and Advances in the Clinical Lab, 2021. 21: p. 31-41."

90 "Cho, A.R. and S.Y. Lee, Biomarkers and their relative contributions to identifying coronary artery stenosis based on coronary computed tomography angiography in asymptomatic adults. Clinica Chimica Acta, 2019. 499: p. 128-133."

91 "Cho, W.C.-S., Research progress in SELDI-TOF MS and its clinical applications. Chinese Journal of Biotechnology, 2006. 22(6): p. 871-877."

92 "Choi, G.S., et al., SH3YL1 protein as a novel biomarker for diabetic nephropathy in type 2 diabetes mellitus. Nutrition, Metabolism and Cardiovascular Diseases, 2021. 31(2): p. 498-505."

93 "Choi, Y.S., et al., Effect of remote ischemic preconditioning on renal dysfunction after complex valvular heart surgery: a randomized controlled trial. J Thorac Cardiovasc Surg, 2011. 142(1): p. 148-54."

94 "Chowdhury, S.R., 血清胱抑素、C反应蛋白和同型半胱氨酸作为生物标志物在2型糖尿病视网膜病变中的作用. 2016."

95 "Chrysohoou, C., et al., Short term omega-3 polyunsaturated fatty acid supplementation induces favorable changes in right ventricle function and diastolic filling pressure in patients with chronic heart failure; A randomized clinical trial. Vascular Pharmacology, 2016. 79: p. 43-50."

96 "Chumbalkar, V., R. Sawaya, and O. Bogler, Proteomics: The New Frontier Also for Brain Tumor Research. Current Problems in Cancer, 2008. 32(3): p. 143-154."

97 "Cicek, M., et al., Use of alpha-lipoic acid in prevention of contrast-induced nephropathy in diabetic patients. Ren Fail, 2013. 35(5): p. 748-53."

98 "Coca, S.G., et al., Plasma Soluble Tumor Necrosis Factor Receptor Concentrations and Clinical Events After Hospitalization: Findings From the ASSESS-AKI and ARID Studies. American Journal of Kidney Diseases, 2023. 81(2): p. 190-200."

99 "Codo?er-Franch, P., et al., Retinol-Binding Protein 4 Levels Are Associated with Measures of Liver and Renal Function and Oxidant/Antioxidant Status in Obese Children. The Journal of Pediatrics, 2013. 163(2): p. 593-595."

100 "Cs?sz, é., et al., Diabetic retinopathy: Proteomic approaches to help the differential diagnosis and to understand the underlying molecular mechanisms. Journal of Proteomics, 2017. 150: p. 351-358."

101 "Cs?sz, é., et al., Quantitative analysis of proteins in the tear fluid of patients with diabetic retinopathy. Journal of Proteomics, 2012. 75(7): p. 2196-2204."

102 "Cui, X., et al., [Volume management of intermittent hemofiltration guided by critical care ultrasound in the treatment of acute kidney injury]. Zhonghua Wei Zhong Bing Ji Jiu Yi Xue, 2023. 35(3): p. 310-315."

103 "Daher, A., et al., Follow up of patients with severe coronavirus disease 2019 (COVID-19): Pulmonary and extrapulmonary disease sequelae. Respiratory Medicine, 2020. 174: p. 106197."

104 "Dalcomune, D.M., et al., Predictive value of cystatin C for the identification of illness severity in adult patients in a mixed intensive care unit. Clinical Biochemistry, 2016. 49(10): p. 762-767."

105 "Dastan, F., et al., Randomized Trial of Carnitine for the Prevention of Perioperative Atrial Fibrillation. Seminars in Thoracic and Cardiovascular Surgery, 2018. 30(1): p. 7-13."

106 "Davey, P., et al., Impact on renal function after endovascular aneurysm repair with uncovered supra-renal fixation assessed by serum cystatin C. Eur J Vasc Endovasc Surg, 2008. 35(4): p. 439-45."

107 "Davis, J., et al., Early diagnosis of acute kidney injury subsequent to severe hypotension and fluid resuscitation in anaesthetized dogs. Veterinary Anaesthesia and Analgesia, 2022. 49(4): p. 344-353."

108 "De Carvalho, J.A.M., et al., Assessment of urinary γ-glutamyltransferase and alkaline phosphatase for diagnosis of diabetic nephropathy. Clinica Chimica Acta, 2011. 412(15): p. 1407-1411."

109 "de Oliveira, T.M., et al., Label-free peptide quantification coupled with in silico mapping of proteases for identification of potential serum biomarkers in gastric adenocarcinoma patients. Clinical Biochemistry, 2020. 79: p. 61-69."

110 "de Peyster, A., et al., Responses of the steroidogenic pathway from exposure to methyl-tert-butyl ether and tert-butanol. Toxicology, 2014. 319: p. 23-37."

111 "Defilippi, C.R., et al., Assessment of Biomarkers of Myocardial injury, Inflammation, and Renal Function in Heart Failure With Reduced Ejection Fraction: The VICTORIA Biomarker Substudy. Journal of Cardiac Failure, 2023. 29(4): p. 448-458."

112 "Degen, D.A., et al., Predictive performance of different kidney function estimation equations in lung transplant patients. Clin Biochem, 2017. 50(7-8): p. 385-393."

113 "del Pilar Chantada-Vázquez, M., et al., Proteomic investigation on bio-corona of Au, Ag and Fe nanoparticles for the discovery of triple negative breast cancer serum protein biomarkers. Journal of Proteomics, 2020. 212: p. 103581."

114 "Demichev, V., et al., A time-resolved proteomic and prognostic map of COVID-19. Cell Systems, 2021. 12(8): p. 780-794.e7."

115 "Demirta?, S., et al., Diagnostic value of serum cystatin C for evaluation of hepatorenal syndrome. Clin Chim Acta, 2001. 311(2): p. 81-9."

116 "Deng, R., et al., Association of CYP3A5, CYP2C8, and ABCB1 Polymorphisms With Early Renal Injury in Chinese Liver Transplant Recipients Receiving Tacrolimus. Transplantation Proceedings, 2018. 50(10): p. 3258-3265."

117 "Di Domenico, F., et al., Oxidative signature of cerebrospinal fluid from mild cognitive impairment and Alzheimer disease patients. Free Radical Biology and Medicine, 2016. 91: p. 1-9."

118 "Ding, D., et al., Prognostic significance of peripheral blood S100A12, S100A8, and S100A9 concentrations in idiopathic pulmonary fibrosis. Cytokine, 2023. 172: p. 156387."

119 "Diorio, C., et al., Evidence of thrombotic microangiopathy in children with SARS-CoV-2 across the spectrum of clinical presentations. Blood Advances, 2020. 4(23): p. 6051-6063."

120 "Dirajlal-Fargo, S., et al., Statin therapy decreases N-terminal pro-B-type natriuretic peptide in HIV: randomized placebo-controlled trial. Aids, 2015. 29(3): p. 313-21."

121 "Dorkhan, M., et al., Glycaemic and nonglycaemic effects of pioglitazone in triple oral therapy of patients with type 2 diabetes. J Intern Med, 2006. 260(2): p. 125-33."

122 "Drey, M., et al., C-terminal agrin fragment (CAF) reflects renal function in patients suffering from severe sepsis or septic shock. Clin Lab, 2015. 61(1-2): p. 69-76."

123 "Du, X., H. Bao, and D. Zhao, Efficacy and safety of combined doxofylline and salbutamol in treatment of acute exacerbation of chronic obstructive pulmonary disease. Rev Assoc Med Bras (1992), 2021. 67(9): p. 1256-1260."

124 "Durkan, A.M. and R.T. Alexander, Acute Kidney Injury Post Neonatal Asphyxia. The Journal of Pediatrics, 2011. 158(2, Supplement): p. e29-e33."

125 "El-Akabawy, H., et al., Urinary neutrophil gelatinase associated lipocalin as an early marker of acute kidney injury in the recipient after liver transplantation. The Egyptian Journal of Critical Care Medicine, 2017. 5(1): p. 49-55."

126 "Elbarbary, N.S., et al., Vitamin B complex supplementation as a homocysteine-lowering therapy for early stage diabetic nephropathy in pediatric patients with type 1 diabetes: A randomized controlled trial. Clin Nutr, 2020. 39(1): p. 49-56."

127 "El-Ghiaty, M.A., et al., Evaluation of the protective effect of Cystone against cisplatin-induced nephrotoxicity in cancer patients, and its influence on cisplatin antitumor activity. Int Urol Nephrol, 2014. 46(7): p. 1367-73."

128 "Elsayed, M.S., et al., Serum cystatin C as an indicator for early detection of diabetic nephropathy in type 2 diabetes mellitus. Diabetes & Metabolic Syndrome: Clinical Research & Reviews, 2019. 13(1): p. 374-381."

129 "Emans, M.E., et al., Determinants of Red Cell Distribution Width (RDW) in Cardiorenal Patients: RDW is Not Related to Erythropoietin Resistance. Journal of Cardiac Failure, 2011. 17(8): p. 626-633."

130 "Erbel, R., et al., Coronary Risk Stratification, Discrimination, and Reclassification Improvement Based on Quantification of Subclinical Coronary Atherosclerosis: The Heinz Nixdorf Recall Study. Journal of the American College of Cardiology, 2010. 56(17): p. 1397-1406."

131 "Eriksson, P., et al., Genetic approach to the role of cysteine proteases in the expansion of abdominal aortic aneurysms. Br J Surg, 2004. 91(1): p. 86-9."

132 "Ferraro, S., et al., Inside ST-elevation myocardial infarction by monitoring concentrations of cardiovascular risk biomarkers in blood. Clinica Chimica Acta, 2012. 413(9): p. 888-893."

133 "Flores-Guerrero, J.L., et al., Triglyceride/HDL cholesterol ratio and lipoprotein insulin resistance Score: Associations with subclinical atherosclerosis and incident cardiovascular disease. Clinica Chimica Acta, 2024. 553: p. 117737."

134 "Folsom, A.R., et al., Parathyroid hormone concentration and risk of cardiovascular diseases: The Atherosclerosis Risk in Communities (ARIC) study. American Heart Journal, 2014. 168(3): p. 296-302."

135 "Fonseca, C.D.d., et al., The renoprotective effects of Heme Oxygenase-1 during contrast-induced acute kidney injury in preclinical diabetic models. Clinics, 2021. 76: p. e3002."

136 "Forsblom, C., et al., Effects of long-term fenofibrate treatment on markers of renal function in type 2 diabetes: the FIELD Helsinki substudy. Diabetes Care, 2010. 33(2): p. 215-20."

137 "Forst, T., et al., Microvascular effects of the inhibition of dipeptidylpeptidase IV by linagliptin in nondiabetic hypertensive patients. J Hypertens, 2016. 34(2): p. 345-50."

138 "Foster, M.C., et al., Filtration Markers as Predictors of ESRD and Mortality in Southwestern American Indians With Type 2 Diabetes. American Journal of Kidney Diseases, 2015. 66(1): p. 75-83."

139 "Fox, E., et al., Pantoprazole, an Inhibitor of the Organic Cation Transporter 2, Does Not Ameliorate Cisplatin-Related Ototoxicity or Nephrotoxicity in Children and Adolescents with Newly Diagnosed Osteosarcoma Treated with Methotrexate, Doxorubicin, and Cisplatin. Oncologist, 2018. 23(7): p. 762-e79."

140 "Freedman, B.I., R.S. Parekh, and W.H.L. Kao, Genetic Basis of Nondiabetic End-Stage Renal Disease. Seminars in Nephrology, 2010. 30(2): p. 101-110."

141 "Freitas, M., et al., High-performance electrochemical immunomagnetic assay for breast cancer analysis. Sensors and Actuators B: Chemical, 2020. 308: p. 127667."

142 "Freitas, M., et al., Quantum dots as nanolabels for breast cancer biomarker HER2-ECD analysis in human serum. Talanta, 2020. 208: p. 120430."

143 "Friedman, A.N., et al., Measuring the glomerular filtration rate in obese individuals without overt kidney disease. Nephron Clin Pract, 2010. 116(3): p. c224-34."

144 "Friedman, A.N., et al., Short-term changes after a weight reduction intervention in advanced diabetic nephropathy. Clin J Am Soc Nephrol, 2013. 8(11): p. 1892-8."

145 "Friedman, S., et al., Efficacy and safety study of cenicriviroc for the treatment of non-alcoholic steatohepatitis in adult subjects with liver fibrosis: CENTAUR Phase 2b study design. Contemporary Clinical Trials, 2016. 47: p. 356-365."

146 "Fu, N., M. Liang, and S. Yang, High Loading Dose of Atorvastatin for the Prevention of Serum Creatinine and Cystatin C-Based Contrast-Induced Nephropathy Following Percutaneous Coronary Intervention. Angiology, 2018. 69(8): p. 692-699."

147 "Fu, W.-J., et al., Changes of the tubular markers in type 2 diabetes mellitus with glomerular hyperfiltration. Diabetes Research and Clinical Practice, 2012. 95(1): p. 105-109."

148 "Fu, X., et al., A new index based on serum creatinine and cystatin C is useful for assessing sarcopenia in patients with advanced cancer. Nutrition, 2021. 82: p. 111032."

149 "Fugmann, T., et al., Proteomic identification of vanin-1 as a marker of kidney damage in a rat model of type 1 diabetic nephropathy. Kidney International, 2011. 80(3): p. 272-281."

150 "Fung, E.T., et al., A biomarker panel for peripheral arterial disease. Vasc Med, 2008. 13(3): p. 217-24."

151 "Gale, J.D., et al., Effect of PF-04634817, an Oral CCR2/5 Chemokine Receptor Antagonist, on Albuminuria in Adults with Overt Diabetic Nephropathy. Kidney International Reports, 2018. 3(6): p. 1316-1327."

152 "Gallagher, S.M., et al., Remote ischemic preconditioning has a neutral effect on the incidence of kidney injury after coronary artery bypass graft surgery. Kidney Int, 2015. 87(2): p. 473-81."

153 "Ganugula, R., et al., Nanocurcumin combined with insulin alleviates diabetic kidney disease through P38/P53 signaling axis. Journal of Controlled Release, 2023. 353: p. 621-633."

154 "Gao, X., et al., Association between serum pepsinogen and atherosclerotic cardiovascular disease. Nutrition, Metabolism and Cardiovascular Diseases, 2021. 31(1): p. 169-177."

155 "García-Berrocoso, T., et al., From brain to blood: New biomarkers for ischemic stroke prognosis. Journal of Proteomics, 2013. 94: p. 138-148."

156 "Garcia-Carretero, R., et al., Cardiovascular risk assessment in prediabetic patients in a hypertensive population: The role of cystatin C. Diabetes & Metabolic Syndrome: Clinical Research & Reviews, 2018. 12(5): p. 625-629."

157 "Ghanipour, L., et al., Detection of Biomarkers with Solid-Phase Proximity Ligation Assay in Patients with Colorectal Cancer. Translational Oncology, 2016. 9(3): p. 251-255."

158 "Ghelich Khan, Z., et al., Potential Role of Allopurinol in Preventing Contrast-Induced Nephropathy in Patients Undergoing Percutaneous Coronary Intervention: A Randomized Placebo-Controlled Trial. Clin Drug Investig, 2017. 37(9): p. 853-860."

159 "Gon?alves, M., et al., Phosphoneurofilament heavy chain and N-glycomics from the cerebrospinal fluid in amyotrophic lateral sclerosis. Clinica Chimica Acta, 2015. 438: p. 342-349."

160 "Gonwa, T.A. and H.M. Wadei, Kidney Disease in the Setting of Liver Failure: Core Curriculum 2013. American Journal of Kidney Diseases, 2013. 62(6): p. 1198-1212."

161 "Gr?nborg, M., et al., Biomarker Discovery from Pancreatic Cancer Secretome Using a Differential Proteomic Approach*S. Molecular & Cellular Proteomics, 2006. 5(1): p. 157-171."

162 "Gu, Z., et al., The application of neutrophil gelatin-related lipid delivery protein in evaluation of renal function, nutrition, anemia and inflammation in patients with CKD. Néphrologie & Thérapeutique, 2021. 17(1): p. 35-41."

163 "Guerchicoff, A., et al., Analysis of biomarkers for risk of acute kidney injury after primary angioplasty for acute ST-segment elevation myocardial infarction: results of the HORIZONS-AMI trial. Catheter Cardiovasc Interv, 2015. 85(3): p. 335-42."

164 "Guías de práctica clínica para el tratamiento de la hipertensión arterial 2007. Revista Espa?ola de Cardiología, 2007. 60(9): p. 968.e1-968.e94."

165 "Gulati, P., et al., Nano-modified screen-printed electrode-based electrochemical immunosensors for oral cancer biomarker detection in undiluted human serum and saliva samples??Electronic supplementary information (ESI) available. See DOI: https://doi.org/10.1039/d3na00682d. Nanoscale Advances, 2024. 6(2): p. 705-721."

166 "Guo, K., et al., Expression and significance of Cystatin-C in clear cell renal cell carcinoma. Biomedicine & Pharmacotherapy, 2018. 107: p. 1237-1245."

167 "Guo, N., et al., Identification of plasma proteins associated with oesophageal cancer chemotherapeutic treatment outcomes using SWATH-MS. Journal of Proteomics, 2022. 266: p. 104684."

168 "Halacova, M., et al., Serum cystatin C level for better assessment of glomerular filtration rate in cystic fibrosis patients treated by amikacin. J Clin Pharm Ther, 2008. 33(4): p. 409-17."

169 "Han, R.X., et al., Detection of early pregnancy-specific proteins in Holstein milk. Journal of Proteomics, 2012. 75(11): p. 3221-3229."

170 "Hanssen, N.M.J., et al., Fasting and post-oral-glucose-load levels of methylglyoxal are associated with microvascular, but not macrovascular, disease in individuals with and without (pre)diabetes: The Maastricht Study. Diabetes & Metabolism, 2021. 47(1): p. 101148."

171 "Harris, V.K., et al., Bri2-23 is a potential cerebrospinal fluid biomarker in multiple sclerosis. Neurobiology of Disease, 2010. 40(1): p. 331-339."

172 "Hart, A., et al., Identification of prognostic biomarkers for antibiotic associated nephrotoxicity in cystic fibrosis. Journal of Cystic Fibrosis, 2024. 23(2): p. 293-299."

173 "Harten, J., et al., Effect of intraoperative fluid optimisation on renal function in patients undergoing emergency abdominal surgery: a randomised controlled pilot study (ISRCTN 11799696). Int J Surg, 2008. 6(3): p. 197-204."

174 "Hassan, M., et al., Urinary cystatin C as a biomarker of early renal dysfunction in type 2 diabetic patients. Diabetes & Metabolic Syndrome: Clinical Research & Reviews, 2021. 15(4): p. 102152."

175 "Hawkesworth, S., et al., Combined food and micronutrient supplements during pregnancy have limited impact on child blood pressure and kidney function in rural Bangladesh. J Nutr, 2013. 143(5): p. 728-34."

176 "Heine, G., et al., High-resolution peptide mapping of cerebrospinal fluid: a novel concept for diagnosis and research in central nervous system diseases. Journal of Chromatography B, 2002. 782(1): p. 353-361."

177 "Hendriks, F.K., et al., Intradialytic Protein Ingestion and Exercise do Not Compromise Uremic Toxin Removal Throughout Hemodialysis. J Ren Nutr, 2023. 33(2): p. 376-385."

178 "Hermans, M.P., S.A. Ahn, and M.F. Rousseau, Increased CRP: An extended biomarker of microvascular risk in men with type 2 diabetes. Journal of Diabetes and its Complications, 2019. 33(11): p. 107413."

179 "Heunisch, F., et al., Urinary ET-1 excretion after exposure to radio-contrast media in diabetic patients and patients with preexisting mild impaired renal function. Life Sciences, 2014. 118(2): p. 440-445."

180 "Hijazi, Z., et al., Biomarkers of inflammation and risk of cardiovascular events in anticoagulated patients with atrial fibrillation. Heart, 2016. 102(7): p. 508-17."

181 "Hijazi, Z., et al., The novel biomarker-based ABC (age, biomarkers, clinical history)-bleeding risk score for patients with atrial fibrillation: a derivation and validation study. Lancet, 2016. 387(10035): p. 2302-2311."

182 "Hilmi, I.A., et al., N-acetylcysteine does not prevent hepatorenal ischaemia-reperfusion injury in patients undergoing orthotopic liver transplantation. Nephrol Dial Transplant, 2010. 25(7): p. 2328-33."

183 "Hirai, K., et al., Serum creatinine/cystatin C ratio as a surrogate marker for sarcopenia in patients with chronic obstructive pulmonary disease. Clinical Nutrition, 2021. 40(3): p. 1274-1280."

184 "Hjort, M., et al., Differences in biomarker concentrations and predictions of long-term outcome in patients with ST-elevation and non-ST-elevation myocardial infarction. Clinical Biochemistry, 2021. 98: p. 17-23."

185 "Hmmier, A., et al., Proteomic analysis of bronchoalveolar lavage fluid (BALF) from lung cancer patients using label-free mass spectrometry. BBA Clinical, 2017. 7: p. 97-104."

186 "Hohnloser, S.H., et al., Efficacy of apixaban when compared with warfarin in relation to renal function in patients with atrial fibrillation: insights from the ARISTOTLE trial. Eur Heart J, 2012. 33(22): p. 2821-30."

187 "Hollinger, A., et al., Proenkephalin A 119-159 (Penkid) Is an Early Biomarker of Septic Acute Kidney Injury: The Kidney in Sepsis and Septic Shock (Kid-SSS) Study. Kidney International Reports, 2018. 3(6): p. 1424-1433."

188 "Holmstr?m, A., et al., Levels of copeptin among elderly patients in relation to systolic heart failure and heart failure with normal ejection fraction. European Geriatric Medicine, 2013. 4(3): p. 139-144."

189 "Hou, Y.Y., et al., Effects of differential-phase remote ischemic preconditioning intervention in laparoscopic partial nephrectomy: A single blinded, randomized controlled trial in a parallel group design. J Clin Anesth, 2017. 41: p. 21-28."

190 "Hou, Y.-y., et al., Effects of differential-phase remote ischemic preconditioning intervention in laparoscopic partial nephrectomy: A single blinded, randomized controlled trial in a parallel group design. Journal of Clinical Anesthesia, 2017. 41: p. 21-28."

191 "Hu, G., Z.-S. Xia, and X. Guo, Differential expression of serum GBP-28, NBP-Cyc 3 and TIMP-1 complicates pregnancy in hypertensive disorder pregnancy. Journal of Reproductive Immunology, 2021. 144: p. 103288."

192 "Hu, Y., et al., Comparative Proteomic Analysis of Intra- and Interindividual Variation in Human Cerebrospinal Fluid. Molecular & Cellular Proteomics, 2005. 4(12): p. 2000-2009."

193 "Huang, J.-T., et al., A Highly Sensitive and Robust Method for Hepatitis B Virus Covalently Closed Circular DNA Detection in Single Cells and Serum. The Journal of Molecular Diagnostics, 2018. 20(3): p. 334-343."

194 "Huang, Y., et al., Elevated serum trimethylamine oxide levels as potential biomarker for diabetic kidney disease. Endocrine Connections, 2023. 12(8)."

195 "Hungund, S.A., et al., Efficacy of nonsurgical periodontal therapy affecting salivary biomarkers in non-diabetic and type 2 diabetic periodontitis patients. An observational study. Journal of Oral Biology and Craniofacial Research, 2023. 13(4): p. 500-505."

196 "Ibrahim, N.E., et al., Blood kidney injury molecule–1 predicts short and longer term kidney outcomes in patients undergoing diagnostic coronary and/or peripheral angiography—Results from the Catheter Sampled Blood Archive in Cardiovascular Diseases (CASABLANCA) study. American Heart Journal, 2019. 209: p. 36-46."

197 "Igarashi, M., et al., Dual blockade of angiotensin II with enalapril and losartan reduces proteinuria in hypertensive patients with type 2 diabetes. Endocr J, 2006. 53(4): p. 493-501."

198 "Iliadis, F., et al., Glomerular filtration rate estimation in patients with type 2 diabetes: creatinine- or cystatin C-based equations? Diabetologia, 2011. 54(12): p. 2987-94."

199 "Imai, A., et al., Serum cystatin C is associated with early stage coronary atherosclerotic plaque morphology on multidetector computed tomography. Atherosclerosis, 2011. 218(2): p. 350-355."

200 "Isakova, T., et al., Change in estimated glomerular filtration rate and fracture risk in the Action to Control Cardiovascular Risk in Diabetes Trial. Bone, 2015. 78: p. 23-27."

201 "Isobe, S., et al., Relationship between pre-procedural microalbuminuria and renal functional changes after coronary computed tomography in diabetic patients. Journal of Cardiology, 2017. 69(4): p. 666-670."

202 "Jackson, C.E., et al., Combined Free Light Chains Are Novel Predictors of Prognosis in Heart Failure. JACC: Heart Failure, 2015. 3(8): p. 618-625."

203 "Jakhotia, S., et al., Circulating levels of Hsp27 in microvascular complications of diabetes: Prospects as a biomarker of diabetic nephropathy. Journal of Diabetes and its Complications, 2018. 32(2): p. 221-225."

204 "Jana, S., et al., Early diagnostic biomarkers for acute kidney injury using cisplatin-induced nephrotoxicity in rat model. Current Research in Toxicology, 2023. 5: p. 100135."

205 "Janickova Zdarska, D., E. Zavadova, and M. Kvapil, The effect of ramipril therapy on cytokines and parameters of incipient diabetic nephropathy in patients with type 1 diabetes mellitus. J Int Med Res, 2007. 35(3): p. 374-83."

206 "Jennersj?, P.E.s., et al., Circadian blood pressure variation in patients with type 2 diabetes – relationship to macro- and microvascular subclinical organ damage. Primary Care Diabetes, 2011. 5(3): p. 167-173."

207 "Jesudason, D.R., E. Pedersen, and P.M. Clifton, Weight-loss diets in people with type 2 diabetes and renal disease: a randomized controlled trial of the effect of different dietary protein amounts. Am J Clin Nutr, 2013. 98(2): p. 494-501."

208 "Ji, Y., B. Shi, and Y. Li, An evolutionary machine learning for multiple myeloma using Runge Kutta Optimizer from multi characteristic indexes. Computers in Biology and Medicine, 2022. 150: p. 106189."

209 "Jiang, Z., et al., Curative Effects of Valsartan Alone or Combined with Alpha-lipoic Acid on Inflammatory Cytokines and Renal Function in Early-stage Diabetic Kidney Disease. J Coll Physicians Surg Pak, 2019. 29(10): p. 1009-1011."

210 "Jiménez-Córdova, M.I., et al., Evaluation of kidney injury biomarkers in an adult Mexican population environmentally exposed to fluoride and low arsenic levels. Toxicology and Applied Pharmacology, 2018. 352: p. 97-106."

211 "Johar, D. and L. Bernstein, A targeted approach toward more accurate assessment of hypertension. Egyptian Journal of Chest Diseases and Tuberculosis, 2017. 66(3): p. 517-536."

212 "Jou, Y.-J., et al., S100A8 as potential salivary biomarker of oral squamous cell carcinoma using nanoLC–MS/MS. Clinica Chimica Acta, 2014. 436: p. 121-129."

213 "Kaiser, T., et al., Limited comparability of creatinine assays in patients with liver cirrhosis and their impact on the MELD score. Practical Laboratory Medicine, 2017. 8: p. 41-48."

214 "Kalayjian, R.C., et al., Plasma Cystatin C Associates With HIV-Associated Neurocognitive Disorder but Is a Poor Diagnostic Marker in Antiretroviral Therapy-Treated Individuals. J Acquir Immune Defic Syndr, 2019. 81(2): p. e49-e54."

215 "Kalimeris, K., et al., Mannitol and renal dysfunction after endovascular aortic aneurysm repair procedures: a randomized trial. J Cardiothorac Vasc Anesth, 2014. 28(4): p. 954-9."

216 "Kanmaz, H.G., et al., Does enteral protein intake affect renal glomerular and tubular functions in very low birth weight infants? Clin Nephrol, 2013. 80(5): p. 355-60."

217 "Karademir, L.D., et al., The efficacy of theophylline in preventing cisplatin-related nephrotoxicity in patients with cancer. Ren Fail, 2016. 38(5): p. 806-14."

218 "Kemmler, W., et al., Safety of a Combined WB-EMS and High-Protein Diet Intervention in Sarcopenic Obese Elderly Men. Clin Interv Aging, 2020. 15: p. 953-967."

219 "Kessoku, T., et al., Efficacy, safety, and tolerability of lubiprostone for the treatment of non-alcoholic fatty liver disease in adult patients with constipation: The LUBIPRONE, double-blind, randomised, placebo-controlled study design. Contemporary Clinical Trials, 2018. 69: p. 40-47."

220 "Khurshid, Z., et al., Chapter Two - Role of Salivary Biomarkers in Oral Cancer Detection, in Advances in Clinical Chemistry, G.S. Makowski, Editor. 2018, Elsevier. p. 23-70."

221 "Kiessling, A.H., et al., Early postoperative serum cystatin C predicts severe acute kidney injury following cardiac surgery: a post-hoc analysis of a randomized controlled trial. J Cardiothorac Surg, 2014. 9: p. 10."

222 "Kikkert, W.J., et al., Relationship between biomarkers and subsequent bleeding risk in ST-segment elevation myocardial infarction patients treated with paclitaxel-eluting stents: a HORIZONS-AMI substudy. J Thromb Thrombolysis, 2013. 35(2): p. 200-8."

223 "Kim, C., et al., Significance of Alanine Aminopeptidase N (APN) in Bile in the Diagnosis of Acute Cellular Rejection After Liver Transplantation. Journal of Surgical Research, 2012. 175(1): p. 138-148."

224 "Kim, H., et al., Exercise and Nutritional Supplementation on Community-Dwelling Elderly Japanese Women With Sarcopenic Obesity: A Randomized Controlled Trial. J Am Med Dir Assoc, 2016. 17(11): p. 1011-1019."

225 "Kim, J.H., et al., Nonalbumin proteinuria is a simple and practical predictor of the progression of early-stage type 2 diabetic nephropathy. Journal of Diabetes and its Complications, 2017. 31(2): p. 395-399."

226 "Kim, Y.-e., et al., Proteinuria as a significant predictive factor for the progression of carotid artery atherosclerosis in non-albuminuric type 2 diabetes. Diabetes Research and Clinical Practice, 2021. 181: p. 109082."

227 "Kimmel, M., et al., Improved estimation of glomerular filtration rate by serum cystatin C in preventing contrast induced nephropathy by N-acetylcysteine or zinc--preliminary results. Nephrol Dial Transplant, 2008. 23(4): p. 1241-5."

228 "Klinkenberg, L.J.J., et al., Cardiac Troponin T and I Release After a 30-km Run. The American Journal of Cardiology, 2016. 118(2): p. 281-287."

229 "Knapik-Kordecka, M., A. Piwowar, and M. Warwas, [Levels of cystatin C, activity of antipapain and antitrypsin in plasma of patients with diabetes mellitus type 2]. Wiad Lek, 2000. 53(11-12): p. 617-22."

230 "Knebel, F., et al., Myocardial Function in Older Male Amateur Marathon Runners: Assessment by Tissue Doppler Echocardiography, Speckle Tracking, and Cardiac Biomarkers. Journal of the American Society of Echocardiography, 2009. 22(7): p. 803-809."

231 "Koch, A., et al., Serum NT-proCNP concentrations are elevated in patients with chronic liver diseases and associated with complications and unfavorable prognosis of cirrhosis. Clinical Biochemistry, 2012. 45(6): p. 429-435."

232 "Komorita, Y., et al., The serum creatinine to cystatin C ratio predicts bone fracture in patients with type 2 diabetes: The Fukuoka Diabetes Registry. Diabetes Research and Clinical Practice, 2018. 146: p. 202-210."

233 "Kontny, F., et al., Pentraxin-3 vs C-reactive protein and other prognostic biomarkers in acute coronary syndrome: A substudy of the Platelet Inhibition and Patients Outcomes (PLATO) trial. Eur Heart J Acute Cardiovasc Care, 2020. 9(4): p. 313-322."

234 "Kos, F.T., et al., Evaluation of the renal function using cystatin C level in the patients receiving cisplatin-based chemotherapy. Ren Fail, 2013. 35(5): p. 705-10."

235 "Kovacs, R.J., et al., Cardiac Safety of TGF-β Receptor I Kinase Inhibitor LY2157299 Monohydrate in Cancer Patients in a First-in-Human Dose Study. Cardiovasc Toxicol, 2015. 15(4): p. 309-23."

236 "Koyner, J.L., Assessment and Diagnosis of Renal Dysfunction in the ICU. Chest, 2012. 141(6): p. 1584-1594."

237 "Krieter, D.H., et al., Clinical cross-over comparison of mid-dilution hemodiafiltration using a novel dialyzer concept and post-dilution hemodiafiltration. Kidney Int, 2005. 67(1): p. 349-56."

238 "Krieter, D.H., et al., Protein-bound uraemic toxin removal in haemodialysis and post-dilution haemodiafiltration. Nephrol Dial Transplant, 2010. 25(1): p. 212-8."

239 "Krievina, G., et al., Ectopic Adipose Tissue Storage in the Left and the Right Renal Sinus is Asymmetric and Associated With Serum Kidney Injury Molecule-1 and Fibroblast Growth Factor-21 Levels Increase. EBioMedicine, 2016. 13: p. 274-283."

240 "Krolewski, A.S. and J.V. Bonventre, High Risk of ESRD in Type 1 Diabetes: New Strategies Are Needed to Retard Progressive Renal Function Decline. Seminars in Nephrology, 2012. 32(5): p. 407-414."

241 "Kubota, N., et al., Impact of Fetuin-A on progression of calcific aortic valve stenosis - The COFRASA - GENERAC study. International Journal of Cardiology, 2018. 265: p. 52-57."

242 "Kuehne, L.K., et al., Cerebrospinal fluid neopterin is brain-derived and not associated with blood-CSF barrier dysfunction in non-inflammatory affective and schizophrenic spectrum disorders. Journal of Psychiatric Research, 2013. 47(10): p. 1417-1422."

243 "Kuk, C., et al., Mining the Ovarian Cancer Ascites Proteome for Potential Ovarian Cancer Biomarkers*S. Molecular & Cellular Proteomics, 2009. 8(4): p. 661-669."

244 "Kukla, A., et al., Cystatin C enhances glomerular filtration rate estimating equations in kidney transplant recipients. Am J Nephrol, 2014. 39(1): p. 59-65."

245 "Kumar, B., et al., Dynamic Alteration in the Vaginal Secretory Proteome across the Early and Mid-Trimesters of Pregnancy. Journal of Proteome Research, 2021. 20(2): p. 1190-1205."

246 "Kunutsor, S.K., et al., Associations of the fatty liver and hepatic steatosis indices with risk of cardiovascular disease: Interrelationship with age. Clinica Chimica Acta, 2017. 466: p. 54-60."

247 "Kupferman, M.E., et al., Molecular analysis of anoikis resistance in oral cavity squamous cell carcinoma. Oral Oncology, 2007. 43(5): p. 440-454."

248 "Ladero, J.M., et al., Serum cystatin C: a non-invasive marker of liver fibrosis or of current liver fibrogenesis in chronic hepatitis C? Annals of Hepatology, 2012. 11(5): p. 648-651."

249 "Lamattina, A.M., et al., Circulating Biomarkers From the Phase 1 Trial of Sirolimus and Autophagy Inhibition for Patients With Lymphangioleiomyomatosis. Chest, 2018. 154(5): p. 1070-1082."

250 "Lamb, E.J., et al., Diagnostic accuracy of cystatin C as a marker of kidney disease in patients with multiple myeloma: calculated glomerular filtration rate formulas are equally useful. Clin Chem, 2004. 50(10): p. 1848-51."

251 "Law, Y.M., et al., Randomized controlled trial of remote ischemic preconditioning in children having cardiac surgery. J Cardiothorac Surg, 2024. 19(1): p. 5."

252 "Le Bricon, T., et al., Changes in plasma cystatin C after renal transplantation and acute rejection in adults. Clin Chem, 1999. 45(12): p. 2243-9."

253 "Lee, B., et al., Effect of ulinastatin on postoperative renal function in patients undergoing robot-assisted laparoscopic partial nephrectomy: a randomized trial. Surg Endosc, 2017. 31(9): p. 3728-3736."

254 "Lee, E.-H., et al., Risk Factors of Postoperative Acute Kidney Injury in Patients Undergoing Esophageal Cancer Surgery. Journal of Cardiothoracic and Vascular Anesthesia, 2014. 28(4): p. 936-942."

255 "Levitsky, J., et al., Plasma protein biomarkers enhance the clinical prediction of kidney injury recovery in patients undergoing liver transplantation. Hepatology, 2014. 60(6): p. 2017-26."

256 "Lewis, E.J., et al., Pyridorin in type 2 diabetic nephropathy. J Am Soc Nephrol, 2012. 23(1): p. 131-6."

257 "Li, F., et al., Identification of urinary Gc-globulin as a novel biomarker for bladder cancer by two-dimensional fluorescent differential gel electrophoresis (2D-DIGE). Journal of Proteomics, 2012. 77: p. 225-236."

258 "Li, J., et al., Short-term and long-term safety and efficacy of tenofovir alafenamide, tenofovir disoproxil fumarate and entecavir treatment of acute-on-chronic liver failure associated with hepatitis B. BMC Infect Dis, 2021. 21(1): p. 567."

259 "Li, S., et al., A head-to-head comparison of homocysteine and cystatin C as pre-procedure predictors for contrast-induced nephropathy in patients undergoing coronary computed tomography angiography. Clinica Chimica Acta, 2015. 444: p. 86-91."

260 "Li, S., et al., Population Pharmacokinetics and Dosing Regimen Optimization of Linezolid in Cerebrospinal Fluid and Plasma of Post-operative Neurosurgical Patients. Journal of Pharmaceutical Sciences, 2023. 112(3): p. 884-892."

261 "Li, W., et al., Beneficial effects of high-dose atorvastatin pretreatment on renal function in patients with acute ST-segment elevation myocardial infarction undergoing emergency percutaneous coronary intervention. Cardiology, 2012. 122(3): p. 195-202."

262 "Liabeuf, S., et al., Plasma beta-2 microglobulin is associated with cardiovascular disease in uremic patients. Kidney International, 2012. 82(12): p. 1297-1303."

263 "Liang, J. and Z. Zhang, Predictors of in-hospital heart failure in patients with acute anterior wall ST-segment elevation myocardial infarction. International Journal of Cardiology, 2023. 375: p. 104-109."

264 "Liao, C.-C., et al., Comparative analysis of novel autoantibody isotypes against citrullinated-inter-alpha-trypsin inhibitor heavy chain 3 (ITIH3)542–556 peptide in serum from Taiwanese females with rheumatoid arthritis, primary Sj?gren's syndrome and secondary Sj?gren's syndrome in rheumatoid arthritis. Journal of Proteomics, 2016. 141: p. 1-11."

265 "Lima, C., et al., Role of proenkephalin in the diagnosis of severe and subclinical acute kidney injury during the perioperative period of liver transplantation. Practical Laboratory Medicine, 2022. 31: p. e00278."

266 "Lindholt, J.S., E.J. Erlandsen, and E.W. Henneberg, Cystatin C deficiency is associated with the progression of small abdominal aortic aneurysms. Br J Surg, 2001. 88(11): p. 1472-5."

267 "Linzbach, S., et al., Role of N-Terminal Pro-Brain Natriuretic Peptide and Cystatin C to Estimate Renal Function in Patients With and Without Heart Failure. The American Journal of Cardiology, 2009. 103(8): p. 1128-1133."

268 "Liu, X., et al., The Preventive Effect of Alprostadil on the Contrast-Induced Nephropathy of Coronary Heart Disease Treated by Percutaneous Coronary Intervention in Moderate and High-Risk Population Stratified by Mehran Score. Angiology, 2022. 73(1): p. 33-41."

269 "Liu, Z., et al., The Health Impact of MAFLD, a Novel Disease Cluster of NAFLD, Is Amplified by the Integrated Effect of Fatty Liver Disease–Related Genetic Variants. Clinical Gastroenterology and Hepatology, 2022. 20(4): p. e855-e875."

270 "Liu, Z.M., et al., Effect of whole soy and purified isoflavone daidzein on renal function--a 6-month randomized controlled trial in equol-producing postmenopausal women with prehypertension. Clin Biochem, 2014. 47(13-14): p. 1250-6."

271 "Lobetti, R., et al., NT-ProBNP and cardiac troponin I in virulent canine babesiosis. Veterinary Parasitology, 2012. 190(3): p. 333-339."

272 "Longenecker, C.T., et al., Rosuvastatin preserves renal function and lowers cystatin C in HIV-infected subjects on antiretroviral therapy: the SATURN-HIV trial. Clin Infect Dis, 2014. 59(8): p. 1148-56."

273 "López-Cuenca, á., et al., Beta-trace protein and cystatin c as predictors of major bleeding in non-ST-segment elevation acute coronary syndrome. Circ J, 2013. 77(8): p. 2088-96."

274 "Lu, J., et al., Serum NGAL Is Superior to Cystatin C in Predicting the Prognosis of Acute-on-Chronic Liver Failure. Annals of Hepatology, 2019. 18(1): p. 155-164."

275 "Lu, Y., et al., Urine AQP5 is a potential novel biomarker of diabetic nephropathy. Journal of Diabetes and its Complications, 2016. 30(5): p. 819-825."

276 "Lucas, G.M., et al., Glomerular filtration rate estimated using creatinine, cystatin C or both markers and the risk of clinical events in HIV-infected individuals. HIV Med, 2014. 15(2): p. 116-23."

277 "Luo, X., et al., A high-quality secretome of A549 cells aided the discovery of C4b-binding protein as a novel serum biomarker for non-small cell lung cancer. Journal of Proteomics, 2011. 74(4): p. 528-538."

278 "Lutsey, P.L., et al., The 25-hydroxyvitamin D3 C-3 epimer: Distribution, correlates, and reclassification of 25-hydroxyvitamin D status in the population-based Atherosclerosis Risk in Communities Study (ARIC). Clinica Chimica Acta, 2015. 442: p. 75-81."

279 "Lyu, M., et al., Do genetic polymorphisms of B-cell CLL/lymphoma 2 confer susceptibility to anti-tuberculous therapy-associated drug-induced liver injury? International Journal of Infectious Diseases, 2020. 91: p. 223-231."

280 "Ma, Y., Y. Chen, and I. Petersen, Expression and epigenetic regulation of cystatin B in lung cancer and colorectal cancer. Pathology - Research and Practice, 2017. 213(12): p. 1568-1574."

281 "Machado, J.D., et al., Combined creatinine-cystatin C CKD-EPI equation significantly underestimates measured glomerular filtration rate in people with type 2 diabetes mellitus. Clin Biochem, 2018. 53: p. 43-48."

282 "Maddens, B., et al., Chitinase-like Proteins are Candidate Biomarkers for Sepsis-induced Acute Kidney Injury*. Molecular & Cellular Proteomics, 2012. 11(6): p. M111.013094."

283 "Maiwall, R., et al., A randomised-controlled trial (TARGET-C) of high vs. low target mean arterial pressure in patients with cirrhosis and septic shock. J Hepatol, 2023. 79(2): p. 349-361."

284 "Malyszko, J., et al., Hepcidin – Potential biomarker of contrast-induced acute kidney injury in patients undergoing percutaneous coronary interventions. Advances in Medical Sciences, 2019. 64(2): p. 211-215."

285 "Mamoulakis, C., et al., Contrast-induced nephropathy in an animal model: Evaluation of novel biomarkers in blood and tissue samples. Toxicology Reports, 2019. 6: p. 395-400."

286 "Mannes, A.J., et al., Cystatin C as a cerebrospinal fluid biomarker for pain in humans. Pain, 2003. 102(3): p. 251-256."

287 "Manoli, I., et al., 1-13C-propionate breath testing as a surrogate endpoint to assess efficacy of liver-directed therapies in methylmalonic acidemia (MMA). Genetics in Medicine, 2021. 23(8): p. 1522-1533."

288 "Mansour, S.G., et al., Kidney Injury and Repair Biomarkers in Marathon Runners. American Journal of Kidney Diseases, 2017. 70(2): p. 252-261."

289 "Marcelino, P., et al., Is Urinary γ-Glutamyl Transpeptidase Superior to Urinary Neutrophil Gelatinase–Associated Lipocalin for Early Prediction of Acute Kidney Injury After Liver Transplantation? Transplantation Proceedings, 2014. 46(6): p. 1812-1818."

290 "Marcovecchio, M.L., et al., Adolescent Type 1 Diabetes Cardio-Renal Intervention Trial (AdDIT): urinary screening and baseline biochemical and cardiovascular assessments. Diabetes Care, 2014. 37(3): p. 805-13."

291 "Mares, J., et al., The assessment of beta amyloid, tau protein and cystatin C in the cerebrospinal fluid: laboratory markers of neurodegenerative diseases. Neurol Sci, 2009. 30(1): p. 1-7."

292 "Mariat, C., Diagnostic et suivi de la dysfonction chronique du greffon rénal : du DFG aux nouveaux biomarqueurs. Néphrologie & Thérapeutique, 2008. 4: p. S204-S207."

293 "Martin, A., et al., Abacavir does not affect circulating levels of inflammatory or coagulopathic biomarkers in suppressed HIV: a randomized clinical trial. Aids, 2010. 24(17): p. 2657-63."

294 "Masaki, H., et al., Bazedoxifene improves renal function and increases renal phosphate excretion in patients with postmenopausal osteoporosis. J Bone Miner Metab, 2020. 38(3): p. 405-411."

295 "Massion, P.P. and R.M. Caprioli, Proteomic Strategies for the Characterization and the Early Detection of Lung Cancer. Journal of Thoracic Oncology, 2006. 1(9): p. 1027-1039."

296 "Matsuda, Y., et al., Impact of Renal Dysfunction on Left Atrial Low-Voltage Areas in Patients With Atrial Fibrillation. Circ J, 2019. 83(5): p. 985-990."

297 "McDonald, J.S., et al., Bilateral Sustained Nephrograms After Parenteral Administration of Iodinated Contrast Material: A Potential Biomarker for Acute Kidney Injury, Dialysis, and Mortality. Mayo Clinic Proceedings, 2018. 93(7): p. 867-876."

298 "McEvoy, J.W., et al., Myocardial Injury Thresholds for 4?High-Sensitivity Troponin Assays in?U.S.?Adults. Journal of the American College of Cardiology, 2023. 81(20): p. 2028-2039."

299 "McManus, D., et al., Association of cystatin C with poor exercise capacity and heart rate recovery: data from the heart and soul study. Am J Kidney Dis, 2007. 49(3): p. 365-72."

300 "Mebus, S., et al., Non-invasive assessment of liver changes in Eisenmenger patients. International Journal of Cardiology, 2017. 249: p. 140-144."

301 "Mejía-Rodríguez, O., et al., Cardiovascular and renal effects of bromocriptine in diabetic patients with stage 4 chronic kidney disease. Biomed Res Int, 2013. 2013: p. 104059."

302 "Meng, Y., et al., Diagnosis and management of nonallergic rhinitis with eosinophilia syndrome using cystatin SN together with symptoms. World Allergy Organization Journal, 2020. 13(7): p. 100134."

303 "Merchant, M.L., et al., Plasma kininogen and kininogen fragments are biomarkers of progressive renal decline in type 1 diabetes. Kidney International, 2013. 83(6): p. 1177-1184."

304 "Metra, M., et al., Effect of serelaxin on cardiac, renal, and hepatic biomarkers in the Relaxin in Acute Heart Failure (RELAX-AHF) development program: correlation with outcomes. J Am Coll Cardiol, 2013. 61(2): p. 196-206."

305 "Meyerson, M., W.A. Franklin, and M.J. Kelley, Molecular classification and molecular genetics of human lung cancers. Seminars in Oncology, 2004. 31: p. 4-19."

306 "Miao, S., et al., Comparison of Different Hydration Strategies in Patients with Very Low-Risk Profiles of Contrast-Induced Nephropathy. Med Sci Monit, 2021. 27: p. e929115."

307 "Michael, I.P., et al., Biochemical and Enzymatic Characterization of Human Kallikrein 5 (hK5), a Novel Serine Protease Potentially Involved in Cancer Progression*. Journal of Biological Chemistry, 2005. 280(15): p. 14628-14635."

308 "Milas, O., et al., Pro-inflammatory cytokines are associated with podocyte damage and proximal tubular dysfunction in the early stage of diabetic kidney disease in type 2 diabetes mellitus patients. Journal of Diabetes and its Complications, 2020. 34(2): p. 107479."

309 "Miller, E.R., 3rd, et al., The effects of n-3 long-chain polyunsaturated fatty acid supplementation on biomarkers of kidney injury in adults with diabetes: results of the GO-FISH trial. Diabetes Care, 2013. 36(6): p. 1462-9."

310 "Mindikoglu, A.L. and S.C. Pappas, New Developments in Hepatorenal Syndrome. Clinical Gastroenterology and Hepatology, 2018. 16(2): p. 162-177.e1."

311 "Mindikoglu, A.L., et al., Estimation of Glomerular Filtration Rate in Patients With Cirrhosis by Using New and Conventional Filtration Markers and?Dimethylarginines. Clinical Gastroenterology and Hepatology, 2016. 14(4): p. 624-632.e2."

312 "MINJA, D.A., 胱抑素c和NTproBNP在保留左心室射血分数的COPD住院患者中的作用. 2019."

313 "Miraghajani, M., et al., Probiotic Soy Milk Consumption and Renal Function Among Type 2 Diabetic Patients with Nephropathy: a Randomized Controlled Clinical Trial. Probiotics Antimicrob Proteins, 2019. 11(1): p. 124-132."

314 "Miyaoka, D., et al., Denosumab Improves Glomerular Filtration Rate in Osteoporotic Patients With Normal Kidney Function by Lowering Serum Phosphorus. J Bone Miner Res, 2019. 34(11): p. 2028-2035."

315 "Mohammed, N.U.G., F.M. Khaleel, and F.I. Gorial, Cystatin D as a new diagnostic marker in rheumatoid arthritis. Gene Reports, 2021. 23: p. 101027."

316 "Mohebi, R., et al., Relation of High-Sensitivity Cardiac Troponin I and Obstructive Coronary Artery Disease in Patients Without Acute Myocardial Infarction. The American Journal of Cardiology, 2022. 173: p. 16-24."

317 "Mondesert, E., et al., Cystatin C for kidney function assessment in patients with facioscapulohumeral muscular dystrophy. Clinica Chimica Acta, 2023. 544: p. 117328."

318 "Montero-Calle, A., et al., Multiplexed Biosensing Diagnostic Platforms Detecting Autoantibodies to Tumor-Associated Antigens from Exosomes Released by CRC Cells and Tissue Samples Showed High Diagnostic Ability for Colorectal Cancer. Engineering, 2021. 7(10): p. 1393-1412."

319 "Moore, J.X., et al., Hemostasis biomarkers and risk of sepsis: the REGARDS cohort. Journal of Thrombosis and Haemostasis, 2016. 14(11): p. 2169-2176."

320 "Morales, D.M., et al., Alterations in Protein Regulators of Neurodevelopment in the Cerebrospinal Fluid of Infants with Posthemorrhagic Hydrocephalus of Prematurity*. Molecular & Cellular Proteomics, 2012. 11(6): p. M111.011973."

321 "Mordi, N.A., et al., Renal and Cardiovascular Effects of sodium-glucose cotransporter 2 (SGLT2) inhibition in combination with loop Diuretics in diabetic patients with Chronic Heart Failure (RECEDE-CHF): protocol for a randomised controlled double-blind cross-over trial. BMJ Open, 2017. 7(10): p. e018097."

322 "Morelli, M.C., et al., Position paper on liver and kidney diseases from the Italian Association for the Study of Liver (AISF), in collaboration with the Italian Society of Nephrology (SIN). Digestive and Liver Disease, 2021. 53: p. S49-S86."

323 "Mouton-Barbosa, E., et al., In-depth Exploration of Cerebrospinal Fluid by Combining Peptide Ligand Library Treatment and Label-free Protein Quantification*. Molecular & Cellular Proteomics, 2010. 9(5): p. 1006-1021."

324 "Mukhtar, A., et al., The safety of modern hydroxyethyl starch in living donor liver transplantation: a comparison with human albumin. Anesth Analg, 2009. 109(3): p. 924-30."

325 "Mukhtar, A., et al., The use of terlipressin during living donor liver transplantation: Effects on systemic and splanchnic hemodynamics and renal function. Crit Care Med, 2011. 39(6): p. 1329-34."

326 "Mullan, A., et al., Effects of a beverage rich in (poly)phenols on established and novel risk markers for vascular disease in medically uncomplicated overweight or obese subjects: A four week randomized placebo-controlled trial. Atherosclerosis, 2016. 246: p. 169-76."

327 "Muntner, P., et al., Overweight, Obesity, and Elevated Serum Cystatin C Levels in Adults in the United States. The American Journal of Medicine, 2008. 121(4): p. 341-348."

328 "Murai, T., et al., Association of epicardial adipose tissue with serum level of cystatin C in type 2 diabetes. PLoS One, 2017. 12(9): p. e0184723."

329 "Mutlu, M., et al., Effects of adenoid/tonsillectomy on inflammatory response in snoring children with witnessed apnoea. Clin Otolaryngol, 2014. 39(5): p. 266-71."

330 "Mychaleckyj, J.C., et al., Reversibility of fenofibrate therapy-induced renal function impairment in ACCORD type 2 diabetic participants. Diabetes Care, 2012. 35(5): p. 1008-14."

331 "Nagao, M., et al., Efficacy and safety of sitagliptin treatment in older adults with moderately controlled type 2 diabetes: the STREAM study. Sci Rep, 2023. 13(1): p. 134."

332 "Nassirpour, R., et al., MicroRNA biomarkers in clinical renal disease: from diabetic nephropathy renal transplantation and beyond. Food and Chemical Toxicology, 2016. 98: p. 73-88."

333 "Nazim, J., et al., Low-grade Albuminuria and Risk Factors of Atherosclerosis in Children with in Type 1 Diabetes. Exp Clin Endocrinol Diabetes, 2016. 124(1): p. 16-21."

334 "Nejat, M., et al., Urinary cystatin C is diagnostic of acute kidney injury and sepsis, and predicts mortality in the intensive care unit. Crit Care, 2010. 14(3): p. R85."

335 "Nergelius, G., et al., Renal dysfunction after total knee replacement is not aggravated by bone cement. Acta Anaesthesiol Scand, 1998. 42(8): p. 974-81."

336 "Nguyen, C.Q. and A.B. Peck, Unraveling the Pathophysiology of Sjogren Syndrome-Associated Dry Eye Disease. The Ocular Surface, 2009. 7(1): p. 11-27."

337 "Nguyen, H.-Q., et al., Label-free quantitative proteomic analysis of serum extracellular vesicles differentiating patients of alcoholic and nonalcoholic fatty liver diseases. Journal of Proteomics, 2021. 245: p. 104278."

338 "Nickerson, H.D. and S. Dutta, JDRF Perspective: Bridging the Gap—Translational Research to Prevent Progression of Diabetic Nephropathy. Seminars in Nephrology, 2012. 32(5): p. 512-516."

339 "Nie, F., et al., Serum klotho protein levels and their correlations with the progression of type 2 diabetes mellitus. Journal of Diabetes and its Complications, 2017. 31(3): p. 594-598."

340 "Nielsen, M.B., et al., P-NGAL Day 1 predicts early but not one year graft function following deceased donor kidney transplantation - The CONTEXT study. PLoS One, 2019. 14(2): p. e0212676."

341 "Nix, W.A., et al., Vitamin B status in patients with type 2 diabetes mellitus with and without incipient nephropathy. Diabetes Research and Clinical Practice, 2015. 107(1): p. 157-165."

342 "Norwitz, E.R., et al., Discriminatory proteomic biomarker analysis identifies free hemoglobin in the cerebrospinal fluid of women with severe preeclampsia. American Journal of Obstetrics and Gynecology, 2005. 193(3, Supplement): p. 957-964."

343 "Oberbauer, R., Biomarkers—A Potential Route for Improved Diagnosis and Management of Ongoing Renal Damage. Transplantation Proceedings, 2008. 40(10, Supplement): p. S44-S47."

344 "Oc, M.A., et al., Correlation of Cystatin-C and radionuclidic measurement method of glomerular filtration rate in patients with lung cancer receiving cisplatin treatment. Ren Fail, 2014. 36(7): p. 1043-50."

345 "Odden, M.C., et al., Risk factors for cardiovascular disease across the spectrum of older age: The Cardiovascular Health Study. Atherosclerosis, 2014. 237(1): p. 336-342."

346 "Oddoze, C., et al., Cystatin C is not more sensitive than creatinine for detecting early renal impairment in patients with diabetes. Am J Kidney Dis, 2001. 38(2): p. 310-6."

347 "Ogawa, Y., et al., Serum cystatin C in diabetic patients: Not only an indicator for renal dysfunction in patients with overt nephropathy but also a predictor for cardiovascular events in patients without nephropathy. Diabetes Research and Clinical Practice, 2008. 79(2): p. 357-361."

348 "Opotowsky, A.R., et al., Creatinine versus cystatin C to estimate glomerular filtration rate in adults with congenital heart disease: Results of the Boston Adult Congenital Heart Disease Biobank. American Heart Journal, 2019. 214: p. 142-155."

349 "Oraby, M.A., et al., Dapagliflozin attenuates early markers of diabetic nephropathy in fructose-streptozotocin-induced diabetes in rats. Biomedicine & Pharmacotherapy, 2019. 109: p. 910-920."

350 "Overath, J.M., et al., Short-term preconditioning enhances the therapeutic potential of adipose-derived stromal/stem cell-conditioned medium in cisplatin-induced acute kidney injury. Experimental Cell Research, 2016. 342(2): p. 175-183."

351 "Pacheco, J.G., et al., Breast cancer biomarker (HER2-ECD) detection using a molecularly imprinted electrochemical sensor. Sensors and Actuators B: Chemical, 2018. 273: p. 1008-1014."

352 "Padhy, M., et al., Serum neutrophil gelatinase associated lipocalin (NGAL) and cystatin C as early predictors of contrast-induced acute kidney injury in patients undergoing percutaneous coronary intervention. Clinica Chimica Acta, 2014. 435: p. 48-52."

353 "Palermo, C. and J.A. Joyce, Cysteine cathepsin proteases as pharmacological targets in cancer. Trends in Pharmacological Sciences, 2008. 29(1): p. 22-28."

354 "Pan, X., et al., High glucose–induced Smad3 linker phosphorylation and CCN2 expression are inhibited by dapagliflozin in a diabetic tubule epithelial cell model. Bioscience Reports, 2021. 41(6)."

355 "Papassotiriou, G.-P., et al., Neutrophil Gelatinase–Associated Lipocalin and Cystatin C Are Sensitive Markers of Renal Injury in Patients With Multiple Myeloma. Clinical Lymphoma Myeloma and Leukemia, 2016. 16(1): p. 29-35."

356 "Papassotiriou, I., et al., Cystatin C levels in patients with β-thalassemia during deferasirox treatment. Blood Cells, Molecules, and Diseases, 2010. 44(3): p. 152-155."

357 "Parcha, V., et al., Clinical, Demographic, and Imaging Correlates of Anemia in Heart Failure With Preserved Ejection Fraction (from the RELAX Trial). Am J Cardiol, 2020. 125(12): p. 1870-1878."

358 "Park, D.C., et al., Clusterin Interacts with Paclitaxel and Confer Paclitaxel Resistance in Ovarian Cancer. Neoplasia, 2008. 10(9): p. 964-IN7."

359 "Park, M., et al., Associations of N-terminal pro–B-type natriuretic peptide with kidney function decline in persons without clinical heart failure in the Heart and Soul Study. American Heart Journal, 2014. 168(6): p. 931-939.e2."

360 "Park, M., et al., Associations of tumor necrosis factor alpha receptor type 1 with kidney function decline, cardiovascular events, and mortality risk in persons with coronary artery disease: Data from the Heart and Soul Study. Atherosclerosis, 2017. 263: p. 68-73."

361 "Patel, D., et al., Effect of cystatin C levels on angiographic atherosclerosis progression and events among postmenopausal women with angiographically decompensated coronary artery disease (from the Women's Angiographic Vitamin and Estrogen [WAVE] study). Am J Cardiol, 2013. 111(12): p. 1681-7."

362 "Patyna, S., et al., Blood ceramides as novel markers for renal impairment in systemic lupus erythematosus. Prostaglandins & Other Lipid Mediators, 2019. 144: p. 106348."

363 "Pavkov, M.E., et al., Comparison of Serum Cystatin C, Serum Creatinine, Measured GFR, and Estimated GFR to Assess the Risk of Kidney Failure in American Indians With Diabetic Nephropathy. American Journal of Kidney Diseases, 2013. 62(1): p. 33-41."

364 "Pavkov, M.E., et al., Elevation of circulating TNF receptors 1 and 2 increases the risk of end-stage renal disease in American Indians with type 2 diabetes. Kidney International, 2015. 87(4): p. 812-819."

365 "Pavkov, M.E., et al., Kidney Disease in Diabetes, in Diabetes in America, C.C. Cowie, et al., Editors. 2018, National Institute of Diabetes and Digestive and Kidney Diseases (US): Bethesda (MD)."

366 "Pedersen, K.R., et al., Failure of remote ischemic preconditioning to reduce the risk of postoperative acute kidney injury in children undergoing operation for complex congenital heart disease: a randomized single-center study. J Thorac Cardiovasc Surg, 2012. 143(3): p. 576-83."

367 "Peralta, C.A., et al., Implementation of a pragmatic randomized trial of screening for chronic kidney disease to improve care among non-diabetic hypertensive veterans. BMC Nephrol, 2017. 18(1): p. 132."

368 "Peralta, C.A., et al., Screening for CKD To Improve Processes of Care among Nondiabetic Veterans with Hypertension: A Pragmatic Cluster-Randomized Trial. Clin J Am Soc Nephrol, 2020. 15(2): p. 174-181."

369 "Petrica, L., et al., Glycated peptides are associated with the variability of endothelial dysfunction in the cerebral vessels and the kidney in type 2 diabetes mellitus patients: a cross-sectional study. Journal of Diabetes and its Complications, 2015. 29(2): p. 230-237."

370 "Petrica, L., et al., Nephro- and neuroprotective effects of rosiglitazone versus glimepiride in normoalbuminuric patients with type 2 diabetes mellitus: a randomized controlled trial. Wien Klin Wochenschr, 2009. 121(23-24): p. 765-75."

371 "Petrica, L., et al., Podocyturia parallels proximal tubule dysfunction in type 2 diabetes mellitus patients independently of albuminuria and renal function decline: A cross-sectional study. Journal of Diabetes and its Complications, 2017. 31(9): p. 1444-1450."

372 "Petty, R.D., et al., Tumor transcriptome reveals the predictive and prognostic impact of lysosomal protease inhibitors in non-small-cell lung cancer. J Clin Oncol, 2006. 24(11): p. 1729-44."

373 "Pevzner, I.B., et al., The effects of antibiotic therapy on neonatal sepsis-associated acute kidney injury. Life Sciences, 2024. 338: p. 122359."

374 "Pinto de Carvalho, L., et al., Renal function and anaemia in acute myocardial infarction. International Journal of Cardiology, 2013. 168(2): p. 1397-1401."

375 "Pipili, C., et al., Prediction of the renal replacement therapy requirement in mechanically ventilated critically ill patients by combining biomarkers for glomerular filtration and tubular damage. Journal of Critical Care, 2014. 29(4): p. 692.e7-692.e13."

376 "Planque, C., et al., Identification of Five Candidate Lung Cancer Biomarkers by Proteomics Analysis of Conditioned Media of Four Lung Cancer Cell Lines*. Molecular & Cellular Proteomics, 2009. 8(12): p. 2746-2758."

377 "Pode Shakked, N., et al., Early prediction of COVID-19-associated acute kidney injury: Are serum NGAL and serum Cystatin C levels better than serum creatinine? Clinical Biochemistry, 2022. 102: p. 1-8."

378 "Poersch, A., et al., A proteomic signature of ovarian cancer tumor fluid identified by highthroughput and verified by targeted proteomics. Journal of Proteomics, 2016. 145: p. 226-236."

379 "Portelius, E., et al., Identification of novel N-terminal fragments of amyloid precursor protein in cerebrospinal fluid. Experimental Neurology, 2010. 223(2): p. 351-358."

380 "Potok, O.A., et al., Estimated GFR Accuracy When Cystatin C– and Creatinine-Based Estimates Are Discrepant in Older Adults. Kidney Medicine, 2023. 5(5): p. 100628."

381 "Pottel, H., E. Schaeffner, and N. Ebert, Evaluating the diagnostic value of rescaled β-trace protein in combination with serum creatinine and serum cystatin C in older adults. Clinica Chimica Acta, 2018. 480: p. 206-213."

382 "Pottel, H., et al., The diagnostic value of rescaled renal biomarkers serum creatinine and serum cystatin C and their relation with measured glomerular filtration rate. Clinica Chimica Acta, 2017. 471: p. 164-170."

383 "Prabhu, A., et al., Neutrophil Gelatinase Associated Lipocalin as a Biomarker for Acute Kidney Injury in Patients Undergoing Coronary Artery Bypass Grafting with Cardiopulmonary Bypass. Annals of Vascular Surgery, 2010. 24(4): p. 525-531."

384 "PramodKumar, T.A., et al., Role of cystatin C in the detection of sight-threatening diabetic retinopathy in Asian Indians with type 2 diabetes. Journal of Diabetes and its Complications, 2023. 37(8): p. 108545."

385 "Prayle, A.P., et al., The pharmacokinetics and toxicity of morning vs. evening tobramycin dosing for pulmonary exacerbations of cystic fibrosis: A randomised comparison. J Cyst Fibros, 2016. 15(4): p. 510-7."

386 "Przybylowski, P., et al., Liver Fatty-Acid–Binding Protein in Heart and Kidney Allograft Recipients in Relation to Kidney Function. Transplantation Proceedings, 2011. 43(8): p. 3064-3067."

387 "Puckett, J.R., et al., Low Versus Standard Urine Output Targets in Patients Undergoing Major Abdominal Surgery: A Randomized Noninferiority Trial. Ann Surg, 2017. 265(5): p. 874-881."

388 "Puurunen, M.K., et al., Biomarkers for the prediction of venous thromboembolism in the community. Thrombosis Research, 2016. 145: p. 34-39."

389 "Qasim, A.N., et al., Cardiovascular risk factors and mitral annular calcification in type 2 diabetes. Atherosclerosis, 2013. 226(2): p. 419-424."

390 "Quintavalle, C., et al., Impact of a high loading dose of atorvastatin on contrast-induced acute kidney injury. Circulation, 2012. 126(25): p. 3008-16."

391 "Ramakrishnudu, A., et al., Remote ischemic preconditioning for prevention of contrast-medium-induced nephropathy: (RenoProtection-trial). Journal of Indian College of Cardiology, 2015. 5(4): p. 297-304."

392 "Ramírez-Boo, M., et al., Characterization of the glycated human cerebrospinal fluid proteome. Journal of Proteomics, 2012. 75(15): p. 4766-4782."

393 "Ramos-Santos, K., et al., Cystatin C is a marker for acute kidney injury, but not for mortality among COVID-19 patients in Mexico. The Brazilian Journal of Infectious Diseases, 2022. 26(3): p. 102365."

394 "Ran, L., et al., Ampelopsis grossedentata supplementation effectively ameliorates the glycemic control in patients with type 2 diabetes mellitus. Eur J Clin Nutr, 2019. 73(5): p. 776-782."

395 "Rashbaum, B., et al., Darunavir/cobicistat/emtricitabine/tenofovir alafenamide in treatment-na?ve patients with HIV-1: subgroup analyses of the phase 3 AMBER study. HIV Res Clin Pract, 2019. 20(1): p. 24-33."

396 "Rasmussen, T.A., et al., Comparison of bone and renal effects in HIV-infected adults switching to abacavir or tenofovir based therapy in a randomized trial. PLoS One, 2012. 7(3): p. e32445."

397 "R?uber, S., et al., Cerebrospinal fluid proteomics indicates immune dysregulation and neuronal dysfunction in antibody associated autoimmune encephalitis. Journal of Autoimmunity, 2023. 135: p. 102985."

398 "Reis, J.P., et al., Parathyroid hormone is associated with incident diabetes in white, but not black adults: The Atherosclerosis Risk in Communities (ARIC) Study. Diabetes & Metabolism, 2016. 42(3): p. 162-169."

399 "Reutens, A.T., et al., A physician-initiated double-blind, randomised, placebo-controlled, phase 2 study evaluating the efficacy and safety of inhibition of NADPH oxidase with the first-in-class Nox-1/4 inhibitor, GKT137831, in adults with type 1 diabetes and persistently elevated urinary albumin excretion: Protocol and statistical considerations. Contemporary Clinical Trials, 2020. 90: p. 105892."

400 "Rigalleau, V., et al., The combination of cystatin C and serum creatinine improves the monitoring of kidney function in patients with diabetes and chronic kidney disease. Clin Chem, 2007. 53(11): p. 1988-9."

401 "Robert, A.M., et al., The evaluation of creatinine clearance, estimated glomerular filtration rate and serum creatinine in predicting contrast-induced acute kidney injury among patients undergoing percutaneous coronary intervention. Cardiovascular Revascularization Medicine, 2012. 13(1): p. 3-10."

402 "Roberts, D.M., et al., Changes in the concentrations of creatinine, cystatin C and NGAL in patients with acute paraquat self-poisoning. Toxicology Letters, 2011. 202(1): p. 69-74."

403 "Robinson, J.L., et al., A Systematic Investigation of the Malignant Functions and Diagnostic Potential of the Cancer Secretome. Cell Reports, 2019. 26(10): p. 2622-2635.e5."

404 "Roderburg, C., et al., Serum concentrations of A Proliferation-Inducing Ligand (APRIL) are elevated in sepsis and predict mortality in critically ill patients. Journal of Critical Care, 2013. 28(5): p. 882.e1-882.e11."

405 "Rohatgi, A., et al., The association between peptidoglycan recognition protein-1 and coronary and peripheral atherosclerosis: Observations from the Dallas Heart Study. Atherosclerosis, 2009. 203(2): p. 569-575."

406 "Rosner, M.H. and W.K. Bolton, Renal Function Testing. American Journal of Kidney Diseases, 2006. 47(1): p. 174-183."

407 "Rothberg, A.E., L.N. McEwen, and W.H. Herman, Severe obesity and the impact of medical weight loss on estimated glomerular filtration rate. PLoS One, 2020. 15(2): p. e0228984."

408 "Ruan, W., et al., The Multidisciplinary Pediatric Liver Transplant. Current Problems in Surgery, 2023. 60(11): p. 101377."

409 "Rubio-Gracia, J., et al., Prognostic value of malnutrition in patients with acute heart failure and its influence on the interpretation of markers of systemic venous congestion. Medicina Clínica (English Edition), 2021. 157(8): p. 371-379."

410 "Rubio-Gracia, J., et al., Prognostic value of multimodal assessment of congestion in acute heart failure. Revista Clínica Espa?ola (English Edition), 2021. 221(4): p. 198-206."

411 "Rüetschi, U., et al., Identification of CSF biomarkers for frontotemporal dementia using SELDI-TOF. Experimental Neurology, 2005. 196(2): p. 273-281."

412 "Ruiz-Argüelles, A., et al., Glomerular Filtration Rate in Patients with Multiple Sclerosis Undergoing Stem Cell Transplantation and Treated with Cyclophosphamide. Lab Med, 2019. 50(1): p. 42-46."

413 "Rutter, M.K., et al., Protection Against Nephropathy in Diabetes with Atorvastatin (PANDA): a randomized double-blind placebo-controlled trial of high- vs. low-dose atorvastatin(1). Diabet Med, 2011. 28(1): p. 100-8."

414 "Ryder, J.R., et al., Effect of surgical versus medical therapy on estimated cardiovascular event risk among adolescents with type 2 diabetes and severe obesity. Surgery for Obesity and Related Diseases, 2021. 17(1): p. 23-33."

415 "Salcedo, E.C., et al., Global Protease Activity Profiling Identifies HER2-Driven Proteolysis in Breast Cancer. ACS Chemical Biology, 2021. 16(4): p. 712-723."

416 "Santos, C., et al., The Value of Tubular Enzymes for Early Detection of Acute Kidney Injury After Liver Transplantation: An Observational Study. Transplantation Proceedings, 2010. 42(9): p. 3639-3643."

417 "Sarangam, M.L., et al., Intestinal Injury Biomarkers Predict Mortality in Pediatric Severe Malaria. mBio, 2022. 13(5)."

418 "Satirapoj, B., et al., Urinary biomarkers of tubular injury to predict renal progression and end stage renal disease in type 2 diabetes mellitus with advanced nephropathy: A prospective cohort study. Journal of Diabetes and its Complications, 2019. 33(9): p. 675-681."

419 "Scherr, J., et al., 72-h kinetics of high-sensitive troponin T and inflammatory markers after marathon. Med Sci Sports Exerc, 2011. 43(10): p. 1819-27."

420 "Schmitt, A., et al., A universal formula based on cystatin C to perform individual dosing of carboplatin in normal weight, underweight, and obese patients. Clin Cancer Res, 2009. 15(10): p. 3633-9."

421 "Sch?n, M., et al., Analysis of type 2 diabetes heterogeneity with a tree-like representation: insights from the prospective German Diabetes Study and the LURIC cohort. The Lancet Diabetes & Endocrinology, 2024. 12(2): p. 119-131."

422 "Schreuder, M.M., et al., Sex-specific temporal evolution of circulating biomarkers in patients with chronic heart failure with reduced ejection fraction. International Journal of Cardiology, 2021. 334: p. 126-134."

423 "Schroder, J., et al., Protein biomarkers and coronary microvascular dilatation assessed by rubidium-82 PET in women with angina pectoris and no obstructive coronary artery disease. Atherosclerosis, 2018. 275: p. 319-327."

424 "Secemsky, E.A., et al., Novel Biomarkers of Cardiac Stress, Cardiovascular Dysfunction, and Outcomes in HIV-Infected Individuals. JACC: Heart Failure, 2015. 3(8): p. 591-599."

425 "Seele, J., et al., Cisterno-lumbar gradient of complement fractions in geriatric patients with suspected normal pressure hydrocephalus. Clinica Chimica Acta, 2018. 486: p. 1-7."

426 "Segar, J.L., et al., Fluid management, electrolytes imbalance and renal management in neonates with neonatal encephalopathy treated with hypothermia. Seminars in Fetal and Neonatal Medicine, 2021. 26(4): p. 101261."

427 "Sezai, A., et al., Cross-Over Trial of Febuxostat and Topiroxostat for Hyperuricemia With Cardiovascular Disease (TROFEO Trial). Circ J, 2017. 81(11): p. 1707-1712."

428 "Sezai, A., et al., Long-Term Comparison of Ethyl Icosapentate vs. Omega-3-Acid Ethyl in Patients With Cardiovascular Disease and Hypertriglyceridemia (DEFAT Trial). Circ J, 2019. 83(6): p. 1368-1376."

429 "Shaaban, A.A., et al., Soluble and membranous endothelial protein C receptor in systemic lupus erythematosus patients: Relation to nephritis. The Egyptian Rheumatologist, 2019. 41(1): p. 25-30."

430 "Shafie, I.N.F., et al., A protocol for the management of canine cerebrospinal fluid for the proteomic assessment of putative biomarkers. The Veterinary Journal, 2013. 197(3): p. 836-841."

431 "Shah, R., et al., Serum Fractalkine (CX3CL1) and Cardiovascular Outcomes and Diabetes: Findings From the Chronic Renal Insufficiency Cohort (CRIC) Study. American Journal of Kidney Diseases, 2015. 66(2): p. 266-273."

432 "Shaikh, S., D.K. Yadav, and R. Rawal, Saliva based non invasive screening of Oral Submucous Fibrosis using ATR-FTIR spectroscopy. Journal of Pharmaceutical and Biomedical Analysis, 2021. 203: p. 114202."

433 "Shema-Didi, L., et al., Prevention of contrast-induced nephropathy with single bolus erythropoietin in patients with diabetic kidney disease: A randomized controlled trial. Nephrology (Carlton), 2016. 21(4): p. 295-300."

434 "Shen, G., et al., Increased Cystatin C Level in ST-Elevation Myocardial Infarction Predisposes the Prognosis of Angioplasty. The American Journal of the Medical Sciences, 2018. 355(6): p. 530-536."

435 "Shima, A., et al., Beraprost Sodium Protects Against Diabetic Nephropathy in Patients with Arteriosclerosis Obliterans: A Prospective, Randomized, Open-label Study. J Nippon Med Sch, 2015. 82(2): p. 84-91."

436 "Shin, Y.J., et al., Age-related differences in kidney injury biomarkers induced by cisplatin. Environmental Toxicology and Pharmacology, 2014. 37(3): p. 1028-1039."

437 "Shlipak, M.G., et al., Biomarkers to Predict Recurrent Cardiovascular Disease: The Heart and Soul Study. The American Journal of Medicine, 2008. 121(1): p. 50-57."

438 "Siegbahn, A., et al., Multiplex protein screening of biomarkers associated with major bleeding in patients with atrial fibrillation treated with oral anticoagulation. Journal of Thrombosis and Haemostasis, 2021. 19(11): p. 2726-2737."

439 "Silva Marques, J., et al., Biomarkers of functional class in systolic heart failure: The relevance of copeptin. Revista Portuguesa de Cardiologia (English Edition), 2012. 31(11): p. 701-710."

440 "Silva, W.A.D., et al., Restrictive versus Liberal Fluid Therapy for Post-Cesarean Acute Kidney Injury in Severe Preeclampsia: a Pilot Randomized Clinical Trial. Clinics (Sao Paulo), 2020. 75: p. e1797."

441 "Simons, N., et al., Kidney and vascular function in adult patients with hereditary fructose intolerance. Molecular Genetics and Metabolism Reports, 2020. 23: p. 100600."

442 "Simonsen, A.H., et al., Identification of a novel panel of cerebrospinal fluid biomarkers for Alzheimer's disease. Neurobiology of Aging, 2008. 29(7): p. 961-968."

443 "Simonsen, A.H., et al., Pre-analytical factors influencing the stability of cerebrospinal fluid proteins. Journal of Neuroscience Methods, 2013. 215(2): p. 234-240."

444 "Sirota, J.C., et al., Urine IL-18, NGAL, IL-8 and serum IL-8 are biomarkers of acute kidney injury following liver transplantation. BMC Nephrol, 2013. 14: p. 17."

445 "Sj?din, M.O.D., J. Bergquist, and M. Wetterhall, Mining ventricular cerebrospinal fluid from patients with traumatic brain injury using hexapeptide ligand libraries to search for trauma biomarkers. Journal of Chromatography B, 2010. 878(22): p. 2003-2012."

446 "Sj?wall, C., et al., Soluble urokinase plasminogen activator receptor levels are associated with severity of fibrosis in nonalcoholic fatty liver disease. Translational Research, 2015. 165(6): p. 658-666."

447 "Skupien, J., et al., The early decline in renal function in patients with type 1 diabetes and proteinuria predicts the risk of end-stage renal disease. Kidney International, 2012. 82(5): p. 589-597."

448 "Solomon, R.J., et al., Contrast-induced nephropathy and long-term adverse events: cause and effect? Clin J Am Soc Nephrol, 2009. 4(7): p. 1162-9."

449 "Song, S., B.M. Song, and H.-Y. Park, Associations of Serum Folate and Homocysteine Concentrations with All-Cause, Cardiovascular Disease, and Cancer Mortality in Men and Women in Korea: the Cardiovascular Disease Association Study. The Journal of Nutrition, 2023. 153(3): p. 760-770."

450 "Sourij, H., et al., Arginine bioavailability ratios are associated with cardiovascular mortality in patients referred to coronary angiography. Atherosclerosis, 2011. 218(1): p. 220-225."

451 "Stabuc, B., et al., Improved prediction of decreased creatinine clearance by serum cystatin C: use in cancer patients before and during chemotherapy. Clin Chem, 2000. 46(2): p. 193-7."

452 "Stack, A.G., et al., Effect of Intensive Urate Lowering With Combined Verinurad and Febuxostat on Albuminuria in Patients With Type 2 Diabetes: A Randomized Trial. Am J Kidney Dis, 2021. 77(4): p. 481-489."

453 "Stokfisz, K., et al., Remote Ischemic Preconditioning and Contrast-Induced Acute Kidney Injury in Patients Undergoing Elective Percutaneous Coronary Intervention: A Randomized Clinical Trial. Current Therapeutic Research, 2020. 93: p. 100599."

454 "Su, J.Z., et al., [Association between high sensitivity C-reactive protein and contrast induced acute kidney injury in patients with acute coronary syndrome undergoing percutaneous coronary intervention: impact of atorvastatin]. Zhonghua Xin Xue Guan Bing Za Zhi, 2011. 39(9): p. 807-11."

455 "Sun, H., et al., A translational study of Galectin-3 as an early biomarker and potential therapeutic target for ischemic-reperfusion induced acute kidney injury. Journal of Critical Care, 2021. 65: p. 192-199."

456 "Sun, Y., et al., Systematic comparison of exosomal proteomes from human saliva and serum for the detection of lung cancer. Analytica Chimica Acta, 2017. 982: p. 84-95."

457 "Suo, X.Q., et al., [Effect of probucol on preventing contrast-induced nephropathy in patients undergoing percutaneous coronary intervention]. Zhonghua Yi Xue Za Zhi, 2017. 97(41): p. 3234-3238."

458 "Suthahar, N., et al., Heart failure and inflammation-related biomarkers as predictors of new-onset diabetes in the general population. International Journal of Cardiology, 2018. 250: p. 188-194."

459 "Tahmasebi, H., et al., Pediatric reference intervals for clinical chemistry assays on Siemens ADVIA XPT/1800 and Dimension EXL in the CALIPER cohort of healthy children and adolescents. Clinica Chimica Acta, 2019. 490: p. 88-97."

460 "Takir, M., et al., Cystatin-C and TGF-β levels in patients with diabetic nephropathy. Nefrología (English Edition), 2016. 36(6): p. 653-659."

461 "Tan, D., et al., Value of urine IL-8, NGAL and KIM-1 for the early diagnosis of acute kidney injury in patients with ureteroscopic lithotripsy related urosepsis. Chinese Journal of Traumatology, 2022. 25(1): p. 27-31."

462 "Tan, L., et al., Early acute kidney injury after liver transplantation in patients with normal preoperative renal function. Clinics and Research in Hepatology and Gastroenterology, 2019. 43(4): p. 475-482."

463 "Tan, S.M.Q., et al., Tocotrienol-Rich Vitamin E from Palm Oil (Tocovid) and Its Effects in Diabetes and Diabetic Nephropathy: A Pilot Phase II Clinical Trial. Nutrients, 2018. 10(9)."

464 "Tang, Y., et al., Aberrant cytokine expression in COVID-19 patients: Associations between cytokines and disease severity. Cytokine, 2021. 143: p. 155523."

465 "Tarney, C.M., et al., Biomarker panel for early detection of endometrial cancer in the Prostate, Lung, Colorectal, and Ovarian cancer screening trial. American Journal of Obstetrics and Gynecology, 2019. 221(5): p. 472.e1-472.e10."

466 "Tato-Costa, J., et al., Therapy-Induced Cellular Senescence Induces Epithelial-to-Mesenchymal Transition and Increases Invasiveness in Rectal Cancer. Clinical Colorectal Cancer, 2016. 15(2): p. 170-178.e3."

467 "Techatanawat, S., et al., Salivary and serum cystatin SA levels in patients with type 2 diabetes mellitus or diabetic nephropathy. Archives of Oral Biology, 2019. 104: p. 67-75."

468 "Terpos, E., et al., Cystatin-C is an independent prognostic factor for survival in multiple myeloma and is reduced by bortezomib administration. Haematologica, 2009. 94(3): p. 372-9."

469 "Thela, L., et al., Blood and cerebrospinal fluid biomarker changes in patients with HIV-associated neurocognitive impairment treated with lithium: analysis from a randomised placebo-controlled trial. J Neurovirol, 2023. 29(2): p. 156-166."

470 "Tlemsani, C., et al., Relationship between the creatinine/cystatin C ratio and muscle mass measured by CT-scan in cancer patients. Clinical Nutrition ESPEN, 2022. 51: p. 412-418."

471 "Tomi?, M., et al., Plasma homocysteine is associated with nonproliferative retinopathy in patients with type 2 diabetes without renal disease. Diabetes & Metabolic Syndrome: Clinical Research & Reviews, 2022. 16(1): p. 102355."

472 "Tommerdahl, K.L., et al., Results from the Effects of MEtformin on cardiovasculaR function in AdoLescents with type 1 Diabetes (EMERALD) study: A brief report of kidney and inflammatory outcomes. Diabetes Obes Metab, 2021. 23(3): p. 844-849."

473 "Toribio, M., et al., Assessing statin effects on cardiovascular pathways in HIV using a novel proteomics approach: Analysis of data from INTREPID, a randomized controlled trial. EBioMedicine, 2018. 35: p. 58-66."

474 "Tran, N.K., et al., Artificial intelligence and machine learning for predicting acute kidney injury in severely burned patients: A proof of concept. Burns, 2019. 45(6): p. 1350-1358."

475 "Tshomba, Y., et al., Comparison of renal perfusion solutions during thoracoabdominal aortic aneurysm repair. Journal of Vascular Surgery, 2014. 59(3): p. 623-633."

476 "Tsuchimoto, A., et al., Urinary neutrophil gelatinase-associated lipocalin: a useful biomarker for tacrolimus-induced acute kidney injury in liver transplant patients. PLoS One, 2014. 9(10): p. e110527."

477 "Tsuda, A., et al., Poor glycemic control is a major factor in the overestimation of glomerular filtration rate in diabetic patients. Diabetes Care, 2014. 37(3): p. 596-603."

478 "Tuttle, K.R., et al., Body weight and eGFR during dulaglutide treatment in type 2 diabetes and moderate-to-severe chronic kidney disease (AWARD-7). Diabetes Obes Metab, 2019. 21(6): p. 1493-1497."

479 "Tuttle, K.R., et al., Dulaglutide versus insulin glargine in patients with type 2 diabetes and moderate-to-severe chronic kidney disease (AWARD-7): a multicentre, open-label, randomised trial. Lancet Diabetes Endocrinol, 2018. 6(8): p. 605-617."

480 "Ulmann, G., et al., Creatinine-to-cystatin C ratio and bioelectrical impedance analysis for the assessement of low lean body mass in cancer patients: Comparison to L3–computed tomography scan. Nutrition, 2021. 81: p. 110895."

481 "Urso, E., et al., Quantification of thymosin β4 in human cerebrospinal fluid using matrix-assisted laser desorption/ionization time-of-flight mass spectrometry. Analytical Biochemistry, 2010. 402(1): p. 13-19."

482 "Uruska, A., et al., Does serum cystatin C level reflect insulin resistance in patients with type 1 diabetes? Clin Biochem, 2014. 47(13-14): p. 1235-8."

483 "Vaduganathan, M., et al., Relation of Serum and Urine Renal Biomarkers to Cardiovascular Risk in Patients with Type 2 Diabetes Mellitus and Recent Acute Coronary Syndromes (From the EXAMINE Trial). Am J Cardiol, 2019. 123(3): p. 382-391."

484 "Vaidya, V.S., et al., Regression of microalbuminuria in type 1 diabetes is associated with lower levels of urinary tubular injury biomarkers, kidney injury molecule-1, and N-acetyl-β-D-glucosaminidase. Kidney International, 2011. 79(4): p. 464-470."

485 "van den Berg, E.H., et al., Cholesterol efflux capacity is impaired in subjects with an elevated Fatty Liver Index, a proxy of non-alcoholic fatty liver disease. Atherosclerosis, 2018. 277: p. 21-27."

486 "van den Berg, E.H., et al., Serum paraoxonase 1 activity is paradoxically maintained in nonalcoholic fatty liver disease despite low HDL cholesterol[S]. Journal of Lipid Research, 2019. 60(1): p. 168-175."

487 "van Hage, M., et al., An update on the prevalence and diagnosis of cat and dog allergy – Emphasizing the role of molecular allergy diagnostics. Molecular Immunology, 2023. 157: p. 1-7."

488 "van Ruiten, C.C., et al., Effect of exenatide twice daily and dapagliflozin, alone and in combination, on markers of kidney function in obese patients with type 2 diabetes: A prespecified secondary analysis of a randomized controlled clinical trial. Diabetes Obes Metab, 2021. 23(8): p. 1851-1858."

489 "van Westing, A.C., et al., Plasma fatty acids and kidney function decline in post-myocardial infarction patients of the Alpha Omega Cohort. Nutrition, Metabolism and Cardiovascular Diseases, 2021. 31(5): p. 1467-1476."

490 "Verma, R., et al., Hyperbaric oxygen therapy (HBOT) suppresses biomarkers of cell stress and kidney injury in diabetic mice. Cell Stress and Chaperones, 2015. 20(3): p. 495-505."

491 "Vijay, S., et al., Utility of urinary biomarkers as a diagnostic tool for early diabetic nephropathy in patients with type 2 diabetes mellitus. Diabetes & Metabolic Syndrome: Clinical Research & Reviews, 2018. 12(5): p. 649-652."

492 "Vílchez, J.A., et al., β-Trace Protein and Prognosis in Patients With Atrial Fibrillation Receiving Anticoagulation Treatment. Chest, 2013. 144(5): p. 1564-1570."

493 "Villela-Torres, M.D.L.L., et al., Copeptin Plasma Levels are Associated with Decline of Renal Function in Patients with Type 2 Diabetes Mellitus. Archives of Medical Research, 2018. 49(1): p. 36-43."

494 "Vrouenraets, S.M., et al., A comparison of measured and estimated glomerular filtration rate in successfully treated HIV-patients with preserved renal function. Clin Nephrol, 2012. 77(4): p. 311-20."

495 "Wallentin, L., et al., Angiotensin-converting enzyme 2 (ACE2) levels in relation to risk factors for COVID-19 in two large cohorts of patients with atrial fibrillation. Eur Heart J, 2020. 41(41): p. 4037-4046."

496 "Wallentin, L., et al., GDF-15 for prognostication of cardiovascular and cancer morbidity and mortality in men. PLoS One, 2013. 8(12): p. e78797."

497 "Wang, D., et al., L1 cell adhesion molecule may be a protective molecule for atrial fibrillation in patients with valvular heart disease. Heliyon, 2023. 9(6): p. e16831."

498 "Wang, K.X., et al., [Effect of electroacupuncture of ""Biao-Ben"" acupoints on renal function and hemorheology and eNOS level in patients with early diabetic nephropathy]. Zhen Ci Yan Jiu, 2022. 47(1): p. 46-52."

499 "Wang, M.R., et al., [Effect of Shenqi Dihuang decoction on inflammatory factor, renal function and microcirculation in patients with early diabetic nephropathy]. Zhongguo Zhong Yao Za Zhi, 2018. 43(6): p. 1276-1281."

500 "Wang, X., et al., [A clinical research on renal protective effect of Xuebijing injection in patients with sepsis]. Zhonghua Wei Zhong Bing Ji Jiu Yi Xue, 2015. 27(5): p. 371-4."

501 "Wang, Y., et al., A Study on Correlation between Contrast-Enhanced Ultrasound Parameters and Pathological Features of Diabetic Nephropathy. Ultrasound in Medicine & Biology, 2022. 48(2): p. 228-236."

502 "Wang, Z., et al., A multiplex protein panel assay for severity prediction and outcome prognosis in patients with COVID-19: An observational multi-cohort study. eClinicalMedicine, 2022. 49: p. 101495."

503 "Wang, Z.L., M. Liu, and Y.Q. Zhang, [The prevention of denhong injection on contrast-induced renal impairment after percutaneous coronary intervention]. Zhongguo Zhong Xi Yi Jie He Za Zhi, 2011. 31(12): p. 1611-4."

504 "Westphal, S., et al., Antihypertensive treatment and homocysteine concentrations. Metabolism, 2003. 52(3): p. 261-3."

505 "Westreich, K.D., et al., Trajectories in estimated glomerular filtration rate in youth-onset type 1 and type 2 diabetes: The SEARCH for Diabetes in Youth Study. Journal of Diabetes and its Complications, 2021. 35(2): p. 107768."

506 "Wetterhall, M., et al., Assessment of the partitioning capacity of high abundant proteins in human cerebrospinal fluid using affinity and immunoaffinity subtraction spin columns. Journal of Chromatography B, 2010. 878(19): p. 1519-1530."

507 "Wijerathna, T.M., et al., Albuminuria and other renal damage biomarkers detect acute kidney injury soon after acute ingestion of oxalic acid and potassium permanganate. Toxicology Letters, 2018. 299: p. 182-190."

508 "Wilkinson, J.D., M. Diamond, and T.L. Miller, The promise of cardiovascular biomarkers in assessing children with cardiac disease and in predicting cardiovascular events in adults. Progress in Pediatric Cardiology, 2011. 32(1): p. 25-34."

509 "Wilson, M., et al., Biomarkers During Recovery From AKI and Prediction of Long-term Reductions in Estimated GFR. American Journal of Kidney Diseases, 2022. 79(5): p. 646-656.e1."

510 "Wippel, H.H., et al., Comparing intestinal versus diffuse gastric cancer using a PEFF-oriented proteomic pipeline. Journal of Proteomics, 2018. 171: p. 63-72."

511 "Wiromrat, P., et al., Serum uromodulin is associated with urinary albumin excretion in adolescents with type 1 diabetes. Journal of Diabetes and its Complications, 2019. 33(9): p. 648-650."

512 "Woitas, R.P., et al., Correlation of serum concentrations of cystatin C and creatinine to inulin clearance in liver cirrhosis. Clin Chem, 2000. 46(5): p. 712-5."

513 "Wojciechowska, M., et al., Remote Ischemic Preconditioning in Renal Protection During Elective Percutaneous Coronary Intervention. Adv Exp Med Biol, 2018. 1116: p. 19-25."

514 "Wu, C.-C., et al., Candidate Serological Biomarkers for Cancer Identified from the Secretomes of 23 Cancer Cell Lines and the Human Protein Atlas*. Molecular & Cellular Proteomics, 2010. 9(6): p. 1100-1117."

515 "Wunnapuk, K., et al., Renal biomarkers predict nephrotoxicity after paraquat. Toxicology Letters, 2013. 222(3): p. 280-288."

516 "Wunnapuk, K., et al., Use of a glyphosate-based herbicide-induced nephrotoxicity model to investigate a panel of kidney injury biomarkers. Toxicology Letters, 2014. 225(1): p. 192-200."

517 "Xiao, H., et al., Proteomic Analysis of Human Saliva From Lung Cancer Patients Using Two-Dimensional Difference Gel Electrophoresis and Mass Spectrometry*. Molecular & Cellular Proteomics, 2012. 11(2): p. M111.012112."

518 "Xiao, H., et al., Quantitative proteomic analysis of microdissected oral epithelium for cancer biomarker discovery. Oral Oncology, 2015. 51(11): p. 1011-1019."

519 "Xiao, N., et al., Kidney function in severely obese adolescents undergoing bariatric surgery. Obesity (Silver Spring), 2014. 22(11): p. 2319-25."

520 "Xu, M., et al., Type 2 Diabetes, Diabetes Genetic Score and Risk of Decreased Renal Function and Albuminuria: A Mendelian Randomization Study. EBioMedicine, 2016. 6: p. 162-170."

521 "Yajima, T. and K. Yajima, Serum creatinine-to-cystatin C ratio as an indicator of sarcopenia in hemodialysis patients. Clinical Nutrition ESPEN, 2023. 56: p. 200-206."

522 "Yan, G.L., et al., [Effects of remote ischemic preconditioning on contrast-induced acute kidney injury after percutaneous coronary intervention in patients with chronic total occlusion]. Zhonghua Yi Xue Za Zhi, 2021. 101(11): p. 776-781."

523 "Yang, T., et al., Serum proteomics analysis of candidate predictive biomarker panel for the diagnosis of trastuzumab-based therapy resistant breast cancer. Biomedicine & Pharmacotherapy, 2020. 129: p. 110465."

524 "Yang, X., et al., Prognosis and antibody profiles in survivors of critical illness from COVID-19: a prospective multicentre cohort study. British Journal of Anaesthesia, 2022. 128(3): p. 491-500."

525 "Yang, Y., et al., The efficacy of biomarkers in the diagnosis of acute kidney injury secondary to liver cirrhosis. Medicine (Baltimore), 2021. 100(14): p. e25411."

526 "Yetkin, E., et al., Increased plasma levels of cystatin C and transforming growth factor-beta1 in patients with coronary artery ectasia: can there be a potential interaction between cystatin C and transforming growth factor-beta1. Coron Artery Dis, 2007. 18(3): p. 211-4."

527 "Yildirim, E. and A.T. Cabbar, Association between copeptin and contrast-induced nephropathy in patients with ST-elevation myocardial infarction. Revista Portuguesa de Cardiologia (English Edition), 2019. 38(12): p. 873-879."

528 "Yin, L., et al., Probucol for the prevention of cystatin C-based contrast-induced acute kidney injury following primary or urgent angioplasty: a randomized, controlled trial. Int J Cardiol, 2013. 167(2): p. 426-9."

529 "Yohannes, E., et al., Molecular Targets for Diabetes Mellitus-associated Erectile Dysfunction*. Molecular & Cellular Proteomics, 2010. 9(3): p. 565-578."

530 "Yokoyama, H., T. Inoue, and K. Node, Effect of insulin-unstimulated diabetic therapy with miglitol on serum cystatin C level and its clinical significance. Diabetes Res Clin Pract, 2009. 83(1): p. 77-82."

531 "Yoo, J.-J., et al., Estimation of renal function in patients with liver cirrhosis: Impact of muscle mass and sex. Journal of Hepatology, 2019. 70(5): p. 847-854."

532 "Yoshii, I., T. Chijiwa, and N. Sawada, Screening osteoporotic femoral neck without measuring bone mineral density with the use of tartrate resistant acid phosphatase-5b and serum-creatinine-to-cystatin C ratio in Japanese postmenopausal women. Journal of Orthopaedic Science, 2020. 25(4): p. 671-676."

533 "Youssef, M.I., A.A.A. Mahmoud, and R.H. Abdelghany, A new combination of sitagliptin and furosemide protects against remote myocardial injury induced by renal ischemia/reperfusion in rats. Biochemical Pharmacology, 2015. 96(1): p. 20-29."

534 "Yu, Y.B., H.Z. Zhuang, and C. Liu, [Effect of Qishen Huoxue Granule for auxiliary treatment of critical cases of acute kidney injury]. Zhongguo Zhong Xi Yi Jie He Za Zhi, 2010. 30(8): p. 819-22."

535 "Zagidullin, N.S., et al., Nephroprotective effects of remote ischemic preconditioning in coronary angiography. Clin Hemorheol Microcirc, 2017. 65(3): p. 299-307."

536 "Zamora, E., et al., Renal function largely influences Galectin-3 prognostic value in heart failure. International Journal of Cardiology, 2014. 177(1): p. 171-177."

537 "Zang, T., et al., Characterization of the Blister Fluid Proteome for Pediatric Burn Classification. Journal of Proteome Research, 2019. 18(1): p. 69-85."

538 "Zawadzka, A.M., et al., Phosphoprotein Secretome of Tumor Cells as a Source of Candidates for Breast Cancer Biomarkers in Plasma*. Molecular & Cellular Proteomics, 2014. 13(4): p. 1034-1049."

539 "Zechner, C., et al., Hypophosphatemia in acute liver failure of a broad range of etiologies is associated with phosphaturia without kidney damage or phosphatonin elevation. Translational Research, 2021. 238: p. 1-11."

540 "Zeng, J., W. Tong, and P. Zheng, Decreased risk of acute kidney injury with intracranial pressure monitoring in patients with moderate or severe brain injury. J Neurosurg, 2013. 119(5): p. 1228-32."

541 "Zhang, D., et al., Resistive index as predictor of acute kidney injury in patients with non-ST-segment elevation myocardial infarction. The American Journal of the Medical Sciences, 2024. 367(3): p. 190-194."

542 "Zhang, J., et al., New strategy for clinical etiologic diagnosis of acute ischemic stroke and blood biomarker discovery based on machine learning??Electronic supplementary information (ESI) available. See https://doi.org/10.1039/d2ra02022j. RSC Advances, 2022. 12(23): p. 14716-14723."

543 "Zhang, J.-B., et al., Biomarkers of Renal Function in Type 2 Diabetic Patients with Cognitive Impairment. Neuroscience Letters, 2016. 610: p. 19-23."

544 "Zhang, L., et al., Developing an ensemble machine learning model for early prediction of sepsis-associated acute kidney injury. iScience, 2022. 25(9): p. 104932."

545 "Zhang, N., et al., Quantitative analysis of differentially expressed saliva proteins in human immunodeficiency virus type 1 (HIV-1) infected individuals. Analytica Chimica Acta, 2013. 774: p. 61-66."

546 "Zhang, Q., et al., Clinical analysis of risk factors for severe COVID-19 patients with type 2 diabetes. Journal of Diabetes and its Complications, 2020. 34(10): p. 107666."

547 "Zhang, X., et al., Profiling serologic biomarkers in cirrhotic patients via high-throughput Fourier transform infrared spectroscopy: toward a new diagnostic tool of hepatocellular carcinoma. Translational Research, 2013. 162(5): p. 279-286."

548 "Zhang, Y., et al., A comprehensive map and functional annotation of the normal human cerebrospinal fluid proteome. Journal of Proteomics, 2015. 119: p. 90-99."

549 "Zhang, Z., et al., Plasma tissue-type plasminogen activator is associated with lipoprotein(a) and clinical outcomes in hospitalized patients with COVID-19. Research and Practice in Thrombosis and Haemostasis, 2023. 7(6): p. 102164."

550 "Zhao, X., et al., Polybrominated diphenyl ethers and decabromodiphenyl ethane in paired hair/serum and nail/serum from corresponding chemical manufacturing workers and their correlations to thyroid hormones, liver and kidney injury markers. Science of The Total Environment, 2020. 729: p. 139049."

551 "Zhou, F., et al., Effects of remote ischemic preconditioning on contrast induced nephropathy after percutaneous coronary intervention in patients with acute coronary syndrome. Medicine (Baltimore), 2018. 97(2): p. e9579."

552 "Zhou, Y., et al., Diabetic Nephropathy Can Be Treated with Calcium Dobesilate by Alleviating the Chronic Inflammatory State and Improving Endothelial Cell Function. Cell Physiol Biochem, 2018. 51(3): p. 1119-1133."

553 "安瑞华, 刘.A.刘.A.杨.A., 血清学标志物在肾积水诊断及判断预后中的意义. 临床泌尿外科杂志, 2018. 33(5)."

554 "半胱氨酸蛋白酶抑制剂C在肾脏疾病诊断中的应用. 山西医药杂志（下半月版）, 2009. 38(10): p. 942-943."

555 "包塔娜, et al., 应用ROC曲线分析血清标志物联合检测对早期糖尿病视网膜病变诊断价值. 当代医学, 2018. 24(29): p. 38-41."

556 "边召允, 胱抑素 C,肌酐,尿素在早期肾损害中的应用评价. 医药前沿, 2012(32): p. 375-376."557 "蔡均均 and 韩涛, 肝硬化患者急性肾损伤的诊断与治疗. 临床肝胆病杂志, 2014(12): p. 1352-1356."

558 "蔡文慧, 血清胱抑素C浓度在Ⅱ型糖尿病肾病早期诊断中的临床价值. 临床和实验医学杂志, 2011. 10(04): p. 255-256."

559 "蔡叶萍, 胱抑素C在诊断妊娠期糖尿病患者早期肾功能损害中的价值. 2013."

560 "蔡远扬, 血清同型半胱氨酸和胱抑素C水平用于糖尿病早期肾损害诊断临床价值. 山西医药杂志, 2023. 52(2): p. 123-125."

561 "曹巧华, CysC、hsCRP、尿糖及尿微量蛋白在糖尿病早期肾损害诊断中的应用价值. 医学食疗与健康, 2021. 19(10): p. 159-160."

562 "曹文斋, 张.A.汪.A., 血浆氧化三甲胺水平与非瓣膜性心房颤动的相关性研究. 中华老年心脑血管病杂志, 2023. 25(4)."

563 "曹雪峰, et al., 血清胱抑素C和基质金属蛋白酶-2联合检测在糖尿病肾病早期诊断的临床意义. 山西医药杂志（下半月版）, 2013. 42(4): p. 444-445."

564 "曹云友 and 姜玉禄, 胱抑素C与其他标志物对早期糖尿病肾病中的诊断效能. 现代预防医学, 2015. 42(14): p. 2669-2671."

565 "曾宪飞, et al., 尿肝型脂肪酸结合蛋白与糖尿病肾损伤的相关性. 现代检验医学杂志, 2013. 28(1): p. 56-58."

566 "曾晓妹, 血清胱抑素C与冠心病的相关性研究. 现代医药卫生, 2012. 28(20): p. 3118-3120."

567 "曾秀雅, 陈海明, and 王燕凤, 四项指标联合检测在肾脏疾病早期诊断中的应用. 中国卫生标准管理, 2017. 8(23): p. 119-122."

568 "柴芙蓉, 射血分数中间值心力衰竭患者血清中sST2的变化及其与相关指标的对比分析. 2019."

569 "陈华, 顾.A.周.A., 糖尿病周围神经病变患者血清总胆汁酸和总胆红素水平变化及其诊断价值分析. 检验医学与临床, 2022. 19(24)."

570 "陈华英, et al., 脑脊液胱抑素C和乳酸脱氢酶在化脓性和病毒性脑膜炎的应用. 实验与检验医学, 2012. 30(1): p. 57-58,72."

571 "陈佳, 曲冬颖, and 韩悦, 子痫前期患者血清PAPP-A、β-hCG、CysC水平及临床价值. 保健医学研究与实践, 2022. 19(2): p. 66-69."

572 "陈金玲, et al., 睾丸素-2在糖尿病肾损伤中的诊断价值. 检验医学与临床, 2023. 20(7): p. 892-894,898."

573 "陈客, 王.A.陈.A.何.A., 尿诱骗受体2/肌酐水平与糖尿病肾小管病预后的关系. 中华肾病研究电子杂志, 2023. 12(2)."

574 陈磊. 芪术胶囊干预糖尿病肾病大鼠肾组织的代谢组学研究. 2015.

575 "陈闻婕, et al., 重症急性胰腺炎合并急性肾损伤的临床预防. 中国中西医结合急救杂志, 2022. 29(5): p. 513-517."

576 "陈曦, 王.A., 血清标志物Urea、Creat、CysC及NGAL检测在早期诊断糖尿病肾病中的应用价值. 当代医药论丛, 2020. 18(7)."

577 "陈跃, 赵春, and 赵娜, 血清Sestrin2与胱抑素C在缺血性脑卒中早期诊断中的临床价值. 健康研究, 2020. 40(6): p. 680-683."

578 "陈志晓, et al., 肾脏相关标志物在评估妊娠高血压综合征肾损伤的研究. 检验医学与临床, 2015. 12(11): p. 1542-1544."

579 "池锐彬, et al., 血清胱抑素C联合APACHEⅡ评分预测脓毒症急性肾损伤的临床研究. 中华急诊医学杂志, 2018. 27(10): p. 1136-1141."

580 "崔红萍, et al., 血清胱抑素C和尿NAG联合检测在糖尿病肾病早期诊断中的应用. 实用检验医师杂志, 2023. 15(4): p. 364-367."

581 "崔巍, 血清同型半胱氨酸和胱抑素C与2型糖尿病肾病的关系. 2014."

582 "代瑛, et al., 肾综合征出血热患者血清Cys-C测定临床意义. 陕西医学杂志, 2011. 40(7): p. 884-886."

583 "戴新贵, NGAL在脓毒症急性肾损伤中的应用价值. 2017."

584 "党艳梅, et al., 血清透明质酸和层黏蛋白等对肾脏纤维化早期诊断的研究. 国际移植与血液净化杂志, 2010. 8(1): p. 26-30."

585 "邓曼, et al., 血清胱抑素C及尿微量白蛋白与尿肌酐比值在糖尿病肾病诊断中的应用. 医学检验与临床, 2017. 28(1): p. 43-44,59."

586 "邓美珍, 糖尿病肾病患者肾功能评价中胱抑素C检测的应用. 中国卫生产业, 2011(31): p. 95,97."

587 "邓勇莹, 陆.A.侯.A., 尿液足细胞标志蛋白在糖尿病患者早期肾功能损害中的诊断价值. 现代医药卫生, 2013. 29(13)."

588 "丁爱华 and 王尚武, 血清视黄醇结合蛋白4在糖尿病肾病早期诊断中的临床价值. 检验医学与临床, 2013. 10(13): p. 1682-1683."

589 "丁修冬, et al., 血清Hcy、Mb、Cys-C检测对肾功能损害的评价. 标记免疫分析与临床, 2010. 17(5): p. 284-286."

590 "丁燕玲, et al., 胱抑素C在肾病、糖尿病、高血压患者中的检测结果分析. 现代中西医结合杂志, 2008. 17(31): p. 4907."

591 "董佳月 and 倪红英, 胱抑素C对体外循环术后急性肾损伤的早期预测价值的研究. 中国现代医生, 2017. 55(25): p. 4-7,封3."

592 "董敏 and 周厚清, 胱抑素C在糖尿病性肾病中的价值. 中国卫生检验杂志, 2009. 19(7): p. 1590-1591."

593 "董平, 钟.A.宋.A., 尿肾损伤分子1和尿胱抑素C在重症监护室万古霉素肾损伤早期诊断中的作用. 中国临床药理学杂志, 2016. 32(2)."

594 "董霞 and 韩睿, 血清生物标志物对糖尿病肾病早期诊治的临床价值. 医学综述, 2016. 22(10): p. 1945-1948."

595 "董霞, 血清生物标志物对糖尿病肾病早期诊治的临床价值. 医学综述, 2016. 22(10): p. 1945-1948."

596 "范铿娜, 自身免疫性脑炎与病毒性脑炎临床特征分析及鉴别诊断标志物初探. 2021."

597 "范玲, NGAL Cys-C ET-1联合检测对高血压肾损伤的诊断价值. 基层医学论坛, 2020. 24(22): p. 3211-3212."

598 "方金山, 早期肾损伤标志物在老年高血压肾损害中的诊断价值. 医疗装备, 2020. 33(8): p. 39-40."

599 "方美丽 and 贾国昌, 早期肾功能损伤检验方法的探讨. 中国社区医师（医学专业）, 2011. 13(12): p. 191."

600 "费素娟, 马.A., 血清胱抑素C对结直肠组织良恶性病变诊断价值. 中国中西医结合消化杂志, 2019. 27(2)."

601 "冯慧月, 血清半胱氨酸蛋白酶抑制剂C与急性脑梗死的临床相关性分析. 2020."

602 "冯倩, 糖尿病肾病患者尿液非靶向代谢组学研究. 2021."

603 "俸家富, 急性肾损伤的实验室检测标志物. 中华检验医学杂志, 2014(6): p. 410-414."

604 "俸家富, 杨.A.黄.A.陈.A., 胱抑素C及其eGFR方程在诊断肝硬化患者肾损害中的应用. 现代检验医学杂志, 2016. 31(4)."

605 "府伟灵, 黄.A., 急性肾损伤早期实验诊断标志物. 国际检验医学杂志, 2010. 31(5)."

606 "付凤仙, 高.A.张.A.李.A., 子痫前期患者血清CXCL16、FGF21表达水平及临床意义. 实用医学杂志, 2020. 36(8)."

607 "付文金, 尿液组织因子促凝活性检测方法的建立及其在糖尿病肾病中的应用研究. 2011."

608 "付燕 and 赵斌, 急性肾损伤早期诊断的生物标志物. 中国危重病急救医学, 2012. 24(9): p. 571-573."

609 "付仲娇, 血清S100β蛋白水平、颈动脉粥样硬化与脑微出血的相关性研究. 2023."

610 "傅宏杰, 急性冠脉综合征患者血清可溶性CD40L的临床研究. 2011."

611 "高丹, 应用尿PCX/CR鉴别血尿来源和早期诊断肾脏损伤的性能评价. 2014."

612 "高继东, 刘.A., 血清胱抑素、视黄醇结合蛋白和白细胞介素-6联合检测在老年人糖尿病早期肾损害中的应用. 中国老年学杂志, 2014. 34(5)."

613 "高娟梅, 血清CysC和尿PCX在妊娠糖尿病早期肾损害诊断中的应用价值. 中国优生与遗传杂志, 2016. 24(5)."

614 "高科, 胱抑素C联合尿RBP检测在糖尿病患者早期肾损伤中诊断价值. 中国实用医药, 2019. 14(8): p. 58-59."

615 "高丽欣, et al., 子痫前期患者血清CXCL16、FGF21表达水平及临床意义. 实用医学杂志, 2020. 36(8): p. 1102-1106."

616 "高利萍, 血清胱抑素C和β<,2>微球蛋白评价糖尿病肾脏损害的临床意义. 2010."

617 "高倩, et al., 急性脑梗死患者血清微小RNA-145、程序性细胞死亡因子4 mRNA水平变化及诊断价值研究. 实用心脑肺血管病杂志, 2022. 30(2): p. 18-23."

618 "高翔, et al., 血浆肝素结合蛋白对脓毒症相关急性肾损伤的早期诊断及预后预测. 临床急诊杂志, 2021. 22(10): p. 647-652."

619 "高星辰, 陶月红, and 郭玉秀, 血清胱抑素C联合视黄醇结合蛋白在儿童1型糖尿病酮症酸中毒急性肾损伤中的预测价值. 中国妇幼保健, 2023. 38(12): p. 2199-2202."

620 "高照华, et al., 血清CEA、NSE、CA199、CA153、CA125对2型糖尿病视网膜病变的预测价值. 徐州医科大学学报, 2022. 42(3): p. 181-188."

621 "葛锁华, et al., 胃癌患者手术治疗前后血清CEA、IL-8和Cys C检测的临床意义. 放射免疫学杂志, 2011. 24(4): p. 399."

622 "宫敏敏, 杨亦彬, and 张世先, 急性肾损伤相关生物学标志物在重症监护病房患者中的应用价值. 中南大学学报（医学版）, 2015. 40(10): p. 1083-1088."

623 "龚倩, 阚.A.林.A.梁.A., 尿L-FABP与ACR联合检测在妊娠期高血压综合征早期肾损伤中的诊断价值. 检验医学与临床, 2019. 16(9)."

624 "龚心琰, 血清胱抑素C水平与慢性心力衰竭的相关性研究. 2017."

625 "拱忠影, ALS患者血清及脑脊液生物标志物探索. 2020."

626 "顾冠聪, 周懿忆, and 陈华, 糖尿病周围神经病变患者血清总胆汁酸和总胆红素水平变化及其诊断价值分析. 检验医学与临床, 2022. 19(24): p. 3396-3400."

627 "顾光大, 胱抑素C在肝硬化病人肾小球滤过率评估中的价值. 当代医学, 2007(23): p. 159-160."

628 "顾科萍, 姜.A., 血清β2微球蛋白、胱抑素C、肌酐、超敏C反应蛋白对早期糖尿病肾病的评价价值. 右江医学, 2020. 48(1)."

629 "顾明, 胡.A., 慢性心力衰竭合并2型糖尿病患者血清生长分化因子-15和胱抑素C的变化. 北华大学学报（自然科学版）, 2022. 23(5)."

630 "关畅, Galectin-3和Cys-C在老年2型心肾综合征患者中的诊断价值. 2021."

631 "官瑞磊, et al., 老年动脉粥样硬化性脑梗死患者血管重构与血清胱抑素C水平的关系研究. 中华老年心脑血管病杂志, 2021. 23(5): p. 515-518."

632 "韩静, 子痫前期患者血清胱抑素C水平变化的研究. 2013."

633 "韩涛, 蔡.A., 肝硬化患者急性肾损伤的诊断与治疗. 临床肝胆病杂志, 2014. 30(12)."

634 "韩颖, 李素芬, and 刘艳, 血清胱抑素C与糖尿病肾病. 国际内分泌代谢杂志, 2014. 34(3): p. 207-210."

635 "韩悦, 陈.A.曲.A., 子痫前期患者血清PAPP-A、β-hCG、CysC水平及临床价值. 保健医学研究与实践, 2022. 19(2)."

636 "韩宗海 and 祁月英, 胱抑素C在Ⅱ型糖尿病早期肾脏损害诊断中的价值. 医学信息, 2015(37): p. 350-351."

637 "汉雯, 老年心血管疾病患者左室肥厚与肾功能的相关研究. 2015."

638 "郝建军, 血清胱抑素C、尿微量白蛋白在糖尿病早期肾损害诊断中的应用价值评估. 实验与检验医学, 2011. 29(2): p. 171-172."

639 "郝丽, 包.A.潘.A.齐.A., 应用ROC曲线分析血清标志物联合检测对早期糖尿病视网膜病变诊断价值. 当代医学, 2018. 24(29)."

640 "郝晓萍, et al., 尿肝型脂肪酸结合蛋白血清胱抑素C对急性失代偿性心力衰竭患者发生急性肾损伤的预测价值. 中国急救医学, 2019. 39(9): p. 859-863."

641 "何晗, 刘新君, and 魏炯, 外周血Th17细胞对脓毒症急性肾损伤的早期诊断价值. 检验医学与临床, 2018. 15(11): p. 1597-1599."

642 "何灵杰, 中西医结合治疗早中期糖尿病肾病前后CysC的变化及临床意义. 2008."

643 "何龙, 急性脑梗死患者早期肾功能指标与脑微出血的相关性研究. 2019."

644 "何敏, et al., 血清胱抑素C在多发性软组织挫伤患者急性肾损伤早期诊断中的应用. 肾脏病与透析肾移植杂志, 2013. 22(1): p. 10-14."

645 "何囡囡, et al., 肾损伤分子1及胱抑素C对万古霉素早期肾损害的诊断价值. 中国急救复苏与灾害医学杂志, 2018. 13(8): p. 763-765."

646 "何囡囡, ICU患者万古霉素谷浓度与肾损害的相关性及早期生物学标志物的研究. 2014."

647 "何艳, 陈.A.林.A.洪.A.陈.A., 脑脊液胱抑素C和乳酸脱氢酶在化脓性和病毒性脑膜炎的应用. 实验与检验医学, 2012. 30(1)."

648 "洪国粦, 谢.A.练.A.林.A., 肾损伤标志物在儿童过敏性紫癜性肾炎中的应用价值比较. 医学理论与实践, 2018. 31(13)."

649 "呼双琴, 李.A., 血清胱抑素C在诊断2型糖尿病早期肾损伤的应用及临床价值. 中国实验诊断学, 2015. 19(7)."

650 "胡军红, et al., 尿NGAL与血清Cys-C联合检测在糖尿病肾病早期诊断中的应用. 实验与检验医学, 2017. 35(6): p. 921-923."

651 "胡美玲 and 顾明, 慢性心力衰竭合并2型糖尿病患者血清生长分化因子-15和胱抑素C的变化. 北华大学学报（自然科学版）, 2022. 23(5): p. 616-619."

652 "胡晓璐, 代谢和炎性因子预测妊娠期糖尿病的价值探讨. 2022."

653 "胡雪峰, 探讨心肌酶及胱抑素C水平对急性心肌梗死 患者的临床诊断价值分析. 中国保健营养, 2017. 27(34): p. 284-285."

654 "华川, 王.A., 肾损伤标志物在糖尿病肾病早期诊断中的应用. 解放军医药杂志, 2012. 24(2)."

655 "黄安乐, 血清胱抑素C与老年射血分数保留性心力衰竭的相关性研究. 2019."

656 "黄健, 冠脉CTA对比剂应用对患者肾功能的影响. 2013."

657 "黄健伟, 王.A.顾.A., 血清胱抑素C检测对肝肾综合征的诊断价值. 中国实验诊断学, 2007. 11(12)."

658 "黄君富, 急性肾损伤早期实验诊断标志物. 国际检验医学杂志, 2010. 31(5): p. 462-464."

659 "黄琦, et al., 2型糖尿病肾病患者血清趋化素与胱抑素C水平的相关性. 成都医学院学报, 2019. 14(6): p. 732-735."

660 "黄珊, 肾功能指标与认知功能和ATN标志物的相关性及阿尔茨海默病诊断模型的研究. 2022."

661 "黄秀丽, 张惠莉, and 高继东, 血清胱抑素C在早期糖尿病肾病诊断中的价值. 中国医学前沿杂志（电子版）, 2010. 02(3): p. 76-79."

662 "黄雪娥, 中性粒细胞明胶酶相关载脂蛋白、胱抑素C在PCI术后早期预测对比剂肾病中的意义. 2013."

663 "黄艳芳, 陈燕, and 余小龙, 恶性肿瘤患者血清胱抑素C与β2-微球蛋白检测的意义. 福建医药杂志, 2012. 34(3): p. 83-85."

664 "黄玉蓉, 刘.A.张.A., 阻塞性睡眠呼吸暂停患者早期肾损伤生物标志物的研究. 兵团医学, 2019. 0(3)."

665 "霍颖超, 脑小血管病临床风险评估相关标志物及与卒中预后关系的研究. 2020."

666 "季红慧 and 罗秀英, 血浆标志物与慢性心力衰竭患者急性心力衰竭发作及肾功能异常的关系研究. 浙江医学, 2016. 38(12): p. 943-945,1030."

667 "贾爱华, et al., 胱抑素C及其他诊断糖尿病肾病标志物效能比较. 中国医药导报, 2012. 9(27): p. 58-59+61."

668 "贾燕午 and 李彦, 血清胱抑素C、同型半胱氨酸及尿β2-微球蛋白在诊断肾衰竭中的应用价值. 山西卫生健康职业学院学报, 2020. 30(3): p. 29-30."

669 "简志刚, 池.A.梁.A.邹.A.魏.A., 血清胱抑素C联合APACHEⅡ评分预测脓毒症急性肾损伤的临床研究. 中华急诊医学杂志, 2018. 27(10)."

670 "江城, et al., 血中性粒细胞明胶酶相关脂质运载蛋白及胱抑素C对输尿管结石梗阻致急性肾损伤的早期诊断价值. 东南国防医药, 2018. 20(3): p. 240-243."

671 "姜宇海 and 顾科萍, 血清β2微球蛋白、胱抑素C、肌酐、超敏C反应蛋白对早期糖尿病肾病的评价价值. 右江医学, 2020. 48(1): p. 45-48."

672 "蒋芳, 脑卒中患者发生急性肾损伤的危险因素及预测标志物的研究. 2019."

673 "蒋倩, 重度阻塞性睡眠呼吸暂停低通气综合征患者启动早期肾损害. 2016."

674 "蒋清翠 and 周建明, 胱抑素C在糖尿病性肾病中的应用评价. 现代医药卫生, 2007. 23(9): p. 1313-1314."

675 "蒋琰, et al., 血清胱抑素C、同型半胱氨酸联合检测对诊断高血压早期肾病的意义. 重庆医学, 2015(9): p. 1193-1196."

676 "焦彬, et al., 血肌酐与胱抑素C的比值在溃疡性结肠炎合并骨骼肌质量减少中的意义. 现代消化及介入诊疗, 2022. 27(11): p. 1392-1396,1401."

677 "焦翔 and 张继东, 胱抑素C及α1-微球蛋白检测对糖尿病肾病诊断作用. 中国社区医师, 2021. 37(22): p. 92-93."

678 "金玲玲, 血尿酸、胱抑素-C、CA125、同型半胱氨酸与左心功能不全相关性的临床研究. 2015."

679 "金旗, 肺血栓栓塞症蛋白质组学及介入治疗研究. 2020."

680 "靳鹏, et al., 左心疾病相关性肺动脉高压患者血清生化标志物的特征. 中国循环杂志, 2016. 31(4): p. 362-366."

681 "靳鹏, PH-LHD循环标志物的变化及其参与肺血管重构的机制. 2016."

682 "井长信, 贾.A.王.A.刘.A., 胱抑素C及其他诊断糖尿病肾病标志物效能比较. 中国医药导报, 2012. 9(27)."

683 "敬婧, 尿液细胞外囊泡VEGF-A165b在糖尿病肾病中的诊断价值研究. 2022."

684 "鞠建国 and 吴家辉, 血清胱抑素C检测评价妊高征肾功能. 中西医结合心血管病电子杂志, 2016. 4(16): p. 40-40,41."

685 "阚林, et al., 尿L-FABP与ACR联合检测在妊娠期高血压综合征早期肾损伤中的诊断价值. 检验医学与临床, 2019. 16(9): p. 1164-1166."

686 "阚明, 林.A.张.A., 糖尿病肾病患者血清胱抑素C检测的临床意义. 中国医药导刊, 2011. 13(10)."

687 "康敏, 朱.A.张.A., 生物标志物联合检测在早期糖尿病肾病诊断中的应用. 检验医学与临床, 2020. 17(2)."

688 "赖丽娟, 姜建珍, and 徐晓薇, 血清胱抑素C、肿瘤坏死因子-α水平对早期糖尿病肾病的诊断价值. 中外医学研究, 2023. 21(29): p. 71-75."

689 "雷寓淇, et al., 糖尿病肾病患者Piwi相互作用RNA差异表达谱分析. 中华肾脏病杂志, 2023. 39(4): p. 253-262."

690 "黎晓冬, 糖尿病视网膜病变伴肾病的临床特征及基于细胞焦亡研究补肾活血方对db/db小鼠视网膜和肾脏的保护作用. 2023."

691 "黎艳, et al., 胱抑素C在早期糖尿病肾病诊疗中的作用. 湖北民族学院学报（医学版）, 2012. 29(3): p. 28-30."

692 "李爱军 and 李银萍, Cys C、hsCRP、UMA联合检测在诊断糖尿病早期肾损害中的价值. 中国现代医生, 2010. 48(8): p. 84-84."

693 "李成博, 脑梗死患者血清sLOX-1、Cyst-C等生物标志物与颈动脉粥样硬化斑块形成的相关性. 2016."

694 "李东杰, 脑梗死患者APC、APS及AT-Ⅲ等生物标志物与颈动脉粥样硬化的相关性分析. 2017."

695 "李飞星, 血清白介素6-受体、胱抑素C、超敏C反应蛋白在急性冠脉综合征中的表达及对易损斑块的预测价值. 2018."

696 "李丰尧, 检测血清胱抑素C在糖尿病肾病临床价值分析. 现代预防医学, 2012. 39(13): p. 3360-3361."

697 "李刚, 血清胱抑素C在糖尿病肾病早期诊断中的价值. 中国保健营养（中旬刊）, 2014(5): p. 2736-2736."

698 "李光荣, 刘靳波, and 明兰, 血清视黄醇结合蛋白和胱抑素C在2型糖尿病早期肾功能损害中的应用. 检验医学与临床, 2014(19): p. 2663-2664,2667."

699 "李海霞, 王.A.韩.A., 血清胱抑素C在ICU危重患者急性肾损伤早期诊断中的价值. 临床急诊杂志, 2013. 14(4)."

700 "李红艳, 血清胱抑素C与慢性缺血性心力衰竭患者心功能水平的关系. 中国医药指南, 2014(32): p. 131-132."

701 "李静文, 基于医疗大数据平台的糖尿病肾病早期诊断的相关指标研究. 2021."

702 "李菊丹, 血清胱抑素C在糖尿病肾病早期诊断中的意义. 中国冶金工业医学杂志, 2012. 29(3): p. 274-275."

703 "李君君, 心型脂肪酸结合蛋白、神经元特异性烯醇化酶与急性脑梗死病情转归及预后评估的相关性. 2016."

704 "李莉, et al., 测定血清胱搁1素C在肾脏疾病中的诊断价值. 中华全科医学, 2011. 09(3): p. 457-458."

705 "李平, 蔡伟娟, and 张丽翠, 胱抑素 C 尿素和肌酐在不同程度肾病中的临床价值. 检验医学与临床, 2012(18): p. 2292-2293."

706 "李青霖, 高龄老年人AKI预后分析及自噬和ASPPs在AKI中的早期诊断价值. 2017."

707 "李青泉, 血清Cys-C、H-FABP及NAG在老年2型心肾综合征中的临床价值评估. 2020."

708 "李素华, 何.A.桑.A.刘.A.王.A., 血清胱抑素C在多发性软组织挫伤患者急性肾损伤早期诊断中的应用. 肾脏病与透析肾移植杂志, 2013. 22(1)."

709 "李伟 and 呼双琴, 血清胱抑素C在诊断2型糖尿病早期肾损伤的应用及临床价值. 中国实验诊断学, 2015(7): p. 1175-1176."

710 "李文红, 血清Cys-C、VEGF、CA153联合检测在乳腺癌早期诊断中的临床价值. 2020."

711 "李雯妮, et al., 血生长停滞特异性蛋白6、胱抑素C与老年2型糖尿病肾病相关性的研究. 现代生物医学进展, 2016. 16(36): p. 7034-7037."

712 "李锡敬 and 许柳芹, 血清胱抑素 C 检测在肾脏疾病诊断中的应用. 检验医学与临床, 2012(23): p. 2982-2983."

713 "李香, 袁.A.类.A.李.A.金.A.成.A., 组织蛋白酶K、胱抑素C与冠心病及其危险因素的相关性. 中国老年学杂志, 2015. 35(23)."

714 "李湘, 尚.A.张.A., 血清Cystatin C、MMP-2及hs-CRP/PAB对急性心肌梗死患者PCI术后并发心衰的预测价值. 河北医药, 2019. 41(3)."

715 "李小全, 王跃玲, and 李伟华, 血清ANCA、补体C5a和胱抑素C联合检测对小血管炎性肾炎的诊断价值. 医学新知杂志, 2018. 28(z1): p. 6-7."

716 "李晓军, 降钙素原、白细胞介素--6、同型半胱氨酸及D--二聚体在痛风患者中的表达及其临床意义. 2023."

717 "李晓琳, 血清胱抑素C测定在糖尿病肾病早期肾损伤中的检测价值. 天津医科大学学报, 2011. 17(4): p. 536-538."

718 "李鑫, et al., 2型糖尿病和糖尿病肾病患者血清微小RNA-148b-3p的水平变化及意义. 中华肾脏病杂志, 2018. 34(5): p. 348-354."

719 "李星缘, 张.A.彭.A.宋.A., 胱抑素C与β2-微球蛋白在多发性骨髓瘤病情及近期疗效评估中的应用. 国际肿瘤学杂志, 2019. 46(10)."

720 "李绪飞 and 丁树红, 高血压患者血清胱抑素-C和尿微量白蛋白检测的临床意义. 西部医学, 2011. 23(10): p. 1920-1921."

721 "李艳梅, 锌-α2-糖蛋白与原发性肾小球疾病肾间质纤维化及白蛋白诱导NRK-52E细胞转分化程度的关系. 2014."

722 "李艳平, 2型糖尿病肾病与氨基酸及脂质代谢的相关性研究. 2019."

723 "李尧, 血清-腹水白蛋白梯度联合胱抑素C检测在早期诊断肝肾综合征中的价值. 2023."

724 "李荫桂, 张华, and 胡筱梅, 血清胱抑素C检测在狼疮性肾病肾功能监测中的意义. 国际检验医学杂志, 2013. 34(6): p. 750-752."

725 "李永莉, 血浆NGAL和CysC在糖尿病肾病早期诊断中的应用价值. 临床医学研究与实践, 2019. 4(32): p. 129-131."

726 "李远眺 and 覃兰, 胱抑素-C在肾脏疾病中的应用价值. 右江医学, 2007. 35(1): p. 37-38."

727 "李云婷, et al., 血小板活化标志物CD62P,CD63与血清胱抑素C 联合检测在糖尿病肾病早期诊断的临床研究. 现代检验医学杂志, 2013. 28(1): p. 53-55."

728 "李泽源, 血清胱抑素C与急性缺血性脑卒中(AIS)复发风险之间的关系研究及我院AIS老年患者潜在不适宜用药分析. 2021."

729 "梁继铁 and 郭应军, N末端B型利钠肽原和胱抑素C联合检测对Ⅱ型心肾综合征的诊断价值. 右江民族医学院学报, 2014(2): p. 175-177."

730 "梁景彪, 血清总胆红素和脑钠肽前体在慢性心力衰竭的临床研究. 中国卫生标准管理, 2019. 10(21): p. 98-99."

731 "梁雪岩, 血清胱抑素C(CystaiC)在糖尿病肾功能损害诊断中的临床意义. 甘肃科技, 2014. 30(15): p. 140-141."

732 "梁毅珊, 血清胱抑素C对糖尿病肾病诊断的现实意义. 医药前沿, 2012. 02(8): p. 209-210."

733 "廖洪, 系统性红斑狼疮患者狼疮性肾炎诊断的多指标临床评价. 四川医学, 2014. 35(1)."

734 "廖涛, T淋巴细胞lncRNA表达水平与系统性红斑狼疮的关联性分析. 2022."

735 "林辉, 张江淮, and 阚明, 糖尿病肾病患者血清胱抑素C检测的临床意义. 中国医药导刊, 2011. 13(10): p. 1713,1715."

736 "林泉根, 梁嘉福, and 卢登球, 糖尿病肾病早期血、尿胱抑素C测定的临床价值研究. 包头医学, 2015. 39(1): p. 16-18."

737 "林伟卓, 血清 Cys C、β2-MG、α1-MG 在先天性肾积水患儿中的表达及术前诊断价值? 国际检验医学杂志, 2015(16): p. 2317-2318."

738 "林伟卓, 血清Cys C、β_2-MG、α_1-MG在先天性肾积水患儿中的表达及术前诊断价值. 国际检验医学杂志, 2015. 36(16): p. 2317-2318."

739 "林长亮 and 冯星火, 中性粒细胞明胶酶相关载脂蛋白、肾损伤分子-1及胱抑素-C对ICU脓毒症患者并发急性肾损伤的诊断效能. 中国实用乡村医生杂志, 2020. 27(5): p. 54-56."

740 "刘春晓, NGAL、Cys-C与急性有机磷农药中毒急性肾损伤的关联研究. 2010."

741 "刘晗 and 高继东, 血清胱抑素、视黄醇结合蛋白和白细胞介素-6联合检测在老年人糖尿病早期肾损害中的应用. 中国老年学杂志, 2014. 34(5): p. 1391-1392."

742 "刘汉冕, ICU患者急性肾损伤的早期诊断标志物初探. 中国实用医药, 2014(31): p. 61-62."

743 "刘环, 血清Cys-C与尿mALB联合检测在糖尿病肾病早期诊断中的应用价值. 国际检验医学杂志, 2015(18): p. 2724-2726."

744 "刘慧, 肾功指标在重度子痫前期中的临床意义. 2022."

745 "刘建球, 血清胱抑素C及尿微量蛋白对糖尿病肾病早期损害的诊断价值. 中国当代医药, 2013. 20(33): p. 103-104."

746 "刘健男, et al., 血清学标志物在肾积水诊断及判断预后中的意义. 临床泌尿外科杂志, 2018. 33(5): p. 393-397."

747 "刘婧, et al., 糖尿病肾病患者血浆NGAL和血清CysC水平改变及其早期诊断价值. 现代生物医学进展, 2015. 15(30): p. 5945-5947,5900."

748 "刘露露, Ⅰ--Ⅳ期糖尿病肾病血清胱抑素C与颈动脉内膜中层厚度的关系. 2019."

749 "刘敏, 卢.A.薛.A.王.A.钟.A.钟.A., 血清胱抑素C(Cys-C)在新生儿缺氧缺血性脑病(HIE)肾功能评价中的价值. 实验与检验医学, 2016. 34(3)."

750 "刘全良, et al., 探讨血清胱抑素C检测在糖尿病肾病诊断的临床意义. 标记免疫分析与临床, 2012. 19(2): p. 117-118."

751 "刘婉秋, 氨基酸与脂质的代谢物谱异常在2型糖尿病肾病关联研究. 2021."

752 "刘伟, 倪瑛, and 何婕, 血管外肺水联合肾损伤标志物检测对脓毒症致AKI患者的预后评估价值. 现代实用医学, 2019. 31(7): p. 972-974,封4."

753 "刘卫涛, 血清胱抑素C、血清β_2-微球蛋白、全血超敏C反应蛋白联合检测高血压早期肾损害的临床价值. 临床医学研究与实践, 2018. 3(19)."

754 "刘小丽, 张海涛, and 黄玉蓉, 阻塞性睡眠呼吸暂停患者早期肾损伤生物标志物的研究. 兵团医学, 2019(3): p. 4-5."

755 "刘晓敏, 王新芹, and 刘东声, 血清糖类抗原19-9糖类抗原125和胱抑素C在胰腺癌诊断中的临床意义. 实用医技杂志, 2013. 20(10): p. 1088-1089."

756 "刘芯宇, Cys C与尿微量清蛋白测定对糖尿病肾病早期诊断价值的比较. 检验医学与临床, 2013(18): p. 2383-2384."

757 "刘永贤, 胱抑素C在临床中的应用. 医学信息, 2013. 26(6): p. 528-529."

758 "刘章锁, 雷.A.周.A.乔.A.高.A.刘.A.吕.A.潘.A.刘.A., 糖尿病肾病患者Piwi相互作用RNA差异表达谱分析. 中华肾脏病杂志, 2023. 39(4)."

759 "刘振良, 胡立禄, and 向乾才, 胱抑素 C与高血压的关系. 心血管病学进展, 2014(5): p. 609-613."

760 "刘智和, NGAL,NSE,BDNF与2型糖尿病周围神经病变相关性研究. 2022."

761 "刘忠, 阿.阿.A.胡.A.谢.A.哈.A., 糖尿病患者的早期肾损伤指标. 中国老年学杂志, 2011. 31(22)."

762 "龙俊宏, 徐.A.贾.A.张.A.王.A.丁.A., 胱抑素C及其他标志物诊断糖尿病肾病效能的比较. 西安交通大学学报（医学版）, 2010. 31(4)."

763 "娄雪菡, 血清胱抑素 C 诊断早期急性肾损伤的价值分析. 河南外科学杂志, 2015(4): p. 86-87."

764 "卢登球, 林.A.梁.A., 糖尿病肾病早期血、尿胱抑素C测定的临床价值研究. 包头医学, 2015. 39(1)."

765 "卢志勇, et al., 血清胱抑素C（Cys-C）在新生儿缺氧缺血性脑病（HIE）肾功能评价中的价值. 实验与检验医学, 2016. 34(3): p. 352-354."

766 "芦烨, 陈愉, and 赵立, 多种生物标志物与社区获得性肺炎严重程度的相关性分析. 国际呼吸杂志, 2020. 40(19): p. 1486-1490."

767 "陆放, et al., 血清胱抑素C水平评估2型糖尿病患者肾病预后的价值. 中华检验医学杂志, 2023. 46(4): p. 375-384."

768 "陆雷群, 马晓英, and 陈玲, 胱抑素C和同型半胱氨酸与糖尿病肾病的相关性研究. 临床内科杂志, 2013. 30(4): p. 256-257."

769 "陆洋, 早期肾损伤标志物检验在诊断老年高血压肾损害中的临床意义. 中文科技期刊数据库（文摘版）医药卫生, 2021(4)."

770 "罗兵, et al., 血清肿瘤特异性生长因子水平在慢性肾脏病患者肾功能损害严重程度评估中的应用. 东南大学学报（医学版）, 2020. 39(2): p. 145-150."

771 罗光成. 人附睾分泌蛋白4对糖尿病肾病的诊断价值. 2017.

772 "罗红艳, et al., 慢性阻塞性肺疾病患者早期肾损害的生物标志物评价. 宁夏医科大学学报, 2013. 35(6): p. 626-630."

773 "罗佳鑫, DTI用于紫癜性肾炎评价的研究. 2021."

774 "罗丽娅, 周力, and 陈晓琴, 血清胱抑素C对肝硬化患者肾功能损害的诊断价值. 贵州医药, 2013. 37(1): p. 26-27."

775 "罗萍, et al., 相关生物标志物在糖尿病肾病早期诊断的临床意义. 检验医学与临床, 2017. 14(14): p. 2082-2085."

776 "罗文辉 and 郑虹, 肝移植术后急性肾损伤与诊断新指标. 医学综述, 2011. 17(21): p. 3303-3306."

777 "罗秀英, 季.A., 血浆标志物与慢性心力衰竭患者急性心力衰竭发作及肾功能异常的关系研究. 浙江医学, 2016. 38(12)."

778 "吕波, 王.A.张.A.刘.A., 2型糖尿病早期肾病患者血清胱抑素C水平观察. 中国疗养医学, 2010. 19(6)."

779 "吕磊, 殷.A., 血清胱抑素对老年高血压早期肾功能损害的诊断价值. 山东医药, 2012. 52(1)."

780 "吕明珠, et al., 血清与尿硫氧还原蛋白比值对儿童急性肾盂肾炎诊断及病情预测价值. 中国医师进修杂志, 2019. 42(12): p. 1076-1080."

781 "马安然 and 费素娟, 血清胱抑素C对结直肠组织良恶性病变诊断价值. 中国中西医结合消化杂志, 2019. 27(2): p. 139-142,146."

782 "马聪, et al., 血清胱抑素C作为新型肾功指标在狼疮肾炎中的临床意义. 现代生物医学进展, 2020. 20(16): p. 3050-3056."

783 "马进, 夏海平, and 马瑞, 急性脑梗死患者血浆内皮损伤标志物的变化及其临床意义. 中国老年学杂志, 2016. 36(23): p. 5851-5853."

784 "马丽明 and 杨丽丽, 高血压肾损伤患者外周血FIB、FDP、CysC水平变化及意义. 齐齐哈尔医学院学报, 2021. 42(10): p. 841-843."

785 "马瑞, 马.A.夏.A., 急性脑梗死患者血浆内皮损伤标志物的变化及其临床意义. 中国老年学杂志, 2016. 36(23)."

786 "马珍 and 杨朝美, 血清α1-MG和NGAL在狼疮肾炎早期诊断中的临床价值. 国际检验医学杂志, 2022. 43(23): p. 2933-2936."

787 "梅娥, 吕.A.陈.A.查.A., 血清与尿硫氧还原蛋白比值对儿童急性肾盂肾炎诊断及病情预测价值. 中国医师进修杂志, 2019. 42(12)."

788 "蒙志平, 血清胱抑素C在糖尿病伴有肾脏损害患者检测中的作用. 中国现代药物应用, 2009. 03(17): p. 61-62."

789 "明兰, 李.A.刘.A., 血清视黄醇结合蛋白和胱抑素C在2型糖尿病早期肾功能损害中的应用. 检验医学与临床, 2014. 11(19)."

790 "穆恩, 吕黎新, and 武子霞, 生物学标志物在心功能衰竭早期肾损伤中的诊断价值. 中国中西医结合急救杂志, 2016. 23(5): p. 545-546."

791 "倪兆慧, 张.A.王.A.何.A.王.A.邱.A.郑.A.杨.A.庞.A.沈.A.牟.A., 生物标志物NGAL、NAG、胱抑素C诊断对比剂急性肾损伤的价值. 兰州大学学报（医学版）, 2018. 44(1)."

792 "牛庆慧, 钟.A., 乙肝肝硬化、慢性乙肝患者相关生化指标差异对比研究. 临床医学进展, 2023. 13(11)."

793 "彭海英, 宗先旭, and 裴莉, 血清胱抑素 C和尿微量白蛋白联合检测在糖尿病患者早期发现肾损伤中的诊断价值. 临床军医杂志, 2015(6): p. 648-649."

794 "钱磊, 血清胱抑素C检测用于2型糖尿病肾病早期诊断. 实用医药杂志, 2014. 31(12)."

795 "秦安东, et al., 血清胱抑素C检测对肝硬化患者肾功能损害的诊断价值. 国际检验医学杂志, 2013. 34(19): p. 2617-2619."

796 饶显群. 慢性阻塞性肺疾病合并肺动脉高压的生物标志物及其意义. 2018.

797 "任静, 血浆内脂素与2型糖尿病及相关肾病的关系研究. 2014."

798 "任苗, 贺.A., 早期肾损伤标志物对老年高血压肾损害的诊断价值. 慢性病学杂志, 2019. 0(9)."

799 "任苗苗 and 吴坚, 糖尿病肾病早期诊断标志物与中医辨证分型的关系. 中医学报, 2017. 32(7): p. 1175-1178."

800 "阮红刚, 付潮泓, and 许腊梅, 肾出血热各阶段血清RBP、β2-MG、 Cys-C水平变化及其临床意义. 内科急危重症杂志, 2019. 25(2): p. 144-145."

801 沙宇毅. 急性肾损伤新型早期诊断标志物研究及评价. 2014.

802 "尚健静, 赵.A.牛.A.张.A., 肝硬化并发肾功能损害患者血清胱抑素C与肾功能指标相关性分析. 中国基层医药, 2014. 21(11)."

803 "沈剑媛, 邓.A.龙.A.卢.A.邓.A., 血清胱抑素C及尿微量白蛋白与尿肌酐比值在糖尿病肾病诊断中的应用. 医学检验与临床, 2017. 28(1)."

804 "沈立婷, 血清胱抑素C与终末期肾病患者心脑血管并发症的相关性研究. 2014."

805 "沈薇薇, et al., MPV、PDW、铁蛋白在评估狼疮性肾炎患者肾损伤程度中的价值. 检验医学, 2020. 35(1): p. 33-36."

806 "沈薇薇, et al., 狼疮性肾炎患者血清胆红素水平的变化及意义. 临床输血与检验, 2020. 22(5): p. 533-537."

807 "盛西陵, et al., 血清NGAL和Cys-C联合检测对急诊PCI术后造影剂相关急性肾功能损伤的诊断价值. 浙江中西医结合杂志, 2015(7): p. 662-664,665."

808 "师小娜 and 张碧丽, 生物学标志物联合检测在儿童尿路感染定位诊断中的意义. 中华实用儿科临床杂志, 2013. 28(5): p. 357-360."

809 "时慧 and 王加平, 血清SCC、CEA、CysC、IL-8联合检测在肺癌诊断中的价值. 医药前沿, 2016. 6(30): p. 180-181."

810 "宋培, 3种血清标志物在尿毒症合并心力衰竭诊断中的应用价值. 国际检验医学杂志, 2019. 40(20): p. 2475-2478."

811 "宋文炜, 血清胱抑素C联合β2-微球蛋白检测对妊娠期糖尿病早期肾损伤的临床价值研究. 国际检验医学杂志, 2016. 37(22): p. 3134-3136."

812 "宋醒良, et al., 应用操作特征曲线分析生物学标志物在慢性心力衰竭中的价值. 当代医学, 2019. 25(7): p. 3-6."

813 "苏晓梅, et al., 检测尿微量白蛋白与血清胱抑素C对糖尿病早期肾功能损伤的临床探讨. 按摩与康复医学（下旬刊）, 2011. 02(7): p. 49-50."

814 "孙美娜, 高血压合并急性冠脉综合征患者血清Adropin变化的研究. 2016."

815 "孙琦, 常见血液指标在阿尔茨海默病及相关疾病中的变化. 2019."

816 "孙倩倩 and 谈敏, 胱抑素C在糖尿病肾病肾功能评估中的地位. 国外医学（老年医学分册）, 2009. 30(6): p. 266-269."

817 "孙青雯, 血清和肽素水平测定在心力衰竭患者诊断和预后评价中的价值. 2014."

818 "孙欣欣 and 李荣山, 生物学标志物在Ⅰ型心肾综合征急性肾损伤早期诊断中的应用. 国际移植与血液净化杂志, 2015(6): p. 19-22."

819 "覃兰, 李.A., 胱抑素-C在肾脏疾病中的应用价值. 右江医学, 2007. 35(1)."

820 "谭灵灿 and 朱涛, 肝移植术后急性肾损伤的早期诊断. 国际麻醉学与复苏杂志, 2017. 38(10): p. 938-942."

821 "谭同均, et al., Cystatin C在糖尿病肾病中的应用评价. 中国现代医药杂志, 2008. 10(1): p. 60-62."

822 "汤天凤, et al., 肾小管损伤标志物在局灶节段性肾小球硬化患者中的变化及意义. 肾脏病与透析肾移植杂志, 2010. 19(4): p. 317-323."

823 "唐国建, 葛.A.吴.A.祁.A., 胃癌患者手术治疗前后血清CEA、IL-8和Cys C检测的临床意义. 放射免疫学杂志, 2011. 24(4)."

824 "唐劲松, 卢健坤, and 王嘉贤, Cys-C、NGAL及KIM-1对妊娠期糖尿病早期肾损伤的诊断价值. 中国实用医刊, 2017. 44(23): p. 7-9."

825 "唐雪敏, et al., 尿视黄醇结合蛋白与血胱抑素C在妊娠高血压疾病早期肾损害中的诊断意义. 国际移植与血液净化杂志, 2017. 15(6): p. 29-31."

826 "万小健, 陈辉, and 朱科明. 胱抑素C及炎症标志物对脓毒症急性肾损伤患者预后的诊断价值. in 第十五次长江流域麻醉学学术年会暨2010年中南六省麻醉学学术年会暨2010年湖北省麻醉学学术年会. 2010. 中国湖北武汉."

827 万小健. 胱抑素C及炎症标志物对脓毒症急性肾损伤患者预后的诊断价值. 2010.

828 "汪丹丹, 胱抑素C在早期糖尿病肾病中的临床应用. 2015."

829 "汪润, et al., 左心室射血分数保留性心力衰竭患者预后相关生物靶向标志物的表达特征. 岭南心血管病杂志, 2020. 26(2): p. 190-194."

830 "汪涛, 李.A.李.A.张.A.简.A., 夜间高血压对原发性高血压患者早期肾脏损害的影响. 中国循环杂志, 2015. 30(7)."

831 "王兵, 杨.A.王.A.高.A., 尿胰岛素样生长因子结合蛋白7对脓毒症所致急性肾损伤的早期诊断价值. 中华急诊医学杂志, 2017. 26(9)."

832 "王峰, et al., 脑脊液胱抑素C和血清降钙素原检测在化脓性和病毒性脑膜炎鉴别诊断中的意义. 现代实用医学, 2014. 26(12): p. 1472-1473."

833 "王钢, NAG、CYSC、ET-1、RBP联合检测对早期糖尿病肾病的诊断价值. 2011."

834 "王海龙, 李.A.范.A.汪.A.常.A.马.A.陈.A.杨.A.李.A.徐.A., 2型糖尿病和糖尿病肾病患者血清微小RNA-148b-3p的水平变化及意义. 中华肾脏病杂志, 2018. 34(5)."

835 "王豪言, 首次部分缓解的多发性骨髓瘤患者中性粒细胞/淋巴细胞比值、单核细胞/淋巴细胞比值的预后意义. 2023."

836 "王红练, 血清胱抑素C、β2-微球蛋白及传统标志物检测评价糖尿病肾功能损害的临床价值. 国际检验医学杂志, 2018. 39(20): p. 2528-2530."

837 "王晶 and 陈曦, 血清标志物Urea、Creat、CysC及NGAL检测在早期诊断糖尿病肾病中的应用价值. 当代医药论丛, 2020. 18(07): p. 1-2."

838 "王晶 and 陈曦, 血清标志物Urea、Great、CysC及NGAL检测在早期诊断糖尿病肾病中的应用价值. 当代医药论丛, 2020. 18(7): p. 1-2."

839 "王晶, 血清胆红素与糖尿病肾病及全身炎症标志物的相关性研究. 2022."

840 "王婧, 糖尿病肾病血清胱抑素C、尿转铁蛋白与肾小球滤过率的相关性研究. 2013."

841 "王军强, 尿微量白蛋白和血清胱抑素C联合检测在糖尿病早期肾病中的临床价值. 饮食保健, 2020. 7(24): p. 57."

842 "王坤元, 急性肾损伤早期标志物诊断价值评价. 国际泌尿系统杂志, 2012. 32(1): p. 119-124."

843 "王露 and 樊启红, NGAL、KIM-1、Cys-C对早产儿急性肾损伤的诊断价值. 临床误诊误治, 2015(10): p. 107-109."

844 "王芊 and 华川, 肾损伤标志物在糖尿病肾病早期诊断中的应用. 解放军医药杂志, 2012. 24(02): p. 56-58."

845 "王清, 张.A.崔.A.温.A., 2型糖尿病肾病患者血清胱抑素C和同型半胱氨酸水平的检测及其临床意义. 吉林大学学报（医学版）, 2015. 41(5)."

846 "王清平, 顾建文, and 黄健伟, 胱抑素C对评估肝硬化患者肾小球滤过率的价值. 临床和实验医学杂志, 2006. 5(12): p. 1891-1893."

847 "王清平, 顾建文, and 黄健伟, 血清胱抑素C检测对肝肾综合征的诊断价值. 中国实验诊断学, 2007. 11(12): p. 1644-1646."

848 "王珊 and 甄明慧, 多种生物标志物联合检测对儿童脓毒症相关急性肾损伤的诊断价值. 中国疗养医学, 2021. 30(01): p. 96-98."

849 "王世明, 王.A.李.A.刘.A., 急性胰腺炎发生急性肾损伤的早期预测指标. 临床肝胆病杂志, 2022. 38(5)."

850 "王淑敏, 胱抑素C与尿微量白蛋白检测对糖尿病早期肾损伤的临床意义. 中国保健, 2008. 16(24): p. 1284-1285."

851 "王术艺, 沈.A.李.A.卜.A.金.A.陈.A.杨.A., MPV、PDW、铁蛋白在评估狼疮性肾炎患者肾损伤程度中的价值. 检验医学, 2020. 35(1)."

852 "王霜, HMGB1和sRAGE在社区获得性肺炎中的作用研究. 2022."

853 "王婷, 罗.A.毛.A.汪.A.颜.A., 相关生物标志物在糖尿病肾病早期诊断的临床意义. 检验医学与临床, 2017. 14(14)."

854 "王卫东, et al., 尿诱骗受体2/肌酐水平与糖尿病肾小管病预后的关系. 中华肾病研究电子杂志, 2023. 12(2): p. 61-66."

855 "王卫东, 尿DcR2/Cr水平与糖尿病肾小管病不良预后的关系*. 2023."

856 "王文潇, 基于CiteSpaceV脓毒症急性肾损伤研究的可视化分析. 2020."

857 "王小芳, et al., 尿NGAL检测对急性肾小球肾炎早期肾损伤诊断的临床意义. 家庭医药·就医选药, 2020(11): p. 78-79."

858 "王小刚, 曾.A.李.A.谈.A., 尿肝型脂肪酸结合蛋白与糖尿病肾损伤的相关性. 现代检验医学杂志, 2013. 28(1)."

859 "王兴亮, 秦.A.曾.A.刘.A., 血清胱抑素C检测对肝硬化患者肾功能损害的诊断价值. 国际检验医学杂志, 2013. 34(19)."

860 "王亚平, et al., 胱抑素C在糖尿病肾病早期诊断中的应用. 临床检验杂志, 2006. 24(3): p. 240-240."

861 "王垚, 孙滨, and 杨明, 血清胱抑素C在糖尿病肾病诊断中的临床意义. 中国民康医学, 2011. 23(15): p. 1844-1845."

862 "王兆星, et al., 老年患者注射造影剂后肾损伤指标的动态变化. 临床和实验医学杂志, 2016(3): p. 296-299."

863 "王兆星, 造影剂肾病早期诊断的生物标志物. 肾脏病与透析肾移植杂志, 2011. 20(3): p. 275-279."

864 "韦慧玲, Cys-C在儿童急性肾损伤病情评估中的应用价值分析. 社区医学杂志, 2015. 13(22): p. 56-57."

865 "韦兰区, et al., 慢性阻塞性肺病急性加重期联合检测胱抑素C、前白蛋白和降钙素原的临床效果分析. 医药前沿, 2018. 8(28): p. 21-23."

866 "韦薇, 血清生物标志物对脑梗死超早期患者重组组织型纤溶酶原激活剂静脉溶栓预后预测价值的研究. 2019."

867 "魏炯, 何.A.刘.A., 外周血Th17细胞对脓毒症急性肾损伤的早期诊断价值. 检验医学与临床, 2018. 15(11)."

868 "魏琰, 高.A.王.A.孟.A.李.A.崔.A., 急性脑梗死患者血清微小RNA-145、程序性细胞死亡因子4mRNA水平变化及诊断价值研究. 实用心脑肺血管病杂志, 2022. 30(2)."

869 "温洁新, 阿尔茨海默病和轻度认知障碍患者血尿生物标志物的筛选. 2014."

870 "吴波 and 杜强, 血清胱抑素C对早期糖尿病肾损伤的诊断价值. 中国老年学杂志, 2011. 31(8): p. 1464-1465."

871 "吴浩能, 崔.A.赵.A.黄.A., 血清胱抑素C和尿NAG联合检测在糖尿病肾病早期诊断中的应用. 实用检验医师杂志, 2023. 15(4)."

872 "吴惠毅, 张.A.赵.A.郑.A.杨.A.杨.A.张.A.马.A.陈.A., β痕迹蛋白在老年糖尿病患者肾损害评估中应用的观察. 中国糖尿病杂志, 2012. 20(12)."

873 "吴家玉, et al., 血清胱抑素C和尿NGAL联合检测在急性肾损伤病情评估及预后中的价值. 中山大学学报（医学科学版）, 2014. 35(1): p. 152-155,封3."

874 "吴坚, 任.A., 糖尿病肾病早期诊断标志物与中医辨证分型的关系. 中医学报, 2017. 32(7)."

875 "吴建芳, 滕.A., 尿足细胞标志蛋白、血清胱抑素和高敏C反应蛋白联合检测诊断早期糖尿病肾病的价值. 现代中西医结合杂志, 2012. 21(1)."

876 "吴坤利, 吴.A., 胱抑素C和尿微量白蛋白联合检测对诊断糖尿病早期肾损伤的价值. 中国医药指南, 2010. 8(25)."

877 "吴磊, 血浆D-二聚体、纤维蛋白原、Hcy水平及Willis环不完整性与偏头痛的相关性研究. 2022."

878 "吴颖, et al., 腹腔镜保留肾单位手术后急性肾损伤的危险因素分析及临床意义. 中华全科医学, 2023. 21(9): p. 1457-1460."

879 "吴永国, et al., 尿微量清蛋白联合血清胱抑素在早期糖尿病肾病诊断中的应用. 国际医药卫生导报, 2016. 22(15): p. 2346-2348."

880 "伍利利, 唐恩燕, and 石亮, 血小板与淋巴细胞比值预测肝硬化患者发生急性肾损伤的价值分析. 中国卫生检验杂志, 2019. 29(15): p. 1802-1804,1808."

881 "武鹏 and 周婧婷, Cysc一种新的肾疾病诊断标志物应用研究. 健康之路, 2013. 12(11): p. 44."

882 "武晓静, 靳.A.郑.A.顾.A.赖.A., 左心疾病相关性肺动脉高压患者血清生化标志物的特征. 中国循环杂志, 2016. 31(4)."

883 "武子霞, 穆.A.吕.A., 生物学标志物在心功能衰竭早期肾损伤中的诊断价值. 中国中西医结合急救杂志, 2016. 23(5)."

884 "向瑞, 血清Cys--C、RBP、NGAL在糖尿病肾病早期诊断中的应用价值. 2020."

885 "项国谦, et al., 血清胱抑素C在糖尿病肾病早期诊断中的临床价值. 中国中西医结合肾病杂志, 2011. 12(10): p. 901-902."

886 "肖梦云, 潘明娇, and 涂晓文, 血清胱抑素C对早期糖尿病肾病肾小球损害的诊断价值. 武警医学, 2018. 29(1): p. 35-37,41."

887 "肖文霞, 糖尿病肾病早期肾脏MR功能成像与尿蛋白排泄率的比较研究. 2012."

888 "肖筱婵, Cys--C与动脉粥样硬化型脑梗死的相关性分析. 2020."

889 "肖筱婵, et al., 血清标志物对动脉粥样硬化脑梗死的临床价值. 广东医学, 2020. 41(15): p. 1616-1621."

890 "谢连志, et al., 肾损伤标志物在儿童过敏性紫癜性肾炎中的应用价值比较. 医学理论与实践, 2018. 31(13): p. 1899-1900,1904."

891 "谢龙, 老年心衰患者血清可溶性ST2的临床意义及其与心功能关系的研究. 2020."

892 "谢能轩, et al., 血清胱抑素C和β2-微球蛋白在糖尿病肾病诊断中的临床意义. 中国实验诊断学, 2012. 16(3): p. 441-443."

893 "邢昌赢, 陆.A.张.A.段.A.袁.A.张.A.毛.A., 血清胱抑素C水平评估2型糖尿病患者肾病预后的价值. 中华检验医学杂志, 2023. 46(4)."

894 "邢志洁, Connexin43在单纯性肥胖儿童尿外泌体中的表达变化及临床意义. 2020."

895 "熊锋莉 and 黄晓光, Cys-C和β2-MG联合检测对肾病综合征出血热诊断和治疗的意义. 基层医学论坛, 2014(26): p. 3550-3551."

896 "熊声贺, et al., 高龄慢性肾脏病人群血清拉曼光谱评价体系建立. 国际检验医学杂志, 2018. 39(14): p. 1744-1748."

897 "徐飞, DD捐献儿童移植肾功能延迟恢复的相关研究. 2019."

898 "徐静, et al., 胱抑素C及其他标志物诊断糖尿病肾病效能的比较. 西安交通大学学报(医学版), 2010. 31(04): p. 481-483."

899 "徐力 and 王会琴, 早期糖尿病肾病患者血清胱抑素C及其他标志物与尿微量白蛋白相关性探讨. 中国实用医刊, 2011. 38(15): p. 119-121."

900 "徐丽霞, 刘五高, and 钟益芳, 血清AFP、CA19-9、Hcy、CysC、和SE-CAD联检对原发性肝癌的诊断价值. 放射免疫学杂志, 2013. 26(3): p. 287-288."

901 "徐明伟, 徐建东, and 吴善玲, 血清胱抑素C在糖尿病及高血压早期肾损害中的临床意义. 甘肃医药, 2012. 31(11): p. 821-823."

902 "徐尚福, 黄.A.廖.A.王.A., 2型糖尿病肾病患者血清趋化素与胱抑素C水平的相关性. 成都医学院学报, 2019. 14(6)."

903 "徐晓薇, 赖.A.姜.A., 血清胱抑素C、肿瘤坏死因子-α水平对早期糖尿病肾病的诊断价值. 中外医学研究, 2023. 21(29)."

904 "许慧慧, et al., 老年脓毒症诱发急性肾损伤患者血清NGAL、CysC水平变化及诊断价值研究. 中国实验诊断学, 2020. 24(7): p. 1138-1140."

905 "许腊梅, 阮.A.付.A., 肾出血热各阶段血清RBP、β_2-MG、Cys-C水平变化及其临床意义. 内科急危重症杂志, 2019. 25(2)."

906 "许琴, 胱抑素C在糖尿病肾病早期诊断中的预测价值. 2020."

907 "许颖, et al., 唾液中差异性蛋白在2型糖尿病早期诊断中的临床研究. 成都医学院学报, 2016. 11(3): p. 292-296."

908 许颖. 唾液中差异性蛋白在2型糖尿病早期诊断中的临床研究. 2016.

909 "许月仙, 血清同型半胱氨酸、胱抑素C在诊断糖尿病肾病中的诊断价值分析. 糖尿病新世界, 2022. 25(1): p. 184-186,190."

910 "闫大志, et al., 以低血糖昏迷为表现的2型糖尿病1例. 中外健康文摘, 2013(21): p. 179-180."

911 "严华, 何.A.符.A., 多种生化指标联合检测对高血压肾损害早期诊断的价值. 中国临床新医学, 2012. 5(7)."

912 "颜建华, 胱抑素C尿微量清蛋白糖化血红蛋白检测对糖尿病肾病早期诊断的意义. 现代医药卫生, 2012. 28(2): p. 234-235."

913 "杨才, et al., 血清胱抑素C、尿微量清蛋白对糖尿病患者肾功能早期损伤诊断的临床应用. 中国社区医师, 2018. 34(13): p. 125,127."

914 "杨朝美, 马.A., 血清α1-MG和NGAL在狼疮肾炎早期诊断中的临床价值. 国际检验医学杂志, 2022. 43(23)."

915 "杨改清, 官.A.徐.A.李.A.胥.A.孙.A.王.A., 老年动脉粥样硬化性脑梗死患者血管重构与血清胱抑素C水平的关系研究. 中华老年心脑血管病杂志, 2021. 23(5)."

916 "杨洪芬, et al., 胱抑素在老年2型糖尿病肾功能损害评价中的临床应用. 中国老年学杂志, 2013. 33(3): p. 669-670."

917 "杨敬慈, 李.A.赵.A.付.A., 血小板活化标志物CD62P,CD63与血清胱抑素C 联合检测在糖尿病肾病早期诊断的临床研究. 现代检验医学杂志, 2013. 28(1)."

918 "杨礼彬 and 何光晏, 高敏肌钙蛋白T和胱抑素联合测定在糖尿病肾病并发急性心肌梗死临床应用. 心血管病防治知识, 2018(1): p. 66-68."

919 "杨立业, 陈.A.黎.A.杨.A.李.A., 睾丸素-2在糖尿病肾损伤中的诊断价值. 检验医学与临床, 2023. 20(7)."

920 "杨丽丽, 马.A., 高血压肾损伤患者外周血FIB、FDP、CysC水平变化及意义. 齐齐哈尔医学院学报, 2021. 42(10)."

921 "杨平金, 血清胱抑素C联合尿微量白蛋白检验对糖尿病患者早期肾功能损伤的诊断价值. 中文科技期刊数据库（引文版）医药卫生, 2023(4)."

922 "杨荣礼, 徐.A.李.A.高.A.卢.A.杨.A., 老年糖尿病肾病病人血清6项指标检测水平与尿微量白蛋白的关系. 安徽医药, 2021. 25(11)."

923 "杨瑞琦, BNP、CysC、NLR与AECOPD并肺动脉高压的相关性研究. 2023."

924 "杨书英, et al., 尿胰岛素样生长因子结合蛋白7对脓毒症所致急性肾损伤的早期诊断价值. 中华急诊医学杂志, 2017. 26(9): p. 1010-1014."

925 "杨蔚洁, et al., 血清胱抑素C诊断早期糖尿病肾病肾损害. 中国临床医学, 2008. 15(2): p. 223-224."

926 "杨晓萍, 王.A.潘.A.唐.A.赵.A.黄.A., 血清心型脂肪酸结合蛋白在急性肾损伤诊断中的临床意义. 临床肾脏病杂志, 2018. 18(4)."

927 "杨晓英, 基于CHARLS数据分析社区中老年人胱抑素--C与慢性病及肌酐/胱抑素--C与失能的关联. 2023."

928 "杨亚超, et al., 脑出血并发脑微出血患者的肾脏损伤标志物研究. 中华神经科杂志, 2015. 48(6): p. 469-474."

929 "杨彦民, 张.A., 诊断早期肾损伤的标志物. 中国社区医师（医学专业）, 2009. 11(14)."

930 "杨渝伟, et al., 胱抑素C及其eGFR方程在诊断肝硬化患者肾损害中的应用. 现代检验医学杂志, 2016. 31(4): p. 24-29."

931 "杨渝伟, et al., 肾损伤、脂质异常与清蛋白尿糖尿病肾病及非清蛋白尿糖尿病肾病发生的关系. 国际检验医学杂志, 2020. 41(16): p. 1944-1949,1953."

932 "杨征, 吴静宇, and 凌爱华, 血清同型半胱氨酸与血清胱抑素C联合检测在社区筛查早期糖尿病肾病中的应用. 糖尿病新世界, 2019. 22(2): p. 57-60."

933 "姚迪, et al., Cystatinc在早期诊断糖尿病肾损害中的意义. 中国实验诊断学, 2009. 13(10): p. 1389-1390."

934 "姚桂凤, 血清胱抑素C及β2微球蛋白在2型糖尿病患者早期肾损害中的临床应用价值. 2021."

935 "姚美娟, et al., 血、尿5项指标对初诊2型糖尿病患者早期肾功能损伤的诊断价值. 临床和实验医学杂志, 2012. 11(24): p. 1926-1927,1930."

936 "叶辉, 早期糖尿病肾病尿podocalyxin阳性细胞或/和细胞碎片与临床诊断的关系. 2013."

937 "依力夏提·依麻木 and 再努拉·阿不都艾尼, 血清胱抑素C早期诊断糖尿病肾病的价值. 右江医学, 2015. 43(6): p. 688-690."

938 "殷宇刚 and 吕磊, 血清胱抑素对老年高血压早期肾功能损害的诊断价值. 山东医药, 2012. 52(1): p. 79-80."

939 "银芳颖, 荧光层析法肾功能损伤标志物Cys C、ALB无创检测技术方法的建立. 2019."

940 "于洋, B型脑钠肽、胱抑素C在预测心脏瓣膜置换术后急性肾损伤发生的作用及临床意义. 2021."

941 "余盛武, 基于血清生物标志物的老年髋部骨折患者肌少症预测模型研究. 2022."

942 "郁爱平, 瞿.A., N末端脑利钠肽前体与胱抑素C对慢性心衰的诊断价值. 现代消化及介入诊疗, 2019. 24(A02)."

943 "袁学超, 经皮冠状动脉介入治疗患者高敏C反应蛋白及降钙素原对造影剂肾病的预测价值. 2017."

944 "张碧丽, 师.A., 生物学标志物联合检测在儿童尿路感染定位诊断中的意义. 中华实用儿科临床杂志, 2013. 28(5)."

945 "张彩香, et al., 高尿酸血症患者血清胱抑素 C与肾小球滤过率的相关性研究. 中国糖尿病杂志, 2016. 24(7): p. 594-597."

946 "张冬晓, FetuiN-A作为疾病标志物对糖尿病肾病的意义. 2016."

947 "张欢欢, et al., β痕迹蛋白在老年糖尿病患者肾损害评估中应用的观察. 中国糖尿病杂志, 2012. 20(12): p. 922-924."

948 "张会芬, et al., 血清中性粒细胞明胶酶相关脂质运载蛋白、胱抑素C及尿白蛋白肌酐比值、N-乙酰-β-D-氨基葡萄糖苷酶检测在早期糖尿病肾脏疾病中的应用价值. 中国糖尿病杂志, 2018. 26(4): p. 309-315."

949 "张建国, et al., NGAL在脓毒症性肾损伤早期诊断中的临床价值. 中国实用医药, 2015. 10(24): p. 46-47."

950 "张凯聪, 狼疮性肾炎的临床特征及血浆非对称性二甲基精氨酸的意义研究. 2019."

951 "张蕾, 血清胱抑素C评价肿瘤患者急性肾损伤预后的相关性分析. 医药论坛杂志, 2022. 43(3): p. 48-51,55."

952 "张立娟, 孙树荣, and 武佳蕾, 血清胱抑素 C 在老年性急性肾损伤患者中早期诊断价值的研究. 检验医学与临床, 2016. 13(9): p. 1158-1160."

953 "张敏, 张.A.邹.A., 高血压肾病患者血清NGAL与Cys-c水平探讨. 中国卫生检验杂志, 2013. 23(9)."

954 "张敏敏, 中轴型脊柱关节炎患者血肿瘤标志物异常的临床意义. 2020."

955 "张鹏, 长期应用阿德福韦酯对慢性乙型肝炎患者早期肾脏损害的临床研究. 2017."

956 "张清平, 邹长进, and 张敏, 高血压肾病患者血清NGAL与Cys-c水平探讨. 中国卫生检验杂志, 2013. 23(9): p. 2203-2204."

957 "张秋梅, 曾.A.蒋.A., RBP、CysC、sICAM-1、u-MALB联合检测对早期糖尿病肾病的诊断价值. 热带医学杂志, 2019. 19(1)."

958 "张世先, 宫.A.杨.A., 急性肾损伤相关生物学标志物在重症监护病房患者中的应用价值. 中南大学学报（医学版）, 2015. 40(10)."

959 "张晓菊, et al., 多生物标志物检测在中西医结合治疗糖尿病肾病中的临床应用. 山西医药杂志, 2019. 48(19): p. 2343-2346."

960 "张研, et al., 2型糖尿病肾病患者血清胱抑素C和同型半胱氨酸水平的检测及其临床意义. 吉林大学学报（医学版）, 2015. 41(5): p. 998-1003."

961 "张衍胜 and 王洋, 血清胱抑素C和尿微量白蛋白检测对糖尿病早期肾损伤的诊断价值. 实用临床医学, 2012. 13(2): p. 13-14."

962 "张瑜, 刘.A.扈.A.张.A.李.A., 糖尿病肾病患者血浆NGAL和血清CysC水平改变及其早期诊断价值. 现代生物医学进展, 2015. 15(30)."

963 "张宇, 基于RT--3DE技术探讨肝豆扶木颗粒干预肝肾亏虚型Wilson病患者心脏损害的临床研究. 2023."

964 "章霞, 血清Cys C是否是评估冠心病患者eGFR的简易敏感指标. 2014."

965 "赵传燕, et al., 血清胃泌素前体释放肽在慢性肾脏病中的临床意义与诊断价值. 实用临床医药杂志, 2022. 26(5): p. 62-65,70."

966 "赵芳, et al., 肝硬化并发肾功能损害患者血清胱抑素C与肾功能指标相关性分析. 中国基层医药, 2014. 21(11): p. 1678-1680."

967 "赵枫, 王.A.王.A.李.A., 脑脊液胱抑素C和血清降钙素原检测在化脓性和病毒性脑膜炎鉴别诊断中的意义. 现代实用医学, 2014. 26(12)."

968 "赵洪灿, 项.A.陶.A.冯.A., 血清胱抑素C在糖尿病肾病早期诊断中的临床价值. 中国中西医结合肾病杂志, 2011. 12(10)."

969 "赵慧 and 陈苓, 胱抑素C和炎症因子在脓毒症致急性肾损伤儿童中的临床价值. 中国妇幼健康研究, 2016. 27(8): p. 965-967."

970 "赵立科, 病理性黄疸早产儿血清胱抑素C与急性肾损伤的相关性研究. 2021."

971 "赵丽菲 and 赵威, 干化学法检测尿微清蛋白作为早期肾脏损伤初筛实验的应用价值. 检验医学与临床, 2014(z1): p. 149-150."

972 赵良. 急性肾损伤的早期诊断. 2010.

973 赵良. 急性肾损伤的早期诊断. 2011.

974 "赵娜, 陈.A.赵.A., 血清Sestrin2与胱抑素C在缺血性脑卒中早期诊断中的临床价值. 健康研究, 2020. 40(6)."

975 "赵娜, 血清胱抑素 C、表皮生长因子、血管细胞黏附分子-1在儿童紫癜性肾炎诊断中的价值. 中国临床医生杂志, 2016. 44(12): p. 48-50."

976 "赵鹏, et al., 脓毒症合并急性肾损伤患者生物学标志物检测及其与肾动脉阻力指数的关系. 临床和实验医学杂志, 2019. 18(7): p. 730-733."

977 "赵庆, 张茂平教授学术思想及治疗糖尿病肾病经验的临床研究. 2016."

978 "赵杨 and 张泓, 动脉血气指标与老年百草枯中毒患者肾损伤程度的相关性. 中国老年学杂志, 2020. 40(13): p. 2824-2827."

979 "赵英, 林成芳, and 杨劲, 胱抑素C及α1-微球蛋白检测对糖尿病肾病诊断价值的比较分析. 国际检验医学杂志, 2010. 31(9): p. 935-936,938."

980 "赵迎新, 冠脉造影剂肾损伤早期诊断研究. 2018."

981 "赵岳, 早期甲状腺功能失调对血清胱抑素C水平的影响. 2017."

982 "甄明慧, 王.A., 多种生物标志物联合检测对儿童脓毒症相关急性肾损伤的诊断价值. 中国疗养医学, 2021. 30(1)."

983 "郑兵, 血清胱抑素C与β2-微球蛋白对糖尿病早期肾损伤的价值分析. 中国误诊学杂志, 2010. 10(10): p. 2301-2302."

984 "郑国军, et al., 中性粒细胞明胶酶相关脂蛋白和胱抑素C作为糖尿病肾病早期标志物的评价. 广东医学, 2019. 40(23): p. 3285-3289."

985 "郑虹, 罗.A., 肝移植术后急性肾损伤与诊断新指标. 医学综述, 2011. 17(21)."

986 "郑群, 刘.A.赵.A.牟.A.扈.A., 血清胱抑素C与同型半胱氨酸联合检测对高血压早期肾病的诊断价值. 国际检验医学杂志, 2017. 38(21)."

987 "郑湘毅, 何喜子, and 梁素娟, 血β2微球蛋白、血清胱抑素C及其他标志物的检测在高血压病肾损害诊断中的意义. 医学临床研究, 2011. 28(11): p. 2060-2063."

988 "郑亚莉, 罗.A.兰.A.曹.A., 慢性阻塞性肺疾病患者早期肾损害的生物标志物评价. 宁夏医科大学学报, 2013. 35(6)."

989 "郑艳宇, 早期诊断标志物在糖尿病肾病临床诊断中的应用. 健康女性, 2022(3): p. 75-77."

990 "钟赟, 多发性骨髓瘤肾损害的早期评估. 2012."

991 "仲信明 and 夏爱华, 血清胱抑素C在诊断糖尿病肾病中的相关性研究. 医学检验与临床, 2008. 19(4): p. 49-50."

992 "周春华, et al., 血清胱抑素C在模拟高强度一次性力竭军事训练大鼠早期肾损伤诊断中的作用. 解放军医学杂志, 2014. 39(8): p. 660-663."

993 "周华, 马.A.卢.A.栾.A.王.A.冉.A., 血清胱抑素C作为新型肾功指标在狼疮肾炎中的临床意义. 现代生物医学进展, 2020. 20(16)."

994 "周华胜, 胱抑素C在糖尿病性肾病中的探讨. 医学信息（下旬刊）, 2011. 24(6): p. 290-290."

995 "周青霞, 血管内皮生长因子及血清胱抑素C对糖尿病合并高血压的诊断价值. 实用临床医药杂志, 2016. 20(9): p. 168-169."

996 "周伟伟, et al., 心肌酶及胱抑素C水平对急性心肌梗死患者的临床诊断价值分析. 中外医疗, 2016. 35(12): p. 56-58."

997 "周晓萍, 许.A.石.A.吴.A., 唾液中差异性蛋白在2型糖尿病早期诊断中的临床研究. 成都医学院学报, 2016. 11(3)."

998 "周永年, Cys-C在肾脏疾病诊断中的应用与探讨. 医学信息, 2009. 22(12): p. 2760-2761."

999 "周悦昌, 陈丽萍, and 王玉华, 血清胱抑素C测定在2型糖尿病肾病早期诊断中的临床应用. 中国现代医药杂志, 2010. 12(5): p. 80-81."

1000 "朱广林, 稳定期慢阻肺患者膈肌功能超声评估与临床特征相关性分析及其临床意义. 2023."

1001 "朱名安, 王.A.彭.A., 冠心病合并代谢综合征患者固醇调节元件结合蛋白表达水平与脂代谢指标相关性研究. 陕西医学杂志, 2020. 49(4)."

1002 "朱清红, 张吉才, and 康敏, 生物标志物联合检测在早期糖尿病肾病诊断中的应用. 检验医学与临床, 2020. 17(02): p. 178-182."

1003 "朱涛, 谭.A., 肝移植术后急性肾损伤的早期诊断. 国际麻醉学与复苏杂志, 2017. 38(10)."

1004 "朱贤慧, 冠心病合并糖尿病阿司匹林抵抗中医证素研究及机制探讨. 2014."

1005 "朱晓峰 and 伍惠静, 胱抑素C心肌肌钙蛋白I和N末端-脑钠肽前体联合检测对老年心力衰竭的诊断价值. 山西医药杂志, 2019. 48(15): p. 1907-1909."

"Basic experiments, proteomics, other small molecules, drug trials, correlation research analysis, modeling(528)"

1 "Abdel-Hakeem, E.A., et al., Angiotensin 1–7 mitigates rhabdomyolysis induced renal injury in rats via modulation of TLR-4/NF-kB/iNOS and Nrf-2/heme?oxygenase-1 signaling pathways. Life Sciences, 2022. 303: p. 120678."

2 "Abdel-Wahab, B.A., et al., Febuxostat alleviates Arsenic Trioxide-Induced renal injury in Rats: Insights on the crosstalk between NLRP3/TLR4, Sirt-1/NF-κB/TGF-β signaling Pathways, and miR-23b-3p, miR-181a-5b expression. Biochemical Pharmacology, 2023. 216: p. 115794."

3 "Abdul-Hussien, H., et al., Doxycycline therapy for abdominal aneurysm: Improved proteolytic balance through reduced neutrophil content. J Vasc Surg, 2009. 49(3): p. 741-9."

4 "Abouelela, M.E., et al., Ethyl acetate extract of Ceiba pentandra (L.) Gaertn. reduces methotrexate-induced renal damage in rats via antioxidant, anti-inflammatory, and antiapoptotic actions. Journal of Traditional and Complementary Medicine, 2020. 10(5): p. 478-486."

5 "AbouEzzeddine, O.F., et al., Galectin-3 in heart failure with preserved ejection fraction. A RELAX trial substudy (Phosphodiesterase-5 Inhibition to Improve Clinical Status and Exercise Capacity in Diastolic Heart Failure). JACC Heart Fail, 2015. 3(3): p. 245-52."

6 "Aboyoussef, A.M., et al., Enoxaparin prevents CXCL16/ADAM10-mediated cisplatin renal toxicity: Role of the coagulation system and the transcriptional factor NF-κB. Life Sciences, 2021. 270: p. 119120."

7 "Abu-Saleh, N., et al., Increased Intra-abdominal Pressure Induces Acute Kidney Injury in an Experimental Model of Congestive Heart Failure. Journal of Cardiac Failure, 2019. 25(6): p. 468-478."

8 "Adolph, E., et al., Renal Insufficiency Following Radiocontrast Exposure Trial (REINFORCE): a randomized comparison of sodium bicarbonate versus sodium chloride hydration for the prevention of contrast-induced nephropathy. Coron Artery Dis, 2008. 19(6): p. 413-9."

9 "Ahn, S.B., et al., Use of a Recombinant Biomarker Protein DDA Library Increases DIA Coverage of Low Abundance Plasma Proteins. Journal of Proteome Research, 2021. 20(5): p. 2374-2389."

10 "Akkucuk, F.G., et al., The effect of HES (130/0.4) usage as the priming solution on renal function in children undergoing cardiac surgery. Ren Fail, 2013. 35(2): p. 210-5."

11 "Aldweib, N., et al., MELD-XI score is not associated with adverse outcomes in ambulatory adults with a Fontan circulation. International Journal of Cardiology Congenital Heart Disease, 2021. 4: p. 100182."

12 "Algin, H.I., et al., Which Mechanism is Effective on the Hyperamylasaemia After Coronary Artery Bypass Surgery? Heart Lung Circ, 2017. 26(5): p. 504-508."

13 "Al-Kindi, S.G., et al., Soluble CD14 and Risk of Heart Failure and Its Subtypes in Older Adults. Journal of Cardiac Failure, 2020. 26(5): p. 410-419."

14 "Aluru, S.V., et al., Tear Fluid Protein Changes in Dry Eye Syndrome Associated with Rheumatoid Arthritis: A Proteomic Approach. The Ocular Surface, 2017. 15(1): p. 112-129."

15 "Amarasiri, S.S., et al., Nephroprotective mechanisms of Ambrette (Abelmoschus moschatus Medik.) leaf extracts in adriamycin mediated acute kidney injury model of Wistar rats. Journal of Ethnopharmacology, 2022. 292: p. 115221."

16 "Anand, I.S., et al., Comparison of once-daily versus twice-daily dosing of valsartan in patients with chronic stable heart failure. Vasc Health Risk Manag, 2010. 6: p. 449-55."

17 "Ansquer, J.C., et al., Effect of fenofibrate on kidney function: a 6-week randomized crossover trial in healthy people. Am J Kidney Dis, 2008. 51(6): p. 904-13."

18 "António, M., et al., Label-free dynamic light scattering assay for C-reactive protein detection using magnetic nanoparticles. Analytica Chimica Acta, 2022. 1222: p. 340169."

19 "Aupy, P., et al., Identifying and Avoiding tcDNA-ASO Sequence-Specific Toxicity for the Development of DMD Exon 51 Skipping Therapy. Molecular Therapy - Nucleic Acids, 2020. 19: p. 371-383."

20 "Auwerx, C., et al., The individual and global impact of copy-number variants on complex human traits. The American Journal of Human Genetics, 2022. 109(4): p. 647-668."

21 "Aydo?an, H., et al., Adding 75mg pregabalin to analgesic regimen reduces pain scores and opioid consumption in adults following percutaneous nephrolithotomy. Brazilian Journal of Anesthesiology (English Edition), 2014. 64(5): p. 335-342."

22 "Azizi, Z., et al., Evidence for an association between allostatic load and multisensory integration in middle-aged and older adults. Archives of Gerontology and Geriatrics, 2024. 116: p. 105155."

23 "Babenko, B., et al., A deep learning model for novel systemic biomarkers in photographs of the external eye: a retrospective study. The Lancet Digital Health, 2023. 5(5): p. e257-e264."

24 "Baetta, R., et al., Proteomics in cardiovascular diseases: Unveiling sex and gender differences in the era of precision medicine. Journal of Proteomics, 2018. 173: p. 62-76."

25 "Bajaj, P., et al., Freshly isolated primary human proximal tubule cells as an in vitro model for the detection of renal tubular toxicity. Toxicology, 2020. 442: p. 152535."

26 "Bayes-Genis, A., et al., Soluble ST2 Serum Concentration and Renal Function in Heart?Failure. Journal of Cardiac Failure, 2013. 19(11): p. 768-775."

27 "Beasley, J.M., et al., Higher Biomarker-Calibrated Protein Intake Is Not Associated with Impaired Renal Function in Postmenopausal Women1,2. The Journal of Nutrition, 2011. 141(8): p. 1502-1507."

28 "Beck, H.C., M. Overgaard, and L. Melholt Rasmussen, Plasma proteomics to identify biomarkers – application to cardiovascular diseases. Translational Proteomics, 2015. 7: p. 40-48."

29 "Bellia, C., et al., Fetuin-A serum levels are not correlated to kidney function in long-lived subjects. Clinical Biochemistry, 2012. 45(9): p. 637-640."

30 "Bender, A., et al., Long-term creatine supplementation is safe in aged patients with Parkinson disease. Nutr Res, 2008. 28(3): p. 172-8."

31 "Bertinchant, J.P., et al., Evaluation of cardiac troponin I and T levels as markers of myocardial damage in doxorubicin-induced cardiomyopathy rats, and their relationship with echocardiographic and histological findings. Clin Chim Acta, 2003. 329(1-2): p. 39-51."

32 "Beunders, R., et al., Assessing GFR With Proenkephalin. Kidney International Reports, 2023. 8(11): p. 2345-2355."

33 "Blot, S., et al., The influence of acute kidney injury on antimicrobial dosing in critically ill patients: are dose reductions always necessary? Diagnostic Microbiology and Infectious Disease, 2014. 79(1): p. 77-84."

34 "Bolin, P., Jr., et al., Optimizing tacrolimus therapy in the maintenance of renal allografts: 12-month results. Transplantation, 2008. 86(1): p. 88-95."

35 "Bonfim, A.V., et al., RFRIST Study (Fractional Flow Reserve in Functional Quantification of Renal Allograft Artery Stenosis): Rationale and Study Design. Revista Brasileira de Cardiologia Invasiva (English Edition), 2012. 20(4): p. 420-426."

36 "Borràs, E., et al., Protein-Based Classifier to Predict Conversion from Clinically Isolated Syndrome to Multiple Sclerosis*. Molecular & Cellular Proteomics, 2016. 15(1): p. 318-328."

37 "Bor?tnar, ?., et al., Uromodulin and microRNAs in Kidney Transplantation-Association with Kidney Graft Function. Int J Mol Sci, 2020. 21(16)."

38 "Boschetti, E., L.E. Hernández-Castellano, and P.G. Righetti, Progress in farm animal proteomics: The contribution of combinatorial peptide ligand libraries. Journal of Proteomics, 2019. 197: p. 1-13."

39 "Bourgonje, A.R., et al., Systemic oxidative stress associates with new-onset hypertension in the general population. Free Radical Biology and Medicine, 2022. 187: p. 123-131."

40 "Brott, D.A., et al., Renal biomarker changes associated with hyaline droplet nephropathy in rats are time and potentially compound dependent. Toxicology, 2013. 303: p. 133-138."

41 "Butler, J.M., et al., A missense variant in CST3 exerts a recessive effect on susceptibility to age-related macular degeneration resembling its association with Alzheimer's disease. Hum Genet, 2015. 134(7): p. 705-15."

42 "Cabras, T., et al., Proteomic investigation of whole saliva in Wilson's disease. Journal of Proteomics, 2015. 128: p. 154-163."

43 "Camisasca, D.R., et al., A proteomic approach to compare saliva from individuals with and without oral leukoplakia. Journal of Proteomics, 2017. 151: p. 43-52."

44 "Campbell, E., et al., Enzyme - Switch sensors for therapeutic drug monitoring of immunotherapies. Biosensors and Bioelectronics, 2023. 237: p. 115488."

45 "Caragata, M., et al., Enrichment and identification of glycoproteins in human saliva using lectin magnetic bead arrays. Analytical Biochemistry, 2016. 497: p. 76-82."

46 "Cárdenas-González, M.C., et al., Proximal renal tubular injury in rats sub-chronically exposed to low fluoride concentrations. Toxicology and Applied Pharmacology, 2013. 272(3): p. 888-894."

47 "Cardinaels, E.P.M., et al., Clinical Interpretation of Elevated Concentrations of Cardiac Troponin T, but Not Troponin I, in Nursing Home Residents. Journal of the American Medical Directors Association, 2015. 16(10): p. 884-891."

48 "Carmel, R., Biomarkers of cobalamin (vitamin B-12) status in the epidemiologic setting: a critical overview of context, applications, and performance characteristics of cobalamin, methylmalonic acid, and holotranscobalamin II1234. The American Journal of Clinical Nutrition, 2011. 94(1): p. 348S-358S."

49 "Carnicelli, A.P., et al., Elevated Uric Acid Prevalence and Clinical Outcomes in Patients with Heart Failure with Preserved Ejection Fraction: Insights from RELAX. Am J Med, 2020. 133(12): p. e716-e721."

50 "Castillo-Lopez, E., et al., Diet and phytogenic supplementation substantially modulate the salivary proteome in dairy cows. Journal of Proteomics, 2023. 273: p. 104795."

51 "Cedazo-Minguez, A. and B. Winblad, Biomarkers for Alzheimer’s disease and other forms of dementia: Clinical needs, limitations and future aspects. Experimental Gerontology, 2010. 45(1): p. 5-14."

52 "Cederberg, K.L.J., et al., Proteomic insights into the pathophysiology of periodic limb movements and restless legs syndrome. Sleep Health, 2024. 10(1, Supplement): p. S161-S169."

53 "?ernocká, H., L. ?ímánková, and V. Ostatná, Fetuin and asialofetuin at charged surfaces: Influence of sialic acid presence. Journal of Electroanalytical Chemistry, 2021. 902: p. 115801."

54 "Chan, M.K., et al., Blood-based immune-endocrine biomarkers of treatment response in?depression. Journal of Psychiatric Research, 2016. 83: p. 249-259."

55 "Chang, C.K., et al., Systemic inflammation, coagulopathy, and acute renal insufficiency following endovascular thoracoabdominal aortic aneurysm repair. J Vasc Surg, 2009. 49(5): p. 1140-6."

56 "Chen, G.L. and J.Z. Su, [Atorvastatin attenuated contrast induced renal function damage]. Zhonghua Xin Xue Guan Bing Za Zhi, 2009. 37(5): p. 389-93."

57 "Chen, H.H., et al., Low-dose dopamine or low-dose nesiritide in acute heart failure with renal dysfunction: the ROSE acute heart failure randomized trial. Jama, 2013. 310(23): p. 2533-43."

58 "Chen, M.X., et al., Clinical interpretation of serum hepcidin-25 in inflammation and renal dysfunction. Journal of Mass Spectrometry and Advances in the Clinical Lab, 2022. 24: p. 43-49."

59 "Chen, W., et al., Effects of long non-coding RNA LINC00667 on renal tubular epithelial cell proliferation, apoptosis and renal fibrosis via the miR-19b-3p/LINC00667/CTGF signaling pathway in chronic renal failure. Cellular Signalling, 2019. 54: p. 102-114."

60 "Chen, Y., et al., Assessment of osteopontin as an early nephrotoxicity indicator in human renal proximal tubule cells and its application in evaluating lanthanum-induced nephrotoxicity. Ecotoxicology and Environmental Safety, 2024. 271: p. 115928."

61 "Chen, Y., et al., Renal dysfunction in AQP4 NMOSD and MS; a potential predictor of relapse and prognosis. Clinical Immunology, 2024. 259: p. 109875."

62 "Chen, Z., et al., Down-regulation of UBA6 exacerbates brain injury by inhibiting the activation of Notch signaling pathway to promote cerebral cell apoptosis in rat acute cerebral infarction model. Molecular and Cellular Probes, 2020. 53: p. 101612."

63 "Chenevier-Gobeaux, C., et al., Presepsin (sCD14-ST) in emergency department: The need for adapted threshold values? Clinica Chimica Acta, 2014. 427: p. 34-36."

64 "Cheng, L. and X.F. Tang, [Influence of levocarnitine on heart function and endocrine among patients with heart failure]. Zhonghua Liu Xing Bing Xue Za Zhi, 2013. 34(6): p. 630-2."

65 "Chiu, C.-Y., et al., The value of total protein in guiding management of infectious parapneumonic effusion by using matrix-assisted laser desorption/ionization time-of-flight mass spectrometry. Journal of Microbiology, Immunology and Infection, 2015. 48(5): p. 483-489."

66 "Cofiell, R., et al., Eculizumab reduces complement activation, inflammation, endothelial damage, thrombosis, and renal injury markers in aHUS. Blood, 2015. 125(21): p. 3253-62."

67 "Corcoran, D., et al., Vascular effects of serelaxin in patients with stable coronary artery disease: a randomized placebo-controlled trial. Cardiovasc Res, 2021. 117(1): p. 320-329."

68 "Costa-da-Silva, A.C., et al., Salivary ZG16B expression loss follows exocrine gland dysfunction related to oral chronic graft-versus-host disease. iScience, 2022. 25(1): p. 103592."

69 "Cremer, S.E., et al., The canine activated platelet secretome (CAPS): A translational model of thrombin‐evoked platelet activation response. Research and Practice in Thrombosis and Haemostasis, 2021. 5(1): p. 55-68."

70 "Cukoski, S., et al., Feasibility and impact of ketogenic dietary interventions in polycystic kidney disease: KETO-ADPKD—a randomized controlled trial. Cell Reports Medicine, 2023. 4(11): p. 101283."

71 "Cunsolo, V., et al., Zeus, Aesculapius, Amalthea and the proteome of goat milk. Journal of Proteomics, 2015. 128: p. 69-82."

72 "Curci, D., et al., Proteome-Wide Analysis Using SOMAscan Identifies and Validates Epidermal Growth Factor as a Disease Marker of Collagenous Gastritis. Gastro Hep Advances, 2022. 1(5): p. 689-702."

73 "Dai, F., et al., MicroRNA-375 inhibits laryngeal squamous cell carcinoma progression via targeting CST1. Brazilian Journal of Otorhinolaryngology, 2022. 88: p. S108-S116."

74 "D'Alessandro, A., et al., The egg white and yolk interactomes as gleaned from extensive proteomic data. Journal of Proteomics, 2010. 73(5): p. 1028-1042."

75 "Dardashti, A., et al., Erythropoietin and protection of renal function in cardiac surgery (the EPRICS Trial). Anesthesiology, 2014. 121(3): p. 582-90."

76 "Dartt, D.A., Tear Lipocalin: structure and Function. The Ocular Surface, 2011. 9(3): p. 126-138."

77 "Das, M., et al., Electrochemical detection of acute renal disease biomarker by Galinstan nanoparticles interfaced to bilayer polymeric structured dirhenium heptoxide film. Bioelectrochemistry, 2022. 147: p. 108194."

78 "de Gonzalo-Calvo, D., et al., Circulating microRNAs as emerging cardiac biomarkers responsive to acute exercise. International Journal of Cardiology, 2018. 264: p. 130-136."

79 "de Souza Santos, V., et al., Silymarin protects against radiocontrast-induced nephropathy in mice. Life Sciences, 2019. 228: p. 305-315."

80 "Demir, A., et al., A comparison of the effects of ketamine and remifentanil on renal functions in coronary artery bypass graft surgery. Ren Fail, 2015. 37(5): p. 819-26."

81 "Demir, N., et al., Efficacy and safety of rectal ibuprofen for patent ductus arteriosus closure in very low birth weight preterm infants. J Matern Fetal Neonatal Med, 2017. 30(17): p. 2119-2125."

82 "Devarajan, P., Proteomics for Biomarker Discovery in Acute Kidney Injury. Seminars in Nephrology, 2007. 27(6): p. 637-651."

83 "Dhami, R., M.A. Passini, and E.H. Schuchman, Identification of Novel Biomarkers for Niemann–Pick Disease Using Gene Expression Analysis of Acid Sphingomyelinase Knockout Mice. Molecular Therapy, 2006. 13(3): p. 556-564."

84 "Dipchand, A.I., et al., Myocyte growth, repair, and oxidative stress following pediatric heart transplantation. Pediatr Transplant, 2014. 18(7): p. 764-70."

85 "Dmitrieva, N.I., et al., Middle-age high normal serum sodium as a risk factor for accelerated biological aging, chronic diseases, and premature mortality. eBioMedicine, 2023. 87: p. 104404."

86 "Doi, Y., et al., The effect of cholecalciferol supplementation on allograft function in incident kidney transplant recipients: A randomized controlled study. American Journal of Transplantation, 2021. 21(9): p. 3043-3054."

87 "Doig, G.S., et al., Intravenous amino acid therapy for kidney function in critically ill patients: a randomized controlled trial. Intensive Care Med, 2015. 41(7): p. 1197-208."

88 "dos Santos, K.F., et al., Use of proteomics in the study of the acute phase of caprine arthritis encephalitis in seminal plasma. Small Ruminant Research, 2019. 181: p. 39-44."

89 "Dowling, P., et al., Proteomic and cell biological profiling of the renal phenotype of the mdx-4cv mouse model of Duchenne muscular dystrophy. European Journal of Cell Biology, 2020. 99(1): p. 151059."

90 "Doyle, M.E., et al., Autoimmune Dacryoadenitis of NOD/LtJ Mice and Its Subsequent Effects on Tear Protein Composition. The American Journal of Pathology, 2007. 171(4): p. 1224-1236."

91 "Driscoll, A., et al., Predictors of incident heart failure in patients after an acute coronary syndrome: The LIPID heart failure risk-prediction model. International Journal of Cardiology, 2017. 248: p. 361-368."

92 "Driver, T.H., et al., Low Serum Bicarbonate and Kidney Function Decline: The Multi-Ethnic Study of Atherosclerosis (MESA). American Journal of Kidney Diseases, 2014. 64(4): p. 534-541."

93 "Du, X., et al., Nicorandil Decreases Renal Injury in Patients With Coronary Heart Disease Complicated With Type I Cardiorenal Syndrome. J Cardiovasc Pharmacol, 2021. 78(5): p. e675-e680."

94 "Ederoth, P., et al., Ciclosporin to Protect Renal function In Cardiac Surgery (CiPRICS): a study protocol for a double-blind, randomised, placebo-controlled, proof-of-concept study. BMJ Open, 2016. 6(12): p. e012299."

95 "Ederoth, P., et al., Cyclosporine before Coronary Artery Bypass Grafting Does Not Prevent Postoperative Decreases in Renal Function: A Randomized Clinical Trial. Anesthesiology, 2018. 128(4): p. 710-717."

96 "Eijsvogels, T.M.H., et al., Impact of Statin Use on Exercise-Induced Cardiac Troponin Elevations. The American Journal of Cardiology, 2014. 114(4): p. 624-628."

97 "El-Samahy, M.A., et al., A proposed sample handling of ovine cotyledon for proteomic studies. Analytical Biochemistry, 2020. 593: p. 113585."

98 "Epstein, M., et al., Atorvastatin does not induce glomerular or tubular dysfunction even at high doses. J Cardiometab Syndr, 2007. 2(3): p. 163-7."

99 "Espinosa, C., A. Cuesta, and M.á. Esteban, Effects of dietary polyvinylchloride microparticles on general health, immune status and expression of several genes related to stress in gilthead seabream (Sparus aurata L.). Fish & Shellfish Immunology, 2017. 68: p. 251-259."

100 "Ewees, M.G.E.-D., et al., Modulation of mi-RNA25/Ox-LDL/NOX4 signaling pathway by polyphenolic compound Hydroxytyrosol as a new avenue to alleviate cisplatin-induced acute kidney injury, a mechanistic study in rats. Environmental Toxicology and Pharmacology, 2023. 103: p. 104262."

101 "Fach, E.M., et al., In Vitro Biomarker Discovery for Atherosclerosis by Proteomics*. Molecular & Cellular Proteomics, 2004. 3(12): p. 1200-1210."

102 "F?ste, C.K., et al., Characterisation of potential novel allergens in the fish parasite Anisakis simplex. EuPA Open Proteomics, 2014. 4: p. 140-155."

103 "Fan, Y., et al., Preventive effect of oral nicorandil on contrast-induced nephropathy in patients with renal insufficiency undergoing elective cardiac catheterization. Heart Vessels, 2016. 31(11): p. 1776-1782."

104 "Fan, Z., et al., Construct a classification decision tree model to select the optimal equation for estimating glomerular filtration rate and estimate it more accurately. Sci Rep, 2022. 12(1): p. 14877."

105 "Farag, M.R., et al., Yucca schidigera extract modulates the lead-induced oxidative damage, nephropathy and altered inflammatory response and glucose homeostasis in Japanese quails. Ecotoxicology and Environmental Safety, 2018. 156: p. 311-321."

106 "Fedele, F., et al., Levosimendan improves renal function in acute decompensated heart failure: possible underlying mechanisms. Eur J Heart Fail, 2014. 16(3): p. 281-8."

107 "Fernandes, M., et al., CanisOme — The protein signatures of Canis lupus familiaris diseases. Journal of Proteomics, 2016. 136: p. 193-201."

108 "Filippatos, G., et al., Serelaxin in acute heart failure patients with preserved left ventricular ejection fraction: results from the RELAX-AHF trial. Eur Heart J, 2014. 35(16): p. 1041-50."

109 "Filler, G., et al., Beta-trace protein as a marker of GFR — History, indications, and future research. Clinical Biochemistry, 2014. 47(13): p. 1188-1194."

110 "Folsom, A.R., et al., Effect of 9p21 genetic variation on coronary heart disease is not modified by other risk markers. The Atherosclerosis Risk in Communities (ARIC) Study. Atherosclerosis, 2012. 224(2): p. 435-439."

111 "Foster, M.C., et al., Non-GFR Determinants of Low-Molecular-Weight Serum Protein Filtration Markers in the Elderly: AGES-Kidney and MESA-Kidney. American Journal of Kidney Diseases, 2017. 70(3): p. 406-414."

112 "Foster, M.C., et al., Relations of Measures of Endothelial Function and Kidney Disease: The Framingham Heart Study. American Journal of Kidney Diseases, 2008. 52(5): p. 859-867."

113 "Foucher, C., et al., New Fixed-Dose Combinations of Fenofibrate/Simvastatin Therapy Significantly Improve the Lipid Profile of High-Risk Patients with Mixed Dyslipidemia Versus Monotherapies. Cardiovasc Ther, 2015. 33(6): p. 329-37."

114 "Franco-Martínez, L., et al., Changes in salivary analytes in canine parvovirus: A high-resolution quantitative proteomic study. Comparative Immunology, Microbiology and Infectious Diseases, 2018. 60: p. 1-10."

115 "Friedman, A.N., et al., Comparative effects of low-carbohydrate high-protein versus low-fat diets on the kidney. Clin J Am Soc Nephrol, 2012. 7(7): p. 1103-11."

116 "Fucikova, A., et al., Plasma concentration of fibronectin is decreased in patients with hypertrophic cardiomyopathy. Clinica Chimica Acta, 2016. 463: p. 62-66."

117 "Gao, J., et al., Microheterogeneity and preanalytical stability of protein biomarkers of inflammation and renal function. Talanta, 2021. 223: p. 121774."

118 "Gao, X.F., et al., Effect of Hybrid Blood Purification Treatment on Secondary Hyperparathyroidism for Maintenance Hemodialysis Patients. Blood Purif, 2018. 46(1): p. 19-26."

119 "Genco, R.J., Salivary diagnostic tests. The Journal of the American Dental Association, 2012. 143: p. 3S-5S."

120 "Gianazza, E., et al., Gender proteomics I. Which proteins in non-sexual organs. Journal of Proteomics, 2018. 178: p. 7-17."

121 "Gianazza, E., et al., Gender proteomics II. Which proteins in sexual organs. Journal of Proteomics, 2018. 178: p. 18-30."

122 "Giannitsis, E., et al., Gender-specific reference values for high-sensitivity cardiac troponin T and I in well-phenotyped healthy individuals and validity of high-sensitivity assay designation. Clinical Biochemistry, 2020. 78: p. 18-24."

123 "Gok Oguz, E., et al., Lack of nephrotoxicity of gadopentetate dimeglumine-enhanced non-vascular MRI and MRI without contrast agent in patients at high-risk for acute kidney injury. Med Sci Monit, 2013. 19: p. 942-8."

124 "Gokmen, T., et al., Efficacy and safety of oral versus intravenous ibuprofen in very low birth weight preterm infants with patent ductus arteriosus. J Pediatr, 2011. 158(4): p. 549-554.e1."

125 "Goldstein, A. and M.J. Falk, Single Large-Scale Mitochondrial DNA Deletion Syndromes, in GeneReviews(?), M.P. Adam, et al., Editors. 1993, University of Washington, Seattle"

126 "Gomez-Varela, D., A.M. Barry, and M. Schmidt, Proteome-based systems biology in chronic pain. Journal of Proteomics, 2019. 190: p. 1-11."

127 "González-Antu?a, A., et al., Determination of Cystatin C in human serum by isotope dilution mass spectrometry using mass overlapping peptides. Journal of Proteomics, 2015. 112: p. 141-155."

128 "Graziani, F., et al., Enamel matrix derivative stabilizes blood clot and improves clinical healing in deep pockets after flapless periodontal therapy: A Randomized Clinical Trial. J Clin Periodontol, 2019. 46(2): p. 231-240."

129 "Greening, D.W. and R.J. Simpson, A centrifugal ultrafiltration strategy for isolating the low-molecular weight (≤25K) component of human plasma proteome. Journal of Proteomics, 2010. 73(3): p. 637-648."

130 "Grodin, J.L., et al., Implications of Alternative Hepatorenal Prognostic Scoring Systems in Acute Heart Failure (from DOSE-AHF and ROSE-AHF). Am J Cardiol, 2017. 119(12): p. 2003-2009."

131 "Grodin, J.L., et al., Implications of Serum Chloride Homeostasis in Acute Heart Failure (from ROSE-AHF). Am J Cardiol, 2017. 119(1): p. 78-83."

132 "Grufman, H., et al., Elevated IL-27 in patients with acute coronary syndrome is associated with adverse ventricular remodeling and increased risk of recurrent myocardial infarction and cardiovascular death. Cytokine, 2019. 122: p. 154208."

133 "Grunz-Borgmann, E.A., et al., Structural equation modeling identifies markers of damage and function in the aging male Fischer 344 rat. Mechanisms of Ageing and Development, 2016. 156: p. 55-62."

134 "Gruppen, E.G., et al., GlycA, a novel proinflammatory glycoprotein biomarker, and high-sensitivity C-reactive protein are inversely associated with sodium intake after controlling for adiposity: the Prevention of Renal and Vascular End-Stage Disease study11. The American Journal of Clinical Nutrition, 2016. 104(2): p. 415-422."

135 "Gruppen, E.G., et al., Serum paraoxonase-1 activity is associated with light to moderate alcohol consumption: the PREVEND cohort study. The American Journal of Clinical Nutrition, 2018. 108(6): p. 1283-1290."

136 "Gu, S., et al., Human kidney organoids model of Esculentoside A nephrotoxicity to investigate the role of epithelial-mesenchymal transition via STING signaling. Toxicology Letters, 2023. 373: p. 172-183."

137 "Gualano, B., et al., Effects of creatine supplementation on renal function: a randomized, double-blind, placebo-controlled clinical trial. Eur J Appl Physiol, 2008. 103(1): p. 33-40."

138 "Guarrera, S., et al., Peripheral Blood DNA Methylation as Potential Biomarker of Malignant Pleural Mesothelioma in Asbestos-Exposed Subjects. Journal of Thoracic Oncology, 2019. 14(3): p. 527-539."

139 "Gunawardana, C.G., et al., The Human Tau Interactome: Binding to the Ribonucleoproteome, and Impaired Binding of the Proline-to-Leucine Mutant at Position 301 (P301L) to Chaperones and the Proteasome*. Molecular & Cellular Proteomics, 2015. 14(11): p. 3000-3014."

140 "Guo, J., et al., Nanomaterial Labels in Lateral Flow Immunoassays for Point-of-Care-Testing. Journal of Materials Science & Technology, 2021. 60: p. 90-104."

141 "Guo, Z., et al., The influence of influenza A (H1N1) virus on creatinine and cystatin C. Clinica Chimica Acta, 2010. 411(23): p. 2040-2042."

142 "Gupta, S., et al., Ultrasensitive transglutaminase based nanosensor for early detection of celiac disease in human. International Journal of Biological Macromolecules, 2017. 105: p. 905-911."

143 "Gutiérrez, A.M., et al., Detection of potential markers for systemic disease in saliva of pigs by proteomics: A pilot study. Veterinary Immunology and Immunopathology, 2013. 151(1): p. 73-82."

144 "Haddad, E.H., et al., Associations of Circulating Methylmalonic Acid and Vitamin B-12 Biomarkers Are Modified by Vegan Dietary Pattern in Adult and Elderly Participants of the Adventist Health Study 2 Calibration Study. Current Developments in Nutrition, 2020. 4(2): p. nzaa008."

145 "Hadzimuratovic, E., et al., Postasphyxial renal injury in newborns as a prognostic factor of neurological outcome. J Matern Fetal Neonatal Med, 2014. 27(4): p. 407-10."

146 "Haenisch, F., et al., Towards a blood-based diagnostic panel for bipolar disorder. Brain, Behavior, and Immunity, 2016. 52: p. 49-57."

147 "Hamdy, S., et al., Curcumin mitigates gentamicin induced-renal and cardiac toxicity via modulation of Keap1/Nrf2, NF-κB/iNOS and Bcl-2/BAX pathways. Food and Chemical Toxicology, 2024. 183: p. 114323."

148 "Hamzah, L., et al., Early safety of tenofovir alafenamide in patients with a history of tubulopathy on tenofovir disoproxil fumarate: a randomized controlled clinical trial. HIV Med, 2020. 21(3): p. 198-203."

149 "Hanedan, B., et al., Investigation of the effects of hesperidin and chrysin on renal injury induced by colistin in rats. Biomedicine & Pharmacotherapy, 2018. 108: p. 1607-1616."

150 "Hanna, D.A., et al., Lysosomal membrane stabilization by imipramine attenuates gentamicin-induced renal injury: Enhanced LAMP2 expression, down-regulation of cytoplasmic cathepsin D and tBid/cytochrome c/cleaved caspase-3 apoptotic signaling. International Immunopharmacology, 2024. 126: p. 111179."

151 "Hansson, E., et al., An explorative study of inflammation-related proteins associated with kidney injury in male heat-stressed workers. Journal of Thermal Biology, 2023. 112: p. 103433."

152 "Harden, C.J., et al., Evaluation of the salivary proteome as a surrogate tissue for systems biology approaches to understanding appetite. Journal of Proteomics, 2012. 75(10): p. 2916-2923."

153 "Hawthorn, L., et al., Characterization of cell-type specific profiles in tissues and isolated cells from squamous cell carcinomas of the lung. Lung Cancer, 2006. 53(2): p. 129-142."

154 "Hermans, M.A.W., et al., Altered leukocyte subsets and immune proteome indicate proinflammatory mechanisms in mastocytosis. Journal of Allergy and Clinical Immunology, 2022. 150(1): p. 146-156.e10."

155 "Herrera-Gutiérrez, M.E., et al., Variability in renal dysfunction defining criteria and detection methods in intensive care units: Are the international consensus criteria used for diagnosing renal dysfunction? Medicina Intensiva (English Edition), 2012. 36(4): p. 264-269."

156 "Heyse, W., et al., Identification of patient subtypes based on protein expression for prediction of heart failure after myocardial infarction. iScience, 2023. 26(3): p. 106171."

157 "Hiatt, W.R., et al., A validated biomarker panel to identify peripheral artery disease. Vasc Med, 2012. 17(6): p. 386-93."

158 "Hiramoto, J.S., et al., Inflammation and Coagulation Markers and Kidney Function Decline: The Multi-Ethnic Study of Atherosclerosis (MESA). American Journal of Kidney Diseases, 2012. 60(2): p. 225-232."

159 "Hoover, M.E., et al., Proteomic characterization of a trauma-based rat model of heterotopic ossification identifies interactive signaling networks as potential therapeutic targets. Journal of Proteomics, 2020. 226: p. 103907."

160 "Horvati?, A., et al., Quantitative proteomics using tandem mass tags in relation to the acute phase protein response in chicken challenged with Escherichia coli lipopolysaccharide endotoxin. Journal of Proteomics, 2019. 192: p. 64-77."

161 "Hou, J., et al., A fluorescence-based immunochromatographic assay using quantum dot-encapsulated nanoparticles for the rapid and sensitive detection of fetuin-B. Analytica Chimica Acta, 2024. 1288: p. 342143."

162 "Hrubá, P., et al., Molecular diagnostics identifies risks for graft dysfunction despite borderline histologic changes. Kidney International, 2015. 88(4): p. 785-795."

163 "Hu, S., et al., Differentially expressed protein markers in human submandibular and sublingual secretions. Int J Oncol, 2004. 25(5): p. 1423-30."

164 "Hu, X.Y., et al., [Clinical observation on treatment of type 2 cardiac and kidney syndrome by combination of traditional Chinese and Western medicines]. Zhongguo Zhong Yao Za Zhi, 2017. 42(19): p. 3815-3818."

165 "Hui, H., et al., [Protective effect of amlodipine against contrast agent-induced renal injury in elderly patients with coronary heart disease]. Nan Fang Yi Ke Da Xue Xue Bao, 2012. 32(11): p. 1580-3."

166 "Hussain, J., et al., Impaired Renal Function and Major Cardiovascular Events in Young Adults. Journal of the American College of Cardiology, 2023. 82(13): p. 1316-1327."

167 "Iavarone, F., et al., Characterization of salivary proteins of schizophrenic and bipolar disorder patients by top-down proteomics. Journal of Proteomics, 2014. 103: p. 15-22."

168 "Ilies, M., et al., Impact of blood sample collection methods on blood protein profiling studies. Clinica Chimica Acta, 2017. 471: p. 128-134."

169 "Imafidon, C.E., R.O. Akomolafe, and O.G. Oke, Saliva renal function biomarkers as alternatives to plasma concentrations in obesity-induced kidney injury. Obesity Medicine, 2020. 17: p. 100195."

170 "Imanguli, M.M., et al., Changes in salivary proteome following allogeneic hematopoietic stem cell transplantation. Experimental Hematology, 2007. 35(2): p. 184-192."

171 "Inda-Filho, A.J., et al., Do intravenous N-acetylcysteine and sodium bicarbonate prevent high osmolal contrast-induced acute kidney injury? A randomized controlled trial. PLoS One, 2014. 9(9): p. e107602."

172 "Isaacson, R.S., et al., Individualized clinical management of patients at risk for Alzheimer's dementia. Alzheimer's & Dementia, 2019. 15(12): p. 1588-1602."

173 "Ishibashi, S., et al., Efficacy and safety of pemafibrate (K-877), a?selective peroxisome proliferator-activated receptor α modulator, in patients with dyslipidemia: Results from a 24-week, randomized, double blind, active-controlled, phase 3 trial. J Clin Lipidol, 2018. 12(1): p. 173-184."

174 "Ishigami, J., et al., Changes in Serum Intact Fibroblast Growth Factor 23 Concentrations From Midlife to Late Life and Their Predictors in the Community: The ARIC Study. Mayo Clinic Proceedings: Innovations, Quality & Outcomes, 2022. 6(3): p. 209-217."

175 "Issa, V.S., et al., Hypertonic saline solution for prevention of renal dysfunction in patients with decompensated heart failure. Int J Cardiol, 2013. 167(1): p. 34-40."

176 "Issa, V.S., L. Andrade, and E.A. Bocchi, Current strategies for preventing renal dysfunction in patients with heart failure: a heart failure stage approach. Clinics, 2013. 68(3): p. 401-409."

177 "Iversen, E., et al., Performance of Panel-Estimated GFR Among Hospitalized Older Adults. Am J Kidney Dis, 2023. 82(6): p. 715-724."

178 "Ivey, K.L., et al., Associations of proanthocyanidin intake with renal function and clinical outcomes in elderly women. PLoS One, 2013. 8(8): p. e71166."

179 "Ivica, J., G. Sanmugalingham, and R. Selvaratnam, Alerting to acute kidney injury - Challenges, benefits, and strategies. Practical Laboratory Medicine, 2022. 30: p. e00270."

180 "Jackson, C.E., et al., Albuminuria in chronic heart failure: prevalence and prognostic importance. The Lancet, 2009. 374(9689): p. 543-550."

181 "Jeong, M., et al., Change in kidney damage biomarkers after 13weeks of exposing rats to the complex of Paecilomyces sinclairii and its host Bombyx mori larvae. Food and Chemical Toxicology, 2013. 59: p. 177-186."

182 "Jiang, Q., et al., Overexpression of Fetuin-A Counteracts Ectopic Mineralization in a Mouse Model of Pseudoxanthoma Elasticum (Abcc6?/?). Journal of Investigative Dermatology, 2010. 130(5): p. 1288-1296."

183 "Jiang, Q., Q. Li, and J. Uitto, Aberrant Mineralization of Connective Tissues in a Mouse Model of Pseudoxanthoma Elasticum: Systemic and Local Regulatory Factors. Journal of Investigative Dermatology, 2007. 127(6): p. 1392-1402."

184 "Jiang, W.-P., et al., Diagnostic model of saliva peptide finger print analysis of oral squamous cell carcinoma patients using weak cation exchange magnetic beads. Bioscience Reports, 2015. 35(3)."

185 "Jin, S., et al., Plasma factor D is cross-sectionally associated with low-grade inflammation, endothelial dysfunction and cardiovascular disease: The Maastricht study. Atherosclerosis, 2023. 377: p. 60-67."

186 "Jodele, S., et al., A new paradigm: Diagnosis and management of HSCT-associated thrombotic microangiopathy as multi-system endothelial injury. Blood Reviews, 2015. 29(3): p. 191-204."

187 "Jonasson, T.F., et al., Hyperhomocysteinaemia is not associated with increased levels of asymmetric dimethylarginine in patients with ischaemic heart disease. Eur J Clin Invest, 2003. 33(7): p. 543-9."

188 "Jones, T.E., J.V. Peter, and J. Field, Aminoglycoside clearance is a good estimate of creatinine clearance in intensive care unit patients. Anaesth Intensive Care, 2009. 37(6): p. 944-52."

189 "Jonsson, O., et al., Prophylaxis against bone loss in Kock reservoir patients with reduced glomerular filtration rate. Scand J Urol Nephrol, 2005. 39(3): p. 200-5."

190 "Ju, Y., et al., NOD and NOR mice exhibit comparable development of lacrimal gland secretory dysfunction but NOD mice have more severe autoimmune dacryoadenitis. Experimental Eye Research, 2018. 176: p. 243-251."

191 "Jung, W.J., et al., Dose Optimization of Vancomycin Using a Mechanism-based Exposure–Response Model in Pediatric Infectious Disease Patients. Clinical Therapeutics, 2021. 43(1): p. 185-194.e16."

192 "Jungbauer, A. and C. Machold, Chapter 16 Chromatography of proteins, in Journal of Chromatography Library, E. Heftmann, Editor. 2004, Elsevier. p. 669-737."

193 "Kammerer, T., et al., No Differences in Renal Function between Balanced 6% Hydroxyethyl Starch (130/0.4) and 5% Albumin for Volume Replacement Therapy in Patients Undergoing Cystectomy: A Randomized Controlled Trial. Anesthesiology, 2018. 128(1): p. 67-78."

194 "Kang, S.-Y., et al., The association between specific IgE antibodies to component allergens and allergic symptoms on dog and cat exposure among Korean pet exhibition participants. World Allergy Organization Journal, 2022. 15(11): p. 100709."

195 "Karamessinis, P.M., et al., Marked Defects in the Expression and Glycosylation of α2-HS Glycoprotein/Fetuin-A in Plasma from Neonates with Intrauterine Growth Restriction: Proteomics Screening and Potential Clinical Implications*. Molecular & Cellular Proteomics, 2008. 7(3): p. 591-599."

196 "Kaski, J.C., et al., A comparative study of biomarkers for risk prediction in acute coronary syndrome—Results of the SIESTA (Systemic Inflammation Evaluation in non-ST-elevation Acute coronary syndrome) study. Atherosclerosis, 2010. 212(2): p. 636-643."

197 "Keir, H.R., et al., Neutrophil extracellular traps, disease severity, and antibiotic response in bronchiectasis: an international, observational, multicohort study. The Lancet Respiratory Medicine, 2021. 9(8): p. 873-884."

198 "Kelly, J., et al., Complex reference value distributions and partitioned reference intervals across the pediatric age range for 14 specialized biochemical markers in the CALIPER cohort of healthy community children and adolescents. Clinica Chimica Acta, 2015. 450: p. 196-202."

199 "Khames, A., et al., Nicorandil combats doxorubicin–induced nephrotoxicity via amendment of TLR4/P38 MAPK/NFκ-B signaling pathway. Chemico-Biological Interactions, 2019. 311: p. 108777."

200 "Kiessling, A.H., et al., Pre-filling of the extracorporeal circuit with autologous blood is safe, but not effective in optimizing biocompatibility in high-risk patients. Perfusion, 2012. 27(5): p. 371-7."

201 "Kilic, T., et al., Comparison of the long-term prognostic value of Cystatin C to other indicators of renal function, markers of inflammation and systolic dysfunction among patients with acute coronary syndrome. Atherosclerosis, 2009. 207(2): p. 552-558."

202 "Kim, H., et al., Alternative kidney filtration markers and the risk of major macrovascular and microvascular events, and all-cause mortality in individuals with type 2 diabetes in the ADVANCE trial. J Diabetes, 2020. 12(12): p. 929-941."

203 "Kim, H.S., et al., Biochemical and clinical correlation of intraplaque neovascularization using contrast-enhanced ultrasound of the carotid artery. Atherosclerosis, 2014. 233(2): p. 579-583."

204 "Kim, J.E., et al., Nicardipine infusion for hypotensive anesthesia during orthognathic surgery has protective effect on renal function. J Oral Maxillofac Surg, 2014. 72(1): p. 41-6."

205 "Kim, J.H., et al., Effect of erythropoietin on the incidence of acute kidney injury following complex valvular heart surgery: a double blind, randomized clinical trial of efficacy and safety. Crit Care, 2013. 17(5): p. R254."

206 "Kim, K.S., et al., Curcumin ameliorates cadmium-induced nephrotoxicity in Sprague-Dawley rats. Food and Chemical Toxicology, 2018. 114: p. 34-40."

207 "Kishazi, E., et al., Thyroid-associated orbitopathy and tears: A proteomics study. J Proteomics, 2018. 170: p. 110-116."

208 "Klein, R., et al., Oxidized Low-density Lipoprotein and the Incidence of Age-related Macular Degeneration. Ophthalmology, 2019. 126(5): p. 752-758."

209 "K?lling, M., et al., The Circular RNA ciRs-126 Predicts Survival in Critically Ill Patients With Acute Kidney Injury. Kidney International Reports, 2018. 3(5): p. 1144-1152."

210 "Koopal, C., et al., Effect of adding bezafibrate to standard lipid-lowering therapy on post-fat load lipid levels in patients with familial dysbetalipoproteinemia. A randomized placebo-controlled crossover trial. Journal of Lipid Research, 2017. 58(11): p. 2180-2187."

211 "Kooshki, H., et al., Developing a DNA aptamer-based approach for biosensing cystatin-c in serum: An alternative to antibody-based methods. Analytical Biochemistry, 2019. 584: p. 113386."

212 "Korandji, C., et al., Asymmetric dimethylarginine (ADMA) and hyperhomocysteinemia in patients with acute myocardial infarction. Clinical Biochemistry, 2007. 40(1): p. 66-72."

213 "Korytowska-Przybylska, N., et al., Development of a novel method for the simultaneous detection of trimethylamine N-oxide and creatinine in the saliva of patients with chronic kidney disease – Its utility in saliva as an alternative to blood. Journal of Pharmaceutical and Biomedical Analysis, 2023. 234: p. 115519."

214 "Kozono, A., et al., Comparison of predictive accuracy of teicoplanin concentration using creatinine clearance and glomerular filtration rate estimated by serum creatinine or cystatin C. Journal of Infection and Chemotherapy, 2016. 22(5): p. 314-318."

215 "Král, M., et al., Troponin T: Correlation with location and volume of acute brain infarction. International Journal of Cardiology, 2015. 181: p. 127-132."

216 "Krieter, D.H., et al., Effects of a polyelectrolyte additive on the selective dialysis membrane permeability for low-molecular-weight proteins. Nephrol Dial Transplant, 2007. 22(2): p. 491-9."

217 "Krieter, D.H., et al., Matching efficacy of online hemodiafiltration in simple hemodialysis mode. Artif Organs, 2008. 32(12): p. 903-9."

218 "Krieter, D.H., H.D. Lemke, and C. Wanner, A new synthetic dialyzer with advanced permselectivity for enhanced low-molecular weight protein removal. Artif Organs, 2008. 32(7): p. 547-54."

219 "Kroksveen, A.C., et al., Discovery and initial verification of differentially abundant proteins between multiple sclerosis patients and controls using iTRAQ and SID-SRM. Journal of Proteomics, 2013. 78: p. 312-325."

220 "Kusunoki, H., et al., Estimation of Muscle Mass Using Creatinine/Cystatin C Ratio in Japanese Community-Dwelling Older People. Journal of the American Medical Directors Association, 2022. 23(5): p. 902.e21-902.e31."

221 "Laisalmi, M., et al., The effect of ketorolac and sevoflurane anesthesia on renal glomerular and tubular function. Anesth Analg, 2001. 93(5): p. 1210-3."

222 "Lameris, R., et al., A bispecific T?cell engager recruits both type 1 NKT and Vγ9Vδ2-T cells for the treatment of CD1d-expressing hematological malignancies. Cell Reports Medicine, 2023. 4(3): p. 100961."

223 "Landau, M., et al., Correlates of insulin resistance in older individuals with and without kidney disease. Nephrol Dial Transplant, 2011. 26(9): p. 2814-9."

224 "Landi, C., et al., A functional proteomics approach to the comprehension of sarcoidosis. Journal of Proteomics, 2015. 128: p. 375-387."

225 "Laskin, B.L., et al., Estimated versus measured glomerular filtration rate in children before hematopoietic cell transplantation. Biol Blood Marrow Transplant, 2014. 20(12): p. 2056-61."

226 "Laskowska, E., D. Kuczyńska-Wi?nik, and B. Lipińska, Proteomic analysis of protein homeostasis and aggregation. Journal of Proteomics, 2019. 198: p. 98-112."

227 "Leanpolchareanchai, J. and N. Nuchtavorn, Wearable microneedle-based colorimetric and fluorescence sensing for transdermal diagnostics. Talanta Open, 2023. 8: p. 100247."

228 "Lee, B.S., et al., Effect of furosemide on ductal closure and renal function in indomethacin-treated preterm infants during the early neonatal period. Neonatology, 2010. 98(2): p. 191-9."

229 "Lee, C.-T., et al., Gene expression profiling in mouse lung following polymeric hexamethylene diisocyanate exposure. Toxicology and Applied Pharmacology, 2005. 205(1): p. 53-64."

230 "Lee, H.-J., et al., Repeated intravenous infusion of human apolipoprotein(a) kringle V is associated with reversible dose-dependent acute tubulointerstitial nephritis without affecting glomerular filtration function. Toxicology Letters, 2012. 212(3): p. 298-306."

231 "Lehmann, S., et al., Clinical mass spectrometry proteomics (cMSP) for medical laboratory: What does the future hold? Clinica Chimica Acta, 2017. 467: p. 51-58."

232 "Lenehan, P.J., et al., Anemia during SARS-CoV-2 infection is associated with rehospitalization after viral clearance. iScience, 2021. 24(7): p. 102780."

233 "Leung, N., S.H. Nasr, and S. Sethi, How I treat amyloidosis: the importance of accurate diagnosis and amyloid typing. Blood, 2012. 120(16): p. 3206-3213."

234 "Li, B., et al., Fatty acid binding protein 4 has prognostic value in peripheral artery disease. Journal of Vascular Surgery, 2023. 78(3): p. 719-726."

235 "Li, D., et al., SERS based Y-shaped aptasensor for early diagnosis of acute kidney injury??Electronic supplementary information (ESI) available. See https://doi.org/10.1039/d2ra02813a. RSC Advances, 2022. 12(25): p. 15910-15917."

236 "Li, F., et al., Differential MicroRNA Expressions in Human Peripheral Blood Mononuclear Cells Are Predictive of Renal Allograft Function. Transplantation Proceedings, 2019. 51(3): p. 715-721."

237 "Li, N., et al., [Application effects of bundle nursing of citric acid extracorporeal anticoagulation on continuous renal replacement therapy of severe burn patients]. Zhonghua Shao Shang Yu Chuang Mian Xiu Fu Za Zhi, 2022. 38(1): p. 29-37."

238 "Li, Y., et al., Associations between perfluoroalkyl substances and thyroid hormones after high exposure through drinking water. Environmental Research, 2021. 194: p. 110647."

239 "Li, Y., et al., Decreased expression of ADAM10 on monocytes is associated with chronic allograft dysfunction in kidney transplant recipients. International Immunopharmacology, 2023. 115: p. 109710."

240 "Li, Y., et al., Determinants of serum half-lives for linear and branched perfluoroalkyl substances after long-term high exposure—A study in Ronneby, Sweden. Environment International, 2022. 163: p. 107198."

241 "Liang, Y., et al., MiR-100-3p and miR-877-3p regulate overproduction of IL-8 and IL-1β in mesangial cells activated by secretory IgA from IgA nephropathy patients. Experimental Cell Research, 2016. 347(2): p. 312-321."

242 "Likhvantsev, V.V., et al., Nuclear DNA as Predictor of Acute Kidney Injury in Patients Undergoing Coronary Artery Bypass Graft: A Pilot Study. Journal of Cardiothoracic and Vascular Anesthesia, 2017. 31(6): p. 2080-2085."

243 "Lin, C.-C., et al., Identification of protein expression alterations in gefitinib-resistant human lung adenocarcinoma: PCNT and mPR play key roles in the development of gefitinib-associated resistance. Toxicology and Applied Pharmacology, 2015. 288(3): p. 359-373."

244 "Lindberg, M., et al., Distribution of creatinine and estimated glomerular filtration rate in healthy schoolchildren: The Health Oriented Pedagogical Project (HOPP). Scand J Clin Lab Invest, 2021. 81(3): p. 244-249."

245 "Lindholm, ?., et al., Effect of sibutramine on weight reduction in women with polycystic ovary syndrome: a randomized, double-blind, placebo-controlled trial. Fertil Steril, 2008. 89(5): p. 1221-1228."

246 "Liu, W.J., et al., Renoprotective effect of alprostadil in combination with statins in patients with mild to moderate renal failure undergoing coronary angiography. Chin Med J (Engl), 2013. 126(18): p. 3475-80."

247 "Lomivorotov, V.V., et al., Infusion of 7.2% NaCl/6% hydroxyethyl starch 200/0.5 in on-pump coronary artery bypass surgery patients: a randomized, single-blind pilot study. Shock, 2014. 41(3): p. 193-9."

248 "Longenecker, C.T., et al., Reductions in Plasma Cystatin C After Initiation of Antiretroviral Therapy Are Associated With Reductions in Inflammation: ACTG A5224s. J Acquir Immune Defic Syndr, 2015. 69(2): p. 168-77."

249 "Louati, K., et al., Differential Proteome Profiling Analysis under Pesticide Stress by the Use of a Nano-UHPLC-MS/MS Untargeted Proteomic-Based Approach on a 3D-Developed Neurospheroid Model: Identification of Protein Interactions, Prognostic Biomarkers, and Potential Therapeutic Targets in Human IDH Mutant High-Grade Gliomas. Journal of Proteome Research, 2023. 22(11): p. 3534-3558."

250 "Lozano-Paniagua, D., et al., Renal tubular dysfunction in greenhouse farmers exposed to pesticides unveiled by a panel of molecular biomarkers of kidney injury. Environmental Research, 2023. 238: p. 117200."

251 "Lundberg, M., et al., Multiplexed Homogeneous Proximity Ligation Assays for High-throughput Protein Biomarker Research in Serological Material*. Molecular & Cellular Proteomics, 2011. 10(4): p. M110.004978."

252 "Lunde, N.N., et al., Increased levels of legumain in plasma and plaques from patients with carotid atherosclerosis. Atherosclerosis, 2017. 257: p. 216-223."

253 "Luo, H.-L., et al., Serum human epididymis protein 4 is associated with disease severity in patients with IgA nephropathy. Clinical Biochemistry, 2024. 123: p. 110701."

254 "Ma, S., et al., Effects and mechanisms of Chinese herbal medicine on IgA nephropathy. Phytomedicine, 2023. 117: p. 154913."

255 "Maes, O.C., et al., Characterization of α1-antitrypsin as a heme oxygenase-1 suppressor in Alzheimer plasma. Neurobiology of Disease, 2006. 24(1): p. 89-100."

256 "Magnadóttir, B., et al., Deiminated proteins and extracellular vesicles - Novel serum biomarkers in whales and orca. Comparative Biochemistry and Physiology Part D: Genomics and Proteomics, 2020. 34: p. 100676."

257 "Mahajan, A., et al., Daily oral sodium bicarbonate preserves glomerular filtration rate by slowing its decline in early hypertensive nephropathy. Kidney Int, 2010. 78(3): p. 303-9."

258 "Mahfoud, F., et al., Renal hemodynamics and renal function after catheter-based renal sympathetic denervation in patients with resistant hypertension. Hypertension, 2012. 60(2): p. 419-24."

259 "Maioli, M., et al., Bioimpedance-Guided Hydration for the Prevention of Contrast-Induced Kidney?Injury: The HYDRA Study. J Am Coll Cardiol, 2018. 71(25): p. 2880-2889."

260 "Malard, V., et al., Analytical constraints for the analysis of human cell line secretomes by shotgun proteomics. Journal of Proteomics, 2012. 75(3): p. 1043-1054."

261 "Malyszko, J., et al., Copeptin in Relation to New York Heart Association Class in Heart Transplant Recipients and Kidney Transplant Recipients. Transplantation Proceedings, 2010. 42(10): p. 4259-4262."

262 "Mancia, G., et al., Guía de práctica clínica de la ESH/ESC para el manejo de la hipertensión arterial (2013). Hipertensión y Riesgo Vascular, 2013. 30: p. 4-91."

263 "Manconi, B., et al., Top-down proteomic profiling of human saliva in multiple sclerosis patients. Journal of Proteomics, 2018. 187: p. 212-222."

264 "Martin, P.G.P., et al., Transcriptomic modifications of the thyroid gland upon exposure to phytosanitary-grade fipronil: Evidence for the activation of compensatory pathways. Toxicology and Applied Pharmacology, 2020. 389: p. 114873."

265 "Marzougui, Z., et al., Marine toxin C17-SAMT causes major structural damage to vital organs in mice following subchronic toxicity trials. Ecotoxicology and Environmental Safety, 2023. 256: p. 114887."

266 "Mateos, J., et al., High-resolution quantitative proteomics applied to the study of the specific protein signature in the sputum and saliva of active tuberculosis patients and their infected and uninfected contacts. Journal of Proteomics, 2019. 195: p. 41-52."

267 "McDonald, J.S., et al., Postcontrast Acute Kidney Injury in Pediatric Patients: A Cohort Study. American Journal of Kidney Diseases, 2018. 72(6): p. 811-818."

268 "Meert, N., et al., Comparison of removal capacity of two consecutive generations of high-flux dialysers during different treatment modalities. Nephrol Dial Transplant, 2011. 26(8): p. 2624-30."

269 "Meseguer-Donlo, J., et al., HIV infection is associated with upregulated circulating levels of the inflammaging miR-21-5p. Journal of Microbiology, Immunology and Infection, 2023. 56(5): p. 931-938."

270 "Miao, Y., et al., Alprostadil plays a protective role in contrast-induced nephropathy in the elderly. Int Urol Nephrol, 2013. 45(4): p. 1179-85."

271 "Michos, E.D., et al., 25-hydroxyvitamin D levels, vitamin D binding protein gene polymorphisms and incident coronary heart disease among whites and blacks: The ARIC study. Atherosclerosis, 2015. 241(1): p. 12-17."

272 "Millard, R.W. and M. Tranter, Complementary, Alternative, and Putative Nontroponin Biomarkers of Acute Coronary Syndrome: New Resources for Future Risk Assessment Calculators. Revista Espa?ola de Cardiología (English Edition), 2014. 67(4): p. 312-320."

273 "Misko, A., et al., Mucolipidosis IV, in GeneReviews(?), M.P. Adam, et al., Editors. 1993, University of Washington, Seattle"

274 "Mitrovic, V., et al., Cardio-renal effects of the A1 adenosine receptor antagonist SLV320 in patients with heart failure. Circ Heart Fail, 2009. 2(6): p. 523-31."

275 "Mocroft, A., et al., Interruption of antiretroviral therapy is associated with increased plasma cystatin C. Aids, 2009. 23(1): p. 71-82."

276 "Mohapatra, S., et al., Seasonal variation in fluorescence characteristics of dissolved organic matter in wastewater and identification of proteins through HRLC-MS/MS. Journal of Hazardous Materials, 2021. 413: p. 125453."

277 "Mohebi, R., et al., Inflammatory biomarkers and risk of cardiovascular events in patients undergoing coronary angiography. American Heart Journal, 2022. 252: p. 51-59."

278 "Moisan, A., et al., Inhibition of EGF Uptake by Nephrotoxic Antisense Drugs In?Vitro and Implications for Preclinical Safety Profiling. Molecular Therapy - Nucleic Acids, 2017. 6: p. 89-105."

279 "Moliner, P., et al., Bio-profiling and bio-prognostication of chronic heart failure with mid-range ejection fraction. International Journal of Cardiology, 2018. 257: p. 188-192."

280 "Mollenhauer, B., et al., Direct quantification of CSF α-synuclein by ELISA and first cross-sectional study in patients with neurodegeneration. Experimental Neurology, 2008. 213(2): p. 315-325."

281 "Moore, P.K., R.K. Hsu, and K.D. Liu, Management of Acute Kidney Injury: Core Curriculum 2018. American Journal of Kidney Diseases, 2018. 72(1): p. 136-148."

282 "Morales-García, L.J. and M.S. Pacheco-Delgado, Serum free light chain reference intervals in an Optilite and their influence on clinical guidelines. Clinical Biochemistry, 2021. 92: p. 54-60."

283 "Morrow, A.J., et al., Rationale and design of the Medical Research Council's Precision Medicine with Zibotentan in Microvascular Angina (PRIZE) trial. American Heart Journal, 2020. 229: p. 70-80."

284 "Mountford, S.J., et al., Application of a Sulfoxonium Ylide Electrophile to Generate Cathepsin X?Selective Activity-Based Probes. ACS Chemical Biology, 2020. 15(3): p. 718-727."

285 "Muccilli, V., et al., Protein profile of exhaled breath condensate determined by high resolution mass spectrometry. Journal of Pharmaceutical and Biomedical Analysis, 2015. 105: p. 134-149."

286 "Murphy, C.H., et al., Does supplementation with leucine-enriched protein alone and in combination with fish-oil-derived n-3 PUFA affect muscle mass, strength, physical performance, and muscle protein synthesis in well-nourished older adults? A randomized, double-blind, placebo-controlled trial. Am J Clin Nutr, 2021. 113(6): p. 1411-1427."

287 "Naito, S., et al., Comparison of nephrotoxicity between two gadolinium-contrasts, gadodiamide and gadopentetate in patients with mildly diminished renal failure. J Toxicol Sci, 2017. 42(3): p. 379-384."

288 "Nakamura, A., et al., Contrast between innovator drug- and generic drug-induced renal dysfunction on coronary angiography (CONTRAST study). Heart Vessels, 2014. 29(5): p. 603-10."

289 "Nakamura, N., et al., Transcript profiling in the testes and prostates of postnatal day 30 Sprague-Dawley rats exposed prenatally and lactationally to 2-hydroxy-4-methoxybenzophenone. Reproductive Toxicology, 2018. 82: p. 111-123."

290 "Nassirpour, R., S.K. Ramaiah, and L.O. Whiteley, Nephron segment specific microRNA biomarkers of pre-clinical drug-induced renal toxicity: Opportunities and challenges. Toxicology and Applied Pharmacology, 2016. 312: p. 34-41."

291 "Nasu, T., et al., A genome-wide association study for highly sensitive cardiac troponin T levels identified a novel genetic variation near a RBAK–ZNF890P locus in the Japanese general population. International Journal of Cardiology, 2021. 329: p. 186-191."

292 "Nawa, T., et al., Continuous intravenous infusion of nicorandil for 4 hours before and 24 hours after percutaneous coronary intervention protects against contrast-induced nephropathy in patients with poor renal function. Int J Cardiol, 2015. 195: p. 228-34."

293 "Nedelkov, D. and R.W. Nelson, Analysis of native proteins from biological fluids by biomolecular interaction analysis mass spectrometry (BIA/MS): exploring the limit of detection, identification of non-specific binding and detection of multi-protein complexes. Biosensors and Bioelectronics, 2001. 16(9): p. 1071-1078."

294 "Nedelkov, D., et al., Investigation of Human Protein Variants and Their Frequency in the General Population*. Molecular & Cellular Proteomics, 2007. 6(7): p. 1183-1187."

295 "Ng, T.M.H., et al., Tolvaptan vs. furosemide-based diuretic regimens in patients hospitalized for heart failure with hyponatremia (AQUA-AHF). ESC Heart Fail, 2020. 7(4): p. 1927-1934."

296 "Nissen, A., et al., Expanding the bovine milk proteome through extensive fractionation. Journal of Dairy Science, 2013. 96(12): p. 7854-7866."

297 "Niu, X., et al., Harmine mitigates LPS-induced acute kidney injury through inhibition of the TLR4-NF-κB/NLRP3 inflammasome signalling pathway in mice. European Journal of Pharmacology, 2019. 849: p. 160-169."

298 "Nocera, A.L., et al., Cystatin SN is a potent upstream initiator of epithelial-derived type 2 inflammation in chronic rhinosinusitis. Journal of Allergy and Clinical Immunology, 2022. 150(4): p. 872-881."

299 "Nordon, I., et al., The role of proteomic research in vascular disease. Journal of Vascular Surgery, 2009. 49(6): p. 1602-1612."

300 "Nusair, S.D., et al., Evaluation of orellanine-induced toxicity from the mushroom Cortinarius orellanus and the antagonistic effect of Petroselinum crispum. Toxicon, 2022. 214: p. 1-7."

301 "Ohayon, L., X. Zhang, and P. Dutta, The role of extracellular vesicles in regulating local and systemic inflammation in cardiovascular disease. Pharmacological Research, 2021. 170: p. 105692."

302 "Ohman, M., et al., Biochemical effects of consumption of eggs containing omega-3 polyunsaturated fatty acids. Ups J Med Sci, 2008. 113(3): p. 315-23."

303 "Ordu, S., et al., Effects of ivabradine therapy on heart failure biomarkers. Cardiol J, 2015. 22(5): p. 501-9."

304 "Ozaki, N., et al., Identification of genes involved in gentamicin-induced nephrotoxicity in rats – A toxicogenomic investigation. Experimental and Toxicologic Pathology, 2010. 62(5): p. 555-566."

305 "Ozkan, B., et al., Associations of N-terminal pro-B-type natriuretic peptide, estimated glomerular filtration rate, and mortality in US adults. American Heart Journal, 2023. 264: p. 49-58."

306 "Paisey, R.B., et al., Alstr?m Syndrome, in GeneReviews(?), M.P. Adam, et al., Editors. 1993, University of Washington, Seattle"

307 "Pan, L., et al., Development and internal validation of a prediction model for acute kidney injury following cardiac valve replacement surgery. Int J Cardiol, 2023. 370: p. 345-350."

308 "Panagiotou, A., et al., A Randomized Trial of Recombinant Human C1-Esterase-Inhibitor in the Prevention of Contrast-Induced Kidney?Injury. JACC: Cardiovascular Interventions, 2020. 13(7): p. 833-842."

309 "Panferov, A.S. and S.V. Kotov, [A comparative analysis of simultaneous bilateral versus staged supine mini-percutaneous nephrolithotomy in patients with bilateral kidney stones]. Urologiia, 2019(2): p. 31-35."

310 "Pang, P.S., et al., Rationale, design, and results from RENO-DEFEND 1: a randomized, dose-finding study of the selective A1 adenosine antagonist SLV320 in patients hospitalized with acute heart failure. Am Heart J, 2011. 161(6): p. 1012-23.e3."

311 "Pang, P.S., et al., Use of High-Sensitivity Troponin T to Identify?Patients With Acute Heart Failure at?Lower?Risk for Adverse Outcomes: An Exploratory Analysis From the RELAX-AHF Trial. JACC: Heart Failure, 2016. 4(7): p. 591-599."

312 "Parada-Cruz, B., et al., Inflammation- and cancer-related microRNAs in rat renal cortex after subchronic exposure to fluoride. Chemico-Biological Interactions, 2023. 379: p. 110519."

313 "Parikh, C.R., et al., Urinary IL-18 is an early predictive biomarker of acute kidney injury after cardiac surgery. Kidney International, 2006. 70(1): p. 199-203."

314 "Park, C., et al., Nicardipine Effects on Renal Function During Spine Surgery. Clin Spine Surg, 2017. 30(7): p. E954-e958."

315 "Park, J.S., et al., Application of cystatin C reduction ratio to high-flux hemodialysis as an alternative indicator of the clearance of middle molecules. Korean J Intern Med, 2010. 25(1): p. 77-81."

316 "Parry, S., et al., Cervicovaginal fluid proteomic analysis to identify potential biomarkers for preterm birth. American Journal of Obstetrics and Gynecology, 2020. 222(5): p. 493.e1-493.e13."

317 "Penk, J., et al., Furosemide response predicts acute kidney injury in children after cardiac surgery. The Journal of Thoracic and Cardiovascular Surgery, 2019. 157(6): p. 2444-2451."

318 "Percy, A.J., et al., Advances in multiplexed MRM-based protein biomarker quantitation toward clinical utility. Biochimica et Biophysica Acta (BBA) - Proteins and Proteomics, 2014. 1844(5): p. 917-926."

319 "Perera, R., et al., Programme Grants for Applied Research, in Long-term monitoring in primary care for chronic kidney disease and chronic heart failure: a multi-method research programme. 2021, NIHR Journals Library"

320 "Pe?i?, I., et al., Identification and validation of six proteins as marker for endemic nephropathy. Journal of Proteomics, 2011. 74(10): p. 1994-2007."

321 "Pieragostino, D., et al., Pre-analytical factors in clinical proteomics investigations: Impact of ex vivo protein modifications for multiple sclerosis biomarker discovery. Journal of Proteomics, 2010. 73(3): p. 579-592."

322 "Ponikowski, P. and E.A. Jankowska, Pathogenesis and Clinical Presentation of Acute Heart Failure. Revista Espa?ola de Cardiología (English Edition), 2015. 68(4): p. 331-337."

323 "P?ss, J., et al., Angiopoietin-2 and outcome in patients with acute decompensated heart failure. Clin Res Cardiol, 2015. 104(5): p. 380-7."

324 "Post, A., et al., Fibroblast growth factor 21 and protein energy wasting in hemodialysis patients. Clinical Nutrition, 2021. 40(6): p. 4216-4224."

325 "Potok, O.A., et al., The Difference Between Cystatin C- and Creatinine-Based Estimated GFR and Associations With Frailty and Adverse Outcomes: A Cohort Analysis of the Systolic Blood Pressure Intervention Trial (SPRINT). Am J Kidney Dis, 2020. 76(6): p. 765-774."

326 "Prado, Y., et al., Procoagulant phenotype induced by oxidized high-density lipoprotein associates with acute kidney injury and death. Thrombosis Research, 2023. 223: p. 7-23."

327 "Przybylowski, P., J. Malyszko, and J.S. Malyszko, Copeptin in Heart Transplant Recipients Depends on Kidney Function and Intraventricular Septal Thickness. Transplantation Proceedings, 2010. 42(5): p. 1808-1811."

328 "Puolakka, P.A., et al., The effect of parecoxib on kidney function at laparoscopic hysterectomy. Ren Fail, 2009. 31(4): p. 284-9."

329 "Qiu, J., et al., Invasion suppressor cystatin E/M (CST6): high-level cell type-specific expression in normal brain and epigenetic silencing in gliomas. Laboratory Investigation, 2008. 88(9): p. 910-925."

330 "Quintana, M., et al., Inter-individual variability of protein patterns in saliva of healthy adults. Journal of Proteomics, 2009. 72(5): p. 822-830."

331 "Raaijmakers, A., et al., Does Extremely Low Birth Weight Predispose to Low-Renin Hypertension? Hypertension, 2017. 69(3): p. 443-449."

332 "Rajpal, S. and P. Mishra, Next generation biosensors employing molecularly imprinted polymers as sensing elements for in vitro diagnostics. Biosensors and Bioelectronics: X, 2022. 11: p. 100201."

333 "Rasking, L., et al., Ambient black carbon reaches the kidneys. Environment International, 2023. 177: p. 107997."

334 "Razavi, M., et al., High precision quantification of human plasma proteins using the automated SISCAPA Immuno-MS workflow. New Biotechnology, 2016. 33(5, Part A): p. 494-502."

335 "Rebholz, C.M., et al., Risk of ESRD and Mortality Associated With Change in Filtration Markers. American Journal of Kidney Diseases, 2017. 70(4): p. 551-560."

336 "Ricci, Z., et al., Furosemide versus ethacrynic acid in pediatric patients undergoing cardiac surgery: a randomized controlled trial. Crit Care, 2015. 19(1): p. 2."

337 "Rigas, J., Quivering Kidneys: Minimally Invasive Tests for Renal Pathology? (The few, the new, and not-so-new tried and true). Advances in Small Animal Medicine and Surgery, 2016. 29(7): p. 1-3."

338 "Righetti, P.G., et al., Proteome analysis in the clinical chemistry laboratory: Myth or reality? Clinica Chimica Acta, 2005. 357(2): p. 123-139."

339 "Rinde, N.B., et al., Nitric Oxide Precursors and Dimethylarginines as Risk Markers for Accelerated Measured GFR Decline in the General Population. Kidney International Reports, 2023. 8(4): p. 818-826."

340 "Robles, N.R., et al., Sacubitril-Valsartan Improves Anemia of Cardiorenal Syndrome (CRS). Cardiovasc Hematol Agents Med Chem, 2021. 19(1): p. 93-97."

341 "Rocha, A., et al., Transthyretin (ATTR) amyloidosis nephropathy: lessons from a TTR stabilizer molecule. Amyloid, 2017. 24(sup1): p. 81-82."

342 "Romero, R., et al., The maternal plasma proteome changes as a function of gestational age in normal pregnancy: a longitudinal study. American Journal of Obstetrics and Gynecology, 2017. 217(1): p. 67.e1-67.e21."

343 "Ronkainen, J., H. Autio-Harmainen, and M. Nuutinen, Cyclosporin A for the treatment of severe Henoch-Sch?nlein glomerulonephritis. Pediatr Nephrol, 2003. 18(11): p. 1138-42."

344 "Roque, D.R., et al., Association between differential gene expression and body mass index among endometrial cancers from The Cancer Genome Atlas Project. Gynecologic Oncology, 2016. 142(2): p. 317-322."

345 "Ruff, C.T., et al., North American Thrombosis Forum, AF Action Initiative Consensus Document. The American Journal of Medicine, 2016. 129(5, Supplement): p. S1-S29."

346 "Ruiz Ortega, R.A., L. Manzano, and M. Montero-Pérez-Barquero, Diagnóstico de la insuficiencia cardíaca aguda y relevancia de los biomarcadores en pacientes de edad avanzada. Medicina Clínica, 2014. 142: p. 20-25."

347 "Sabbah, H.N., et al., Effects of Angiotensin-Neprilysin Inhibition in Canines with Experimentally Induced Cardiorenal Syndrome. Journal of Cardiac Failure, 2020. 26(11): p. 987-997."

348 "Saitou, M., et al., Functional Specialization of Human Salivary Glands and Origins of Proteins Intrinsic to Human Saliva. Cell Reports, 2020. 33(7): p. 108402."

349 "Salman, S., et al., Pharmacokinetic properties of conventional and double-dose sulfadoxine-pyrimethamine given as intermittent preventive treatment in infancy. Antimicrob Agents Chemother, 2011. 55(4): p. 1693-700."

350 "Sanders-van Wijk, S., et al., Circulating biomarkers of distinct pathophysiological pathways in heart failure with preserved vs. reduced left ventricular ejection fraction. Eur J Heart Fail, 2015. 17(10): p. 1006-14."

351 "Sandilands, E.A., et al., Mechanisms for an effect of acetylcysteine on renal function after exposure to radio-graphic contrast material: study protocol. BMC Clin Pharmacol, 2012. 12: p. 3."

352 "Santin, A.D., et al., Gene expression profiles of primary HPV16- and HPV18-infected early stage cervical cancers and normal cervical epithelium: identification of novel candidate molecular markers for cervical cancer diagnosis and therapy. Virology, 2005. 331(2): p. 269-291."

353 "Saraiva, J.R., et al., Gingivitis in cattle and supplemental protein diet: Insights from proteomic analysis. Journal of Proteomics, 2023. 282: p. 104913."

354 "Sato, H., et al., Evaluation of the Suitability of Dried Saliva Spots for In-Depth Proteome Analyses for Clinical Applications. Journal of Proteome Research, 2022. 21(5): p. 1340-1348."

355 "Sawai, S., et al., Serum levels of complement C4 fragments correlate with disease activity in multiple sclerosis: Proteomic analysis. Journal of Neuroimmunology, 2010. 218(1): p. 112-115."

356 "Schaaij-Visser, T.B.M., et al., Comparative proteome analysis to explore p53 pathway disruption in head and neck carcinogenesis. Journal of Proteomics, 2009. 72(5): p. 803-814."

357 "Schattenberg, J.M., et al., A randomized placebo-controlled trial of elafibranor in patients with primary biliary cholangitis and incomplete response to UDCA. Journal of Hepatology, 2021. 74(6): p. 1344-1354."

358 "Scheppach, J.B., et al., Association of Kidney Function Measures With Signs of Neurodegeneration and Small Vessel Disease on Brain Magnetic Resonance Imaging: The Atherosclerosis Risk in Communities (ARIC) Study. American Journal of Kidney Diseases, 2023. 81(3): p. 261-269.e1."

359 "Schipper, R., et al., SELDI-TOF-MS of saliva: Methodology and pre-treatment effects. Journal of Chromatography B, 2007. 847(1): p. 45-53."

360 "Schmit, P.-O., et al., Towards a routine application of Top-Down approaches for label-free discovery workflows. Journal of Proteomics, 2018. 175: p. 12-26."

361 "Schuetz, A.N., et al., Molecular Classification of Renal Tumors by Gene Expression Profiling. The Journal of Molecular Diagnostics, 2005. 7(2): p. 206-218."

362 "Schützer, K.M., et al., Reversible elevations of serum creatinine levels but no effect on glomerular filtration during treatment with the direct thrombin inhibitor AZD0837. Eur J Clin Pharmacol, 2010. 66(9): p. 903-10."

363 "Scully, P. and D. Goldsmith, The management of end-stage heart failure and reducing the risk of cardiorenal syndrome. Clinical Medicine, 2013. 13(6): p. 610-613."

364 "Seelhammer, T.G., et al., Kinetic estimated glomerular filtration rate and acute kidney injury in cardiac surgery patients. Journal of Critical Care, 2016. 31(1): p. 249-254."

365 "Serrao, S., et al., Top-Down Proteomics of Human Saliva Discloses Significant Variations of the Protein Profile in Patients with Mastocytosis. Journal of Proteome Research, 2020. 19(8): p. 3238-3253."

366 "Sewell, M.D.E., et al., Associations between major psychiatric disorder polygenic risk scores and blood-based markers in UK biobank. Brain, Behavior, and Immunity, 2021. 97: p. 32-41."

367 "Sezai, A., et al., Comparison of febuxostat and allopurinol for hyperuricemia in cardiac surgery patients (NU-FLASH Trial). Circ J, 2013. 77(8): p. 2043-9."

368 "Shah, R.V., et al., Effect of admission oral diuretic dose on response to continuous versus bolus intravenous diuretics in acute heart failure: an analysis from diuretic optimization strategies in acute heart failure. Am Heart J, 2012. 164(6): p. 862-8."

369 "Shalia, K.K., et al., Levels of cathepsins in acute myocardial infarction. Indian Heart Journal, 2012. 64(3): p. 290-294."

370 "Shamsuddin, S.H., et al., Reagentless Affimer- and antibody-based impedimetric biosensors for CEA-detection using a novel non-conducting polymer. Biosensors and Bioelectronics, 2021. 178: p. 113013."

371 "Shariati, S., A. Ghaffarinejad, and E. Omidinia, Early detection of multiple sclerosis (MS) as a neurodegenerative disease using electrochemical nano-aptasensor. Microchemical Journal, 2022. 178: p. 107358."

372 "Sharma, R., et al., Label-free electrochemical impedance biosensor to detect human interleukin-8 in serum with sub-pg/ml sensitivity. Biosensors and Bioelectronics, 2016. 80: p. 607-613."

373 "Shashikumar, N.G., et al., Global proteomic analysis of water buffalo (Bubalus bubalis) saliva at different stages of estrous cycle using high throughput mass spectrometry. Theriogenology, 2018. 110: p. 52-60."

374 "Shawwa, K., et al., Heterogeneity in Acute Kidney Injury Management in Critically Ill Patients: National Survey. The Journal for Nurse Practitioners, 2023. 19(10): p. 104776."

375 "Shi, Q.S., et al., Microvascular activation and exocytosis after exposure to the serum from mismatched recipients by using donor microvascular cultures. Transplant Immunology, 2024. 82: p. 101963."

376 "Shi, Y., et al., Efficacy and Safety of Mizoribine Combined With Tacrolimus in Living Donor Kidney Transplant Recipients: 3-Year Results by a Chinese Single Center Study. Transplant Proc, 2019. 51(5): p. 1337-1342."

377 "Shi, Y., et al., Influence of CYP3A4, CYP3A5 and MDR-1 polymorphisms on tacrolimus pharmacokinetics and early renal dysfunction in liver transplant recipients. Gene, 2013. 512(2): p. 226-231."

378 "Shiba, T., et al., Arteriosclerotic Changes after Intravitreal Injections of Anti-Vascular Endothelial Growth Factor Drugs in Patients with Exudative Age-Related Macular Degeneration. Ophthalmologica, 2016. 235(4): p. 225-32."

379 "Shlipak, M.G., et al., Effect of Structured, Moderate Exercise on Kidney Function Decline in Sedentary Older Adults: An Ancillary Analysis of the LIFE Study Randomized Clinical Trial. JAMA Intern Med, 2022. 182(6): p. 650-659."

380 "Siavashpour, A., et al., Poly (ADP-Ribose) polymerase-1 (PARP-1) overactivity plays a pathogenic role in bile acids-induced nephrotoxicity in cholestatic rats. Toxicology Letters, 2020. 330: p. 144-158."

381 "?íma, M., et al., Adherence with perindopril therapy: a pilot study using therapeutic drug monitoring of perindoprilat and an evaluation of the clearance estimation. Int J Clin Pharm, 2017. 39(5): p. 1095-1100."

382 "Singhal, G., V. Pathak, and M. Kumar, Incidence of contrast induced acute kidney injury in patients undergoing percutaneous coronary intervention in North Indian population. Journal of Indian College of Cardiology, 2017. 7(4): p. 143-148."

383 "Sirota, J.C., et al., Urine IL-18, NGAL, IL-8 and serum IL-8 are biomarkers of acute kidney injury following liver transplantation. BMC Nephrol, 2013. 14: p. 17."

384 "Sitras, V., et al., Differential Placental Gene Expression in Severe Preeclampsia. Placenta, 2009. 30(5): p. 424-433."

385 "Sleat, D.E., et al., Identification and Validation of Mannose 6-Phosphate Glycoproteins in Human Plasma Reveal a Wide Range of Lysosomal and Non-lysosomal Proteins*. Molecular & Cellular Proteomics, 2006. 5(10): p. 1942-1956."

386 "Snips?yr, M.G., et al., Towards identification of novel putative biomarkers for infective endocarditis by serum proteomic analysis. International Journal of Infectious Diseases, 2020. 96: p. 73-81."

387 "Soetaert, A., et al., Daphnia magna and ecotoxicogenomics: Gene expression profiles of the anti-ecdysteroidal fungicide fenarimol using energy-, molting- and life stage-related cDNA libraries. Chemosphere, 2007. 67(1): p. 60-71."

388 "Song, M.-f., et al., Sema 3A as a biomarker of the activated mTOR pathway during hexavalent chromium-induced acute kidney injury. Toxicology Letters, 2018. 299: p. 226-235."

389 "Song, T., et al., Comparison of the nephrotoxic effects of iodixanol versus iohexol in patients with chronic heart failure undergoing coronary angiography or angioplasty. J Interv Cardiol, 2017. 30(3): p. 281-285."

390 "Srirajaskanthan, R., et al., Identification of Mac-2-binding Protein as a Putative Marker of Neuroendocrine Tumors from the Analysis of Cell Line Secretomes*. Molecular & Cellular Proteomics, 2010. 9(4): p. 656-666."

391 "Stavro, P.M., et al., Long-term intake of North American ginseng has no effect on 24-hour blood pressure and renal function. Hypertension, 2006. 47(4): p. 791-6."

392 "Steenvoorden, T.S., et al., Alkaline phosphatase to treat ischaemia-reperfusion injury in living-donor kidney transplantation: APhIRI I feasibility pilot study. Br J Clin Pharmacol, 2023. 89(12): p. 3629-3636."

393 "Steubl, D., et al., Association of Serum Uromodulin With ESKD and Kidney Function Decline in the Elderly: The Cardiovascular Health Study. American Journal of Kidney Diseases, 2019. 74(4): p. 501-509."

394 "Steubl, D., et al., Influence of high-flux hemodialysis and hemodiafiltration on serum C-terminal agrin fragment levels in end-stage renal disease patients. Translational Research, 2014. 164(5): p. 392-399."

395 "Stravers, C.S., et al., Multiplex body fluid identification using surface plasmon resonance imaging with principal component analysis. Sensors and Actuators B: Chemical, 2019. 283: p. 355-362."

396 "Suleimenova, A., et al., Bacterial nanocellulose membrane as novel substrate for biomimetic structural color materials: Application to lysozyme sensing. Biosensors and Bioelectronics: X, 2023. 13: p. 100310."

397 "Sun, D., et al., Analysis of protein expression changes of the Vero E6 cells infected with classic PEDV strain CV777 by using quantitative proteomic technique. Journal of Virological Methods, 2015. 218: p. 27-39."

398 "Sunayama, T., et al., Prognostic value of estimating appendicular muscle mass in heart failure using creatinine/cystatin C. Nutrition, Metabolism and Cardiovascular Diseases, 2023. 33(9): p. 1733-1739."

399 "Takemura, Y., et al., Epigenetic clock analysis in methamphetamine dependence. Psychiatry Research, 2022. 317: p. 114901."

400 "Takiar, R., et al., The associations of 25-hydroxyvitamin D levels, vitamin D binding protein gene polymorphisms, and race with risk of incident fracture-related hospitalization: Twenty-year follow-up in a bi-ethnic cohort (the ARIC Study). Bone, 2015. 78: p. 94-101."

401 "Tang, W., Y. Cao, and X. Ma, Novel prognostic prediction model constructed through machine learning on the basis of methylation-driven genes in kidney renal clear cell carcinoma. Bioscience Reports, 2020. 40(7)."

402 "Tang, X., et al., Proteomics-based analysis of potential therapeutic targets in patients with peritoneal dialysis-associated peritonitis. Biochimica et Biophysica Acta (BBA) - Proteins and Proteomics, 2022. 1870(7): p. 140796."

403 "Tangri, N., et al., Changes in dietary protein intake has no effect on serum cystatin C levels independent of the glomerular filtration rate. Kidney Int, 2011. 79(4): p. 471-7."

404 "Tans, R., et al., Affimers as an alternative to antibodies for protein biomarker enrichment. Protein Expression and Purification, 2020. 174: p. 105677."

405 "Taraskin, A.S., et al., A novel method for multiplex protein biomarker analysis of human serum using quantitative MALDI mass spectrometry. Journal of Pharmaceutical and Biomedical Analysis, 2022. 210: p. 114575."

406 "Taslimi, Y., et al., Profiling inflammatory response in lesions of cutaneous leishmaniasis patients using a non-invasive sampling method combined with a high-throughput protein detection assay. Cytokine, 2020. 130: p. 155056."

407 "Teunissen, C.E., et al., Biobanking of CSF: International standardization to optimize biomarker development. Clinical Biochemistry, 2014. 47(4): p. 288-292."

408 "Thielmann, M., et al., Teprasiran, a Small Interfering RNA, for the Prevention of Acute Kidney Injury in High-Risk Patients Undergoing Cardiac Surgery: A Randomized Clinical Study. Circulation, 2021. 144(14): p. 1133-1144."

409 "Thorlacius, E.M., et al., The Effect of Levosimendan Versus Milrinone on the Occurrence Rate of Acute Kidney Injury Following Congenital Heart Surgery in Infants: A Randomized Clinical Trial. Pediatr Crit Care Med, 2019. 20(10): p. 947-956."

410 "Thyagarajan, B., et al., Analytical and biological variability in biomarker measurement in the Hispanic Community Health Study/Study of Latinos. Clinica Chimica Acta, 2016. 463: p. 129-137."

411 "Ti, Y.X., Z.X. Pan, and C. Wu, [Intervention of astragalus injection on the kidney injury after cardiopulmonary bypass of infants with congenital heart disease]. Zhongguo Zhong Xi Yi Jie He Za Zhi, 2011. 31(5): p. 631-4."

412 "Tiberti, N., et al., Discovery and Verification of Osteopontin and Beta-2-microglobulin as Promising Markers for Staging Human African Trypanosomiasis*. Molecular & Cellular Proteomics, 2010. 9(12): p. 2783-2795."

413 "Tierney, C., et al., Saliva-omics in plasma cell disorders- Proof of concept and potential as a non-invasive tool for monitoring disease burden. Journal of Proteomics, 2021. 231: p. 104015."

414 "Tijani, A.S., D.O. Olori, and E.O. Farombi, Manganese abated indomethacin-induced gastrohepatorenal toxicities in Rats via suppression of oxidative stress, polyamine catabolism, inflammation and activation of Caspase-3. Advances in Redox Research, 2023. 8: p. 100070."

415 "Topaz, G., et al., Impaired renal function is associated with adverse outcomes in patients with chest pain discharged from internal medicine wards. European Journal of Internal Medicine, 2018. 53: p. 57-61."

416 "Topletz-Erickson, A.R., et al., Tucatinib Inhibits Renal Transporters OCT2 and MATE Without Impacting Renal Function in Healthy Subjects. J Clin Pharmacol, 2021. 61(4): p. 461-471."

417 "Tran, B.Q., et al., Proteomic Characterization of Dermal Interstitial Fluid Extracted Using a Novel Microneedle-Assisted Technique. Journal of Proteome Research, 2018. 17(1): p. 479-485."

418 "Tsalik, E.L., et al., Renal systems biology of patients with systemic inflammatory response syndrome. Kidney International, 2015. 88(4): p. 804-814."

419 "Vaes, B., et al., The impact of confounders on the test performance of natriuretic peptides for cardiac dysfunction in subjects aged 80 and older. Peptides, 2012. 38(1): p. 118-26."

420 "van der Burgh, A.C., et al., Sex Differences in the Association Between Serum Testosterone and Kidney Function in the General Population. Kidney International Reports, 2023. 8(7): p. 1342-1351."

421 "van?de?Luitgaarden, I.A.T., et al., Urinary Ethyl Glucuronide Can Be Used as a Biomarker of Habitual Alcohol Consumption in the General Population. The Journal of Nutrition, 2019. 149(12): p. 2199-2205."

422 "Vashist, S.K., A sub-picogram sensitive rapid chemiluminescent immunoassay for the detection of human fetuin A. Biosensors and Bioelectronics, 2013. 40(1): p. 297-302."

423 "Vashist, S.K., E.M. Schneider, and J.H.T. Luong, Rapid sandwich ELISA-based in vitro diagnostic procedure for the highly-sensitive detection of human fetuin A. Biosensors and Bioelectronics, 2015. 67: p. 73-78."

424 "Vashist, S.K., M. Saraswat, and H. Holth?fer, Development of a Rapid Sandwich Enzyme Linked Immunoassay Procedure for the Highly Sensitive Detection of Human Lipocalin-2/NGAL. Procedia Chemistry, 2012. 6: p. 141-148."

425 "Velleca, A., et al., The International Society for Heart and Lung Transplantation (ISHLT) guidelines for the care of heart transplant recipients. The Journal of Heart and Lung Transplantation, 2023. 42(5): p. e1-e141."

426 "Venugopal, V., et al., Occupational Heat Stress and Kidney Health in Salt Pan Workers. Kidney International Reports, 2023. 8(7): p. 1363-1372."

427 "Vitorino, R., et al., Evaluation of different extraction procedures for salivary peptide analysis. Talanta, 2012. 94: p. 209-215."

428 "Voors, A.A., et al., Renal effects of the angiotensin receptor neprilysin inhibitor LCZ696 in patients with heart failure and preserved ejection fraction. Eur J Heart Fail, 2015. 17(5): p. 510-7."

429 "Voors, A.A., et al., Safety and efficacy of the partial adenosine A1 receptor agonist neladenoson bialanate in patients with chronic heart failure with reduced ejection fraction: a phase IIb, randomized, double-blind, placebo-controlled trial. Eur J Heart Fail, 2019. 21(11): p. 1426-1433."

430 "Wallbach, M., et al., Impact of baroreflex activation therapy on renal function--a pilot study. Am J Nephrol, 2014. 40(4): p. 371-80."

431 "Wallimann, T., U. Riek, and M. M?ddel, Intradialytic creatine supplementation: A scientific rationale for improving the health and quality of life of dialysis patients. Medical Hypotheses, 2017. 99: p. 1-14."

432 "Wan, S.H., et al., Differential Response to Low-Dose Dopamine or Low-Dose Nesiritide in Acute Heart Failure With Reduced or Preserved Ejection Fraction: Results From the ROSE AHF Trial (Renal Optimization Strategies Evaluation in Acute Heart Failure). Circ Heart Fail, 2016. 9(8)."

433 "Wang, H., et al., Intact-protein-based High-resolution Three-dimensional Quantitative Analysis System for Proteome Profiling of Biological Fluids*. Molecular & Cellular Proteomics, 2005. 4(5): p. 618-625."

434 "Wang, H., et al., Population Pharmacokinetics and Dosing Simulations of Ceftazidime in Chinese Neonates. Journal of Pharmaceutical Sciences, 2018. 107(5): p. 1416-1422."

435 "Wang, K., et al., Olfaction and kidney function in community-dwelling older adults. PLoS One, 2022. 17(2): p. e0264448."

436 "Wang, M.-L., et al., Estrogen profile- and pharmacogenetics-based lamotrigine dosing regimen optimization: Recommendations for pregnant women with epilepsy. Pharmacological Research, 2021. 169: p. 105610."

437 "Wang, S., et al., Massive Proteinuria-Induced Injury of Tubular Epithelial Cells in Nephrotic Syndrome is Not Exacerbated by Furosemide. Cell Physiol Biochem, 2018. 45(4): p. 1700-1706."

438 "Webb, N.J., et al., Losartan and enalapril are comparable in reducing proteinuria in children. Kidney Int, 2012. 82(7): p. 819-26."

439 "Weeraphan, C., et al., Effective enrichment of cholangiocarcinoma secretomes using the hollow fiber bioreactor culture system. Talanta, 2012. 99: p. 294-301."

440 "Wennberg, A.M.V., et al., Trajectories of plasma IGF-1, IGFBP-3, and their ratio in the Mayo Clinic Study of Aging. Experimental Gerontology, 2018. 106: p. 67-73."

441 "Westphal, S., J. Dierkes, and C. Luley, Effects of fenofibrate and gemfibrozil on plasma homocysteine. Lancet, 2001. 358(9275): p. 39-40."

442 "Wijerathna, T.M., et al., Cellular injury leading to oxidative stress in acute poisoning with potassium permanganate/oxalic acid, paraquat, and glyphosate surfactant herbicide. Environmental Toxicology and Pharmacology, 2020. 80: p. 103510."

443 "Williamson, J.C., et al., A proteomics approach to the identification of biomarkers for psoriasis utilising keratome biopsy. Journal of Proteomics, 2013. 94: p. 176-185."

444 "Windhausen, F., et al., Cystatin C for enhancement of risk stratification in non-ST elevation acute coronary syndrome patients with an increased troponin T. Clin Chem, 2009. 55(6): p. 1118-25."

445 "Woo, J., et al., Effects of IL-1β inhibition on anemia and clonal hematopoiesis in the randomized CANTOS trial. Blood Advances, 2023. 7(24): p. 7471-7484."

446 "Wozniak, J.M., et al., Mortality Risk Profiling of Staphylococcus aureus Bacteremia by Multi-omic Serum Analysis Reveals Early Predictive and Pathogenic Signatures. Cell, 2020. 182(5): p. 1311-1327.e14."

447 "Wright, C.S., et al., Whey Protein Supplementation and Higher Total Protein Intake Do Not Influence Bone Quantity in Overweight and Obese Adults Following a 36-Week Exercise and Diet Intervention12. The Journal of Nutrition, 2017. 147(2): p. 179-186."

448 "Wu, Y., et al., AIM2 inflammasome contributes to aldosterone-induced renal injury via endoplasmic reticulum stress. Clinical Science, 2022. 136(1): p. 103-120."

449 "Wunnapuk, K., et al., Kidney biomarkers in MCPA-induced acute kidney injury in rats: Reduced clearance enhances early biomarker performance. Toxicology Letters, 2014. 225(3): p. 467-478."

450 "Würtz, M., et al., Influence of renal function and platelet turnover on the antiplatelet effect of aspirin. Thromb Res, 2012. 129(4): p. 434-40."

451 "Xia, T., et al., Analysis of amino acids in human blood using UHPLC-MS/MS: Potential interferences of storage time and vacutainer tube in pre-analytical procedure. Clinical Biochemistry, 2016. 49(18): p. 1372-1378."

452 "Xiao, Y., et al., Gene expression profiling of bone marrow stromal cells from juvenile, adult, aged and osteoporotic rats: With an emphasis on osteoporosis. Bone, 2007. 40(3): p. 700-715."

453 "Xu, L., et al., C9orf72 poly(PR) aggregation in nucleus induces ALS/FTD-related neurodegeneration in cynomolgus monkeys. Neurobiology of Disease, 2023. 184: p. 106197."

454 "Yaghubi, E., et al., Effects of l-carnitine supplementation on cardiovascular and bone turnover markers in patients with pemphigus vulgaris under corticosteroids treatment: A randomized, double-blind, controlled trial. Dermatol Ther, 2019. 32(5): p. e13049."

455 "Yang, D., et al., Development and validation of a predictive model for acute kidney injury in patients with moderately severe and severe acute pancreatitis. Clin Exp Nephrol, 2022. 26(8): p. 770-787."

456 "Yang, L., et al., Extensive cytokine analysis in synovial fluid of osteoarthritis patients. Cytokine, 2021. 143: p. 155546."

457 "Yang, S.-M., et al., Dielectrophoresis assisted high-throughput detection system for multiplexed immunoassays. Biosensors and Bioelectronics, 2021. 180: p. 113148."

458 "Yim, E.-K., et al., Proteomic analysis of ursolic acid-induced apoptosis in cervical carcinoma cells. Cancer Letters, 2006. 235(2): p. 209-220."

459 "Yin, G.N., et al., Neuronal pentraxin receptor in cerebrospinal fluid as a potential biomarker for neurodegenerative diseases. Brain Research, 2009. 1265: p. 158-170."

460 "Yin, Z., et al., A label-free electrochemical immunosensor based on PdPtCu@BP bilayer nanosheets for point-of-care kidney injury molecule-1 testing. Journal of Electroanalytical Chemistry, 2022. 917: p. 116420."

461 "Yoo, Y.C., et al., Anesthetics influence the incidence of acute kidney injury following valvular heart surgery. Kidney Int, 2014. 86(2): p. 414-22."

462 "Zaghlool, S.S., et al., Restoring glomerular filtration rate by sulforaphane modulates ERK1/2/JNK/p38MAPK, IRF3/iNOS, Nrf2/HO-1 signaling pathways against folic acid-induced acute renal injury in rats. International Immunopharmacology, 2023. 123: p. 110777."

463 "Zhang, C.Q., et al., [Jinshuibao capsule combined losartan potassium intervened early renal damage of hypertension patients of yin and yang deficiency: a clinical research]. Zhongguo Zhong Xi Yi Jie He Za Zhi, 2013. 33(6): p. 731-5."

464 "Zhang, G., et al., Lead exposure induced developmental nephrotoxicity in Japanese quail (Coturnix japonica) via oxidative stress-based PI3K/AKT pathway inhibition and NF-κB pathway activation. Comparative Biochemistry and Physiology Part C: Toxicology & Pharmacology, 2023. 268: p. 109599."

465 "Zhang, J.C., et al., [The effects of joint administration of 6% hydroxyethyl starch 130/0.4 and high-volume hemofiltration on patients with acute lung injury and acute kidney injury]. Zhongguo Wei Zhong Bing Ji Jiu Yi Xue, 2011. 23(12): p. 755-8."

466 "Zhang, X., et al., Analytical and biological validation of a multiplex immunoassay for acute kidney injury biomarkers. Clinica Chimica Acta, 2013. 415: p. 88-93."

467 "Zhang, X., et al., Efficacy of nicorandil on the prevention of contrast-induced nephropathy in patients with coronary heart disease undergoing percutaneous coronary intervention. Coron Artery Dis, 2020. 31(3): p. 284-288."

468 "Zhang, X., et al., Enhanced external counterpulsation: A new method to alleviate contrast-induced acute kidney injury. Contemp Clin Trials, 2022. 113: p. 106653."

469 "Zhang, Y., et al., Associations of serum cystatin C and its change with new-onset cardiovascular disease in Chinese general population. Nutrition, Metabolism and Cardiovascular Diseases, 2022. 32(8): p. 1963-1971."

470 "Zhang, Y., et al., Urinary-derived extracellular vesicle microRNAs as non‐invasive diagnostic biomarkers for early-stage renal cell carcinoma. Clinica Chimica Acta, 2024. 552: p. 117672."

471 "Zhao, C., et al., The lncRNA MALAT1 participates in regulating coronary slow flow endothelial dysfunction through the miR-181b-5p–MEF2A–ET-1 axis. Vascular Pharmacology, 2021. 138: p. 106841."

472 "Zhao, X., et al., Predictive value of 4-Hydroxyglutamate and miR-149-5p on eclampsia. Experimental and Molecular Pathology, 2021. 119: p. 104618."

473 "Zheng, Z., et al., The HDAC2/SP1/miR-205 feedback loop contributes to tubular epithelial cell extracellular matrix production in diabetic kidney disease. Clinical Science, 2022. 136(3): p. 223-238."

474 "Zhou, D., et al., Cytidine monophosphate kinase is inhibited by the TGF-β signalling pathway through the upregulation of miR-130b-3p in human epithelial ovarian cancer. Cellular Signalling, 2017. 35: p. 197-207."

475 "Zhou, X., et al., Identification of urinary microRNA biomarkers for detection of gentamicin-induced acute kidney injury in rats. Regulatory Toxicology and Pharmacology, 2016. 78: p. 78-84."

476 "Zhou, X.-H., et al., Novel chinmedomics strategy for discovering effective constituents from ShenQiWan acting on ShenYangXu syndrome. Chinese Journal of Natural Medicines, 2016. 14(8): p. 561-581."

477 "Zhou, Y., et al., Measurement of Organ-Specific and Acute-Phase Blood Protein Levels in Early Lyme Disease. Journal of Proteome Research, 2020. 19(1): p. 346-359."

478 "Zhu, C.Z., et al., Short-term oral gavage administration of adenine induces a model of fibrotic kidney disease in rats. Journal of Pharmacological and Toxicological Methods, 2018. 94: p. 34-43."

479 "Zhu, J., et al., Von Willebrand factor and acute type a aortic dissection——VAD study. International Journal of Surgery Open, 2021. 35: p. 100379."

480 "Zhu, Y., et al., Orally-active, clinically-translatable senolytics restore α-Klotho in mice and humans. eBioMedicine, 2022. 77: p. 103912."

481 "Zou, Z.-P., et al., Biomarker-responsive engineered probiotic diagnoses, records, and ameliorates inflammatory bowel disease in mice. Cell Host & Microbe, 2023. 31(2): p. 199-212.e5."

482 "白小岗, et al., miR-9、miR-214在糖尿病肾病中的表达及临床意义. 临床肾脏病杂志, 2021. 21(12): p. 981-985."

483 "曹丽, et al., COPD患者血清HIF-1α水平与心肾综合征的相关性研究. 宁夏医学杂志, 2016. 38(3): p. 204-206."

484 "陈俊羽, 基于生信分析的钙化性主动脉瓣疾病生物标志物的识别与验证和临床预测模型的构建与验证. 2023."

485 "陈桐, 糖尿病和糖尿病肾病患者血清lncRNA GAS5/miR-21ceRNA调控网络的表达变化及意义. 2017."

486 "陈晓岚, 陈.A.石.A.聂.A.陆.A.刘.A., 外泌体miR-93-5p在慢性肾脏病患者中的表达及临床意义. 中华肾脏病杂志, 2021. 37(10)."

487 "陈笑, et al., 外泌体miR-93-5p在慢性肾脏病患者中的表达及临床意义. 中华肾脏病杂志, 2021. 37(10): p. 835-838."

488 "丁莉, 青海地区心房颤动患者外周血中miR-296-3p的表达研究. 2022."

489 "杜琳, 不同他汀对急性冠脉综合征患者介入术后早期肾功能的影响. 2014."

490 "公帅, et al., p2PSA及其相关指标联合NLR和Cys C对前列腺癌的诊断价值分析. 现代诊断与治疗, 2023. 34(14): p. 2065-2069,2075."

491 "龚裕强, 谢.A.潘.A., miR-29c在慢性肾脏病患者血清中的表达及临床意义研究. 浙江医学, 2019. 41(15)."

492 "侯娟娟, et al., H-FABP、hs-cT nI、Hcy和Cys-C对急性心肌梗死早期诊断的临床评价. 国际检验医学杂志, 2015(15): p. 2170-2171,2174."

493 "贾秋利 and 赵旭敏, 血清miR-21、cys C、sCD40L及sICAM-1水平与糖尿病肾病的相关性分析. 医学临床研究, 2022. 39(8): p. 1197-1201."

494 "靳思思, 靳绵绵, and 张一平, 联合检测血清miR-21和miR-25对2型糖尿病肾病的诊断价值. 华南国防医学杂志, 2020. 34(4): p. 228-231."

495 "李青霖, et al., 自噬和p53凋亡刺激蛋白在大鼠急性肾损伤模型中的表达及早期诊断价值. 中华肾病研究电子杂志, 2017. 6(3): p. 120-126."

496 "李霞, 白.A.王.A.白.A.王.A., miR-9、miR-214在糖尿病肾病中的表达及临床意义. 临床肾脏病杂志, 2021. 21(12)."

497 "梁依, 循环miRNAs在老年2型心肾综合征中的诊断价值. 2019."

498 "刘远辉, miRNA-20a靶向Atg7影响的自噬对造影剂诱导的急性肾损伤的机制研究及新的急性肾损伤危险因素探讨. 2016."

499 "吕春燕, 基于“清浊相干”理论的肾间质纤维化尿外泌体源RNA液体活检指标研究. 2018."

500 "马俊瑞, 前列地尔对非ST段抬高型心肌梗死合并肾功能不全PCI患者的肾脏保护效应. 2014."

501 "马宁, miR--194、TSP--1mRNA通过内质网应激在糖尿病肾脏疾病的相关性研究及机制探讨. 2021."

502 "彭卫华, 非侵入性方法监测ANCA相关血管炎肾损害病情变化的研究. 2020."

503 "桑冉, 血清miRNA-17和miRNA-20a含量对慢性心力衰竭患者危险分层及预后的研究. 2020."

504 "孙云霞, 汪.A.张.A.张.A.杜.A.李.A., 预测庆大霉素、多柔比星和腺嘌呤致大鼠肾损伤的生物标志物. 中国药理学与毒理学杂志, 2015. 29(1)."

505 "覃小玲, 新型肾功能标志物评估万古霉素导致肾毒性的应用研究. 2017."

506 "滕树恩, 造影剂用量与肾小球滤过率比值用于预测PCI术后造影剂肾病发生的临床意义. 2016."

507 "汪俊, 基于超高效液相色谱串联质谱的肾细胞癌患者血清非靶向代谢组学研究. 2023."

508 "汪宁, et al., 预测庆大霉素、多柔比星和腺嘌呤致大鼠肾损伤的生物标志物. 中国药理学与毒理学杂志, 2015. 29(1): p. 84-91."

509 "王纯, hsa-miR-4669 在心脏手术相关性急性肾损伤中的早期预测价值. 2022."

510 "王佳欣, 尿外泌体miRNA--615--3p和miRNA--3147在糖尿病肾脏疾病中的表达及其与炎症纤维化的相关性研究. 2022."

511 "王晶, 基于智能手机读数垂直流动技术即时检测肾损伤标志物. 2023."

512 "王思微, 血清microRNA-217在特发性膜性肾病中表达与中医证型分布的研究. 2020."

513 "王万粮, 东洋, and 刘俊丽, 冠心病心力衰竭患者NT-proBNP和胱抑素C水平变化及阿托伐他汀对其的影响. 中西医结合心脑血管病杂志, 2012. 10(11): p. 1293-1294."

514 "王轩, 探索IGF-1、CysC对动脉瘤性蛛网膜下腔出血患者病情及预后的预测价值. 2022."

515 "王耀荣, 胱抑素-C与冠心病的相关性研究. 2014."

516 "温旺荣, 朱.A.陈.A.苏.A., 血清miR-21在糖尿病肾病中的诊断价值. 中国病理生理杂志, 2013. 29(12)."

517 "文璐, miRNAs的表达与原发性IgA肾病病情相关性初步研究. 2014."

518 "吴凡, Cys--C、miRNA--155及miRNA--21在无症状性脑梗死患者中的表达与诊断价值分析. 2019."

519 "谢媚媚, 潘敏, and 龚裕强, miR-29c在慢性肾脏病患者血清中的表达及临床意义研究. 浙江医学, 2019. 41(15): p. 1633-1635,1644."

520 "杨银忠, et al., PTH、IL-6和Cys-C联合检测对急、慢性肾功能衰竭鉴别诊断的评价. 基础医学与临床, 2016. 36(10): p. 1354-1358."

521 "张鸥, 阿托伐他汀对动脉粥样硬化患者外周血中PPARγ的作用研究及相关炎症因子与动脉粥样硬化关系的建模分析. 2016."

522 "张小飞, 血清外泌体miR-328和miR-671-5p有助于预测静脉注射免疫球蛋白治疗川崎病的疗效. 2020."

523 "张一平, 靳.A.靳.A., 联合检测血清miR-21和miR-25对2型糖尿病肾病的诊断价值. 华南国防医学杂志, 2020. 34(4)."

524 "张云峰, 血小板源mir-92a，胱抑素C与Ⅱ型糖尿病下肢缺血的相关性研究. 2016."

525 "赵海燕, 侯.A.李.A.席.A.郑.A., H-FABP、hs-cTnI、Hcy和Cys-C对急性心肌梗死早期诊断的临床评价. 国际检验医学杂志, 2015. 36(15)."

526 "赵旭敏, 贾.A., 血清miR-21、cysC、sCD40L及sICAM-1水平与糖尿病肾病的相关性分析. 医学临床研究, 2022. 39(8)."

527 "朱柄铭, et al., 血清miR-21在糖尿病肾病中的诊断价值. 中国病理生理杂志, 2013. 29(12): p. 2160-2166."

528 "朱炳铭, 血清miR-21在糖尿病肾病中的诊断价值. 2014."

Kidney-related but not acute kidney injury(136)

1 "Abe, M., et al., Efficacy analysis of the lipid-lowering and renoprotective effects of rosuvastatin in patients with chronic kidney disease. Endocr J, 2011. 58(8): p. 663-74."

2 "Akbari, A., et al., Canadian Society of Nephrology Commentary on the KDIGO Clinical Practice Guideline for CKD Evaluation and Management. American Journal of Kidney Diseases, 2015. 65(2): p. 177-205."

3 "Alam, M.L., et al., Soluble ST2 and Galectin-3 and Progression of CKD. Kidney International Reports, 2019. 4(1): p. 103-111."

4 "Bansal, N., et al., Bioelectrical Impedance Analysis Measures and Clinical Outcomes in CKD. American Journal of Kidney Diseases, 2018. 72(5): p. 662-672."

5 "Barreto, D.V., et al., Plasma interleukin-6 is independently associated with mortality in both hemodialysis and pre-dialysis patients with chronic kidney disease. Kidney International, 2010. 77(6): p. 550-556."

6 "Beetham, K.S., et al., Agreement between cystatin-C and creatinine based eGFR estimates after a 12-month exercise intervention in patients with chronic kidney disease. BMC Nephrol, 2018. 19(1): p. 366."

7 "Bellos, I., et al., Association of physical activity with endothelial dysfunction among adults with and without chronic kidney disease: The Maastricht Study. Atherosclerosis, 2023. 383: p. 117330."

8 "Bhavsar, N.A., et al., Comparison of measured GFR, serum creatinine, cystatin C, and beta-trace protein to predict ESRD in African Americans with hypertensive CKD. Am J Kidney Dis, 2011. 58(6): p. 886-93."

9 "Bj?rk, J., et al., Estimation of the glomerular filtration rate in children and young adults by means of the CKD-EPI equation with age-adjusted creatinine values. Kidney International, 2021. 99(4): p. 940-947."

10 "Bourgonje, A.R., et al., Serum free sulfhydryl status associates with new-onset chronic kidney disease in the general population. Redox Biology, 2021. 48: p. 102211."

11 "Bullen, A.L., et al., Biomarkers of Kidney Tubule Health, CKD Progression, and Acute Kidney Injury in SPRINT (Systolic Blood Pressure Intervention Trial) Participants. American Journal of Kidney Diseases, 2021. 78(3): p. 361-368.e1."

12 "Cai, Q., et al., Diet quality and incident chronic kidney disease in the general population: The Lifelines Cohort Study. Clinical Nutrition, 2021. 40(9): p. 5099-5105."

13 "Chen, H.W., et al., [Prevention and Treatment of Shenkang Injection for Contrast-induced Nephropathy in Elder Patients with Chronic Kidney Disease]. Zhongguo Zhong Xi Yi Jie He Za Zhi, 2016. 36(7): p. 792-796."

14 "Chen, Y., et al., [Clinical efficacy and safety of sequential treatment with alprostadil and beraprost sodium for chronic renal failure induced by chronic glomerulonephritis]. Nan Fang Yi Ke Da Xue Xue Bao, 2013. 33(10): p. 1521-4."

15 "Chen, Y.-J., et al., Microneedle patches integrated with lateral flow cassettes for blood-free chronic kidney disease point-of-care testing during a pandemic. Biosensors and Bioelectronics, 2022. 208: p. 114234."

16 "Cohen, J.B., et al., Time-Updated Changes in Estimated GFR and Proteinuria and Major Adverse Cardiac Events: Findings from the Chronic Renal Insufficiency Cohort (CRIC) Study. American Journal of Kidney Diseases, 2022. 79(1): p. 36-44.e1."

17 "Cooper, E.L., et al., Early Rapid Decline in Kidney Function in Medically Managed Patients With Atherosclerotic Renal Artery Stenosis. J Am Heart Assoc, 2019. 8(11): p. e012366."

18 "Correa, S., et al., Myeloperoxidase and the Risk of CKD Progression, Cardiovascular Disease, and Death in the Chronic Renal Insufficiency Cohort (CRIC) Study. American Journal of Kidney Diseases, 2020. 76(1): p. 32-41."

19 "Dai, Q., Y. Wang, and X.L. Qu, [Effects of Wenshen Huatan Recipe on the renal function and foe blood lipids of patients with early-middle stage chronic renal insufficiency of Pi-Shen yang deficiency]. Zhongguo Zhong Xi Yi Jie He Za Zhi, 2012. 32(2): p. 188-90."

20 "de Araújo, T.B., et al., The effects of home-based progressive resistance training in chronic kidney disease patients. Experimental Gerontology, 2023. 171: p. 112030."

21 "de Borst, M.H., et al., Effect of Omega-3 Fatty Acid Supplementation on Plasma Fibroblast Growth Factor 23 Levels in Post-Myocardial Infarction Patients with Chronic Kidney Disease: The Alpha Omega Trial. Nutrients, 2017. 9(11)."

22 "De Silva, P.M.C.S., et al., Occupational heat exposure alone does not explain chronic kidney disease of uncertain aetiology (CKDu) in Sri Lanka. The Journal of Climate Change and Health, 2022. 8: p. 100143."

23 "Delanaye, P., et al., Creatinine-or cystatin C-based equations to estimate glomerular filtration in the general population: impact on the epidemiology of chronic kidney disease. BMC Nephrol, 2013. 14: p. 57."

24 "Dong, F., et al., The clinical research on serum cystatin-C alteration on stage II chronic kidney disease with gubenquduyishen decoction treatment. J Ethnopharmacol, 2010. 131(3): p. 581-4."

25 "Edmonston, D., et al., Single Measurements of Carboxy-Terminal Fibroblast Growth Factor 23 and Clinical Risk Prediction of Adverse Outcomes in CKD. American Journal of Kidney Diseases, 2019. 74(6): p. 771-781."

26 "Fan, Z., et al., Efficacy of Oral Nicorandil to Prevent Contrast-Induced Nephropathy in Patients with Chronic Renal Dysfunction Undergoing an Elective Coronary Procedure. Kidney Blood Press Res, 2019. 44(6): p. 1372-1382."

27 "Fassett, R.G., et al., Effects of atorvastatin on NGAL and cystatin C in chronic kidney disease: a post hoc analysis of the LORD trial. Nephrol Dial Transplant, 2012. 27(1): p. 182-9."

28 "Feng, J.F., et al., Multicenter study of creatinine- and/or cystatin C-based equations for estimation of glomerular filtration rates in Chinese patients with chronic kidney disease. PLoS One, 2013. 8(3): p. e57240."

29 "Ferlizza, E., et al., The effect of chronic kidney disease on the urine proteome in the domestic cat (Felis catus). The Veterinary Journal, 2015. 204(1): p. 73-81."

30 "Foster, M.C., et al., Low Ankle-Brachial Index and the Development of Rapid Estimated GFR Decline and CKD. American Journal of Kidney Diseases, 2013. 61(2): p. 204-210."

31 "Foster, M.C., et al., Serum β-Trace Protein and β2-Microglobulin as Predictors of ESRD, Mortality, and Cardiovascular Disease in Adults With CKD in the Chronic Renal Insufficiency Cohort (CRIC) Study. American Journal of Kidney Diseases, 2016. 68(1): p. 68-76."

32 "Ghaemian, A., et al., Remote ischemic preconditioning to reduce contrast-induced acute kidney injury in chronic kidney disease: a randomized controlled trial. BMC Nephrol, 2018. 19(1): p. 373."

33 "Goldstein, S.L. and P. Devarajan, Progression From Acute Kidney Injury to Chronic Kidney Disease: A Pediatric Perspective. Advances in Chronic Kidney Disease, 2008. 15(3): p. 278-283."

34 "González-Pérez, A., et al., Impact of chronic kidney disease definition on assessment of its incidence and risk factors in patients with newly diagnosed type 1 and type 2 diabetes in the UK: A cohort study using primary care data from the United Kingdom. Primary Care Diabetes, 2020. 14(4): p. 381-387."

35 "Good, D.M., et al., Naturally Occurring Human Urinary Peptides for Use in Diagnosis of Chronic Kidney Disease*. Molecular & Cellular Proteomics, 2010. 9(11): p. 2424-2437."

36 "Goraya, N., et al., Urine citrate excretion identifies changes in acid retention as eGFR declines in patients with chronic kidney disease. Am J Physiol Renal Physiol, 2019. 317(2): p. F502-f511."

37 "Grams, M.E., et al., Risks of Adverse Events in Advanced CKD: The Chronic Renal Insufficiency Cohort (CRIC) Study. American Journal of Kidney Diseases, 2017. 70(3): p. 337-346."

38 "Greenwood, S.A., et al., Effect of exercise training on estimated GFR, vascular health, and cardiorespiratory fitness in patients with CKD: a pilot randomized controlled trial. Am J Kidney Dis, 2015. 65(3): p. 425-34."

39 "Hanatani, S., et al., Non-invasive testing for sarcopenia predicts future cardiovascular events in patients with chronic kidney disease. International Journal of Cardiology, 2018. 268: p. 216-221."

40 "Hein, A.M., et al., Estimated Glomerular Filtration Rate Variability in Patients With Heart Failure and Chronic Kidney Disease. Journal of Cardiac Failure, 2021. 27(11): p. 1175-1184."

41 "Hernandez, R., et al., The association of positive affect and cardiovascular health in Hispanics/Latinos with chronic kidney disease: Results from the Hispanic Community Health Study/Study of Latinos (HCHS/SOL). Preventive Medicine Reports, 2019. 15: p. 100916."

42 "Hezzell, M.J., et al., Measurements of echocardiographic indices and biomarkers of kidney injury in dogs with chronic kidney disease. The Veterinary Journal, 2020. 255: p. 105420."

43 "Hojs, R., et al., Serum cystatin C-based equation compared to serum creatinine-based equations for estimation of glomerular filtration rate in patients with chronic kidney disease. Clin Nephrol, 2008. 70(1): p. 10-7."

44 "Horackova, M., et al., Effect of rofecoxib on the glomerular filtration rate, proteinuria and the renin-angiotensin-aldosterone system in elderly subjects with chronic renal impairment. Int J Clin Pharmacol Ther, 2005. 43(9): p. 413-9."

45 "Hsu, C.Y., et al., Measured GFR does not outperform estimated GFR in predicting CKD-related complications. J Am Soc Nephrol, 2011. 22(10): p. 1931-7."

46 "Hundemer, G.L., et al., Performance of the 2021 Race-Free CKD-EPI Creatinine-?and Cystatin C–Based Estimated GFR Equations Among Kidney Transplant Recipients. American Journal of Kidney Diseases, 2022. 80(4): p. 462-472.e1."

47 "Hutchison, C.A., et al., Serum Polyclonal Immunoglobulin Free Light Chain Levels Predict Mortality in People With Chronic Kidney Disease. Mayo Clinic Proceedings, 2014. 89(5): p. 615-622."

48 "Inker, L.A., et al., KDOQI US Commentary on the 2012 KDIGO Clinical Practice Guideline for the Evaluation and Management of CKD. American Journal of Kidney Diseases, 2014. 63(5): p. 713-735."

49 "Ishigami, J., et al., Cardiac Structure and Function and Subsequent Kidney Disease Progression in Adults With CKD: The Chronic Renal Insufficiency Cohort (CRIC) Study. American Journal of Kidney Diseases, 2023. 82(2): p. 225-236."

50 "Jarocka, I.T., et al., Renal function after percutaneous coronary interventions depending on the type of hydration. Adv Med Sci, 2013. 58(2): p. 369-75."

51 "Jennaro, T.S., et al., Kidney function as a key driver of the pharmacokinetic response to high-dose L-carnitine in septic shock. Pharmacotherapy, 2023. 43(12): p. 1240-1250."

52 "Jeong, T.D., et al., Development and validation of the Korean version of CKD-EPI equation to estimate glomerular filtration rate. Clin Biochem, 2016. 49(9): p. 713-719."

53 "K?dziela, J., et al., Relationship between hemodynamic parameters of renal artery stenosis and the changes of kidney function after renal artery stenting in patients with hypertension and preserved renal function. Blood Press, 2015. 24(1): p. 30-4."

54 "Kammer, M., et al., Integrative analysis of prognostic biomarkers derived from multiomics panels helps discrimination of chronic kidney disease trajectories in people with type 2 diabetes. Kidney International, 2019. 96(6): p. 1381-1388."

55 "Kee, Y.K., et al., Comparison of Different Types of Oral Adsorbent Therapy in Patients with Chronic Kidney Disease: A Multicenter, Randomized, Phase IV Clinical Trial. Yonsei Med J, 2021. 62(1): p. 41-49."

56 "Khan, I.A., et al., N-Terminal Pro-B-Type Natriuretic Peptide and B-Type Natriuretic Peptide for Identifying Coronary Artery Disease and Left Ventricular Hypertrophy in Ambulatory Chronic Kidney Disease Patients. The American Journal of Cardiology, 2006. 97(10): p. 1530-1534."

57 "Ko, H.-Y., et al., Cystatin C and Neutrophil Gelatinase-Associated Lipocalin as Early Biomarkers for Chronic Kidney Disease in Dogs. Topics in Companion Animal Medicine, 2021. 45: p. 100580."

58 "Koning, S.H., et al., Alcohol consumption is inversely associated with the risk of developing chronic kidney disease. Kidney International, 2015. 87(5): p. 1009-1016."

59 "Koyama, A., et al., Orally active prostacyclin analogue beraprost sodium in patients with chronic kidney disease: a randomized, double-blind, placebo-controlled, phase II dose finding trial. BMC Nephrol, 2015. 16: p. 165."

60 "Kratz, M., et al., Relationship Between Chronic Kidney Disease, Glucose Homeostasis, and Plasma Osteocalcin Carboxylation and Fragmentation. Journal of Renal Nutrition, 2021. 31(3): p. 248-256."

61 "Lamb, E.J., et al., The eGFR-C study: accuracy of glomerular filtration rate (GFR) estimation using creatinine and cystatin C and albuminuria for monitoring disease progression in patients with stage 3 chronic kidney disease--prospective longitudinal study in a multiethnic population. BMC Nephrol, 2014. 15: p. 13."

62 "Le, D., et al., Plasma Biomarkers and Incident CKD Among Individuals Without Diabetes. Kidney Medicine, 2023. 5(11): p. 100719."

63 "Lemoine, S., et al., Cystatin C-Creatinine Based Glomerular Filtration Rate Equation in Obese Chronic Kidney Disease Patients: Impact of Deindexation and Gender. Am J Nephrol, 2016. 44(1): p. 63-70."

64 "Liabeuf, S., et al., High circulating levels of large splice variants of tenascin-C is associated with mortality and cardiovascular disease in chronic kidney disease patients. Atherosclerosis, 2011. 215(1): p. 116-124."

65 "Lim, W.H., et al., Comparison of estimated glomerular filtration rate by the chronic kidney disease epidemiology collaboration (CKD-EPI) equations with and without Cystatin C for predicting clinical outcomes in elderly women. PLoS One, 2014. 9(9): p. e106734."

66 "Liu, J., et al., Recombinant Brain Natriuretic Peptide for the Prevention of Contrast-Induced Nephropathy in Patients with Chronic Kidney Disease Undergoing Nonemergent Percutaneous Coronary Intervention or Coronary Angiography: A Randomized Controlled Trial. Biomed Res Int, 2016. 2016: p. 5985327."

67 "Lopes, P., et al., Disposable electrochemical immunosensor for analysis of cystatin C, a CKD biomarker. Talanta, 2019. 201: p. 211-216."

68 "Luo, Y., et al., Serum cystatin C is associated with peripheral artery stiffness in patients with type 2 diabetes mellitus combined with chronic kidney disease. Clinical Biochemistry, 2023. 118: p. 110593."

69 "Malhotra, R., et al., Effects of Intensive Blood Pressure Lowering on Kidney Tubule Injury in CKD: A Longitudinal Subgroup Analysis in SPRINT. Am J Kidney Dis, 2019. 73(1): p. 21-30."

70 "Mapuskar, K.A., et al., Avasopasem manganese (GC4419) protects against cisplatin-induced chronic kidney disease: An exploratory analysis of renal metrics from a randomized phase 2b clinical trial in head and neck cancer patients. Redox Biology, 2023. 60: p. 102599."

71 "Menon, S., et al., Acute Kidney Injury Associated with High Nephrotoxic Medication Exposure Leads to Chronic Kidney Disease after 6?Months. The Journal of Pediatrics, 2014. 165(3): p. 522-527.e2."

72 "Menon, V., et al., Cystatin C as a risk factor for outcomes in chronic kidney disease. Ann Intern Med, 2007. 147(1): p. 19-27."

73 "Miao, J., et al., Clinical utility of single molecule counting technology for quantification of KIM-1 in patients with heart failure and chronic kidney disease. Clinical Biochemistry, 2017. 50(16): p. 889-895."

74 "Mitsnefes, M.M., et al., Serum neutrophil gelatinase-associated lipocalin as a marker of renal function in children with chronic kidney disease. Pediatr Nephrol, 2007. 22(1): p. 101-8."

75 "Narala, K.R., et al., Management of Coronary Atherosclerosis and Acute Coronary Syndromes in Patients With Chronic Kidney Disease. Current Problems in Cardiology, 2013. 38(5): p. 165-206."

76 "Ng, D.K., et al., Time-varying coefficient of determination to quantify the explanatory power of biomarkers on longitudinal GFR among children with chronic kidney disease. Annals of Epidemiology, 2018. 28(8): p. 549-556."

77 "Obiols, J., et al., Validation of a new standardized cystatin C turbidimetric assay: evaluation of the three novel CKD-EPI equations in hypertensive patients. Clin Biochem, 2013. 46(15): p. 1542-7."

78 "Park, S., et al., A Mendelian randomization study found causal linkage between telomere attrition and chronic kidney disease. Kidney International, 2021. 100(5): p. 1063-1070."

79 "Pelander, L., et al., Urinary peptidome analyses for the diagnosis of chronic kidney disease in dogs. The Veterinary Journal, 2019. 249: p. 73-79."

80 "Peralta, C.A., et al., Electronic Decision Support for Management of CKD in Primary Care: A Pragmatic Randomized Trial. Am J Kidney Dis, 2020. 76(5): p. 636-644."

81 "Pierce, C.B., et al., Age- and sex-dependent clinical equations to estimate glomerular filtration rates in children and young adults with chronic kidney disease. Kidney International, 2021. 99(4): p. 948-956."

82 "Powe, N.R. and L.E. Boulware, Population-Based Screening for CKD. American Journal of Kidney Diseases, 2009. 53(3, Supplement 3): p. S64-S70."

83 "Rambod, M., et al., Association of vascular endothelial factors with cardiovascular outcome and mortality in chronic kidney disease patients: A 4-year cohort study. Atherosclerosis, 2014. 236(2): p. 360-365."

84 "Rebholz, C.M., et al., Plasma galectin-3 levels are associated with the risk of incident chronic kidney disease. Kidney International, 2018. 93(1): p. 252-259."

85 "Rehman, T., J. Fought, and R. Solomon, N-acetylcysteine effect on serum creatinine and cystatin C levels in CKD patients. Clin J Am Soc Nephrol, 2008. 3(6): p. 1610-4."

86 "Ristikankare, A., et al., Lack of renoprotective effect of i.v. N-acetylcysteine in patients with chronic renal failure undergoing cardiac surgery. Br J Anaesth, 2006. 97(5): p. 611-6."

87 "Sabanayagam, C., et al., Bidirectional Association of Retinal Vessel Diameters and Estimated GFR Decline: The Beaver Dam CKD Study. American Journal of Kidney Diseases, 2011. 57(5): p. 682-691."

88 "Sakuragi, S., et al., Serum cystatin C level is associated with left atrial enlargement, left ventricular hypertrophy and impaired left ventricular relaxation in patients with stage 2 or 3 chronic kidney disease. International Journal of Cardiology, 2015. 190: p. 287-292."

89 "Saland, J.M., et al., Impaired postprandial lipemic response in chronic kidney disease. Kidney International, 2016. 90(1): p. 172-180."

90 "Schnell, D., et al., Renal resistive index better predicts the occurrence of acute kidney injury than cystatin C. Shock, 2012. 38(6): p. 592-7."

91 "Schwartz, G.J., et al., Improved equations estimating GFR in children with chronic kidney disease using an immunonephelometric determination of cystatin C. Kidney International, 2012. 82(4): p. 445-453."

92 "Sezai, A., et al., Changeover Trial of Febuxostat and Topiroxostat for Hyperuricemia with Cardiovascular Disease: Sub-Analysis for Chronic Kidney Disease (TROFEO CKD Trial). Ann Thorac Cardiovasc Surg, 2020. 26(4): p. 202-208."

93 "Sezai, A., et al., Comparison of febuxostat and allopurinol for hyperuricemia in cardiac surgery patients with chronic kidney disease (NU-FLASH trial for CKD). J Cardiol, 2015. 66(4): p. 298-303."

94 "Shao, Z., X. Meng, and F. Meng, Efficacy and safety of mesenchymal stem cell in Chinese patients with chronic renal failure: A pilot study in Shandong province, China. Pak J Pharm Sci, 2021. 34(3(Special)): p. 1227-1231."

95 "Sharma, A., et al., Betulinic acid attenuates renal fibrosis in rat chronic kidney disease model. Biomedicine & Pharmacotherapy, 2017. 89: p. 796-804."

96 "Shchelochkov, O.A., et al., Chronic kidney disease in propionic acidemia. Genet Med, 2019. 21(12): p. 2830-2835."

97 "Smith, G., et al., Associations between frailty, physical performance, and renal biomarkers in older people with advanced chronic kidney disease. Eur Geriatr Med, 2021. 12(5): p. 943-952."

98 "Smith, K., et al., Fibroblast Growth Factor 23, High-Sensitivity Cardiac Troponin, and Left Ventricular Hypertrophy in CKD. American Journal of Kidney Diseases, 2013. 61(1): p. 67-73."

99 "Song, Y., et al., Carotid intraplaque neovascularization predicts atherosclerotic renal artery stenosis in patients with carotid artery stenosis. Nutrition, Metabolism and Cardiovascular Diseases, 2020. 30(9): p. 1492-1499."

100 "Spector, J.T., et al., Associations of blood lead with estimated glomerular filtration rate using MDRD, CKD-EPI and serum cystatin C-based equations. Nephrol Dial Transplant, 2011. 26(9): p. 2786-92."

101 "Stafford-Smith, M., et al., Acute Kidney Injury and Chronic Kidney Disease After Cardiac Surgery. Advances in Chronic Kidney Disease, 2008. 15(3): p. 257-277."

102 "Stefanowicz, J., et al., Chronic Kidney Disease in Wilms Tumour Survivors – What Do We Know Today?, in Wilms Tumor, M.M. van den Heuvel-Eibrink, Editor. 2016, Codon Publications"

103 "Succar, L., et al., Subclinical chronic kidney disease modifies the diagnosis of experimental acute kidney injury. Kidney International, 2017. 92(3): p. 680-692."

104 "Sun, T., et al., Cordyceps militaris Improves Chronic Kidney Disease by Affecting TLR4/NF-κB Redox Signaling Pathway. Oxid Med Cell Longev, 2019. 2019: p. 7850863."

105 "Tombach, B., et al., Renal tolerance of a neutral gadolinium chelate (gadobutrol) in patients with chronic renal failure: results of a randomized study. Radiology, 2001. 218(3): p. 651-7."

106 "Toth-Manikowski, S.M., et al., Sex Differences in Cardiovascular Outcomes in CKD: Findings From the CRIC Study. American Journal of Kidney Diseases, 2021. 78(2): p. 200-209.e1."

107 "Tuttle, K.R., et al., Effects of Stenting for Atherosclerotic Renal Artery Stenosis on eGFR and Predictors of Clinical Events in the CORAL Trial. Clin J Am Soc Nephrol, 2016. 11(7): p. 1180-1188."

108 "Vupputuri, S., et al., Differential Estimation of CKD Using Creatinine- Versus Cystatin C–Based Estimating Equations by Category of Body Mass Index. American Journal of Kidney Diseases, 2009. 53(6): p. 993-1001."

109 "Waikar, S.S., et al., Biological Variability of Estimated GFR and Albuminuria in CKD. American Journal of Kidney Diseases, 2018. 72(4): p. 538-546."

110 "Wang, D., et al., [Syndrome differentiation-based treatment with traditional Chinese medicine for proteinuria in patients with chronic kidney disease: a randomized multicenter trial]. Nan Fang Yi Ke Da Xue Xue Bao, 2013. 33(4): p. 502-6."

111 "Wang, J., et al., Serum Midkine, estimated glomerular filtration rate and chronic kidney disease-related events in elderly women: Perth Longitudinal Study of Aging Women. Sci Rep, 2020. 10(1): p. 14499."

112 "Warady, B.A., et al., Predictors of Rapid Progression of Glomerular and Nonglomerular Kidney Disease in Children and Adolescents: The?Chronic Kidney Disease in Children (CKiD) Cohort. American Journal of Kidney Diseases, 2015. 65(6): p. 878-888."

113 "White, C.A., et al., Comparison of the new and traditional CKD-EPI GFR estimation equations with urinary inulin clearance: A study of equation performance. Clinica Chimica Acta, 2019. 488: p. 189-195."

114 "Younis, N.N., et al., Inactivation of Wnt/β-catenin/renin angiotensin axis by tumor necrosis factor-alpha inhibitor, infliximab, ameliorates CKD induced in rats. Biochemical Pharmacology, 2021. 185: p. 114426."

115 "Yu, H., et al., Determinants of renal function in patients with renal artery stenosis. Vasc Med, 2011. 16(5): p. 331-8."

116 "Zhang, Q.-L., et al., Epidemiology of chronic kidney disease: Results from a population of older adults in Germany. Preventive Medicine, 2009. 48(2): p. 122-127."

117 "Zou, L.-X., et al., Comparison of bias and accuracy using cystatin C and creatinine in CKD-EPI equations for GFR estimation. European Journal of Internal Medicine, 2020. 80: p. 29-34."

118 "陈雪莹, 慢性肾脏病患者血清胱抑素C测定临床观察及意义. 医学检验与临床, 2015(2): p. 58-58,82."

119 "杜立树, 平龙玉, and 熊伟, 糖化血红蛋白与血清胱抑素C及尿微量清蛋白联合检测在慢性肾衰竭诊断中的应用. 检验医学与临床, 2015(6): p. 834-835."

120 "段晓星, Cystatin C评估慢性肾脏病肾功能的研究. 2009."

121 "葛启斌, et al., 血清NGAL、KIM-1、CysC在肾肿瘤患者中的表达及应用研究. 中国医师杂志, 2017. 19(12): p. 1836-1839."

122 "郭平凡 and 王家驷, 基于血清cystatin c、crea的CKD-EPI公式及相关标志物在慢性肾病中的临床应用评价. 中国卫生产业, 2014(24): p. 131-132."

123 "蒋一航, et al., NGAL与Cys c对于肾移植术后早期肾功能恢复的预测作用. 中华医学杂志, 2015. 95(2): p. 112-115."

124 "李瑞, 血清多种氨基酸联合诊断模型的建立用于慢性肾脏病代偿期的早期发现. 2017."

125 "刘红赛, 联合检测血浆CysC、血浆β2--MG、尿液NAG、尿液NGAL对慢性肾脏病肾小管间质损伤诊断价值的研究. 2020."

126 "庞国菊 and 刘怀平, 慢性肾脏病患者不同时期血清Cys C和GGT的水平. 中国老年学杂志, 2011. 31(22): p. 4462-4463."

127 "钱伟, MicroRNAs与肾移植术后移植物功能状态的相关性研究. 2018."

128 "粟宏伟, 血清胱抑素C评估肾移植患者肾小球滤过率的价值. 2005."

129 "王家驷, 郭.A., 基于血清cystatin c、crea的CKD-EPI公式及相关标志物在慢性肾病中的临床应用评价. 中国卫生产业, 2014. 11(24)."

130 "王启茹, et al., 慢性肾病各期中血清NGAL、CysC和肾功能指标的变化及其临床意义. 医学理论与实践, 2020. 33(20): p. 3349-3351,3355."

131 "王骞, 川黄方联合凯时对脾肾气虚、毒瘀互结型2-4期CKD合并AKI患者急性肾损伤标志物的影响. 2014."

132 "王世农, 血清胱抑素-C、血清肌酐与尿微量白蛋白联合检测在慢性肾病临床诊断中意义. 中国实验诊断学, 2013. 17(3): p. 545-546."

133 "余一海, 张.A.谢.A.王.A., 肾功能损害标志物测定在慢性肾脏病诊断中的临床价值. 解放军医学杂志, 2011. 36(12)."

134 "张成禄, et al., 肾功能损害标志物测定在慢性肾脏病诊断中的临床价值. 解放军医学杂志, 2011. 36(12): p. 1329-1331."

135 "张小东, 蒋.A.王.A.胡.A.尹.A.刘.A.任.A.王.A.范.A., NGAL与Cys c对于肾移植术后早期肾功能恢复的预测作用. 中华医学杂志, 2015. 95(2)."

136 "周翠翠, 吕风华, and 王现伟, 血清同型半胱氨酸和胱抑素C对慢性心力衰竭患者早期肾损伤的诊断价值. 新乡医学院学报, 2021. 38(11): p. 1025-1028."

"The diagnostic marker is not serum cystatin C, or is not a diagnostic test or cannot react alone or extract serum cystatin c(413)"

1 "Adel, F.W., et al., Annexin A1 is a Potential Novel Biomarker of Congestion in Acute Heart Failure. J Card Fail, 2020. 26(8): p. 727-732."

2 "Adeniran, A., et al., Detection of a Peptide Biomarker by Engineered Yeast Receptors. ACS Synthetic Biology, 2018. 7(2): p. 696-705."

3 "Adrees, M., et al., Effects of 18 months of L-T4 replacement in women with subclinical hypothyroidism. Clin Endocrinol (Oxf), 2009. 71(2): p. 298-303."

4 "Ahmad, T., et al., Worsening Renal Function in Patients With Acute Heart Failure Undergoing Aggressive Diuresis Is Not Associated With Tubular Injury. Circulation, 2018. 137(19): p. 2016-2028."

5 "Ahmadpour, S., et al., Alterations of cardiac and renal biomarkers in horses naturally infected with theileria equi. Comparative Immunology, Microbiology and Infectious Diseases, 2020. 71: p. 101502."

6 "Aita, A., et al., Salivary proteomic analysis in asymptomatic and symptomatic SARS-CoV-2 infection: Innate immunity, taste perception and FABP5 proteins make the difference. Clinica Chimica Acta, 2022. 537: p. 26-37."

7 "?kerblom, A., et al., Interleukin-18 in patients with acute coronary syndromes. Clin Cardiol, 2019. 42(12): p. 1202-1209."

8 "Albert, C., et al., Urinary biomarkers may provide prognostic information for subclinical acute kidney injury after cardiac surgery. The Journal of Thoracic and Cardiovascular Surgery, 2018. 155(6): p. 2441-2452.e13."

9 "Alshaikh, H.N., et al., Financial Impact of Acute Kidney Injury After Cardiac Operations in the United States. The Annals of Thoracic Surgery, 2018. 105(2): p. 469-475."

10 "Andersen, T., et al., C-X-C Ligand 16 Is an Independent Predictor of Cardiovascular Death and Morbidity in Acute Coronary Syndromes. Arterioscler Thromb Vasc Biol, 2019. 39(11): p. 2402-2410."

11 "Anderson, J.L., Lipoprotein-Associated Phospholipase A2: An Independent Predictor of Coronary Artery Disease Events in Primary and Secondary Prevention. The American Journal of Cardiology, 2008. 101(12, Supplement): p. S23-S33."

12 "Anderson, J.L.C., S.J.L. Bakker, and U.J.F. Tietge, The triglyceride to HDL-cholesterol ratio and chronic graft failure in renal transplantation. Journal of Clinical Lipidology, 2021. 15(2): p. 301-310."

13 "Anekthanakul, K., et al., Predicting lupus membranous nephritis using reduced picolinic acid to tryptophan ratio as a urinary biomarker. iScience, 2021. 24(11): p. 103355."

14 "Arsenault, B.J., et al., Prediction of cardiovascular events in statin-treated stable coronary patients of the treating to new targets randomized controlled trial by lipid and non-lipid biomarkers. PLoS One, 2014. 9(12): p. e114519."

15 "Arthur, J.M., et al., Evaluation of 32 urine biomarkers to predict the progression of acute kidney injury after cardiac surgery. Kidney International, 2014. 85(2): p. 431-438."

16 "Ascher, S.B., et al., Urine Biomarkers of Kidney Tubule Health and Incident CKD Stage 3 in Women Living With HIV: A Repeated Measures Study. Kidney Medicine, 2021. 3(3): p. 395-404.e1."

17 "Aygun, B., et al., Hydroxyurea treatment decreases glomerular hyperfiltration in children with sickle cell anemia. Am J Hematol, 2013. 88(2): p. 116-9."

18 "Azimi, A., et al., Differential proteomic analysis of actinic keratosis, Bowen’s disease and cutaneous squamous cell carcinoma by label-free LC–MS/MS. Journal of Dermatological Science, 2018. 91(1): p. 69-78."

19 "Badgujar, S.B., et al., A cost-effective method for purification and characterization of human urinary albumin. Journal of Chromatography B, 2019. 1114-1115: p. 31-44."

20 "Bandele, O., et al., Performance of urinary and gene expression biomarkers in detecting the nephrotoxic effects of melamine and cyanuric acid following diverse scenarios of co-exposure. Food and Chemical Toxicology, 2013. 51: p. 106-113."

21 "Basu, R.K., et al., Combining Functional and Tubular Damage Biomarkers Improves Diagnostic Precision for Acute Kidney Injury After Cardiac Surgery. Journal of the American College of Cardiology, 2014. 64(25): p. 2753-2762."

22 "Batte, A., et al., Neutrophil gelatinase-associated lipocalin is elevated in children with acute kidney injury and sickle cell anemia, and predicts mortality. Kidney International, 2022. 102(4): p. 885-893."

23 "Belabbas, D., et al., Effects of Remote Ischemic Pre-Conditioning to Prevent Contrast-Induced Nephropathy after Intravenous Contrast Medium Injection: A Randomized Controlled Trial. Korean J Radiol, 2020. 21(11): p. 1230-1238."

24 "Bergmark, B.A., et al., Klotho, fibroblast growth factor-23, and the renin-angiotensin system - an analysis from the PEACE trial. Eur J Heart Fail, 2019. 21(4): p. 462-470."

25 "Bhandari, S., et al., Multicentre randomized controlled trial of angiotensin-converting enzyme inhibitor/angiotensin receptor blocker withdrawal in advanced renal disease: the STOP-ACEi trial. Nephrol Dial Transplant, 2016. 31(2): p. 255-61."

26 "Bhensdadia, N.M., et al., Urine haptoglobin levels predict early renal functional decline in patients with type 2 diabetes. Kidney International, 2013. 83(6): p. 1136-1143."

27 "Biasucci, L.M., et al., Risk stratification of ischaemic patients with implantable cardioverter defibrillators by C-reactive protein and a multi-markers strategy: results of the CAMI-GUIDE study. Eur Heart J, 2012. 33(11): p. 1344-50."

28 "Bigot-Corbel, é. and S. Kamel, Nouveaux marqueurs biologiques de l’insuffisance rénale aigu?. Revue Francophone des Laboratoires, 2023. 2023(555): p. 32-40."

29 "Bjurman, C., et al., High-sensitive cardiac troponin, NT-proBNP, hFABP and copeptin levels in relation to glomerular filtration rates and a medical record of cardiovascular disease. Clinical Biochemistry, 2015. 48(4): p. 302-307."

30 "Bolignano, D., et al., Neutrophil Gelatinase–Associated Lipocalin (NGAL) as a Marker of Kidney Damage. American Journal of Kidney Diseases, 2008. 52(3): p. 595-605."

31 "Brankovic, M., et al., Plasma cystatin C and neutrophil gelatinase-associated lipocalin in relation to coronary atherosclerosis on intravascular ultrasound and cardiovascular outcome: Impact of kidney function (ATHEROREMO-IVUS study). Atherosclerosis, 2016. 254: p. 20-27."

32 "Brown, J.R., et al., Utility of Biomarkers to Improve Prediction of Readmission or Mortality After Cardiac Surgery. The Annals of Thoracic Surgery, 2018. 106(5): p. 1294-1301."

33 "Bublin, M., et al., Component-resolved diagnosis of kiwifruit allergy with purified natural and recombinant kiwifruit allergens. Journal of Allergy and Clinical Immunology, 2010. 125(3): p. 687-694.e1."

34 "Buemi, A., et al., Is plasma and urine neutrophil gelatinase-associated lipocalin (NGAL) determination in donors and recipients predictive of renal function after kidney transplantation? Clinical Biochemistry, 2014. 47(15): p. 68-72."

35 "Bullen, A.L., et al., The SPRINT trial suggests that markers of tubule cell function in the urine associate with risk of subsequent acute kidney injury while injury markers elevate after the injury. Kidney International, 2019. 96(2): p. 470-479."

36 "Cai, D., et al., Combination therapy with beraprost sodium and aspirin for acute ischemic stroke: a single-center retrospective study. J Int Med Res, 2019. 47(7): p. 3014-3024."

37 "Cai, L., et al., Assays of urine levels of HNL/NGAL in patients undergoing cardiac surgery and the impact of antibody configuration on their clinical performances. Clinica Chimica Acta, 2009. 403(1): p. 121-125."

38 "Carvalho, L.B., et al., Snap-heated freeze-free preservation and processing of the urine proteome using the combination of stabilizor-based technology and filter aided sample preparation. Analytica Chimica Acta, 2019. 1076: p. 82-90."

39 "Cavusoglu, E., et al., Relation of baseline plasma MMP-1 levels to long-term all-cause mortality in patients with known or suspected coronary artery disease referred for coronary angiography. Atherosclerosis, 2015. 239(1): p. 268-275."

40 "Chae, H., et al., Neutrophil Gelatinase-Associated Lipocalin as a Biomarker of Renal Impairment in Patients With Multiple Myeloma. Clinical Lymphoma Myeloma and Leukemia, 2015. 15(1): p. 35-40."

41 "Chaiben, C.L., et al., Salivary proteome analysis of crack cocaine dependents. Archives of Oral Biology, 2021. 121: p. 104952."

42 "Che, B., et al., Multiple biomarkers covering several pathways for the prediction of depression after ischemic stroke. Journal of Affective Disorders, 2021. 280: p. 442-449."

43 "Chen, X., et al., The predictive value of hematological inflammatory markers for acute kidney injury and mortality in adults with hemophagocytic Lymphohistiocytosis: A retrospective analysis of 585 patients. International Immunopharmacology, 2023. 122: p. 110564."

44 "Cheng, G., et al., Serum phospholipase A2 receptor antibodies and immunoglobulin G subtypes in adult idiopathic membranous nephropathy: Clinical value assessment. Clinica Chimica Acta, 2019. 490: p. 135-141."

45 "Chindarkar, N.S., et al., Reference intervals of urinary acute kidney injury (AKI) markers [IGFBP7]?[TIMP2] in apparently healthy subjects and chronic comorbid subjects without AKI. Clinica Chimica Acta, 2016. 452: p. 32-37."

46 "Cholongitas, E., et al., Association Between Ratio of Sodium to Potassium in Random Urine Samples and Renal Dysfunction and Mortality in Patients With Decompensated Cirrhosis. Clinical Gastroenterology and Hepatology, 2013. 11(7): p. 862-867."

47 "Ciregia, F., et al., Putative salivary biomarkers useful to differentiate patients with fibromyalgia. Journal of Proteomics, 2019. 190: p. 44-54."

48 "Collins, S., et al., Early Management of Patients With Acute Heart Failure: State of the Art and Future Directions. A Consensus Document From the Society for Academic Emergency Medicine/Heart Failure Society of America Acute Heart Failure Working Group. Journal of Cardiac Failure, 2015. 21(1): p. 27-43."

49 "Connolly, J.M., et al., Non-invasive and label-free detection of oral squamous cell carcinoma using saliva surface-enhanced Raman spectroscopy and multivariate analysis. Nanomedicine: Nanotechnology, Biology and Medicine, 2016. 12(6): p. 1593-1601."

50 "Cruz, D.N., et al., Role of Biomarkers in the Diagnosis and Management of Cardio-Renal Syndromes. Seminars in Nephrology, 2012. 32(1): p. 79-92."

51 "Cummings, J.J., et al., Intraoperative prediction of cardiac surgery–associated acute kidney injury using urinary biomarkers of cell cycle arrest. The Journal of Thoracic and Cardiovascular Surgery, 2019. 157(4): p. 1545-1553.e5."

52 "Da, Y., et al., Serial Quantification of Urinary Protein Biomarkers to Predict Drug-induced Acute Kidney Injury. Curr Drug Metab, 2019. 20(8): p. 656-664."

53 "Dardashti, A., et al., The predictive value of s-cystatin C for mortality after coronary artery bypass surgery. The Journal of Thoracic and Cardiovascular Surgery, 2016. 152(1): p. 139-146."

54 "Dash, P.K., et al., Biomarkers for the Diagnosis, Prognosis, and Evaluation of Treatment Efficacy for Traumatic Brain Injury. Neurotherapeutics, 2010. 7(1): p. 100-114."

55 "Davis, J., et al., Analytical validation and reference intervals for a commercial multiplex assay to measure five novel biomarkers for acute kidney injury in canine urine. Research in Veterinary Science, 2021. 139: p. 78-86."

56 "de Boer, I.H., et al., Effect of Vitamin D and Omega-3 Fatty Acid Supplementation on Kidney Function in Patients With Type 2 Diabetes: A Randomized Clinical Trial. Jama, 2019. 322(19): p. 1899-1909."

57 "de Melo Bezerra Cavalcante, C.T., et al., Syndecan-1 improves severe acute kidney injury prediction after pediatric cardiac surgery. The Journal of Thoracic and Cardiovascular Surgery, 2016. 152(1): p. 178-186.e2."

58 "de Winter, C.F., et al., A 3-year follow-up study on cardiovascular disease and mortality in older people with intellectual disabilities. Research in Developmental Disabilities, 2016. 53-54: p. 115-126."

59 "Delanaye, P., et al., Performance of the European Kidney Function Consortium (EKFC) creatinine-based equation in United States cohorts. Kidney International, 2024. 105(3): p. 629-637."

60 "Ding, L., et al., Data-driven clustering approach to identify novel phenotypes using multiple biomarkers in acute ischaemic stroke: A retrospective, multicentre cohort study. eClinicalMedicine, 2022. 53: p. 101639."

61 "Droppa, M., et al., Impact of N-acetylcysteine on contrast-induced nephropathy defined by cystatin C in patients with ST-elevation myocardial infarction undergoing primary angioplasty. Clin Res Cardiol, 2011. 100(11): p. 1037-43."

62 "Du, L., et al., inMTSCCA: An Integrated Multi-task Sparse Canonical Correlation Analysis for Multi-omic Brain Imaging Genetics. Genomics, Proteomics & Bioinformatics, 2023. 21(2): p. 396-413."

63 "Echeverría, L.E., et al., Profiles of cardiovascular biomarkers according to severity stages of Chagas cardiomyopathy. International Journal of Cardiology, 2017. 227: p. 577-582."

64 "Edelstein, C.L., Biomarkers of Acute Kidney Injury. Advances in Chronic Kidney Disease, 2008. 15(3): p. 222-234."

65 "Eggers, K.M., et al., High-sensitive cardiac troponin T and its relations to cardiovascular risk factors, morbidity, and mortality in elderly men. American Heart Journal, 2013. 166(3): p. 541-548.e1."

66 "ElAlfy, M.S., et al., Renal iron deposition by magnetic resonance imaging in pediatric β-thalassemia major patients: Relation to renal biomarkers, total body iron and chelation therapy. European Journal of Radiology, 2018. 103: p. 65-70."

67 "Elmas, A.T., et al., Analysis of urine biomarkers for early determination of acute kidney injury in non-septic and non-asphyxiated critically ill preterm neonates. J Matern Fetal Neonatal Med, 2017. 30(3): p. 302-308."

68 "ElSadek, A.E., et al., Kidney injury molecule-1/creatinine as a urinary biomarker of acute kidney injury in critically ill neonates. Journal of Pediatric Urology, 2020. 16(5): p. 688.e1-688.e9."

69 "Emans, M.E., et al., Neutrophil gelatinase-associated lipocalin (NGAL) in chronic cardiorenal failure is correlated with endogenous erythropoietin levels and decreases in response to low-dose erythropoietin treatment. Kidney Blood Press Res, 2012. 36(1): p. 344-54."

70 "Endre, Z.H., Biomarkers of acute kidney injury: time to learn from implementations. Critical Care and Resuscitation, 2021. 23(2): p. 137-140."

71 "Erturk, M., et al., Does intravenous or oral high-dose N-acetylcysteine in addition to saline prevent contrast-induced nephropathy assessed by cystatin C? Coron Artery Dis, 2014. 25(2): p. 111-7."

72 "Fang, X. and W.-W. Zhang, Affinity separation and enrichment methods in proteomic analysis. Journal of Proteomics, 2008. 71(3): p. 284-303."

73 "Ferguson, M.A., V.S. Vaidya, and J.V. Bonventre, Biomarkers of nephrotoxic acute kidney injury. Toxicology, 2008. 245(3): p. 182-193."

74 "Fernando, B.N.T.W., et al., Pilot Study of Renal Urinary Biomarkers for Diagnosis of CKD of Uncertain Etiology. Kidney International Reports, 2019. 4(10): p. 1401-1411."

75 "Formiga, F., et al., Clinical characteristics and one-year mortality according to admission renal function in patients with a first acute heart failure hospitalization. Revista Portuguesa de Cardiologia (English Edition), 2018. 37(2): p. 159-165."

76 "Forni, L.G., et al., Characterising acute kidney injury: The complementary roles of biomarkers of renal stress and renal function. Journal of Critical Care, 2022. 71: p. 154066."

77 "Foster, M.C., et al., Filtration Markers, Cardiovascular Disease, Mortality, and Kidney Outcomes in Stable Kidney Transplant Recipients: The FAVORIT Trial. Am J Transplant, 2017. 17(9): p. 2390-2399."

78 "Foster, M.C., et al., Novel Filtration Markers as Predictors of All-Cause and Cardiovascular Mortality in US Adults. American Journal of Kidney Diseases, 2013. 62(1): p. 42-51."

79 "Fox, E., et al., A pharmacologically-based approach to high dose methotrexate administration to investigate nephrotoxicity and acute kidney injury biomarkers in children and adolescents with newly diagnosed osteosarcoma. Cancer Chemother Pharmacol, 2021. 87(6): p. 807-815."

80 "Fraser, J.S., et al., Renal tubular injury is present in acute inflammatory bowel disease prior to the introduction of drug therapy. Aliment Pharmacol Ther, 2001. 15(8): p. 1131-7."

81 "Fuernau, G., et al., Prognostic impact of established and novel renal function biomarkers in myocardial infarction with cardiogenic shock: A biomarker substudy of the IABP-SHOCK II-trial. Int J Cardiol, 2015. 191: p. 159-66."

82 "Gabelle, A., et al., Plasma β-amyloid 40 levels are positively associated with mortality risks in the elderly. Alzheimer's & Dementia, 2015. 11(6): p. 672-680."

83 "Garcia, S., B. Ko, and S. Adabag, Contrast-Induced Nephropathy and Risk of Acute Kidney Injury and Mortality After Cardiac Operations. The Annals of Thoracic Surgery, 2012. 94(3): p. 772-776."

84 "García-Hernández, V., et al., A tandem mass tag (TMT) proteomic analysis during the early phase of experimental pancreatitis reveals new insights in the disease pathogenesis. Journal of Proteomics, 2018. 181: p. 190-200."

85 "Gautier, J.-C., et al., Evaluation of novel biomarkers of nephrotoxicity in Cynomolgus monkeys treated with gentamicin. Toxicology and Applied Pharmacology, 2016. 303: p. 1-10."

86 "Gerber, C., et al., Proximal tubule proteins are significantly elevated in bladder urine of patients with ureteropelvic junction obstruction and may represent novel biomarkers: A pilot study. Journal of Pediatric Urology, 2016. 12(2): p. 120.e1-120.e7."

87 "Gergei, I., et al., Association of soluble CD40L with short-term and long-term cardiovascular and all-cause mortality: The Ludwigshafen Risk and Cardiovascular Health (LURIC) study. Atherosclerosis, 2019. 291: p. 127-131."

88 "Gheith, I. and A. El-Mahmoudy, Novel and classical renal biomarkers as evidence for the nephroprotective effect of Carica papaya leaf extract. Bioscience Reports, 2018. 38(5)."

89 "Ghys, L.F.E., et al., The effect of feeding, storage and anticoagulant on feline serum cystatin C. The Veterinary Journal, 2015. 206(1): p. 91-96."

90 "Gil-Dones, F., et al., Inside human aortic stenosis: A proteomic analysis of plasma. Journal of Proteomics, 2012. 75(5): p. 1639-1653."

91 "Gipson, D.S., et al., Urinary Epidermal Growth Factor as a Marker of Disease Progression in Children With Nephrotic Syndrome. Kidney International Reports, 2020. 5(4): p. 414-425."

92 "González Rodríguez, J.D., et al., [Proteinuria in urinary infection and acute pyelonephritis in paediatric patients: can it replace scintigraphic studies in diagnostic localisation?]. Nefrologia, 2009. 29(2): p. 163-9."

93 "González, M.A., et al., Proteomic research on new urinary biomarkers of renal disease in canine leishmaniosis: Survival and monitoring response to treatment. Research in Veterinary Science, 2023. 161: p. 180-190."

94 "Gordin, E., et al., Urinary clusterin and cystatin B as biomarkers of tubular injury in dogs following envenomation by the European adder. Research in Veterinary Science, 2021. 134: p. 12-18."

95 "Goren, O. and I. Matot, Perioperative acute kidney injury. British Journal of Anaesthesia, 2015. 115: p. ii3-ii14."

96 "Grams, M.E., et al., Acute Kidney Injury After Major Surgery: A Retrospective Analysis of Veterans Health Administration Data. American Journal of Kidney Diseases, 2016. 67(6): p. 872-880."

97 "Grins, E., et al., Effect of Cyclosporine on Cytokine Production in Elective Coronary Artery Bypass Grafting: A Sub-Analysis of the CiPRICS (Cyclosporine to Protect Renal Function in Cardiac Surgery) Study. J Cardiothorac Vasc Anesth, 2022. 36(7): p. 1985-1994."

98 "Grodin, J.L., et al., Prognostic Implications of Changes in Amino-Terminal Pro–B-Type Natriuretic Peptide in Acute Decompensated Heart Failure: Insights From ASCEND-HF. Journal of Cardiac Failure, 2019. 25(9): p. 703-711."

99 "Gueret, G., et al., [Evaluation of the renal function in cardiac surgery with CPB: role of the cystatin C and the calculated creatinine clearance]. Ann Fr Anesth Reanim, 2007. 26(5): p. 412-7."

100 "Gulati, S., Acute kidney injury in children. Clinical Queries: Nephrology, 2012. 1(1): p. 103-108."

101 "Guo, X.-s., et al., Association of post-procedural early (within 24h) increases in serum creatinine with all-cause mortality after coronary angiography. Clinica Chimica Acta, 2017. 474: p. 96-101."

102 "Gutiérrez, A.M., et al., Proteomic analysis of porcine saliva. The Veterinary Journal, 2011. 187(3): p. 356-362."

103 "Gutiérrez-Corrales, A., et al., Relationship between salivary biomarkers and postoperative swelling after the extraction of impacted lower third molars. International Journal of Oral and Maxillofacial Surgery, 2017. 46(2): p. 243-249."

104 "Haase, M., et al., N-Acetylcysteine does not artifactually lower plasma creatinine concentration. Nephrol Dial Transplant, 2008. 23(5): p. 1581-7."

105 "Haase, M., et al., Phase II, randomized, controlled trial of high-dose N-acetylcysteine in high-risk cardiac surgery patients. Crit Care Med, 2007. 35(5): p. 1324-31."

106 "Haase-Fielitz, A., et al., Low preoperative hepcidin concentration as a risk factor for mortality after cardiac surgery: A pilot study. The Journal of Thoracic and Cardiovascular Surgery, 2013. 145(5): p. 1380-1386."

107 "Hagstr?m, E., et al., Growth Differentiation Factor 15 Predicts All-Cause Morbidity and Mortality in Stable Coronary Heart Disease. Clin Chem, 2017. 63(1): p. 325-333."

108 "Hagstr?m, E., et al., Growth differentiation factor-15 level predicts major bleeding and cardiovascular events in patients with acute coronary syndromes: results from the PLATO study. Eur Heart J, 2016. 37(16): p. 1325-33."

109 "Hall, P.S., et al., The future for diagnostic tests of acute kidney injury in critical care: evidence synthesis, care pathway analysis and research prioritisation. Health Technol Assess, 2018. 22(32): p. 1-274."

110 "Han, W.K., et al., Urinary biomarkers in the early diagnosis of acute kidney injury. Kidney International, 2008. 73(7): p. 863-869."

111 "Hanrieder, J., et al., Temporally resolved differential proteomic analysis of human ventricular CSF for monitoring traumatic brain injury biomarker candidates. Journal of Neuroscience Methods, 2009. 177(2): p. 469-478."

112 "Har, R.L., et al., The urinary cytokine/chemokine signature of renal hyperfiltration in adolescents with type 1 diabetes. PLoS One, 2014. 9(11): p. e111131."

113 "Harari, F., et al., Blood Lead Levels and Decreased Kidney Function in a Population-Based Cohort. American Journal of Kidney Diseases, 2018. 72(3): p. 381-389."

114 "Harjen, H.J., et al., Evaluation of Urinary Clusterin and Cystatin B as Biomarkers for Renal Injury in Dogs Envenomated by the European Adder (Vipera berus). Topics in Companion Animal Medicine, 2022. 46: p. 100586."

115 "Hartenbach, F.A.R.R., et al., Proteomic analysis of whole saliva in chronic periodontitis. Journal of Proteomics, 2020. 213: p. 103602."

116 "Haslene-Hox, H., et al., Increased WD-repeat containing protein 1 in interstitial fluid from ovarian carcinomas shown by comparative proteomic analysis of malignant and healthy gynecological tissue. Biochimica et Biophysica Acta (BBA) - Proteins and Proteomics, 2013. 1834(11): p. 2347-2359."

117 "Hata, A., et al., Distribution of urinary gamma-glutamyltransferase activity in 40- to 74-year-old Japanese women. Practical Laboratory Medicine, 2020. 20: p. e00161."

118 "Hauschke, M., et al., Neutrophil gelatinase-associated lipocalin production negatively correlates with HK-2 cell impairment: Evaluation of NGAL as a marker of toxicity in HK-2 cells. Toxicology in Vitro, 2017. 39: p. 52-57."

119 "Hazle, M.A., et al., Urinary biomarkers and renal near-infrared spectroscopy predict intensive care unit outcomes after cardiac surgery in infants younger than 6 months of age. The Journal of Thoracic and Cardiovascular Surgery, 2013. 146(4): p. 861-867.e1."

120 "Hellenthal, F.A.M.V.I., et al., Circulating Biomarkers and Abdominal Aortic Aneurysm Size. Journal of Surgical Research, 2012. 176(2): p. 672-678."

121 "Hernández, D., et al., Surrogate end points for graft failure and mortality in kidney transplantation. Transplantation Reviews, 2007. 21(2): p. 97-106."

122 "Hijazi, Z., et al., Efficacy and safety of dabigatran compared with warfarin in relation to baseline renal function in patients with atrial fibrillation: a RE-LY (Randomized Evaluation of Long-term Anticoagulation Therapy) trial analysis. Circulation, 2014. 129(9): p. 961-70."

123 "Hoffmann, D., et al., Evaluation of a urinary kidney biomarker panel in rat models of acute and subchronic nephrotoxicity. Toxicology, 2010. 277(1): p. 49-58."

124 "Hoffmann, U., et al., The value of N-acetylcysteine in the prevention of radiocontrast agent-induced nephropathy seems questionable. J Am Soc Nephrol, 2004. 15(2): p. 407-10."

125 "Holmquist, P. and P. Liuba, Urine α-Glutathione S-Transferase, systemic inflammation and arterial function in juvenile type 1 diabetes. Journal of Diabetes and its Complications, 2012. 26(3): p. 199-204."

126 "Hynninen, M.S., et al., N-acetylcysteine for the prevention of kidney injury in abdominal aortic surgery: a randomized, double-blind, placebo-controlled trial. Anesth Analg, 2006. 102(6): p. 1638-45."

127 "Ibrahim, N.E., et al., Worsening Renal Function during Management for Chronic Heart Failure with Reduced Ejection Fraction: Results From the Pro-BNP Outpatient Tailored Chronic Heart Failure Therapy (PROTECT) Study. Journal of Cardiac Failure, 2017. 23(2): p. 121-130."

128 "Iriuchishima, H., et al., Activin A: a novel urinary biomarker of renal impairment in multiple myeloma. Bioscience Reports, 2019. 39(5)."

129 "Ishigami, J., et al., 25-hydroxyvitamin D, Fibroblast Growth Factor 23, and Risk of Acute Kidney Injury Over 20 Years of Follow-Up. Kidney International Reports, 2021. 6(5): p. 1299-1308."

130 "Ismail, A.M., et al., Association of Normal and Mutated APOL1 G2 Rs60910145 alleles with SCD, Body Mass Index, and Renal Function Biomarkers and Indices. Current Research in Translational Medicine, 2024. 72(1): p. 103414."

131 "Ito, M., et al., Plasma neutrophil gelatinase-associated lipocalin predicts major adverse cardiovascular events after cardiac care unit discharge. Journal of Cardiology, 2016. 67(2): p. 184-191."

132 "Jablonski, H., et al., A single intraperitoneal injection of bovine fetuin-A attenuates bone resorption in a murine calvarial model of particle-induced osteolysis. Bone, 2017. 105: p. 262-268."

133 "Jacob, J.T. and B. Ham, Compositional Profiling and Biomarker Identification of the Tear Film. The Ocular Surface, 2008. 6(4): p. 175-185."

134 "Jafari, R., et al., Down-regulation of inflammatory signaling pathways despite up-regulation of Toll-like receptors; the effects of corticosteroid therapy in brain-dead kidney donors, a double-blind, randomized, controlled trial. Mol Immunol, 2018. 94: p. 36-44."

135 "Jaffery, Z., et al., A randomized trial of intravenous n-acetylcysteine to prevent contrast induced nephropathy in acute coronary syndromes. Catheter Cardiovasc Interv, 2012. 79(6): p. 921-6."

136 "Ji, S.S., et al., Urinary Creatinine Concentrations and Its Explanatory Variables in General Chinese Population: Implications for Creatinine Limits and Creatinine Adjustment. Biomedical and Environmental Sciences, 2022. 35(10): p. 899-910."

137 "Jonckheere, S., et al., A model-based analysis of the predictive performance of different renal function markers for cefepime clearance in the ICU. J Antimicrob Chemother, 2016. 71(9): p. 2538-46."

138 "Juraschek, S.P., et al., Effect of a high-protein diet on kidney function in healthy adults: results from the OmniHeart trial. Am J Kidney Dis, 2013. 61(4): p. 547-54."

139 "Juraschek, S.P., et al., Effect of glycemic index and carbohydrate intake on kidney function in healthy adults. BMC Nephrol, 2016. 17(1): p. 70."

140 "Juraschek, S.P., et al., Effects of Lowering Glycemic Index of Dietary Carbohydrate on Plasma Uric Acid Levels: The OmniCarb Randomized Clinical Trial. Arthritis Rheumatol, 2016. 68(5): p. 1281-9."

141 "Justice, J.N., et al., Evaluation of a blood-based geroscience biomarker index in a randomized trial of caloric restriction and exercise in older adults with heart failure with preserved ejection fraction. Geroscience, 2022. 44(2): p. 983-995."

142 "Kaffashian, S., et al., Association of plasma β-amyloid with MRI markers of structural brain aging the 3-City Dijon study. Neurobiology of Aging, 2015. 36(10): p. 2663-2670."

143 "Kammerer, T., et al., Comparison of 6% hydroxyethyl starch and 5% albumin for volume replacement therapy in patients undergoing cystectomy (CHART): study protocol for a randomized controlled trial. Trials, 2015. 16: p. 384."

144 "Kasepalu, T., et al., Remote Ischaemic Preconditioning Reduces Kidney Injury Biomarkers in Patients Undergoing Open Surgical Lower Limb Revascularisation: A Randomised Trial. Oxid Med Cell Longev, 2020. 2020: p. 7098505."

145 "Kestenbaum, B., et al., Vitamin D, Parathyroid Hormone, and Cardiovascular Events Among Older Adults. Journal of the American College of Cardiology, 2011. 58(14): p. 1433-1441."

146 "Khoshbin, E., et al., Is there a renoprotective value to leukodepletion during heart valve surgery? A randomized controlled trial (ROLO). J Cardiothorac Surg, 2021. 16(1): p. 58."

147 "Kifle, D.W., et al., Proteomic analysis of two populations of Schistosoma mansoni-derived extracellular vesicles: 15k pellet and 120k pellet vesicles. Molecular and Biochemical Parasitology, 2020. 236: p. 111264."

148 "Kistler, A.D., et al., Identification of a unique urinary biomarker profile in patients with autosomal dominant polycystic kidney disease. Kidney International, 2009. 76(1): p. 89-96."

149 "Kjaergaard, K.D., et al., Endogenous markers for estimation of renal function in peritoneal dialysis patients. Perit Dial Int, 2013. 33(2): p. 195-204."

150 "Kobayashi, T., et al., Association between high-sensitivity cardiac troponin T levels and incident stroke in the elderly Japanese population: Results from the Tohoku Medical Megabank Community-based Cohort Study. American Heart Journal Plus: Cardiology Research and Practice, 2022. 22: p. 100212."

151 "Koch, A., et al., Elevated asymmetric dimethylarginine levels predict short- and long-term mortality risk in critically ill patients. Journal of Critical Care, 2013. 28(6): p. 947-953."

152 "Kononikhin, A.S., et al., An untargeted approach for the analysis of the urine peptidome of women with preeclampsia. Journal of Proteomics, 2016. 149: p. 38-43."

153 "Kostic, D., et al., The role of renal biomarkers to predict the need of surgery in congenital urinary tract obstruction in infants. Journal of Pediatric Urology, 2019. 15(3): p. 242.e1-242.e9."

154 "Koyner, J.L., et al., Urine Biomarkers and Perioperative Acute Kidney Injury: The?Impact of Preoperative Estimated GFR. Am J Kidney Dis, 2015. 66(6): p. 1006-14."

155 "Kühn, A., et al., Kidney Function as Risk Factor and Predictor of Cardiovascular Outcomes and Mortality Among Older Adults. American Journal of Kidney Diseases, 2021. 77(3): p. 386-396.e1."

156 "Kule?, J., et al., Glomerular and tubular kidney damage markers in canine babesiosis caused by Babesia canis. Ticks and Tick-borne Diseases, 2018. 9(6): p. 1508-1517."

157 "Kwong, Y.T., et al., Imprecision of urinary iothalamate clearance as a gold-standard measure of GFR decreases the diagnostic accuracy of kidney function estimating equations. Am J Kidney Dis, 2010. 56(1): p. 39-49."

158 "La Barbera, G., et al., Saliva as a source of new phosphopeptide biomarkers: Development of a comprehensive analytical method based on shotgun peptidomics. Talanta, 2018. 183: p. 245-249."

159 "Lachenbruch, P.A., et al., Biomarkers and Surrogate Endpoints in Renal Transplantation: Present Status and Considerations for Clinical Trial Design. American Journal of Transplantation, 2004. 4(4): p. 451-457."

160 "Laisalmi-Kokki, M., et al., Potentially detrimental effects of N-acetylcysteine on renal function in knee arthroplasty. Free Radic Res, 2009. 43(7): p. 691-6."

161 "Latchoumycandane, C., L.E. Nagy, and T.M. McIntyre, Chronic ethanol ingestion induces oxidative kidney injury through taurine-inhibitable inflammation. Free Radical Biology and Medicine, 2014. 69: p. 403-416."

162 "Lee, J., et al., Soluble siglec-5 is a novel salivary biomarker for primary Sjogren's syndrome. Journal of Autoimmunity, 2019. 100: p. 114-119."

163 "Lee, N.M., et al., Impact of Isolyte Versus 0.9% Saline on Postoperative Event of Acute Kidney Injury Assayed by Urinary [TIMP-2]?×?[IGFBP7] in Patients Undergoing Cardiac Surgery. Journal of Cardiothoracic and Vascular Anesthesia, 2019. 33(2): p. 348-356."

164 "Lehmann, S., et al., Biomarkers of Alzheimer's disease: The present and the future. Revue Neurologique, 2013. 169(10): p. 719-723."

165 "Leiva, T., et al., Biomarkers of necrotizing enterocolitis in the era of machine learning and omics. Seminars in Perinatology, 2023. 47(1): p. 151693."

166 "Li, T., et al., Granzyme K - A novel marker to identify the presence and rupture of abdominal aortic aneurysm. International Journal of Cardiology, 2021. 338: p. 242-247."

167 "Li, T., et al., Serum Toll-like receptor 4: A novel and promising biomarker for identification of aortic aneurysmal diseases. Clinica Chimica Acta, 2018. 483: p. 69-75."

168 "Li, X., et al., Covalent organic framework-based immunosensor to detect plasma Latexin reveals novel biomarker for coronary artery diseases. Analytica Chimica Acta, 2023. 1284: p. 341993."

169 "Li, Z.T., L.Q. Ban, and F. Chen, [Acupuncture of revised acupoint combination around the skull base for post-stroke mild cognitive impairment: a randomized controlled trial]. Zhongguo Zhen Jiu, 2023. 43(10): p. 1104-8."

170 "Li, Z.Y., et al., [Effects of berberine on the serum cystatin C levels and urine albumin/creatine ratio in patients with type 2 diabetes mellitus]. Zhonghua Yi Xue Za Zhi, 2018. 98(46): p. 3756-3761."

171 "Lin, H.Y.-H., et al., Urinary neutrophil gelatinase-associated lipocalin levels predict cisplatin-induced acute kidney injury better than albuminuria or urinary cystatin C levels. The Kaohsiung Journal of Medical Sciences, 2013. 29(6): p. 304-311."

172 "Lin, P.-H., et al., Research performance of biomarkers from biofluids in periodontal disease publications. Journal of Dental Sciences, 2015. 10(1): p. 61-67."

173 "Lindholm, D., et al., Association of Multiple Biomarkers With Risk of All-Cause and Cause-Specific Mortality After Acute Coronary Syndromes: A Secondary Analysis of the PLATO Biomarker Study. JAMA Cardiol, 2018. 3(12): p. 1160-1166."

174 "Lindstr?m, V., et al., Different elimination patterns of beta-trace protein, beta2-microglobulin and cystatin C in haemodialysis, haemodiafiltration and haemofiltration. Scand J Clin Lab Invest, 2008. 68(8): p. 685-91."

175 "Liu, L., et al., Proteome analysis reveals novel serum biomarkers for Henoch-Sch?nlein purpura in Chinese children. Journal of Proteomics, 2023. 276: p. 104841."

176 "Liu, Y., et al., Serum anti-PLA2R antibody as a diagnostic biomarker of idiopathic membranous nephropathy: The optimal cut-off value for Chinese patients. Clinica Chimica Acta, 2018. 476: p. 9-14."

177 "Ljungberg, J., et al., Mild impairment of renal function (shrunken pore syndrome) is associated with increased risk for future surgery for aortic stenosis. Scand J Clin Lab Invest, 2019. 79(7): p. 524-530."

178 "Lock, E.A. and J.V. Bonventre, Biomarkers in translation; past, present and future. Toxicology, 2008. 245(3): p. 163-166."

179 "Lorenzo, M., et al., Early glomerular filtration rates changes and risk of mortality in acute heart failure. The modifying role of admission renal function and decongestion. European Journal of Internal Medicine, 2023. 115: p. 96-103."

180 "Lu, J., et al., Transcriptional Profiling of Keratinocytes Reveals a Vitamin D-Regulated Epidermal Differentiation Network. Journal of Investigative Dermatology, 2005. 124(4): p. 778-785."

181 "Ludwig, U., et al., MESNA (sodium 2-mercaptoethanesulfonate) for prevention of contrast medium-induced nephrotoxicity - controlled trial. Clin Nephrol, 2011. 75(4): p. 302-8."

182 "Luu, G.T., et al., An Integrated Approach to Protein Discovery and Detection From Complex Biofluids. Molecular & Cellular Proteomics, 2023. 22(7): p. 100590."

183 "Maglinger, B., et al., Influence of BMI on adenosine deaminase and stroke outcomes in mechanical thrombectomy subjects. Brain, Behavior, & Immunity - Health, 2022. 20: p. 100422."

184 "Malard, V., et al., Urine proteomic profiling of uranium nephrotoxicity. Biochimica et Biophysica Acta (BBA) - Proteins and Proteomics, 2009. 1794(6): p. 882-891."

185 "Mallick, A. and J.L. Januzzi, Biomarkers in Acute Heart Failure. Revista Espa?ola de Cardiología (English Edition), 2015. 68(6): p. 514-525."

186 "Manzano-Fernández, S., et al., Complementary Prognostic Value of Cystatin C, N-Terminal Pro-B-Type Natriuretic Peptide and Cardiac Troponin T in Patients With Acute Heart Failure. The American Journal of Cardiology, 2009. 103(12): p. 1753-1759."

187 "Martin-Moreno, P.L., et al., Comparison of Intravenous and Oral Hydration in the Prevention of Contrast-Induced Acute Kidney Injury in Low-Risk Patients: A Randomized Trial. Nephron, 2015. 131(1): p. 51-8."

188 "Marton Filho, M.A., et al., Effects of pneumoperitoneum on kidney injury biomarkers: A randomized clinical trial. PLoS One, 2021. 16(2): p. e0247088."

189 "Massoth, C., et al., Comparison of C-C motif chemokine ligand 14 with other biomarkers for adverse kidney events after cardiac surgery. The Journal of Thoracic and Cardiovascular Surgery, 2023. 165(1): p. 199-207.e2."

190 "Massy, Z.A., et al., Machine Learning-Based Urine Peptidome Analysis to Predict and Understand Mechanisms of Progression to Kidney Failure. Kidney International Reports, 2023. 8(3): p. 544-555."

191 "Matoso, A., et al., Expression microarray analysis identifies novel epithelial-derived protein markers in eosinophilic esophagitis. Modern Pathology, 2013. 26(5): p. 665-676."

192 "Mayer, T., et al., Urine Biomarkers of Tubular Renal Cell Damage for the Prediction of Acute Kidney Injury After Cardiac Surgery—A Pilot Study. Journal of Cardiothoracic and Vascular Anesthesia, 2017. 31(6): p. 2072-2079."

193 "McCullough, P.A. and J.L. Jefferies, Novel Markers and Therapies for Patients with Acute Heart Failure and Renal Dysfunction. The American Journal of Medicine, 2015. 128(3): p. 312.e1-312.e22."

194 "McDuffie, J.E., et al., Urinary parameters predictive of cisplatin-induced acute renal injury in dogs. Cytokine, 2010. 52(3): p. 156-162."

195 "McIlroy, D.R., et al., Combining Novel Renal Injury Markers with Delta Serum Creatinine Early after Cardiac Surgery and Risk-Stratification for Serious Adverse Outcomes: An Exploratory Analysis. Journal of Cardiothoracic and Vascular Anesthesia, 2018. 32(5): p. 2190-2200."

196 "McMahon, G.M. and S.S. Waikar, Biomarkers in Nephrology: Core Curriculum 2013. American Journal of Kidney Diseases, 2013. 62(1): p. 165-178."

197 "Mehta, T.K., et al., Quantitative Detection of Promoter Hypermethylation as a Biomarker of Acute Kidney Injury During Transplantation. Transplantation Proceedings, 2006. 38(10): p. 3420-3426."

198 "Meinitzer, A., et al., Symmetrical and asymmetrical dimethylarginine as predictors for mortality in patients referred for coronary angiography: the Ludwigshafen Risk and Cardiovascular Health study. Clin Chem, 2011. 57(1): p. 112-21."

199 "Metzger, J., et al., Urinary excretion of twenty peptides forms an early and accurate diagnostic pattern of acute kidney injury. Kidney International, 2010. 78(12): p. 1252-1262."

200 "Miklaszewska, M., et al., Reference ranges and impact of selected confounders on classic serum and urinary renal markers in neonatal period. Advances in Medical Sciences, 2017. 62(1): p. 143-150."

201 "Miller, L.M., et al., Association of Urine Biomarkers of Kidney Tubule Injury and Dysfunction With Frailty Index and Cognitive Function in Persons With CKD in SPRINT. American Journal of Kidney Diseases, 2021. 78(4): p. 530-540.e1."

202 "Miller, L.M., et al., Cardiovascular damage phenotypes and all-cause and CVD mortality in older adults. Annals of Epidemiology, 2021. 63: p. 35-40."

203 "Mishra, J., et al., Neutrophil gelatinase-associated lipocalin (NGAL) as a biomarker for acute renal injury after cardiac surgery. The Lancet, 2005. 365(9466): p. 1231-1238."

204 "Miura, Y., et al., Absolute quantification of cholesteryl esters using liquid chromatography-tandem mass spectrometry uncovers novel diagnostic potential of urinary sediment. Steroids, 2017. 123: p. 43-49."

205 "Mohamed, F., et al., Kidney damage biomarkers detect acute kidney injury but only functional markers predict mortality after paraquat ingestion. Toxicology Letters, 2015. 237(2): p. 140-150."

206 "Mohamed, F., et al., Mechanism-specific injury biomarkers predict nephrotoxicity early following glyphosate surfactant herbicide (GPSH) poisoning. Toxicology Letters, 2016. 258: p. 1-10."

207 "Moist, L., et al., Effect of N-acetylcysteine on serum creatinine and kidney function: results of a randomized controlled trial. Am J Kidney Dis, 2010. 56(4): p. 643-50."

208 "Molina, H., et al., A Proteomic Analysis of Human Hemodialysis Fluid*S. Molecular & Cellular Proteomics, 2005. 4(5): p. 637-650."

209 "Neely, B.A., et al., Proteomic Analysis of Urine from California Sea Lions (Zalophus californianus): A Resource for Urinary Biomarker Discovery. Journal of Proteome Research, 2018. 17(9): p. 3281-3291."

210 "Nejat, M., et al., Some biomarkers of acute kidney injury are increased in pre-renal acute injury. Kidney International, 2012. 81(12): p. 1254-1262."

211 "Neves, M.M.P.S., et al., Neutrophil gelatinase-associated lipocalin detection using a sensitive electrochemical immunosensing approach. Sensors and Actuators B: Chemical, 2020. 304: p. 127285."

212 "Neyra, J.A., et al., Kidney Tubular Damage and Functional Biomarkers in Acute Kidney Injury Following Cardiac Surgery. Kidney International Reports, 2019. 4(8): p. 1131-1142."

213 "Nguyen, L.S., et al., Evaluation of neutrophil gelatinase-associated lipocalin and cystatin C as biomarkers of acute kidney injury after ST-segment elevation myocardial infarction treated by percutaneous coronary intervention. Archives of Cardiovascular Diseases, 2019. 112(3): p. 180-186."

214 "Nivy, R., et al., Utility of urinary alkaline phosphatase and γ-glutamyl transpeptidase in diagnosing acute kidney injury in dogs. The Veterinary Journal, 2017. 220: p. 43-47."

215 "Nú?ez, J., et al., Cardiorenal Syndrome in Acute Heart Failure: Revisiting Paradigms. Revista Espa?ola de Cardiología (English Edition), 2015. 68(5): p. 426-435."

216 "Olsson, E., et al., Vitamin D is not associated with incident dementia or cognitive impairment: an 18-y follow-up study in community-living old men1, 2, 3. The American Journal of Clinical Nutrition, 2017. 105(4): p. 936-943."

217 "O'Sullivan, J.A. and B.S. Bochner, Eosinophils and eosinophil-associated diseases: An update. Journal of Allergy and Clinical Immunology, 2018. 141(2): p. 505-517."

218 "Pache de Faria Guimaraes, L., et al., N-acetyl-cysteine is associated to renal function improvement in patients with nephropathic cystinosis. Pediatr Nephrol, 2014. 29(6): p. 1097-102."

219 "Palazzuoli, A., et al., Comparison of Neutrophil Gelatinase-Associated Lipocalin Versus B-Type Natriuretic Peptide and Cystatin C to Predict Early Acute Kidney Injury and Outcome in Patients With Acute Heart Failure. Am J Cardiol, 2015. 116(1): p. 104-11."

220 "Palli, E., et al., The impact of N-acetylcysteine and ascorbic acid in contrast-induced nephropathy in critical care patients: an open-label randomized controlled study. Crit Care, 2017. 21(1): p. 269."

221 "Pavkovic, M., et al., Comparison of the Mesoscale Discovery and Luminex multiplex platforms for measurement of urinary biomarkers in a cisplatin rat kidney injury model. Journal of Pharmacological and Toxicological Methods, 2014. 69(2): p. 196-204."

222 "Peralta, C.A., et al., Associations of Urinary Levels of Kidney Injury Molecule 1 (KIM-1) and Neutrophil Gelatinase-Associated Lipocalin (NGAL) With Kidney Function Decline in the Multi-Ethnic Study of Atherosclerosis (MESA). American Journal of Kidney Diseases, 2012. 60(6): p. 904-911."

223 "Perazella, M.A., The Urine Sediment as a Biomarker of Kidney Disease. American Journal of Kidney Diseases, 2015. 66(5): p. 748-755."

224 "Pérez-Calvo, J.I., et al., Prognostic value of serum cystatin C and N-terminal pro b-type natriuretic peptide in patients with acute heart failure. European Journal of Internal Medicine, 2012. 23(7): p. 599-603."

225 "Perinpam, M., et al., Effect of Demographics on Excretion of Key Urinary Factors Related to Kidney Stone Risk. Urology, 2015. 86(4): p. 690-696."

226 "Pickering, J.W., A.M. Ralib, and Z.H. Endre, Combining creatinine and volume kinetics identifies missed cases of acute kidney injury following cardiac arrest. Crit Care, 2013. 17(1): p. R7."

227 "Pirgakis, K.M., et al., Urinary Cystatin C as an Early Biomarker of Acute Kidney Injury after Open and Endovascular Abdominal Aortic Aneurysm Repair. Annals of Vascular Surgery, 2014. 28(7): p. 1649-1658."

228 "Poletti, P.A., et al., I.v. N-acetylcysteine and emergency CT: use of serum creatinine and cystatin C as markers of radiocontrast nephrotoxicity. AJR Am J Roentgenol, 2007. 189(3): p. 687-92."

229 "Poletti, P.A., et al., N-acetylcysteine does not prevent contrast nephropathy in patients with renal impairment undergoing emergency CT: a randomized study. BMC Nephrol, 2013. 14: p. 119."

230 "Politis, M.D., et al., Recent ambient temperature and fine particulate matter (PM2.5) exposure is associated with urinary kidney injury biomarkers in children. Science of The Total Environment, 2024. 907: p. 168119."

231 "Post, A., et al., Urinary 3-hydroxyisovaleryl carnitine excretion, protein energy malnutrition and risk of all-cause mortality in kidney transplant recipients: Results from the TransplantLines cohort studies. Clinical Nutrition, 2021. 40(4): p. 2109-2120."

232 "P?e?ek, J., et al., Prognostic value of cystatin C in relation to other markers of renal function in early prediction of hospital mortality and major cardiac adverse events in patients with ST elevation myocardial infarction treated by primary percutaneous coronary intervention. Cor et Vasa, 2018. 60(4): p. e352-e360."

233 "Pronschinske, K.B., et al., Neutrophil gelatinase-associated lipocalin and cystatin C for the prediction of clinical events in patients with advanced heart failure and after ventricular assist device placement. The Journal of Heart and Lung Transplantation, 2014. 33(12): p. 1215-1222."

234 "Rakkolainen, I. and J. Vuola, Plasma NGAL predicts early acute kidney injury no earlier than s-creatinine or cystatin C in severely burned patients. Burns, 2016. 42(2): p. 322-328."

235 "Ralib, A.M., et al., The clinical utility window for acute kidney injury biomarkers in the critically ill. Crit Care, 2014. 18(6): p. 601."

236 "Ramesh, G., O. Kwon, and K. Ahn, Netrin-1: A Novel Universal Biomarker of Human Kidney Injury. Transplantation Proceedings, 2010. 42(5): p. 1519-1522."

237 "Rao, V.S., et al., Association of Urine Galectin-3 With Cardiorenal Outcomes in Patients With Heart Failure. Journal of Cardiac Failure, 2024. 30(2): p. 340-346."

238 "Raposeiras-Roubín, S., et al., Relation of Soluble Receptor for Advanced Glycation End Products to Predict Mortality in Patients With Chronic Heart Failure Independently of Seattle Heart Failure Score. The American Journal of Cardiology, 2011. 107(6): p. 938-944."

239 "Rapson, I.R., et al., Serum 25-hydroxyvitamin D is associated with incident peripheral artery disease among white and black adults in the ARIC study cohort. Atherosclerosis, 2017. 257: p. 123-129."

240 "Redouane, B., et al., Effects of Liraglutide on Worsening Renal Function Among Patients With Heart Failure With Reduced Ejection Fraction: Insights From the FIGHT Trial. Circ Heart Fail, 2020. 13(5): p. e006758."

241 "Rhode, H., et al., Urinary Protein-Biomarkers Reliably Indicate Very Early Kidney Damage in Children With Alport Syndrome Independently of Albuminuria and Inflammation. Kidney International Reports, 2023. 8(12): p. 2778-2793."

242 "Riad, A., et al., MD-2 is a new predictive biomarker in dilated cardiomyopathy and exerts direct effects in isolated cardiomyocytes. International Journal of Cardiology, 2018. 270: p. 278-286."

243 "Ricci, Z., et al., High-dose fenoldopam reduces postoperative neutrophil gelatinase-associated lipocaline and cystatin C levels in pediatric cardiac surgery. Crit Care, 2011. 15(3): p. R160."

244 "Ristikankare, A., et al., Effects of levosimendan on renal function in patients undergoing coronary artery surgery. J Cardiothorac Vasc Anesth, 2012. 26(4): p. 591-5."

245 "Ristiniemi, N., et al., Cystatin C as a predictor of all-cause mortality and myocardial infarction in patients with non-ST-elevation acute coronary syndrome. Clinical Biochemistry, 2012. 45(7): p. 535-540."

246 "Rizk, D.V., et al., A Novel Method for Rapid Bedside Measurement of GFR. J Am Soc Nephrol, 2018. 29(6): p. 1609-1613."

247 "Roscigno, G., et al., Urinary Dickkopf-3 and Contrast-Associated Kidney Damage. Journal of the American College of Cardiology, 2021. 77(21): p. 2667-2676."

248 "Roy, R., et al., Acute serum protein and cytokine response of single dose of prednisone in adult volunteers. Steroids, 2022. 178: p. 108953."

249 "Ruiz, P., et al., Urinary cystatin C and N-acetyl-beta-D-glucosaminidase (NAG) as early biomarkers for renal disease in dogs with leishmaniosis. Veterinary Parasitology, 2023. 318: p. 109930."

250 "Rumpel, J., et al., Urine Biomarkers for the Assessment of Acute Kidney Injury in Neonates with Hypoxic Ischemic Encephalopathy Receiving Therapeutic Hypothermia. The Journal of Pediatrics, 2022. 241: p. 133-140.e3."

251 "Salinger-Martinovic, S., et al., Renal dysfunction as intrahospital prognostic indicator in acute pulmonary embolism. International Journal of Cardiology, 2020. 302: p. 143-149."

252 "Salvador, C.L., et al., Renal function, sex and age influence purines and pyrimidines in urine and could lead to diagnostic misinterpretation. Molecular Genetics and Metabolism, 2023. 140(3): p. 107649."

253 "Saraf, A., et al., Biomarker profile in stable Fontan patients. International Journal of Cardiology, 2020. 305: p. 56-62."

254 "Sasaki, A., et al., Comparison of Renal Biomarkers with Glomerular Filtration Rate in Susceptibility to the Detection of Gentamicin-Induced Acute Kidney Injury in Dogs. Journal of Comparative Pathology, 2014. 151(2): p. 264-270."

255 "Scheppach, J.B., et al., Albuminuria and Estimated GFR as Risk Factors for Dementia in Midlife and Older Age: Findings From the ARIC Study. American Journal of Kidney Diseases, 2020. 76(6): p. 775-783."

256 "Scheurlen, K.M., et al., Serum uromodulin and Roux-en-Y gastric bypass: improvement of a marker reflecting nephron mass. Surgery for Obesity and Related Diseases, 2019. 15(8): p. 1319-1325."

257 "Schilcher, G., et al., Early detection and intervention using neutrophil gelatinase-associated lipocalin (NGAL) may improve renal outcome of acute contrast media induced nephropathy: a randomized controlled trial in patients undergoing intra-arterial angiography (ANTI-CIN Study). BMC Nephrol, 2011. 12: p. 39."

258 "Schmidt, I.M., et al., Plasma Kidney Injury Molecule 1 in CKD: Findings From the Boston Kidney Biopsy Cohort and CRIC Studies. American Journal of Kidney Diseases, 2022. 79(2): p. 231-243.e1."

259 "Serrano-Mendioroz, I., et al., Vitamin D-binding protein as a biomarker of active disease in acute intermittent porphyria. Journal of Proteomics, 2015. 127: p. 377-385."

260 "Shafi, T., et al., Estimating residual kidney function in dialysis patients without urine collection. Kidney International, 2016. 89(5): p. 1099-1110."

261 "Sharma, P., et al., A comprehensive proteomic profiling of urinary exosomes and the identification of early non-invasive biomarker in patients with coronary artery disease. Journal of Proteomics, 2024. 293: p. 105059."

262 "Sharma, R.K., Biomarkers of acute kidney injury. Clinical Queries: Nephrology, 2012. 1(1): p. 13-17."

263 "Shaver, L.N., et al., Effect of Intentional Weight Loss on Mortality Biomarkers in Older Adults With Obesity. J Gerontol A Biol Sci Med Sci, 2019. 74(8): p. 1303-1309."

264 "Shen, Y., et al., High-sensitivity C-reactive protein and cystatin C independently and jointly predict all-cause mortality among the middle-aged and elderly Chinese population. Clinical Biochemistry, 2019. 65: p. 7-14."

265 "Sherief, L.M., et al., Screening of renal dysfunction among Burkitt lymphoma survivors by novel markers. Hematology, 2017. 22(5): p. 265-273."

266 "Shlipak, M.G., et al., Cystatin-C and inflammatory markers in the ambulatory elderly. Am J Med, 2005. 118(12): p. 1416."

267 "Siew, E.D., et al., Distinct injury markers for the early detection and prognosis of incident acute kidney injury in critically ill adults with preserved kidney function. Kidney International, 2013. 84(4): p. 786-794."

268 "Silvetti, S., et al., Preoperative Urinary Neutrophil Gelatinase-Associated Lipocalin and Outcome in High-Risk Heart Failure Patients Undergoing Cardiac Surgery. Journal of Cardiothoracic and Vascular Anesthesia, 2014. 28(2): p. 323-327."

269 "Singer, E., et al., Urinary neutrophil gelatinase-associated lipocalin distinguishes pre-renal from intrinsic renal failure and predicts outcomes. Kidney International, 2011. 80(4): p. 405-414."

270 "Sionis, A., et al., Update on Ischemic Heart Disease and Intensive Cardiac Care. Revista Espa?ola de Cardiología (English Edition), 2015. 68(3): p. 234-241."

271 "Sohrabian, A., et al., Particle enhanced turbidimetric immunoassay for the determination of urine cystatin C on Cobas c501. Clinical Biochemistry, 2012. 45(4): p. 339-344."

272 "Solbu, M.D., et al., Kidney function and markers of renal damage after renal denervation. Does method of measurement matter? The Reshape CV-Risk Study. J Clin Hypertens (Greenwich), 2021. 23(5): p. 954-962."

273 "Soler-García, á.A., et al., A urinary biomarker profile for children with HIV-associated renal diseases. Kidney International, 2009. 76(2): p. 207-214."

274 "Song, J.W., et al., Double-blinded, randomized controlled trial of N-acetylcysteine for prevention of acute kidney injury in high risk patients undergoing off-pump coronary artery bypass. Nephrology (Carlton), 2015. 20(2): p. 96-102."

275 "Soria, J., et al., Tear proteome and protein network analyses reveal a novel pentamarker panel for tear film characterization in dry eye and meibomian gland dysfunction. Journal of Proteomics, 2013. 78: p. 94-112."

276 "Srisawat, N., et al., Urinary biomarkers and renal recovery in critically ill patients with renal support. Clin J Am Soc Nephrol, 2011. 6(8): p. 1815-23."

277 "Starodubtseva, N.L., et al., Investigation of urine proteome of preterm newborns with respiratory pathologies. Journal of Proteomics, 2016. 149: p. 31-37."

278 "Stypmann, J., et al., Neutrophil gelatinase-associated lipocalin (NGAL) in heart transplant recipients after conversion to everolimus therapy. Journal of Cardiology, 2015. 66(4): p. 347-352."

279 "Suárez-Fernández, A., et al., Determination of Cystatin C in human urine by isotope dilution tandem mass spectrometry. Journal of Pharmaceutical and Biomedical Analysis, 2020. 177: p. 112889."

280 "Sumaya, W., et al., Fibrin clot properties independently predict adverse clinical outcome following acute coronary syndrome: a PLATO substudy. Eur Heart J, 2018. 39(13): p. 1078-1085."

281 "Sun, B., et al., Urinary biomarker evaluation for early detection of gentamycin-induced acute kidney injury. Toxicology Letters, 2019. 300: p. 73-80."

282 "Sun, P.-p., et al., Urine macrophages reflect kidney macrophage content during acute tubular interstitial and glomerular injury. Clinical Immunology, 2019. 205: p. 65-74."

283 "Sundaram, N., et al., Biomarkers for early detection of sickle nephropathy. Am J Hematol, 2011. 86(7): p. 559-66."

284 "Svennberg, E., et al., NT-proBNP is a powerful predictor for incident atrial fibrillation — Validation of a multimarker approach. International Journal of Cardiology, 2016. 223: p. 74-81."

285 "Szummer, K., et al., Relationship of plasma erythropoietin to long-term outcome in acute coronary syndrome. International Journal of Cardiology, 2010. 143(2): p. 165-170."

286 "Takahashi, G., et al., Diagnostic accuracy of procalcitonin and presepsin for infectious disease in patients with acute kidney injury. Diagnostic Microbiology and Infectious Disease, 2016. 86(2): p. 205-210."

287 "Talalak, K., et al., A facile low-cost enzymatic paper-based assay for the determination of urine creatinine. Talanta, 2015. 144: p. 915-921."

288 "Tang, W.H.W., et al., Usefulness of Plasma Galectin-3 Levels in Systolic Heart Failure to Predict Renal Insufficiency and Survival. The American Journal of Cardiology, 2011. 108(3): p. 385-390."

289 "Tolomeo, P., et al., Importance of cystatin C in estimating glomerular filtration rate: the PARADIGM-HF trial. Eur Heart J, 2023. 44(24): p. 2202-2212."

290 "Tomaschitz, A., et al., Aldosterone/Renin Ratio Determines Peripheral and Central Blood Pressure Values Over a Broad Range. Journal of the American College of Cardiology, 2010. 55(19): p. 2171-2180."

291 "Tomaschitz, A., et al., Association of Plasma Aldosterone With Cardiovascular Mortality in Patients With Low Estimated GFR: The Ludwigshafen Risk and Cardiovascular Health (LURIC) Study. American Journal of Kidney Diseases, 2011. 57(3): p. 403-414."

292 "Tomaschitz, A., et al., Independent association between 1,25-dihydroxyvitamin D, 25-hydroxyvitamin D and the renin–angiotensin system: The Ludwigshafen Risk and Cardiovascular Health (LURIC) study. Clinica Chimica Acta, 2010. 411(17): p. 1354-1360."

293 "Tomonaga, Y., et al., Insights on urinary NGAL obtained in a primary care setting. Clinica Chimica Acta, 2012. 413(7): p. 733-739."

294 "Tonkin, A.M., et al., Biomarkers in stable coronary heart disease, their modulation and cardiovascular risk: The LIPID biomarker study. Int J Cardiol, 2015. 201: p. 499-507."

295 "Tonomura, Y., et al., Evaluation of the usefulness of urinary biomarkers for nephrotoxicity in rats. Toxicology, 2010. 273(1): p. 53-59."

296 "Torigoe, K., et al., 20-Hour preprocedural hydration is not superior to 5-hour preprocedural hydration in the prevention of contrast-induced increases in serum creatinine and cystatin C. Int J Cardiol, 2013. 167(5): p. 2200-3."

297 "Torralba-Cabeza, M.-á., et al., Cystatin C and NT-proBNP as prognostic biomarkers in Fabry disease. Molecular Genetics and Metabolism, 2011. 104(3): p. 301-307."

298 "Tuttolomondo, A., et al., Fetuin-A and CD40 L plasma levels in acute ischemic stroke: Differences in relation to TOAST subtype and correlation with clinical and laboratory variables. Atherosclerosis, 2010. 208(1): p. 290-296."

299 "Tziakas, D., et al., Spot urine albumin to creatinine ratio outperforms novel acute kidney injury biomarkers in patients with acute myocardial infarction. International Journal of Cardiology, 2015. 197: p. 48-55."

300 "Ueland, T., et al., ALCAM predicts future cardiovascular death in acute coronary syndromes: Insights from the PLATO trial. Atherosclerosis, 2020. 293: p. 35-41."

301 "V??r?niemi, K., et al., Lower glomerular filtration rate is associated with higher systemic vascular resistance in patients without prevalent kidney disease. J Clin Hypertens (Greenwich), 2014. 16(10): p. 722-8."

302 "Valente, M.A.E., et al., Urinary Proteins in Heart Failure. Progress in Cardiovascular Diseases, 2012. 55(1): p. 44-55."

303 "Vashist, S.K., Graphene-based immunoassay for human lipocalin-2. Analytical Biochemistry, 2014. 446: p. 96-101."

304 "Vejux, A., et al., Oxysterols and multiple sclerosis: Physiopathology, evolutive biomarkers and therapeutic strategy. The Journal of Steroid Biochemistry and Molecular Biology, 2021. 210: p. 105870."

305 "Velazquez, E.J., et al., Rationale and design of the comParIson Of sacubitril/valsartaN versus Enalapril on Effect on nt-pRo-bnp in patients stabilized from an acute Heart Failure episode (PIONEER-HF) trial. American Heart Journal, 2018. 198: p. 145-151."

306 "Verbrugge, F.H., et al., Novel Urinary Biomarkers in Detecting Acute Kidney Injury, Persistent Renal Impairment, and All-Cause Mortality Following Decongestive Therapy in Acute Decompensated Heart Failure. Journal of Cardiac Failure, 2013. 19(9): p. 621-628."

307 "Verbrugge, F.H., et al., Prognostic Value of Glomerular Filtration Changes Versus Natriuretic Response in Decompensated Heart Failure With Reduced Ejection. Journal of Cardiac Failure, 2014. 20(11): p. 817-824."

308 "Vijayan, A., et al., Clinical Use of the Urine Biomarker [TIMP-2]?× [IGFBP7] for?Acute Kidney Injury Risk Assessment. American Journal of Kidney Diseases, 2016. 68(1): p. 19-28."

309 "von Jeinsen, B., et al., Urinary neutrophil gelatinase-associated lipocalin and cystatin C compared to the estimated glomerular filtration rate to predict risk in patients with suspected acute myocardial infarction. International Journal of Cardiology, 2017. 245: p. 6-12."

310 "Wagener, G., et al., Urinary Neutrophil Gelatinase-Associated Lipocalin and Acute Kidney Injury After Cardiac Surgery. American Journal of Kidney Diseases, 2008. 52(3): p. 425-433."

311 "Walker, S.J. and A. Xu, Biomarker Discovery using Molecular Profiling Approaches, in International Review of Neurobiology. 2004, Academic Press. p. 1-30."

312 "Wang, B., et al., Arg-liposome-amplified colorimetric immunoassay for selective and sensitive detection of cystatin C to predict acute kidney injury. Analytica Chimica Acta, 2022. 1236: p. 340562."

313 "Wang, H., et al., Development of a novel immunoassay for the simple and fast quantitation of neutrophil gelatinase-associated lipocalin using europium(III) chelate microparticles and magnetic beads. Journal of Immunological Methods, 2019. 470: p. 15-19."

314 "Wang, J., et al., Lipid microsphere-coated PGE1 improves peritoneal transport and reduces inflammation in peritoneal dialysis: A randomized clinical pilot trial. Semin Dial, 2021. 34(3): p. 235-244."

315 "Wang, J., et al., Paper-based multiplex colorimetric vertical flow assay with smartphone readout for point-of-care detection of acute kidney injury biomarkers. Sensors and Actuators B: Chemical, 2023. 390: p. 134029."

316 "Wang, Q., et al., iTRAQ technology-based identification of human peripheral serum proteins associated with depression. Neuroscience, 2016. 330: p. 291-325."

317 "Wang, W.-J., et al., A simple electrochemical immunosensor based on a gold nanoparticle monolayer electrode for neutrophil gelatinase-associated lipocalin detection. Talanta, 2022. 246: p. 123530."

318 "Wang, Y.S., et al., Prediction of the severity of acute kidney injury after on-pump cardiac surgery. Journal of Clinical Anesthesia, 2022. 78: p. 110664."

319 "Weaver, V.M., et al., Differences in urine cadmium associations with kidney outcomes based on serum creatinine and cystatin C. Environmental Research, 2011. 111(8): p. 1236-1242."

320 "Weidemann, F., et al., Early detection of organ involvement in Fabry disease by biomarker assessment in conjunction with LGE cardiac MRI: results from the SOPHIA study. Molecular Genetics and Metabolism, 2019. 126(2): p. 169-182."

321 "Welberry Smith, M.P., et al., Serum aminoacylase-1 is a novel biomarker with potential prognostic utility for long-term outcome in patients with delayed graft function following renal transplantation. Kidney International, 2013. 84(6): p. 1214-1225."

322 "Wettersten, N., et al., Urinary Biomarkers and Kidney Outcomes: Impact of Indexing Versus Adjusting for Urinary Creatinine. Kidney Medicine, 2021. 3(4): p. 546-554.e1."

323 "White, M., et al., Cardiac signaling molecules and plasma biomarkers after cardiac transplantation: Impact of tacrolimus versus cyclosporine. The Journal of Heart and Lung Transplantation, 2013. 32(12): p. 1222-1232."

324 "Widera, C., et al., Diagnostic and prognostic value of sex- and age-specific cutpoints for high-sensitivity Troponin T in non-ST-elevation acute coronary syndrome. International Journal of Cardiology, 2019. 275: p. 13-19."

325 "Wijkstr?m, J., et al., Clinical and Pathological Characterization of Mesoamerican Nephropathy: A New Kidney Disease in Central America. American Journal of Kidney Diseases, 2013. 62(5): p. 908-918."

326 "Woitas, R.P., et al., Cystatin C is independently associated with total and cardiovascular mortality in individuals undergoing coronary angiography. The Ludwigshafen Risk and Cardiovascular Health (LURIC) study. Atherosclerosis, 2013. 229(2): p. 541-548."

327 "Wong, J., et al., Predicting residual kidney function in hemodialysis patients using serum β-trace protein and β2-microglobulin. Kidney International, 2016. 89(5): p. 1090-1098."

328 "Wu, C.-K., et al., Renal-related biomarkers and long-term mortality in the US subjects with different coronary risks. Atherosclerosis, 2011. 216(1): p. 226-236."

329 "Wu, H.-B., et al., Can Renal Resistive Index Predict Acute Kidney Injury After Acute Type A Aortic Dissection Repair? The Annals of Thoracic Surgery, 2017. 104(5): p. 1583-1589."

330 "Wu, L., et al., GDF-15 and sST-2 act as biomarkers of disease severity but not independent predictors in idiopathic membranous nephropathy. International Immunopharmacology, 2022. 111: p. 109150."

331 "Xing, K., et al., Effect of rhBNP on renal function in STEMI-HF patients with mild renal insufficiency undergoing primary PCI. Heart Vessels, 2016. 31(4): p. 490-8."

332 "Yagmur, E., et al., Hyaluronan serum concentrations are elevated in critically ill patients and associated with disease severity. Clinical Biochemistry, 2012. 45(1): p. 82-87."

333 "Yamaguchi, Y., et al., The Plasma Proteome Fingerprint Associated with Circulating Carotenoids and Retinol in Older Adults. The Journal of Nutrition, 2022. 152(1): p. 40-48."

334 "Yamamoto, T., et al., Cystatin C as a predictor of mortality and cardiovascular morbidity after cardiac resynchronization therapy. Circ J, 2013. 77(11): p. 2751-6."

335 "Yokota, H., et al., Absence of Increased α1-Microglobulin in IgA Nephropathy Proteinuria*. Molecular & Cellular Proteomics, 2007. 6(4): p. 738-744."

336 "Zhang, L., et al., Serum creatinine/cystatin C ratio is a predictor of all-cause mortality for older adults over 80 years. Heliyon, 2023. 9(3): p. e14214."

337 "Zhou, H., et al., Exosomal Fetuin-A identified by proteomics: A novel urinary biomarker for detecting acute kidney injury. Kidney International, 2006. 70(10): p. 1847-1857."

338 "曹玉涵, 史.A.吴.A.彭.A.肖.A.徐.A.窦.A., 尿液外泌体来源hsa_circ_0008925与IgA肾病肾脏纤维化 的相关性研究. 皖南医学院学报, 2022. 41(6)."

339 "曹玉涵, 汪.A., 尿液中哺乳动物雷帕霉素靶蛋白mRNA表达量与IgA肾病患者肾脏纤维化的相关性研究. 皖南医学院学报, 2020. 39(3)."

340 "车妙琳, et al., 联合应用标志物在心脏手术后急性肾损伤的早期诊断. 中华肾脏病杂志, 2011. 27(3): p. 164-169."

341 "陈灿锋, et al., 生物标志物联合检测早期在心脏手术后急性肾损伤诊断中的应用. 国际检验医学杂志, 2017. 38(24): p. 3467-3468."

342 "陈纯波, 池.A.邓.A.袁.A.王.A.吕.A.龙.A.孙.A., 尿NAG联合血清CysC预测重症患者急性肾损伤诊断和预后的临床价值. 中华急诊医学杂志, 2016. 25(2)."

343 "陈海平, 王.A., 造影剂肾病早期诊断的生物标志物. 肾脏病与透析肾移植杂志, 2011. 20(3)."

344 "陈顺煌, 射血分数保留与射血分数降低心衰的生物学标记物的对比研究. 2017."

345 "陈召金, 黄敏辉, and 姚慧梅, 肾小管损伤标志物在儿科疾病联合诊断中的临床分析. 检验医学与临床, 2013(22): p. 2990-2991."

346 "陈志强, 评价联合生物学标志物在心脏手术相关急性肾损伤的早期诊断价值. 2016."

347 "池锐彬, 尿NAG联合血清CysC预测重症患者急性肾损伤诊断和预后的临床价值. 2016."

348 "范春玲 and 刘永哲, 白介素-6联合中性粒细胞明胶酶相关载脂蛋白对紫绀型先天性心脏病患儿体外循环术后急性肾损伤的预测价值. 中国心血管病研究, 2023. 21(8): p. 697-703."

349 "冯晓辉, 急性肾损伤生物标志物在冠脉造影及PCI术患者尿液中的变化. 2011."

350 "龚好, 中性粒细胞明胶酶相关脂质运载蛋白在急性失代偿性心力衰竭后急性肾损伤中的应用价值. 2015."

351 "韩慧敏, 膜性肾病患者特异性血清标志物的表达及临床特征分析. 2023."

352 "何卓雄, 司.A., 尿液足细胞标志蛋白Podocalyxin在诊断肾脏疾病早期损害中的价值. 中国医药指南, 2011. 9(20)."

353 "候大勇, 刘.A., 肾小管标记物对原发性肾病综合征诊断价值. 昆明医科大学学报, 2016. 37(6)."

354 "黄文彩, 彭.A.邓.A.张.A.付.A.陈.A.陈.A.刘.A.章.A., NGAL和KIM-1在重症监护室危重患者早期急性肾损伤中的诊断价值. 检验医学与临床, 2013. 10(18)."

355 "黄文彦, 刘.A.沈.A.孙.A.匡.A.张.A.张.A.周.A.李.A., 补体应答基因-32在儿童急性肾损伤中的早期预测价值. 中华儿科杂志, 2014. 52(7)."

356 "蒋蕾, 尿肾素对危重症患儿急性肾损伤的早期预测价值及与预后的相关性研究. 2020."

357 "康福新, et al., NGAL变异值在成人危重病患者急性肾损伤中的早期诊断价值. 海南医学, 2018. 29(24): p. 3423-3426."

358 "康福新, et al., 中性粒细胞明胶酶相关脂质运载蛋白变化值对成人ICU患者发生急性肾损伤的诊断价值. 实用医学杂志, 2019. 35(5): p. 760-763."

359 "李晋 and 周永年, 半胱氨酸蛋白酶抑制剂C在肾脏疾病诊断中的应用. 山西医药杂志（下半月版）, 2009. 38(20): p. 942-943."

360 "李梅, 雷湘菊, and 伍晓铭, 胱抑素检测在心脏疾病诊断及预后评估中的作用. 临床医学工程, 2018. 25(7): p. 903-904."

361 "李琦, 李.A.王.A.杨.A., 冠状动脉旁路移植术后急性肾损伤的危险因素分析. 标记免疫分析与临床, 2015. 22(10)."

362 "李轶春, et al., 尿液胱抑素C检测在肾损伤中的诊断价值. 现代医学与健康研究（电子版）, 2022. 6(23): p. 52-55."

363 "李宇球, 多种生物标志物在HFmrEF、HFpEF和HFrEF患者中的诊断意义. 2017."

364 "李源, et al., 冠状动脉旁路移植术后急性肾损伤的危险因素分析. 标记免疫分析与临床, 2015. 22(10): p. 978-981."

365 "李紫莹, et al., 尿细胞周期停滞标志物对老年患者腔镜腹部大手术后急性肾损伤的预测价值. 国际麻醉学与复苏杂志, 2023. 44(1): p. 62-67."

366 "联合功能和小管损伤标志物提高心脏术后急性肾损伤诊断. 内科, 2014. 9(6)."

367 "梁国玲, 李.A.黄.A.刘.A.彭.A.岑.A., 尿液胱抑素C检测在肾损伤中的诊断价值. 现代医学与健康研究电子杂志, 2022. 6(23)."

368 "梁有卓, 陈.A.王.A.杨.A.彭.A.林.A., 生物标志物联合检测早期在心脏手术后急性肾损伤诊断中的应用. 国际检验医学杂志, 2017. 38(24)."

369 "梁雨凡, 最低血小板计数对失血性休克患者发生急性肾损伤的预测价值. 2023."

370 "刘春, 能量代谢调节在PCI围手术期与衰老进程中的肾脏保护作用. 2019."

371 "刘华杰, et al., 补体应答基因-32在儿童急性肾损伤中的早期预测价值. 中华儿科杂志, 2014. 52(7): p. 494-499."

372 "刘俊丽, 王.A.东.A., 冠心病心力衰竭患者NT-proBNP和胱抑素C水平变化及阿托伐他汀对其的影响. 中西医结合心脑血管病杂志, 2012. 10(11)."

373 "刘琳, 动脉粥样硬化性烟雾综合征肾脏损伤标志物的研究. 2018."

374 "刘宁, 急性ST段抬高型心肌梗死患者住院时间影响因素研究. 2022."

375 "刘荣欣, 心脏X综合征患者血清Adropin、PTx-3、GGT水平变化研究. 2014."

376 "刘瑜 and 候大勇, 肾小管标记物对原发性肾病综合征诊断价值. 昆明医科大学学报, 2016. 37(6): p. 105-108."

377 "刘志红, 汤.A.郑.A.尹.A.张.A., 肾小管损伤标志物在局灶节段性肾小球硬化患者中的变化及意义. 肾脏病与透析肾移植杂志, 2010(4)."

378 "麻贤辉 and 王鑫, DPP4、PCT和CysC在急性冠脉综合征早期诊断中的价值研究. 全科医学临床与教育, 2021. 19(12): p. 1064-1067."

379 "马雪丽, 肾氧饱和度、肾阻力指数、sCysC和eGFR-CysC在心脏手术相关急性肾损伤中的早期诊断价值比较. 2020."

380 "彭兰芬, et al., NGAL 和 KIM-1在重症监护室危重患者早期急性肾损伤中的诊断价值. 检验医学与临床, 2013(18): p. 2356-2358."

381 沈雄文. 尿胱抑素C检测方法的建立及初步临床应用. 2006.

382 "史媛慧, et al., 尿液外泌体来源hsa_circ_0008925与IgA肾病肾脏纤维化的相关性研究. 皖南医学院学报, 2022. 41(6): p. 528-531."

383 "宋文琪, 田.A.刘.A.任.A.蔡.A.张.A., 尿液肾损伤标志物联合检测对儿童紫癜性肾炎的诊断价值. 中华检验医学杂志, 2022. 45(7)."

384 "孙华东, 血清Klotho蛋白对心脏瓣膜置换术后急性肾损伤监测的研究. 2013."

385 "田晓怡, et al., 尿液肾损伤标志物联合检测对儿童紫癜性肾炎的诊断价值. 中华检验医学杂志, 2022. 45(7): p. 732-737."

386 "汪裕伟 and 曹玉涵, 尿液中哺乳动物雷帕霉素靶蛋白mRNA表达量与IgA肾病患者肾脏纤维化的相关性研究. 皖南医学院学报, 2020. 39(3): p. 217-221."

387 "王会芹, 探讨TIMP-2及IGFBP7在心脏术后AKI的早期诊断价值. 2016."

388 "王瑞, 血清Angiopoietin--like Protein2水平与冠心病的相关性研究. 2018."

389 "王三凤, 尿nephrin与重症新生儿急性肾损伤及预后的相关性研究. 2018."

390 "王伟伟, et al., 血清心型脂肪酸结合蛋白在急性肾损伤诊断中的临床意义. 临床肾脏病杂志, 2018. 18(4): p. 229-233."

391 "王亚平, 彭春艳, and 朱名安, 冠心病合并代谢综合征患者固醇调节元件结合蛋白表达水平与脂代谢指标相关性研究. 陕西医学杂志, 2020. 49(4): p. 419-423,438."

392 "王荧荧, 预测儿童原发性肾病综合征激素疗效的尿液标志物研究. 2012."

393 "吴春涛, NGAL、 Cys C在瓣膜病换瓣术后早期诊断急性肾损伤的意义. 河北医科大学学报, 2019. 40(12): p. 1388-1392."

394 "伍利利, 唐恩燕, and 石亮, 中性粒细胞与淋巴细胞比值预测急性胰腺炎患者发生急性肾损伤的价值分析. 现代实用医学, 2018. 30(9): p. 1149-1150,1239,封2."

395 "严玉澄, 车.A.钱.A.戴.A.吴.A.倪.A.薛.A., 联合应用标志物在心脏手术后急性肾损伤的早期诊断. 中华肾脏病杂志, 2011. 27(3)."

396 "杨洁, 唐丽红, and 陈丽, 5项生化指标在肾功能损伤诊断和预后评估中的价值. 医药前沿, 2018. 8(14): p. 198-199."

397 "姚慧梅, 陈.A.黄.A., 肾小管损伤标志物在儿科疾病联合诊断中的临床分析. 检验医学与临床, 2013. 10(22)."

398 "于臻, cTnI对老年非心源性心肌损伤患者预后影响的临床研究. 2019."

399 "袁欣欣, 基于ITRAQ技术的“IGT肾病”尿液蛋白标志物研究. 2018."

400 "张国富 and 杨彦民, 诊断早期肾损伤的标志物. 中国社区医师（医学专业半月刊）, 2009. 11(14): p. 165."

401 "张翩, et al., 尿液中性粒细胞明胶酶相关载脂蛋白应用于早期诊断对比剂肾病的价值. 上海医学, 2013. 36(3): p. 194-199."

402 "张婷, 汪盛平, and 曹文斋, 血浆氧化三甲胺水平与非瓣膜性心房颤动的相关性研究. 中华老年心脑血管病杂志, 2023. 25(4): p. 381-385."

403 "张艺欣, 宁莉, and 魏殿军, Semaphorin 3A早期诊断对比剂急性肾损伤的价值. 检验医学, 2018. 33(11): p. 997-1003."

404 "张勇, 彭.A.陈.A.许.A.彭.A.庄.A.王.A.俞.A.李.A., ANCA相关性小血管炎肾损害患者尿液肾损伤生物标志物的检测及意义. 中国中西医结合肾病杂志, 2017. 18(10)."

405 "张志宏, 新型生物分子Galectin-3和FGF-23与慢性肾脏病患者心血管事件风险的关联研究. 2017."

406 "赵登旺, et al., 尿胰岛素生长因子结合蛋白7在急性肾损伤诊断中的意义. 内科急危重症杂志, 2017. 23(1): p. 48-49,56."

407 "赵绍林, 李.A.陈.A.王.A.周.A.付.A., 中性粒细胞与淋巴细胞比值在有机磷农药中毒急性肾损伤中的诊断价值. 环境与职业医学, 2019. 36(10)."

408 "赵文可, 早发冠心病的临床特点及其血清学标志物的研究. 2020."

409 "郑邵雄, Kim-1时间分辨荧光免疫分析法的建立及其在肾病中的临床应用. 2022."

410 "朱家全, N-乙酰半胱氨酸保护体外循环术后急性肾损伤及机理研究. 2007."

411 "卓杨, 尿NGAL在ACS患者介入术后对比剂肾病的早期诊断. 2015."

412 "邹艳清, ICU患者急性肾损伤的早期诊断标志物初探. 临床合理用药杂志, 2015. 8(3): p. 7-8,10."

413 "邹颖刚, et al., Clusterin 在急性肾损伤诊断中的意义. 中国实验诊断学, 2013. 17(5): p. 982,封3."

Review(110)

1 "American Transplant Congress 2007 Executive and Program Planning Committees and Abstract Review Committees. American Journal of Transplantation, 2007. 7: p. 17-586."

2 "Ars, E., et al., Consensus document on autosomal dominant polycystic kindey disease from the Spanish Working Group on Inherited Kindey Diseases. Review 2020. Nefrología (English Edition), 2022. 42(4): p. 367-389."

3 "Benny, P.A., et al., A review of omics approaches to study preeclampsia. Placenta, 2020. 92: p. 17-27."

4 "Bharucha, T., et al., Mass spectrometry-based proteomic techniques to identify cerebrospinal fluid biomarkers for diagnosing suspected central nervous system infections. A systematic review. Journal of Infection, 2019. 79(5): p. 407-418."

5 "De Luca Canto, G., et al., Biomarkers associated with obstructive sleep apnea and morbidities: a scoping review. Sleep Medicine, 2015. 16(3): p. 347-357."

6 "Hersi, M., et al., Risk factors associated with the onset and progression of Alzheimer’s disease: A systematic review of the evidence. NeuroToxicology, 2017. 61: p. 143-187."

7 "Lavrentieva, A., et al., Renal replacement therapy for acute kidney injury in burn patients, an international survey and a qualitative review of current controversies. Burns, 2022. 48(5): p. 1079-1091."

8 "Mani, V., et al., Multiplexed sensing techniques for cardiovascular disease biomarkers - A review. Biosensors and Bioelectronics, 2022. 216: p. 114680."

9 "McIlroy, D.R., et al., Systematic review and consensus definitions for the Standardised Endpoints in Perioperative Medicine (StEP) initiative: renal endpoints. British Journal of Anaesthesia, 2018. 121(5): p. 1013-1024."

10 "NICE Evidence Reviews Collection, in Evidence reviews for cystatin C based equations to estimate GFR in adults, children and young people: Chronic kidney disease: Evidence review M. 2021, National Institute for Health and Care Excellence (NICE)"

11 "Ramakrishnan, P., et al., A systematic review of studies comparing potential biochemical biomarkers of frailty with frailty assessments. European Geriatric Medicine, 2017. 8(5): p. 397-407."

12 "Roberts, A. and S. Gandhi, A concise review on potential cancer biomarkers and advanced manufacturing of smart platform-based biosensors for early-stage cancer diagnostics. Biosensors and Bioelectronics: X, 2022. 11: p. 100178."

13 "Schoenfeld, S.R., S. Kasturi, and K.H. Costenbader, The epidemiology of atherosclerotic cardiovascular disease among patients with SLE: A systematic review. Seminars in Arthritis and Rheumatism, 2013. 43(1): p. 77-95."

14 "Shaw, A., Update on acute kidney injury after cardiac surgery. The Journal of Thoracic and Cardiovascular Surgery, 2012. 143(3): p. 676-681."

15 "Slater, M.B., et al., A systematic review of RIFLE criteria in children, and its application and association with measures of mortality and morbidity. Kidney International, 2012. 81(8): p. 791-798."

16 "Sun, H.Z., G. Plastow, and L.L. Guan, Invited review: Advances and challenges in application of feedomics to improve dairy cow production and health. Journal of Dairy Science, 2019. 102(7): p. 5853-5870."

17 "Swedish Council on Health Technology, A., SBU Systematic Review Summaries, in Methods to Estimate and Measure Renal Function (Glomerular Filtration Rate): A Systematic Review. 2013, Swedish Council on Health Technology Assessment (SBU)"

18 "Zabka, T.S., et al., The use of emerging safety biomarkers in nonclinical and clinical safety assessment – The current and future state: An IQ DruSafe industry survey. Regulatory Toxicology and Pharmacology, 2021. 120: p. 104857."

19 "敖强国, 肾脏损伤内源性生物学标志物研究进展. 中国临床保健杂志, 2020. 23(1): p. 35-41."

20 "边娟, 孙红梅, and 李飞, 早发冠心病的相关血清学标志物研究进展. 山东医药, 2019. 59(21): p. 112-114."

21 "卜一珊, 崔.A.魏.A., 药物性肾损害诊断中肾损害标志物的研究进展. 天津药学, 2017. 29(4)."

22 "曹丽娜 and 万远太, 肝肾综合征早期生物学标志物的研究进展. 医学综述, 2020. 26(12): p. 2435-2439,2445."

23 "曾萍萍, 痛风患者早期肾损伤诊断生物标志物的研究进展. 中文科技期刊数据库（全文版）医药卫生, 2023(4)."

24 "常雪妮 and 董晨明, 新型生物标志物在危重症患者急性肾损伤早期诊断中的研究进展. 西北国防医学杂志, 2018. 39(12): p. 831-835."

25 陈嵩. 胱抑素C与心力衰竭关系研究进展. 2014.

26 "崔蓉, 魏薇, and 卜一珊, 药物性肾损害诊断中肾损害标志物的研究进展. 天津药学, 2017. 29(4): p. 62-65."

27 "崔炜, 赵.A., 1型心肾综合征的早期诊断及生物标志物研究进展. 中华肾病研究电子杂志, 2022. 11(2)."

28 "戴启宇, 宋蓓, and 霍剑锋, 胱抑素C在临床诊断肾疾病中的应用现状. 实用医药杂志, 2010. 27(05): p. 461-463."

29 "戴启宇, 王会英*, and 钱磊, 肾疾病诊断标志物 Cysc 的临床及实验室研究进展. 世界最新医学信息文摘（电子版）, 2012(5): p. 34-36."

30 "翟红艳 and 龙艳, 血清胱抑素C在糖尿病肾病早期诊断方面的研究进展. 中国临床新医学, 2011. 04(6): p. 585-588."

31 "丁国华, 徐.A.陈.A., 急性肾损伤早期诊断及预后标志物的研究进展. 广西医学, 2017. 39(2)."

32 "董晨明, 常.A., 新型生物标志物在危重症患者急性肾损伤早期诊断中的研究进展. 西北国防医学杂志, 2018. 39(12)."

33 "杜跃亮, 闫艳芳, and 张翠翠, 前列地尔脂微球注射液延缓早中期慢性肾脏病进展的疗效分析. 中国实用医药, 2012. 17(17): p. 180-181."

34 "范卫华, 胱抑素C的检测方法及临床应用进展. 山东医药, 2009. 49(43): p. 116-117."

35 "高翔羽, 王.A.苏.A., 新生儿急性肾损伤生物标志物研究现状. 中华妇幼临床医学杂志（电子版）, 2021. 17(1)."

36 "郭巾瑜, 帕金森病进展过程中的血清胱抑素C水平变化研究. 2016."

37 "韩飞 and 李文华, 对比剂急性肾损害的早期生物学标志物研究进展. 医学综述, 2010. 16(18): p. 2736-2739."

38 "韩飞, 对比剂急性肾损害的早期生物学标志物研究进展. 医学综述, 2010. 16(18): p. 2736-2739."

39 "韩捷, 李.A.江.A.李.A.王.A.肖.A., 痛风患者早期肾损伤诊断生物标志物的研究进展. 山东医药, 2019. 59(14)."

40 "何奔, 张.A.沈.A., 胱抑素C作为对比剂肾损伤的早期诊断生物标志物的研究进展. 上海交通大学学报（医学版）, 2015. 35(8)."

41 "何庆南, 李.A.杨.A.党.A., 新生儿急性肾损伤研究进展. 中华实用儿科临床杂志, 2014. 29(17)."

42 "侯振江 and 魏明竟, 胱抑素C及其检测方法研究进展. 国际检验医学杂志, 2007. 28(11): p. 1013-1015."

43 "黄富亮, 急性肾损伤的新型生物标志物研究进展. 医学检验与临床, 2018. 29(2): p. 34-36,33."

44 "黄江南, 蒙.A.廖.A.唐.A.韦.A.刘.A., 血清胱抑素C在不同疾病中的应用研究进展. 广西医学, 2023. 45(23)."

45 "黄中坚, et al., 重症监护病房诊断重症急性肾损伤的研究进展. 大医生, 2021. 6(12): p. 104-106."

46 "贾克刚, 李.A., 急性肾损伤标志物及其在心血管相关肾病中应用的研究进展. 实用检验医师杂志, 2015. 7(2)."

47 "贾维坤, 于.A., 心脏手术相关急性肾损伤的新型生物标志物研究进展. 河北医药, 2020. 42(3)."

48 "姜丹, 汪.A., 胱抑素C在神经系统疾病中的作用研究进展. 现代医药卫生, 2016. 32(2)."

49 "金鑫 and 李方毅, 血清胱抑素C的研究进展. 医学信息, 2013(19): p. 510-510."

50 "孔蕾, et al., 血清胱抑素C水平与糖尿病视网膜病变相关研究进展. 世界最新医学信息文摘, 2022. 22(74): p. 36-41."

51 "李放, 李.A.苑.A., 对比剂肾病早期肾损伤生物学标志物研究进展. 山东医药, 2015. 55(3)."

52 "李建秋, et al., 新生儿急性肾损伤研究进展. 中华实用儿科临床杂志, 2014. 29(17): p. 1345-1348."

53 "李峻岭, 张.A., 肾损伤早期诊断标志物的研究现状. 中华临床医师杂志（电子版）, 2012. 6(19)."

54 "李萍 and 韦华, 胱抑素C与糖尿病认知功能障碍关系研究进展. 右江民族医学院学报, 2022. 44(2): p. 293-296."

55 "李顺宝, 苑国富, and 李放, 对比剂肾病早期肾损伤生物学标志物研究进展. 山东医药, 2015(3): p. 95-97."

56 "李文华, 韩.A., 对比剂急性肾损害的早期生物学标志物研究进展. 医学综述, 2010. 16(18)."

57 "李雪梅, 马.A., 法布雷病相关生物标志物新进展. 中华内科杂志, 2022. 61(3)."

58 "李雪媛, et al., 痛风患者早期肾损伤诊断生物标志物的研究进展. 山东医药, 2019. 59(14): p. 99-102."

59 "李引, 早期急性肾损伤标志物研究进展. 2015."

60 "李永姝 and 贾克刚, 急性肾损伤标志物及其在心血管相关肾病中应用的研究进展. 实用检验医师杂志, 2015. 7(02): p. 115-118."

61 "李雨薇, et al., 新生儿急性肾损伤生物标志物的研究进展. 当代医药论丛, 2023. 21(11): p. 69-72."

62 刘莉莉. AKI生物学标志物研究进展. 2012.

63 "刘荣静, et al., 胱抑素C诊断急性冠脉综合征的价值评价. 中国实验诊断学, 2010. 14(3): p. 429-431."

64 "刘伟敬, 许.A.王.A.许.A., AKI生物学标志物新进展. 中国医学创新, 2013. 10(28)."

65 "刘孝琴, 王.A.陈.A., 急性肾损伤新型早期生物标志物的研究进展. 牡丹江医学院学报, 2022. 43(2)."

66 "马杰 and 李雪梅, 法布雷病相关生物标志物新进展. 中华内科杂志, 2022. 61(3): p. 336-341."

67 "马晓峰, 陈.A., 血清胱抑素C与心房颤动关系的最新研究进展. 临床医学进展, 2023. 13(2)."

68 "梅长林, 肖.A.盛.A.孙.A., 急性肾损伤早期诊断生物标志物研究进展. 现代生物医学进展, 2013. 13(16)."

69 "蒙玉民, et al., 血清胱抑素C在不同疾病中的应用研究进展. 广西医学, 2023. 45(23): p. 2905-2909."

70 "牟迎东, 张琳琳, and 张培荣, 急性肾损伤早期生物学标志物研究的新进展. 中华危重症医学杂志（电子版）, 2015(3): p. 191-196."

71 "穆心苇, 薛.A.牛.A., 急性肾损伤生物学标志物在心脏外科术后的研究进展. 国际外科学杂志, 2013. 40(10)."

72 "潘柏申, 王.A.郭.A., 估算肾小球滤过率在慢性肾脏疾病诊疗中的研究现状. 中华检验医学杂志, 2014. 37(12)."

73 "孙林, 郭玮, and 潘柏申, 急性肾损伤相关标志物的研究进展. 中华检验医学杂志, 2014(6): p. 425-429."

74 "谭向来, 心脏手术后急性肾损伤相关生物学标志物的最新研究进展. 医学综述, 2014. 20(1): p. 72-74."

75 "陶怡婷, 急性肾损伤早期标志物及其在体外循环术后应用的研究进展. 医学综述, 2013. 19(6): p. 986-988."

76 "汪冰, 胱抑素C在神经系统疾病中的作用研究进展. 现代医药卫生, 2016. 32(2): p. 214-216."

77 "王蓓丽, 郭玮, and 潘柏申, 估算肾小球滤过率在慢性肾脏疾病诊疗中的研究现状. 中华检验医学杂志, 2014. 37(12): p. 899-902."

78 "王惠颖, 苏敏, and 高翔羽, 新生儿急性肾损伤生物标志物研究现状. 中华妇幼临床医学杂志（电子版）, 2021. 17(1): p. 7-14."

79 "王建彬 and 邱龄, 胱抑素C与冠心病关系的研究进展. 中西医结合心脑血管病杂志, 2021. 19(5): p. 783-786."

80 "王军, 杨.A.覃.A., 急性肾损伤的早期生物学标志物的研究现状. 热带医学杂志, 2013. 13(8)."

81 "王来亮, 罗群, and 周芳芳, 蛋白质组学在急性肾损伤尿液标志物研究中的进展. 中华危重病急救医学, 2015(5): p. 395-397."

82 "王立军, 赵.A., 急性肾损伤新型早期诊断标志物研究进展及评价. 医学综述, 2010. 16(19)."

83 "王明秋 and 李香玲, 造影剂急性肾损伤新型早期诊断标志物的研究现状. 实用医学杂志, 2016. 32(18): p. 3103-3104,3105."

84 "王鹏飞, 沈玲红, and 何奔, 急性肾损伤生物标志物研究进展. 内科理论与实践, 2010. 5(3): p. 268-272."

85 "王婷婷, 陈丽梅, and 刘孝琴, 急性肾损伤新型早期生物标志物的研究进展. 牡丹江医学院学报, 2022. 43(02): p. 138-141."

86 "王小龙, et al., 老年急性肾损伤诊断与防治研究进展. 人民军医, 2018. 61(11): p. 1051-1055,1059."

87 "王玉萍, et al., 对比剂肾病研究进展. 介入放射学杂志, 2017. 26(6): p. 572-575."

88 "王子暄, et al., 心衰生物标志物的研究进展. 河南大学学报（医学版）, 2021. 40(6): p. 452-458."

89 "魏明竞（审校）, 侯.综.A., 胱抑素C及其检测方法研究进展. 国际检验医学杂志, 2007. 28(11)."

90 "肖燎原, et al., 急性肾损伤早期诊断生物标志物研究进展. 现代生物医学进展, 2013. 13(16): p. 3183-3185."

91 "徐亮, 陈星华, and 丁国华, 急性肾损伤早期诊断及预后标志物的研究进展. 广西医学, 2017. 39(02): p. 238-242."

92 "许琛, et al., AKI生物学标志物新进展. 中国医学创新, 2013(28): p. 162-164."

93 "薛寅莹, 牛永胜, and 穆心苇, 急性肾损伤生物学标志物在心脏外科术后的研究进展. 国际外科学杂志, 2013. 40(10): p. 677-680."

94 "闫亚亚, 刘.A.荣.A., 心肾综合征新型生物学标志物研究进展. 临床医药实践, 2021. 30(5)."

95 "杨春华 and 杜荣花, 血清胱抑素C临床应用的研究进展. 河北医药, 2015. 37(15): p. 2355-2356."

96 "杨帆, 周.A., 糖尿病肾病损伤标志物的研究进展. 华夏医学, 2019. 32(1)."

97 "杨剑辉（审校）, 王.综.A., 急性肾损伤早期标志物诊断价值评价. 国际泌尿系统杂志, 2012. 32(1)."

98 "杨俊生, 覃学勇, and 王军, 急性肾损伤的早期生物学标志物的研究现状. 热带医学杂志, 2013. 13(08): p. 1057-1060."

99 "尹辉, et al., 糖尿病肾病血清胱抑素C检测意义及益气养阴活血法干预的研究进展. 河北中医, 2012. 34(4): p. 633-636."

100 "于洋 and 贾维坤, 心脏手术相关急性肾损伤的新型生物标志物研究进展. 河北医药, 2020. 42(3): p. 457-462."

101 "张淑香 and 李峻岭, 肾损伤早期诊断标志物的研究现状. 中华临床医师杂志（电子版）, 2012. 6(19): p. 5976-5978."

102 "张维峰, 沈玲红, and 何奔, 胱抑素C作为对比剂肾损伤的早期诊断生物标志物的研究进展. 上海交通大学学报(医学版), 2015. 35(08): p. 1229-1233."

103 "章霞 and 柯永胜, 胱抑素C与冠心病关系的研究进展. 国际老年医学杂志, 2014. 35(2): p. 84-87."

104 "赵飞, 急性肾损伤新型早期诊断标志物研究进展及评价. 医学综述, 2010. 16(19): p. 2927-2930."

105 "赵红亮 and 崔炜, 1型心肾综合征的早期诊断及生物标志物研究进展. 中华肾病研究电子杂志, 2022. 11(2): p. 109-113."

106 "赵万霞, et al., 糖尿病肾病早期标志物研究新进展. 国际内分泌代谢杂志, 2018. 38(3): p. 192-195."
[truncated: 50,301 more chars]
